# Exploration of TRPM8 Binding Sites by β-Carboline-Based Antagonists and Their In Vitro Characterization and In Vivo Analgesic Activities

**DOI:** 10.1021/acs.jmedchem.0c00816

**Published:** 2020-07-29

**Authors:** Alessia Bertamino, Carmine Ostacolo, Alicia Medina, Veronica Di Sarno, Gianluigi Lauro, Tania Ciaglia, Vincenzo Vestuto, Giacomo Pepe, Manuela Giovanna Basilicata, Simona Musella, Gerardina Smaldone, Claudia Cristiano, Sara Gonzalez-Rodriguez, Asia Fernandez-Carvajal, Giuseppe Bifulco, Pietro Campiglia, Isabel Gomez-Monterrey, Roberto Russo

**Affiliations:** † Department of Pharmacy, University of Salerno, Via G. Paolo II 132, 84084 Fisciano, Salerno, Italy; ‡ Department of Pharmacy, University Federico II of Naples, Via D. Montesano 49, 80131 Naples, Italy; § IDiBE, Universitas Miguel Herna′ndez, Avda de la Universidad, 032020 Elche, Spain; ∥ European Biomedical Research Institute (EBRIS), Via S. De Renzi 50, 84125 Salerno, Italy

## Abstract

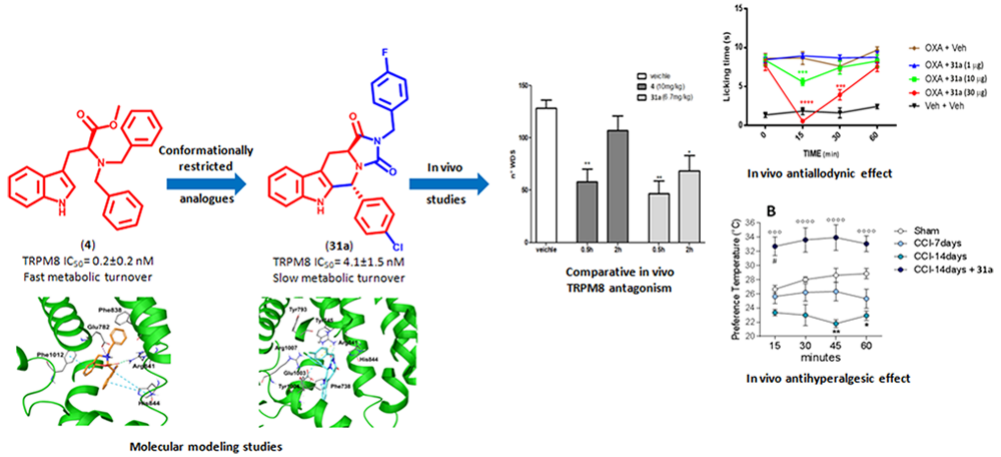

Transient receptor potential melastatin 8 (TRPM8) ion channel represents a valuable pharmacological option for several therapeutic areas. Here, a series of conformationally restricted derivatives of the previously described TRPM8 antagonist *N*,*N*′-dibenzyl tryptophan **4** were prepared and characterized in vitro by Ca^2+^-imaging and patch-clamp electrophysiology assays. Molecular modeling studies led to identification of a broad and well-defined interaction network of these derivatives inside the TRPM8 binding site, underlying their antagonist activity. The (5*R*,11a*S*)-5-(4-chlorophenyl)-2-(4-fluorobenzyl)-5,6,11,11a-tetrahydro-1*H*-imidazo[1′,5′:1,6]pyrido[3,4-*b*]indole-1,3(2*H*)-dione (**31a**) emerged as a potent (IC_50_ = 4.10 ± 1.2 nM), selective, and metabolically stable TRPM8 antagonist. In vivo, **31a** showed significant target coverage in an icilin-induced WDS (at 11.5 mg/kg ip), an oxaliplatin-induced cold allodynia (at 10–30 μg sc), and CCI-induced thermal hyperalgesia (at 11.5 mg/kg ip) mice models. These results confirm the tryptophan moiety as a solid pharmacophore template for the design of highly potent modulators of TRPM8-mediated activities.

## Introduction

The transient receptor potential melastatin type 8 (TRPM8) is a member of the thermo-TRP family^1^ of polymodal, nonselective, and Ca^2+^ permeable ion channel, identified as the physiological sensor of environmental cold.^2^ TRPM8 is activated by a range of innocuous to noxious cold temperatures (10–28 °C),^2c,3^ natural and synthetic cooling agent,^2c,4^ membrane depolarization,^5^ changes in extracellular osmolarity^6^ and phosphatidylinositol 4,5-biphosphate (PIP_2_).^7^

**Chart 1:**
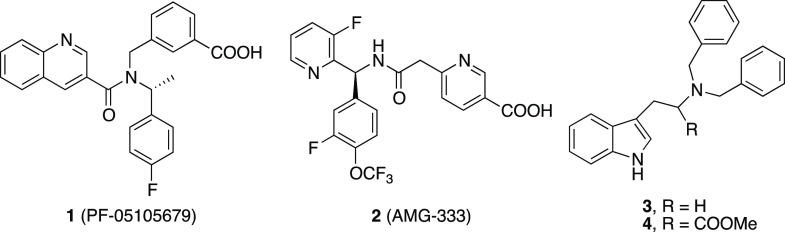
Structures of Some TRPM8 Antagonists

Originally expressed in a prostate cancer cell line,^8^ TRPM8 was subsequently detected in a subset of primary afferent neurons in the dorsal root ganglion (DRG) and trigeminal ganglia (TG),^2c,9^ which innervate cold highly sensitive tissues, such as skin, oral cavity epithelium, teeth, tongue, and cornea.^9a,10^ TRPM8 is also expressed in visceral tissues innervated by pelvic or vagal nerves,^11^ several tumor cells,^12^ macrophages,^13^ and different regions in rodents brain.^14^ Regulation of the TRPM8-expression and/or -morphological changes in pathological processes involving these tissues may represent a new opportunity for the therapeutic intervention in pain, cancer, inflammation, and metabolic diseases, among others.^15^ In particular, there is a large body of evidence that correlates the hypersensitivity to cold, typical of neuropathic pain models, after nerve injury or oxaliplatin-treatment with augmented expression of TRPM8 in sensory neurons,^16^ suggesting that blocking the channel can be a suitable approach to treat these pain conditions. In fact, TRPM8 gene deletion^17^ or pharmacological inhibition of the channel in both animal models and humans is correlated with a decreased cold hypersensitivity in neuropatic,^17,18^ chronic visceral pain,^19^ and also migraine.^20^ Considering these findings and the potential activity of TRPM8 antagonists also in cancer and other pathologies,^21^ it is easy to understand the effort of the academic groups and pharmaceutical/biotech companies to develop potent and selective TRPM8 modulators.^22^ To date, two antagonists, the quinoline-3-carboxamido derivative PF-05105679^18a^ and the amino-2-oxoethyl nicotinic acid derivative AMG-333^20^ (Chart 1), have been evaluated for the treatment of cold related pain and migraine, respectively, although they have not passed phase I studies. In 2017, two undisclosed structures, named RQ 00434739^23^ and Ice 3682,^18d^ have reached clinical trials for the treatment of neuropathic pain in Japan and Israel, respectively.

In the past years, the information obtained through mutagenesis experiments^24^ and molecular modeling studies^25^ on the structure–function of TRPM8 channels has suggested the existence of several independent and overlapping pockets in the TRPM8 binding site able to interact with different antagonist chemotypes.^15c,22,26^ This makes it difficult to rationalize pharmacological results, particularly in the context of neuropathic pain, where also agonists of TRPM8 are able to induce analgesia,^27^ as well as to define the molecular basis for TRPM8 antagonism. Recently the group of Lee^28a,28b^ resolved the structure of full-length TRPM8 protein from the collared flycatcher *Ficedula albicollis* (TRPM8_FA_) using cryoelectron microscopy. The network of interactions generated from the TRPM8_FA_/menthol, icilin, or lipids lays the structural basis for the design and identification of potent and selective ligands. Importantly, in 2019 two novel structures of TRPM8 complexed with the two antagonists AMTB and TC-I 2014 (PDB codes 6O6R and 6O72) were released, thus providing further important structural details for aiding the identification of TRPM8 modulators.^28c^

In this context, we have also recently generated a homology model of human TRPM8 using the TRPM8_FA_ structure as template to rationalize the potent antagonist activity showed by tryptamine^29^ and tryptophan-based^18b^ TRPM8 modulators (**3** and **4**, Chart 1). In patch-clamp recordings, these compounds were more potent (IC_50_ = 367 and 0.2 nM, respectively) than the well-known TRPM8 antagonist BCTC. In vivo, compound **4** attenuated icilin-induced shaking behaviors and reversed oxaliplatin-induced cold allodynia in mice model. Docking studies disclosed the voltage sensor region (VSLD), in the transmembrane segments portion S1–S4, as a possible binding site for these derivatives, highlighting the ability of both compounds to affect the network of interactions established between TM (S1–S4) and the TRP domain at C-terminal of the channel subunits.

In order to deepen the structural requirements necessary for the TRPM8 antagonist activity of these indol-based derivatives, we designed and synthesized a new series of conformationally restricted analogues of **4** pursuing a double aim: (a) to increase the metabolic stability of our lead compound by decreasing its amino acid character; (b) to explore new TRPM8/antagonist interactions leading to the potential discovery of SAR clues. In this paper, we discuss the design and synthesis of three different series of tryptophan restricted analogues of the lead compound **4**, namely, tetrahydro-β-carbolines (THBCs), THBC-based diketopiperazines, and THBC-based hydantoin derivatives, as well as the results of TRPM8 antagonist activity obtained by assays of Ca^2+^fluorescence and patch-clamp measurements. These data were rationalized by molecular modeling studies defining new structural requirements for the TRPM8 antagonist activity. Finally, the most potent compound identified was tested in three different in vivo pain models.

## Results and Discussion

### Chemistry

**Scheme 1:**
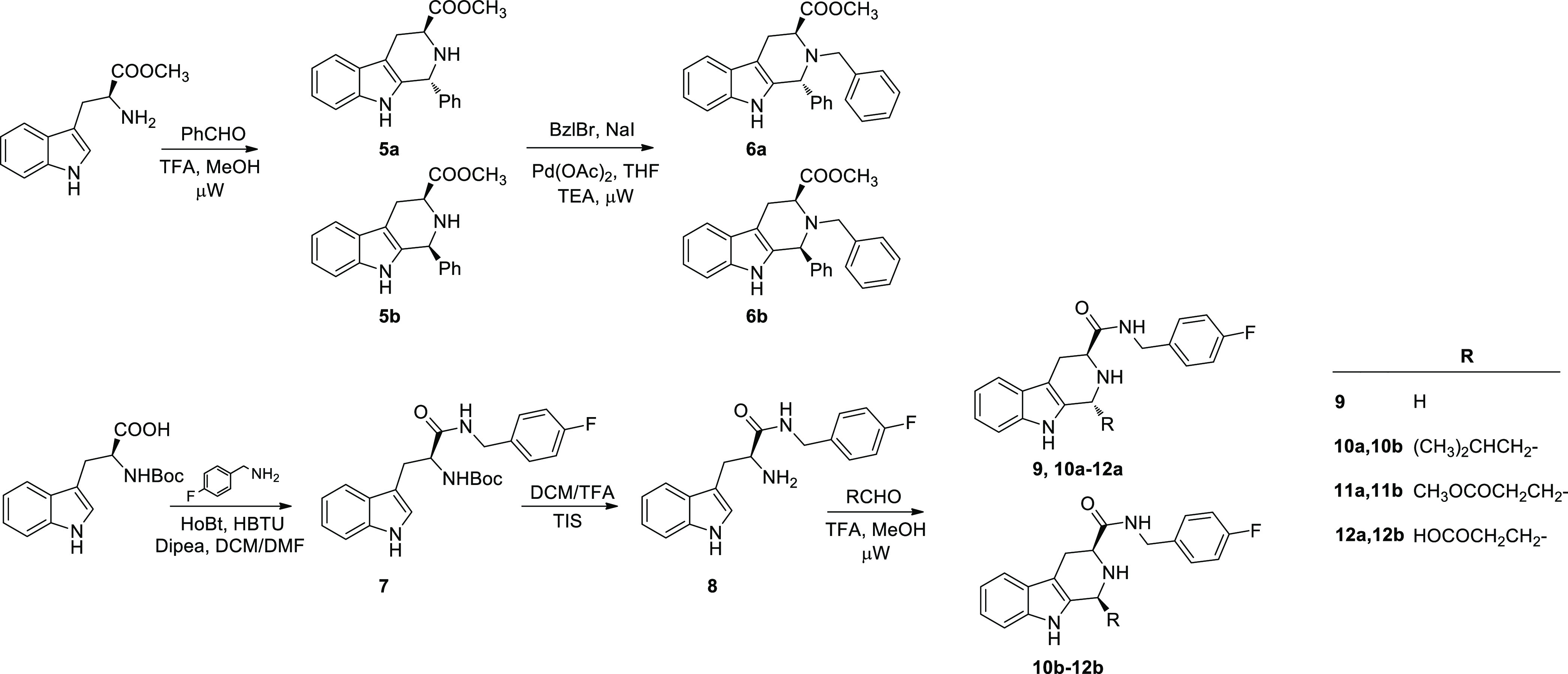
Synthesis of Substituted Tetrahydro-β-carbolines (**6a**,**b** and **10**–**12a**,**b**)

Tetrahydrobetacarbolines (THBCs) **6a**,**b**, **9**, and **10**–**12a**,**b**, were synthesized as depicted in Scheme 1.

Starting from a microwave assisted Pictet–Spengler reaction of *L*-Trp-OMe with benzaldehyde and TFA in methanol the THBC intermediates **5a**,**b** were obtained as a diasteroisomeric mixtures (2:1 *cis*:*trans*), which were resolved by flash chromatography. N-benzylation reaction of the pure diastereoisomers with benzyl bromide, sodium iodide, and triethylamine in THF using palladium acetate as catalyst and microwave irradiation led to the final *trans*-(**6a**) and *cis*-(**6b**) THBCs in 62% and 59% yields, respectively. The relative configuration for **6a** and **6b** was assigned by ROESY NMR spectra considering the cross peak between H1 and H3 that is present for **6b** (H1, δ 4.96 ppm; H3, δ 3.87 ppm, Figure S8), while it is missing in **6a** (H1, δ 5.39 ppm; H3, δ 3.87 ppm, Figure S5). Assuming that the absolute configuration for the l-tryptophan moiety is maintained in the reaction conditions, the configuration at C1 was assigned accordingly. The same key correlation was used to assign the absolute configuration of the other THBCs derivatives (**10**–**12a**,**b**). Reaction of NBoc-*L*-Trp-OH with 4-F-benzylamine, using HoBt and HBTU as coupling agents and DIPEA as base in a mixture of DCM/DMF, gave the amide intermediate **7**, which was then deprotected in DCM/TFA (3:1 v:v). The free amine **8** was subjected to a Pictet–Spengler reaction with formaldehyde or isovaleraldehyde or methyl-4-oxobutanoate or 4-oxobuthanoic acid in the above-described conditions, leading to the formation of THBC **9** (55% of yield) and the *trans*–*cis* mixtures **10**–**12a**,**b**. Flash chromatography allowed the separation of the corresponding *trans***10**–**12a** (35–43% yields) and *cis***10**–**12b** (33–43% yields) diastereoisomers. On the basis of 2D NMR correlations and considering the fixed configuration at C-3 as *S*, we assigned the configurations to the THBC *trans***10**–**12a** and *cis***10**–**12b** as (1*R*,3*S*) and (1*S*,3*S*), respectively.

**Scheme 2:**
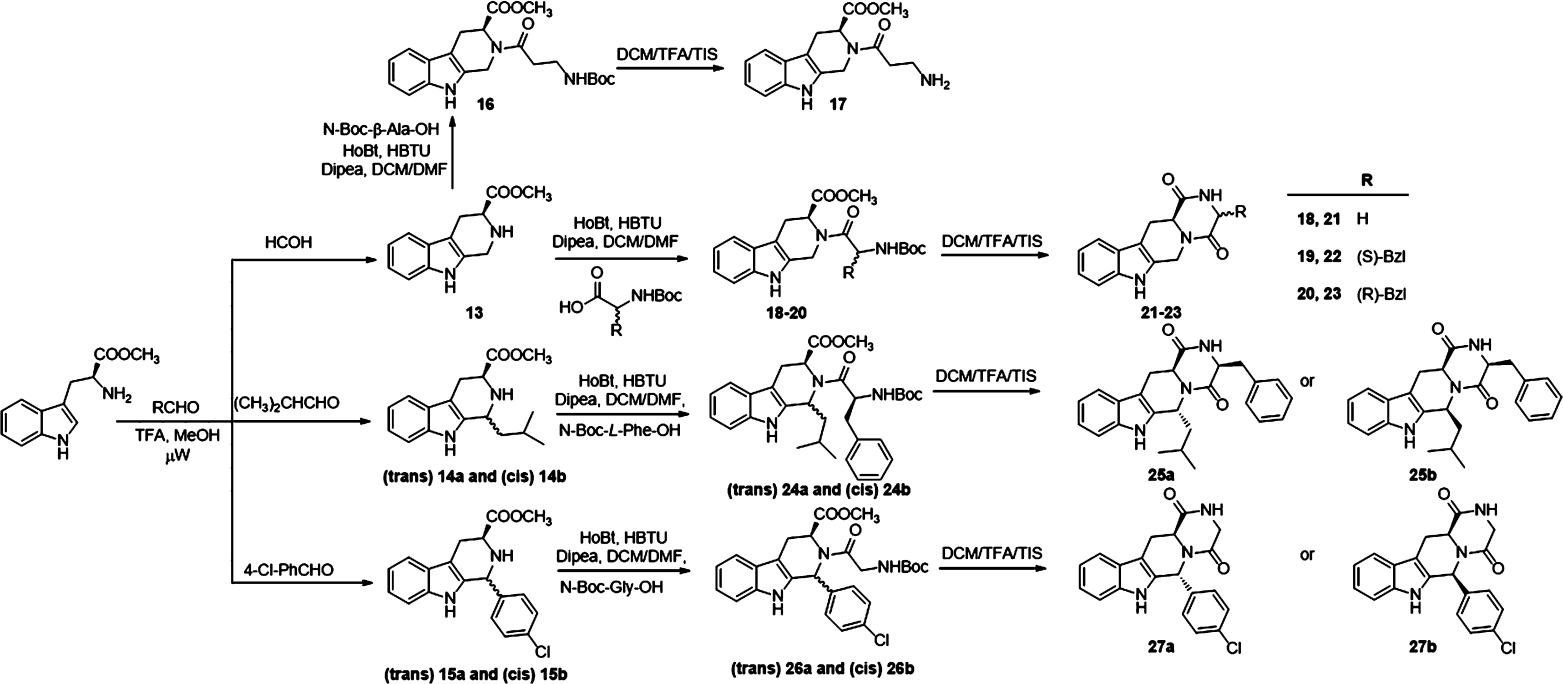
Synthesis of Tetrahydropyrazino[1′,2′:1,6]pyrido[3,4-*b*]indole-1,4(6*H*,7*H*)-dione Derivatives (**17**, **21**–**23**, **25a**, **25b**, **27a**, and **27b**)

THBC derivatives **13**, **14a**,**b,** and **15a**,**b**, were obtained following the procedure described above by reaction of *L*-Trp-OMe with formaldehyde, isovaleraldehyde, and 4-Cl-benzaldehyde, respectively (Scheme 2). Coupling of these intermediates with NHBoc-β-Ala-OH, NHBoc-Gly-OH, NHBoc-*L*-Phe-OH, or NHBoc-*D*-Phe-OH using HoBt, HBTU in DCM/DMF gave the pseudo dipeptide intermediates **16, 18**–**20**, **24a**,**b**, and **26a**,**b** in 25–71% yield, respectively. Boc-deprotection of the amino group in TFA acid medium of the derivatives **18**–**20**, **24a**,**b**, and **26a**,**b** followed by a spontaneous intramolecular cyclization provided the final THBC-based diketopiperazines **21**–**23**, **25a**,**b**, **27a**,**b** with 73–85% of yields. As expected, the treatment of **16** with TFA gave the unprotected derivative **17** in quantitative yield. In order to determine the relative configuration for the tetrahydropyrazino[1′,2′:1,6]pyrido[3,4-*b*]indole-1,4(6*H*,7*H*)-dione derivatives, the cross peak between H6 and H12a was investigated through ROESY NMR. For example, **27b** shows a cross peak between δ 6.16 ppm (H6) and δ 4.30 ppm (H12a, Figure S43) while this correlation is missing for the diastereoisomer **27a** (δ 6.67 ppm; δ 4.18 ppm, Figure S40). The absolute configuration was attributed considering the retention of the l-tryptophan chirality.

**Scheme 3:**
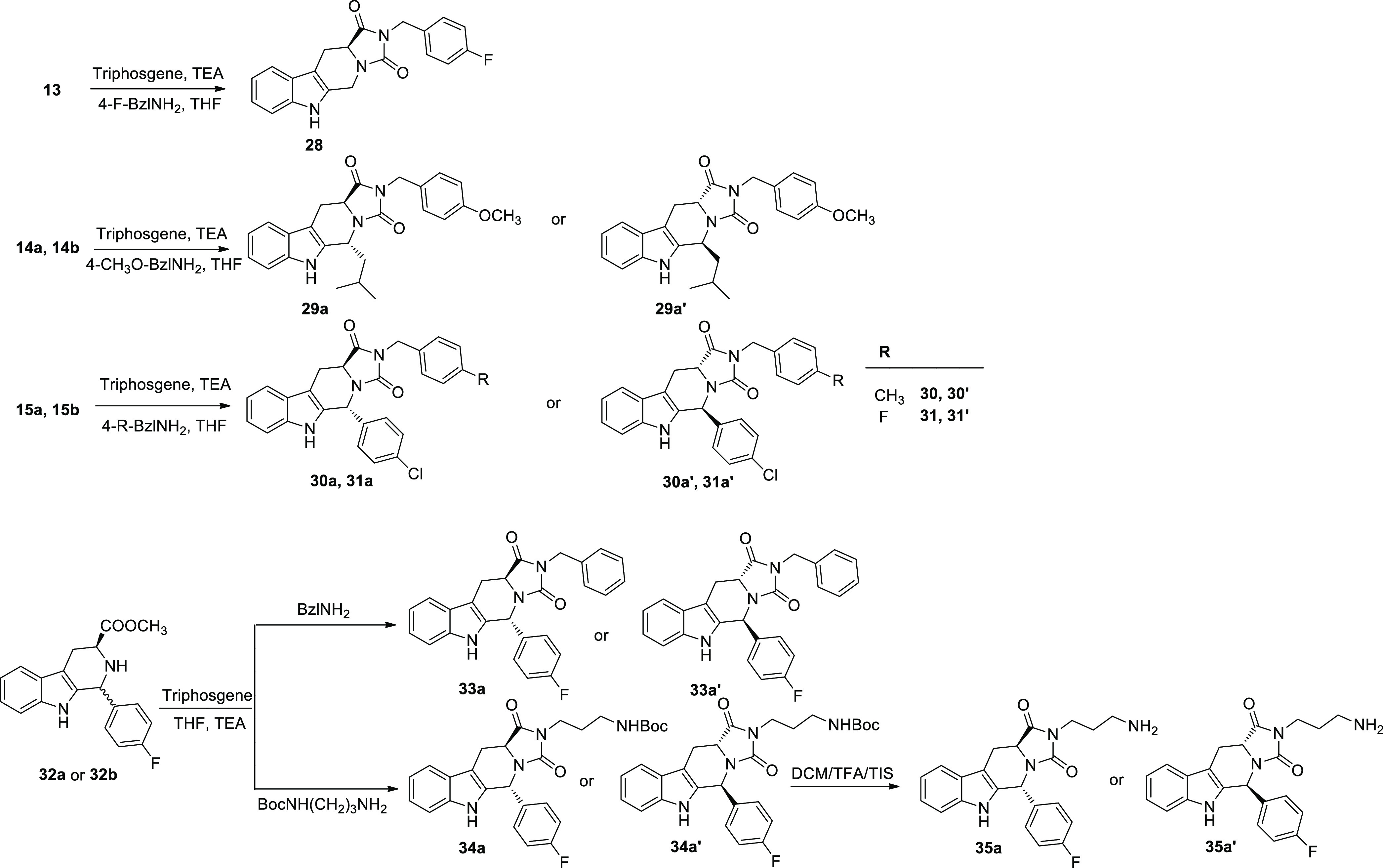
Synthesis of Tetrahydro-1*H*-imidazo[1′,5′:1,6]pyrido[3,4-*b*]indole-1,3(2*H*)-dione Derivatives (**28**, **29a**–**31a**, **33a**, **35a** and Their Enantiomers **29a′**–**31a′**, **33a**′, **35a**′)

Final THBC-based hydantoin compounds were obtained through the synthetic method reported in Scheme 3. Reaction of the starting THBCs **13**, **14a**, **14b**, **15a**, **15b** and the now synthesized **32a** and **32b** with triphosgene and different amines, such as 4-F, 4-OMe, 4-Me-benzylamine, benzylamine, and NHBoc propylendiamine in THF using TEA as base, led to the final hydantoin derivatives **28**, **29a**–**31a**, **33**–**34a**, **29a′**–**31a′**, **33**–**34a′** in one step and with 35–62% yields. NMR data, α_D_ values, and circular dichroism experiments (see Supporting Information) showed that the reaction of *trans* (1*R*,3*S*) THBCs **14a, 15a**, and **32a** originated the *trans* derivatives (5*R*,11a*S*) **29a**–**31a**, **33a**, and **34a**, while the cyclization reaction from the *cis* analogues (1*S*,3*S*) **14b**, **15b**, and **32b** led to the formation of the *trans* enantiomers (5*S*,11a*R*) **29a′**–**31a′**, **33a′**, and **34a′**. For instance, the configuration of THBCs **15a** and **15b** was assessed by ROESY NMR, investigating the correlation between H3 and H1, assuming retention of configuration for the l-tryptophan moiety. As shown in Figure S89, only compound **15b** showed the investigated correlation (H1, δ 5.25 ppm; H3, δ 3.99 ppm). After cyclization reaction of **15b** to the hydantoine derivative (**30a′**) the cross peak between the same hydrogens (H11a δ 4.30 ppm, H5 δ 6.29 ppm) was not detected (Figure S89). This is in accordance with literature^30^ that describes the epimerization at C-3 position of the (1*S*,3*S*) THBCs during the cyclization process, resulting in the formation of the most stable *trans* (5*S*,11a*R*) THBC-based hydantoin derivatives. Moreover, for all the THBC-based hydantoin enantiomeric couples, as expected, we observed the same NMR chemical shifts and opposite values for α_D_ (see Experimental Section) and specular circular dichroism spectra (Figure S89). Removal of the Boc protecting group from **34a** and **34a′** using TFA and triisopopylsilane (TIS) in dichloromethane led to the final products **35a** and **35a′**, respectively.

**Scheme 4:**
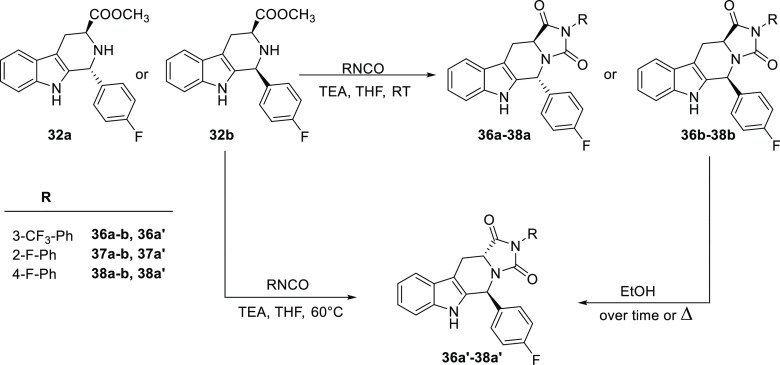
Synthesis of THBC-base 2-Arylhydantoin Derivatives (**36**–**38**, Their Enantiomers **36b**–**38b**, and Their Diastereoisomers **36a′**–**38a′**)

On the other hand, Scheme 4 reports the synthesis of the *N*-aryl hydantoin derivatives **36a**–**38a** and **36b**–**38b**. In this case, a different chemical approach is required because of the minor reactivity of anilines. Intermediates THBC **32a** and **32b** were coupled with 3CF_3_ or 2F or 4-F-phenyl isocyanate in basic medium of TEA. In these conditions, we obtained the corresponding (5*R*,11a*S*) *trans*- and (5*S*,11a*S*) *cis*-hydantoins (**36a**–**38a** and **36b**–**38b**, respectively), which were isolated and characterized by 2D NMR spectroscopy. In particular, the *cis* configuration was evidenced by the correlation between H11a and H5, corresponding to δ 4.53 ppm and δ 5.86 ppm, respectively, for compound **36b** (Figure S76). Absolute configuration was determined as described above. The formation of the *cis* intermediates, which was not observed with the *N*-benzyl or *N*-alkyl analogs, can be explained by the increased stability of the kinetic control species due to the higher rigidity of this structure. However, C11a epimerization was not suppressed and we noticed that the *cis* conformers converted to their thermodynamically more stable *trans* congeners (5*S*,11a*R*) **36a′**–**38a′**, with a conversion kinetic depending on experimental conditions. High temperatures and alcoholic solvents such as methanol and ethanol favored the conversion to the *trans* derivative, while in aqueous media at room temperature the *cis* conformers were more stable (Figure S2). Therefore, given the spontaneous trend of *cis*-hydantoins toward *trans*-conversion, we considered inappropriate the pharmacological testing of all the *cis* isomers and we decided to assay only **36b** for its pharmacological activity, due to its higher stability in water environment in comparison with its congeners **37b** and **38b**, which were almost fully converted to the *trans* isomers during 60 min regardless of the solvent used (Figure S1). In addition, the corresponding C-11a epimers **36a′**–**38a′** could be obtained directly by reaction of **32b** with the corresponding isocyanates and TEA at 60 °C for 30 min in 39–45% yields.

### Pharmacological Characterization. Screening by Ca^2+^-Imaging Assay

TRPM8 blocker activity of all synthesized compounds was tested by Ca^2+^ fluorimetric assays using HEK-293 cells stably expressing the rat isoform of TRPM8 channels, using menthol and AMTB as prototypical agonist and antagonist, respectively. All the compounds showed an antagonist activity higher than the canonical TRPM8 antagonist AMTB, although lower than the lead compound **4** with IC_50_ values in the 100–0.3 μM range (Table 1).

**Table 1:**
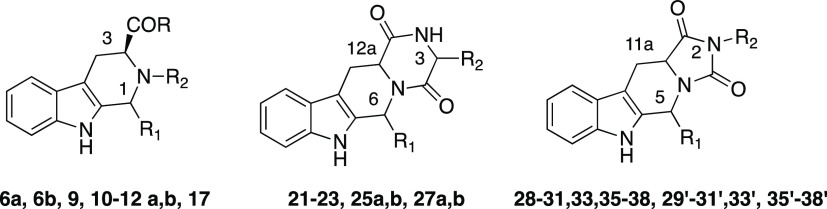
Potency of Synthesized Compounds as TRPM8 Antagonists

compd	R	R_1_	R_2_	configuration	IC_50_ (μM)^a^
**4**					0.09 ± 0.08
**6a**	OCH_3_	C_6_H_5_	CH_2_C_6_H_5_	1*R*, 3*S*	1.3 ± 0.7
**6b**	OCH_3_	C_6_H_5_	CH_2_C_6_H_5_	1*S*,3*S*	1.6 ± 0.9
**9**	NHCH_2_(4-F)Ph	H	H	3*S*	0.9 ± 0.4
**10a**	NHCH_2_(4-F)Ph	CH_2_CH(CH_3_)_2_	H	1*R*,3*S*	4.6 ± 1.3
**10b**	NHCH_2_(4-F)Ph	CH_2_CH(CH_3_)_2_	H	1*S*,3*S*	6.2 ± 1.2
**11a**	NHCH_2_(4-F)Ph	CH_2_CH_2_COOCH_3_	H	1*R*,3*S*	1.1 ± 0.5
**11b**	NHCH_2_(4-F)Ph	CH_2_CH_2_COOCH_3_	H	1*S*,3*S*	3.0 ± 1.2
**12a**	NHCH_2_(4-F)Ph	CH_2_CH_2_COOH	H	1*R*,3*S*	5.0 ± 1.2
**12b**	NHCH_2_(4-F)Ph	CH_2_CH_2_COOH	H	1*S*,3*S*	22.0 ± 1.4
**17**	OCH_3_	H	COCH_2_CH_2_NH_2_	3*S*	>100
**21**		H	H	12a*S*	11.4 ± 1.6
**22**		H	CH_2_C_6_H_5_	3*S*,12a*S*	1.6 ± 0.7
**23**		H	CH_2_C_6_H_5_	3*R*,12a*S*	0.4 ± 0.1
**25a**		CH_2_CH(CH_3_)_2_	CH_2_C_6_H_5_	3*S*,6*R,*12a*S*	4.1 ± 1.1
**25b**		CH_2_CH(CH_3_)_2_	CH_2_C_6_H_5_	3*S*,6*S,*12a*S*	1.3 ± 0.6
**27a**		4-Cl-C_6_H_4_	H	6*R*,12a*S*	1.5 ± 1.1
**27b**		4-Cl-C_6_H_4_	H	6*S*,12a*S*	1.7 ± 0.8
**28**		H	CH_2_4-(F)-C_6_H_4_	11a*S*	17.8 ± 1.2
**29a**		CH_2_CH(CH_3_)_2_	CH_2_4-(OCH_3_)C_6_H_4_	5*R*,11a*S*	2.8 ± 1.2
**29a′**		CH_2_CH(CH_3_)_2_	CH_2_4-(OCH_3_)C_6_H_4_	5*S*,11a*R*	22.9 ± 1.4
**30a**		4-(Cl)C_6_H_5_	CH_2_4-(CH_3_)C_6_H_4_	5*R*,11a*S*	0.8 ± 0.4
**30a′**		4-(Cl)C_6_H_5_	CH_2_4-(CH_3_)C_6_H_4_	5*S*,11a*R*	2.3 ± 0.8
**31a**		4-(Cl)C_6_H_5_	CH_2_4-(F)-C_6_H_4_	5*R*,11a*S*	0.5 ± 0.3
**31a′**		4-(Cl)C_6_H_5_	CH_2_4-(F)C_6_H_4_	5*S*,11a*R*	>30
**33a**		4-(F)C_6_H_5_	CH_2_C_6_H_4_	5*R*,11a*S*	6.4 ± 1.2
**33a′**		4-(F)C_6_H_5_	CH_2_C_6_H_4_	5*S*,11a*R*	17.5 ± 1.4
**35a**		4-(F)C_6_H_5_	(CH_2_)_3_NH_2_	5*R*,11a*S*	5.1 ± 1.2
**35a′**		4-(F)C_6_H_5_	(CH_2_)_3_NH_2_	5*S*,11a*R*	27.2 ± 1.4
**36a**		4-(F)C_6_H_5_	3-(CF_3_)C_6_H_4_	5*R*,11a*S*	2.8 ± 1.5
**36a′**		4-(F)C_6_H_5_	3-(CF_3_)C_6_H_4_	5*S*,11a*R*	7.8 ± 2.4
**36b**		4-(F)C_6_H_5_	3-(CF_3_)C_6_H_4_	5*S*,11a*S*	0.2 ± 0.2
**37a**		4-(F)C_6_H_5_	2-(F)C_6_H_4_	5*R*,11a*S*	4.4 ± 1.3
**37a′**		4-(F)C_6_H_5_	2-(F)C_6_H_4_	5*S*,11a*R*	5.1 ± 2.1
**38a**		4-(F)C_6_H_5_	4-(F)C_6_H_4_	5*R*,11a*S*	6.7 ± 1.2
**38a′**		4-(F)C_6_H_5_	4-(F)-C_6_H_4_	5*S*,11a*R*	7.2 ± 0.9
AMTB					7.3 ± 1.5

a Values are expressed as the mean ± standard deviation of at least three independent measurements.

In the 1,2,3-substituted THBCs (**6**, **9**–**12**, and **17**) series, the antagonist activity is conditioned by the relative configuration at position 1 when long and linear aliphatic chains are used. Thus, the *trans* derivatives (1*R*,3*S*) **11a** and **12a** are around 4 times more active than the corresponding *cis* diastereoisomers (1*S*,3*S*) **11b** and **12b** (IC_50_= 1.1 μM and 3.0 μM for the **11a** and **11b**, respectively, and 5 μM and 22 μM for **12a** and **12b**, respectively). The influence of the configurational pattern was not observed for other diastereoisomer couples bearing bulkier alkyl or planar aryl substituents in C-1 (**6a**/**6b**, **10a**/**10b**). Indeed, the unsubstituted derivative at position C-1 (**9**) had an interesting antagonistic activity with an IC_50_ value of 0.9 μM, while the restricted pseudo dipeptide Trp-β-Ala **17**, bearing a polar 3-aminopropanoic chain in position 2, was completely inactive (IC_50_ > 100 μM).

Further expansion of the structure from TBHCs to diketopiperazines (**21**–**23**, **25a**,**b**, **27a**,**b**) retained the antagonist activity. The unsubstituted TBHC-based diketopiperazine **21**, for instance, maintained good potency (11.4 μM), but the introduction of a benzyl substituent at C-3 (**22**, **23**, and **25**) significantly increased activity with IC_50_ value in the range 4–0.4 μM. In this case, the configuration at position 3 did not significantly influence compound potencies that were comparable for the (3*R*,12a*S*) diastereoisomer **23** and its 3-epimer (3*S*,12a*S*) **22** (IC_50_ = 1.6 ± 0.7 μM and 0.4 ± 0.1 μM, respectively).

The *trans* derivative **25a** (3*S*,6*R*,12a*S*) bearing the isobutyl moiety at C-6 was significantly less potent than its *cis* diastereoisomer **25b** (3*S*,6*S,*12a*S*) (IC_50_ = 4.1 ± 1.1 μM vs 1.3 ± 0.6 μM), while the introduction of the 4-Cl-phenyl moiety at position C-6 (**27**) was well tolerated and no differences in potency were evidenced between the 6*R*,12a*S* and the 6*S*,12a*S* isomers (**27a** and **27b**, IC_50_ = 1.5 ± 1.1 μM and 1.7 ± 0.8 μM, respectively).

Finally, the diketopiperazines ring was simplified to the five-membered hydantoin system. For this series (**28**, **29a**–**31a**, **33a**, **35a**, **36a**–**38a**, **36b** and their enantiomers **29a′**–**31a′**, **33a′**, **35a′**–**38a′**) the most active compounds **30a** and **31a**, with IC_50_ in the range 0.5–0.8 μM, feature an aromatic group at C-5 position and a benzyl group at N-2 and retain the *trans* configuration, 5*R*,11a*S*. The unsubstituted compound **28** and the C-6 alkyl derivative **29a** showed reduced potency. On the other hand, the *N*-benzyl *trans* isomers *(*5*S*,11a*R*) **29a′**–**31a′**, **33a′**, **35a′**, were less active than their corresponding *trans* 5*R*,11a*S* enantiomers. This difference was not statistically significant for the *N*-aryl derivatives (**37a**/**37a′** and **38a**/**38a′**) except for the compound **36a** (5*R*,11aS), containing a 3-trifluoromethyphenyl substituent at N2, which was about 3-fold more potent than its enantiomer **36a′** (5*S*,11a*R*) (IC_50_ = 2.8 μM and 7.8 μM, respectively). Compound **36b**, the only tested *cis* derivative of this series, showed a remarkable higher potency (IC_50_ = 0.2 μM) than its two *trans* analogues **36a** and **36a′**.

### Patch-Clamp Electrophysiology Assay

Functional assay identified derivatives **6a**, **9**, **11a**, **23**, **31a**, and **36b** to be among the most effective and potent TRPM8 antagonist compounds with IC_50_ values in the submicromolar range. To provide direct evidence for this activity, these derivatives were tested in HEK-293 cells transiently expressing the human TRPM8 isoform by whole-cell voltage clamp experiments. Moreover, we decided to perform whole-cell voltage clamp experiments also for compounds **12a** and **31a′** in order to further highlight the pharmacophoric properties of the ester group in **11a** and of the stereocenters of **31a**. As shown in Table 2, the well-known TRPM8 antagonist BCTC (300 nM), used as reference, produced a complete inhibition of menthol-gated TRPM8 currents, with an IC_50_ of 501 nM. THBC-based diketopiperazine **23** and the hydantoin derivatives **31a** have concentration-dependent antagonistic activity, showing IC_50_ of 6.57 ± 1.21 nM and of 4.10 ± 1.52 nM, respectively. The THBC **6a** and **9** showed decreased potency. The propanoic ester derivative **11a**, identified as a potent inhibitor of menthol-induced increase of intracellular Ca^2+^ levels (IC_50_ = 0.8 μM), antagonized the effect of menthol with an EC_50_ of 15.41 nM, while its acid free analogue **12a** inhibited only 34% of the menthol-induced current at the maximum concentration of 300 nM. To determine the role of the relative configuration at the stereocenters in the hTRPM8-blocking activity of compound **31a**, the pharmacological effect of its 5*S*,11a*R* enantiomer, namely, **31a′**, was also investigated. As shown in Table 2, **31a′** weakly inhibited menthol-induced currents showing very weak efficacy (11% inhibition) when compared to the 5*R*,11a*S* enantiomer, therefore confirming the crucial role of the configurations in the pharmacological properties of this series of compounds.

**Table 2:**
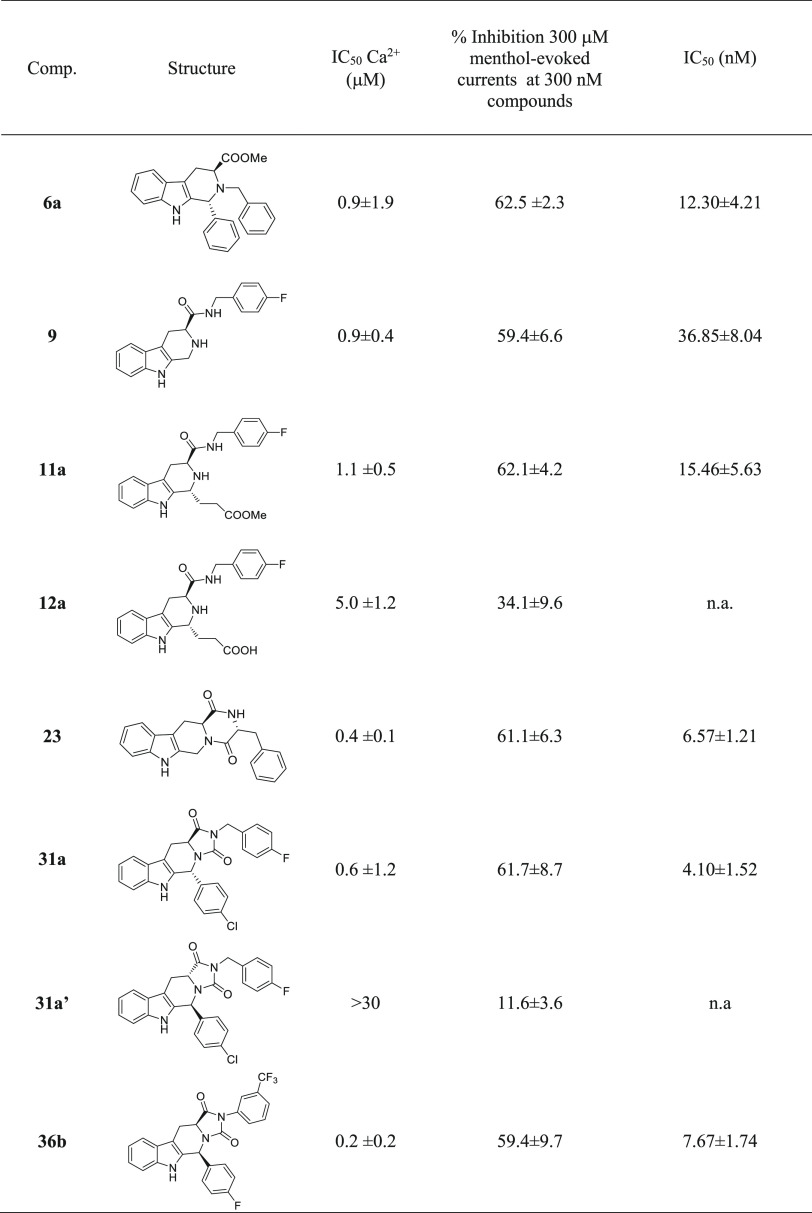
Full in Vitro Pharmacological Characterization for Selected Compounds

The activity of compound **36b**, which proved to be a powerful antagonist of TRPM8 in Ca^2+^ fluorimetric assay, was confirmed by patch clamp experiment with an IC_50_ of 7.67 nM, and an inhibition efficacy of the menthol evoked currents of 59.4%. In light of the reported spontaneous epimerization of the *cis* isomer **36b** to its *trans* congener (**36a′**) we hypothesized that the *cis*-isomer contributed mainly to this pharmacological activity. Thus, **36b** was assayed in a time course stability test, and results confirmed that the percentage of epimerization was negligible during patch-clamp electrophysiology assays (Figure S1).

### Selectivity Studies

The most potent compounds identified by patch clamp studies (**6a**, **9**, **11a**, **23**, **31a**, and **36b**) were subjected to further in vitro characterization by assessment of their selectivity toward TRPV1, TRPA1, and Na_v1.7_ channels by calcium fluorimetric experiments. TRPV1 and TRPA1 channels belong to the TRP superfamily and share a high degree of homology with TRPM8.^1^ On the other hand, Na_v1.7_ channels are reported to be involved in several neuropathic pain pathways, also modulated by TRPM8.^31^ All the derivatives were unable to modulate these channels, showing no activity as agonists or antagonists. Only compounds **6a** and **9** showed a negligible antagonistic activity over Na_v1.7_ with IC_50_ > 10 μM (Figure S2).

### Molecular Modeling and Structural Rationale

**Figure 1:**
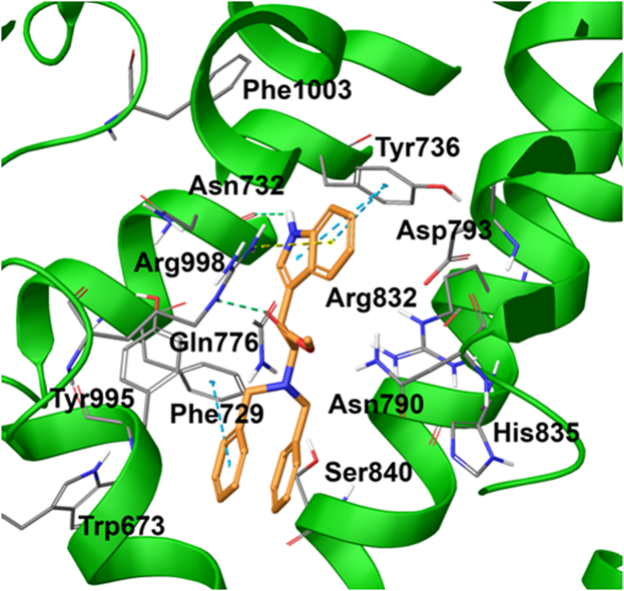
Compound **4** (colored by atom type; C orange, N blue, O red, polar H light gray) in docking with TRPM8 (represented in green ribbons; residues colored by atom type; C gray, N blue, O red, polar H light gray) in the TC-I 2014 ligand binding site. H bonds are represented with green dotted lines, π–cation interactions with yellow dotted lines, and π–π interactions with light blue dotted lines (PDB code 6O72).

The TRPM8 three-dimensional structures complexed with the two antagonists AMTB and TC-I 2014 (PDB codes 6O6R and 6O72) released by Diver et al. in 2019 revealed new important details for developing potential modulators of this protein.^28c^ The preliminary analysis and superposition of both of the TRPM8 structures revealed a very similar protein architecture when bound with the two different antagonists. Starting from these premises, the binding mode of the lead compound **4** was first re-evaluated by considering the TC-I 2014-bound TRPM8 protein structure (PDB code 6O72), chosen as reference system since it featured a better resolution if compared with that originally complexed with AMTB (PDB code 6O6R). In particular, the obtained docking poses of the lead compound **4** revealed a binding mode different from what was reported in the original paper,^43^ in which an homology modeling structure of the protein was accounted. Indeed, in the TC-I 2014-bound protein structure (PDB code 6O72), compound **4** adopted a particular shape in which one aromatic function was in front of another one, establishing an intramolecular π–π stacking interaction. Specifically, the aromatic functions of **4** were π–π stacked with several residues stabilizing the ligand/protein complex and allowing a large set of additional interactions, such as H-bond contacts. Indeed, the indole function of **4** was involved in both π–π stacking (with Tyr736) and π–cation (with Arg998) interactions, whereas one benzyl function also established an edge-to-face π–π stacking with Phe729 (Figure 1). Also, H-bonds were detected for compound **4** with Asn732 and Gln776 (Figure 1).

In order to shed light about the possible mechanism of action of the reported β-carboline-based TRPM8 antagonists, molecular docking calculations (Glide software) were performed. With the aim of rationalizing the molecular basis behind the different antagonistic activity of the tested molecules, we specifically investigated the predicted protein–ligand complexes related to most representative compounds **6a**, **9**, **11a**, **11b**, **12a**, **12b**, **23**, **31a**, **31a′**, **36a**, **36a′**, **36b**. In this way, we investigated both the influence of the molecular architecture, namely, accounting the tetrahydro-β-carboline (**6a**, **9**, **11a**, **11b**, **12a**, **12b**), tetrahydropyrazino[1′,2′:1,6]pyrido[3,4-*b*]indole-1,4(6*H*,7*H*)-dione (**23**), tetrahydro-1*H*-imidazo[1′,5′:1,6]pyrido[3,4-*b*]indole-1,3(2*H*)-dione (**31a**, **31a′**, **36a**, **36a′**, **36b**) scaffolds while also considering the effects of the different substituents as well as the impact of the specific stereoarrangements for the three chemotypes on the observed biological activity.

**Figure 2:**
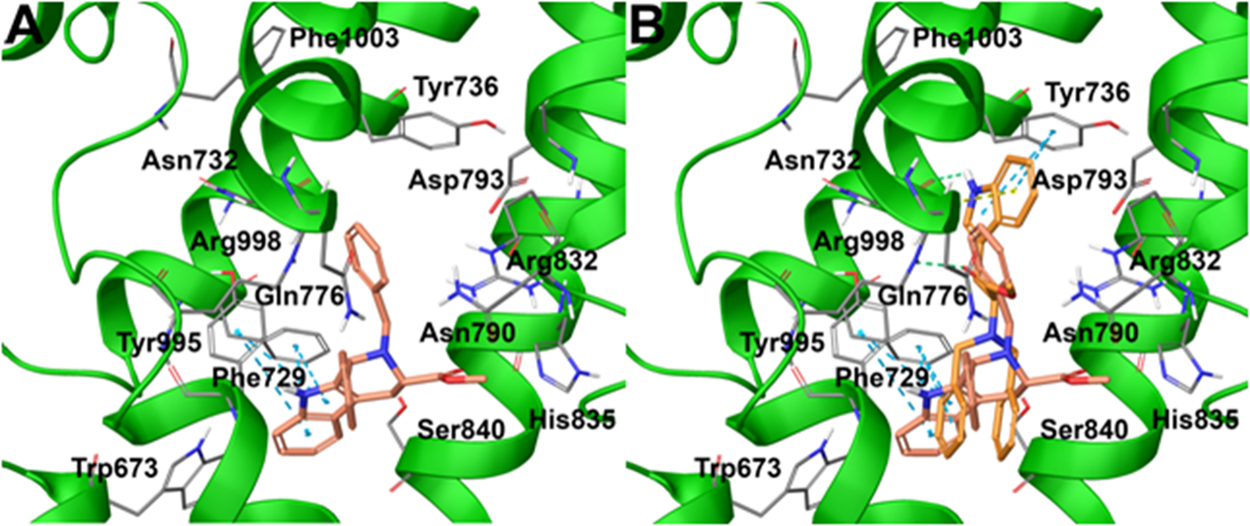
(A) Predicted binding modes of **6a** (colored by atom type, C pink) in docking with TRPM8 (represented in green ribbons; residues colored by atom type; C gray, N blue, O red, polar H light gray) and (B) superposition between the predicted binding modes of **6a** and **4** into the TRPM8 TC-I 2014 ligand binding site. H bonds are represented with green dotted lines, π–cation interactions with yellow dotted lines, and π–π interactions with light blue dotted lines (PDB code 6O72).

**Figure 3:**
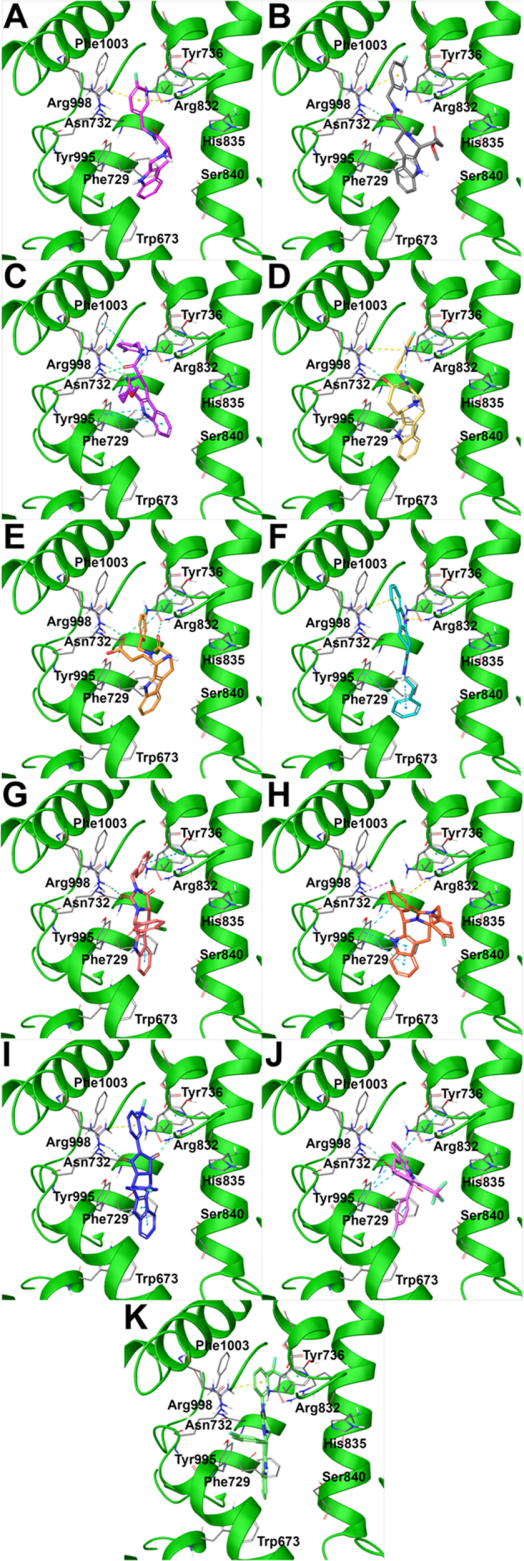
Predicted binding modes of (A) compound **9** (colored by atom type, C light violet), (B) **11a** (colored by atom type, C gray), (C) **11b** (colored by atom type, C purple), (D) **12a** (colored by atom type, C yellow), (E) **12b** (colored by atom type, C orange), (F) **23** (colored by atom type, C pale blue), (G) **31a** (colored by atom type, C pale red), (H) **31a′** (colored by atom type, C red-orange), (I) **36a** (colored by atom type, C violet), (J) **36a′** (colored by atom type, C light purple), (K) **36b** (colored by atom type, C light green) in docking with TRPM8 (represented in green ribbons; residues colored by atom type; C gray, N blue, O red, polar H light gray) in the TC-I 2014 ligand binding site. H bonds are represented with green dotted lines, π–cation interactions with yellow dotted lines, and π–π interactions with light blue dotted lines (PDB code 6O72).

The analysis of the ligand docking poses on this specific protein structure highlighted further details for clarifying the action of the investigated compounds at a molecular level (Figures 2 and 3). First, the tetrahydro-β-carboline-based compound **6a**, more conformationally restricted if compared with its parent compound **4**, showed a slightly different binding mode due to the flip of the indole moiety (Figure 2A). On the other hand, the careful analysis of the superimposed poses of **4** and **6a** highlighted a similar total shape (Figure 2B), and this was further confirmed by detecting a similar set of key interactions for both the compounds, such as the π–π stacking with Phe729 and the polar contacts with Gln776 and Asn790. Also, an additional π–π was detected with Tyr995, whereas the terminal benzyl moiety established a partial π–π contact with Tyr736 (Figure 2A).

Compound **9** occupied the TRPM8 binding site showing π–cation interactions with Arg832 and Arg998 and further π–π interactions with Tyr736 (as in the starting compound **4**; *vide supra*) and Phe1003 through the 4-fluorobenzyl function, whereas π–π stacking contacts were detected with Phe729 and Tyr995 through the indole moiety (Figure 3A). The introduction of a substituent at C-1, as in compounds **11a**, **11b**, **12a**, **12b**, determined a similar accommodation in the TRPM8 binding site (Figure 3B–E). Specifically, in the cases of compounds **11a** and **12a** the tetrahydro-β-carboline moiety was oriented in front of Phe729 and Tyr995 residues, while the 4-fluorobenzyl substituent interacted again with Arg998 through a π–cation and with Tyr736 through a π–π stacking (Figure 3B and Figure 3D). Also, the acid moiety in **12a** allowed a further H-bond interaction with Arg998 (Figure 3D). On the other hand, the different stereochemical arrangements of the related analogs **11b** and **12b** (featuring 1*S*,3*S* configuration, instead of 1*R*,3*S* as for compounds **11a** and **12a**) determined a slightly different binding mode. Specifically, for compound **11b**, the 4-fluorobenzyl substituent was inserted in a deep cavity in front of Phe1003, while the π–π stacking interactions with Phe729 and Tyr995 were again detected as well as further H-bonds with Asn732 and Arg998 (Figure 3C). A quite similar binding mode was observed for compound **12b**, in which the terminal carboxylate function was involved in H-bond interactions with Asn732 and Arg998, whereas the 4-fluorobenzyl substituent showed in this case a π–π interaction with Tyr736 (Figure 3E)

The introduction of a conformational restriction in compound **23**, featuring four fused rings (tetrahydropyrazino[1′,2′:1,6]pyrido[3,4-*b*]indole-1,4(6*H*,7*H*)-dione scaffold), determined a different placement in the binding site, namely, with the indole moiety establishing a π–cation interaction with Arg998 and Arg832, whereas the terminal benzyl moiety made further π–π contacts with Phe729 and Tyr995 (Figure 3F). Concerning compound **31a**, again featuring four fused rings (tetrahydro-1*H*-imidazo[1′,5′:1,6]pyrido[3,4-*b*]indole-1,3(2*H*)-dione scaffold), the presence of a substituent at C-5 determined a flip of the indole moiety, able to interact with Phe729 and Tyr995 through π–π stacking contacts, as previously observed for **11a**, **11b**, **12a**, **12b** that, interestingly, also featured an additional substituent at C-1, corresponding to C-5 in **31a**/**31a′**. Also, the 4-Cl-phenyl substituent at C-5 determined further π–π interaction with Tyr736, whereas an H-bond contact was established with Arg998 (Figure 3G). As expected, a similar binding mode was detected for compound **36a**, featuring the same absolute configurational pattern of **31a**, but the presence of a phenyl substituent at N-2 instead of a benzyl determined a slightly different accommodation of the tetrahydro-1*H*-imidazo[1′,5′:1,6]pyrido[3,4-*b*]indole-1,3(2*H*)-dione core and the consequent lack of the π–π stacking between the aromatic substituent at C-5 and Tyr736 (as observed for **31a**), replaced by an additional π–cation with Arg998 (Figure 3I). On the other hand, the corresponding enantiomeric species of **31a** and **36a**, namely, compounds **31a′** and **36a′**, respectively, showed a different occupation of the TRPM8 binding site due to the different stereoarrangements, especially for what concerns the position of the terminal substituted benzyl and aryl moieties, not in line with all the above-reported structure–activity observations, suggesting the poor consistency of this mode of binding that could explain the detected related decreases of antagonistic activity against TRPM8 (Figure 3H and Figure 3J). Interestingly, compound **36b**, the only one of the series featuring the 5*S*,11a*S* absolute configuration, showed a three-dimensional arrangement onto the TRPM8 compatible with the establishment of the key interactions with the receptor counterpart, namely, the π–π stacking with Phe736 through the terminal 3-(CF_3_)-aryl moiety (also able to interact with Arg998 through a π–cation) as well as the π–π interaction with Phe729 and Tyr995 with the indole moiety (Figure 3K). In summary, the comparison of the predicted binding modes related to the reference compound **4** and of the new identified TRPM8 inhibitors disclosed a similar accommodation in the ligand binding site, with the subsequent respect of a network of specific interactions with key residues in the receptor counterpart (e.g., Phe729, Tyr736, Tyr995, Arg998). These *in silico* results shed light on the rationalization of the observed antagonistic activity of the new identified compounds, providing structural insights for the development of new agents able to interfere with the activity of this target. Starting from these encouraging data at a molecular level, we then moved to the investigation of specific molecular properties of the identified compounds (e.g., in vitro metabolism; *vide infra*) for selecting the most promising items and for further deepening their antagonistic pharmacological profile against TRPM8.

### In Vitro Metabolism

**Figure 4:**
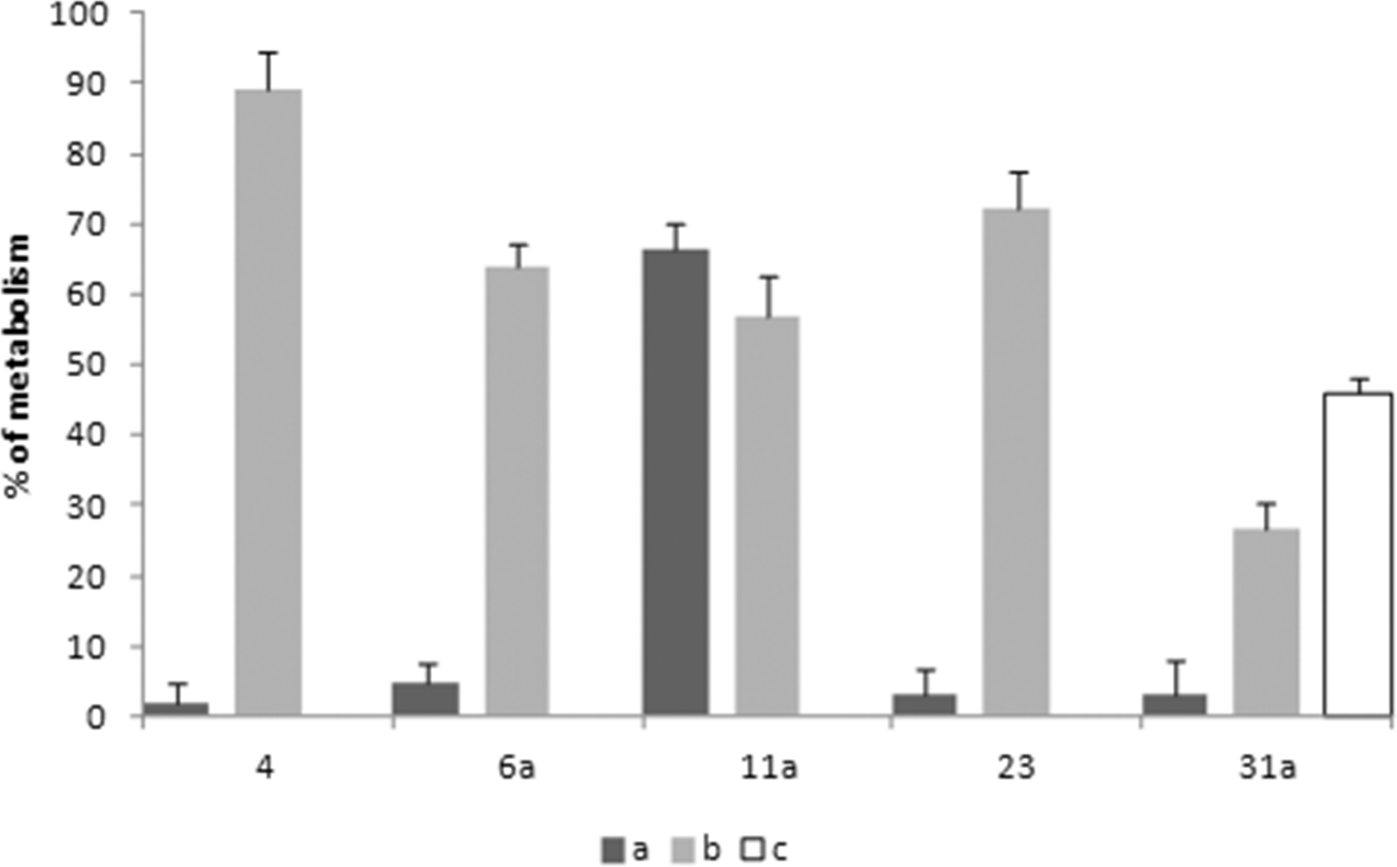
In vitro metabolic stability assays for the selected compounds: (a) compounds’ unspecific metabolism in absence of cofactors calculated as ([ ]t_60_/[ ]t_0_) × 100, black bars; (b) compounds’ metabolic stability under phase I metabolism (gray bars, protocol I; see materials and methods section); (c) compound **31a** metabolic stability under phase I + phase II metabolism (white bar, protocol II; see materials and methods section).

The most potent compounds analyzed by patch-clamp electrophysiological assays were further characterized for their metabolic stability using human liver microsomes as in vitro model. Compound **4** was used as reference, considering that its main pitfall was represented by metabolic instability that the newly synthesized compounds were aimed in overcoming. As shown in Figure 4, almost all the compounds proved to be stable in the absence of metabolic cofactors (NADPH or UDP-GlcUA/NADPH) except for **11a**, showing unspecific metabolic liability (black bars). In fact, after 60 min in contact with liver microsomes, in the absence of any metabolic cofactors, **11a** turnover was 66.5 ± 3.8%. When the phase I metabolism conditions were mimicked (see protocol I, material and methods section), compound **4** was massively metabolized with a turnover percentage of 98.3 ± 3.1% (Figure 4, gray bars), in accordance with our previously reported data.^18b^ Indeed, the newly synthesized analogues showed improved metabolic stability with a metabolic turnover in the range 1.1–72.0% under phase I metabolism conditions. In particular, compound **31a** with a phase I metabolic turnover of 26.5 ± 3.9% was the most stable compound. For these reasons, stability of derivative **31a** was further challenged using a different protocol that involved both phase I and phase II metabolic cofactors. As shown in Figure 4 (white bar), **31a** proved to have a slow metabolic turnover (46.0 ± 2.3%)^32^ in the experimental conditions used and was then selected for the in vivo pharmacological assays.

### In Vivo Experiments

#### Effect of **4** and **31a** on Icilin-Induced WDS

**Figure 5:**
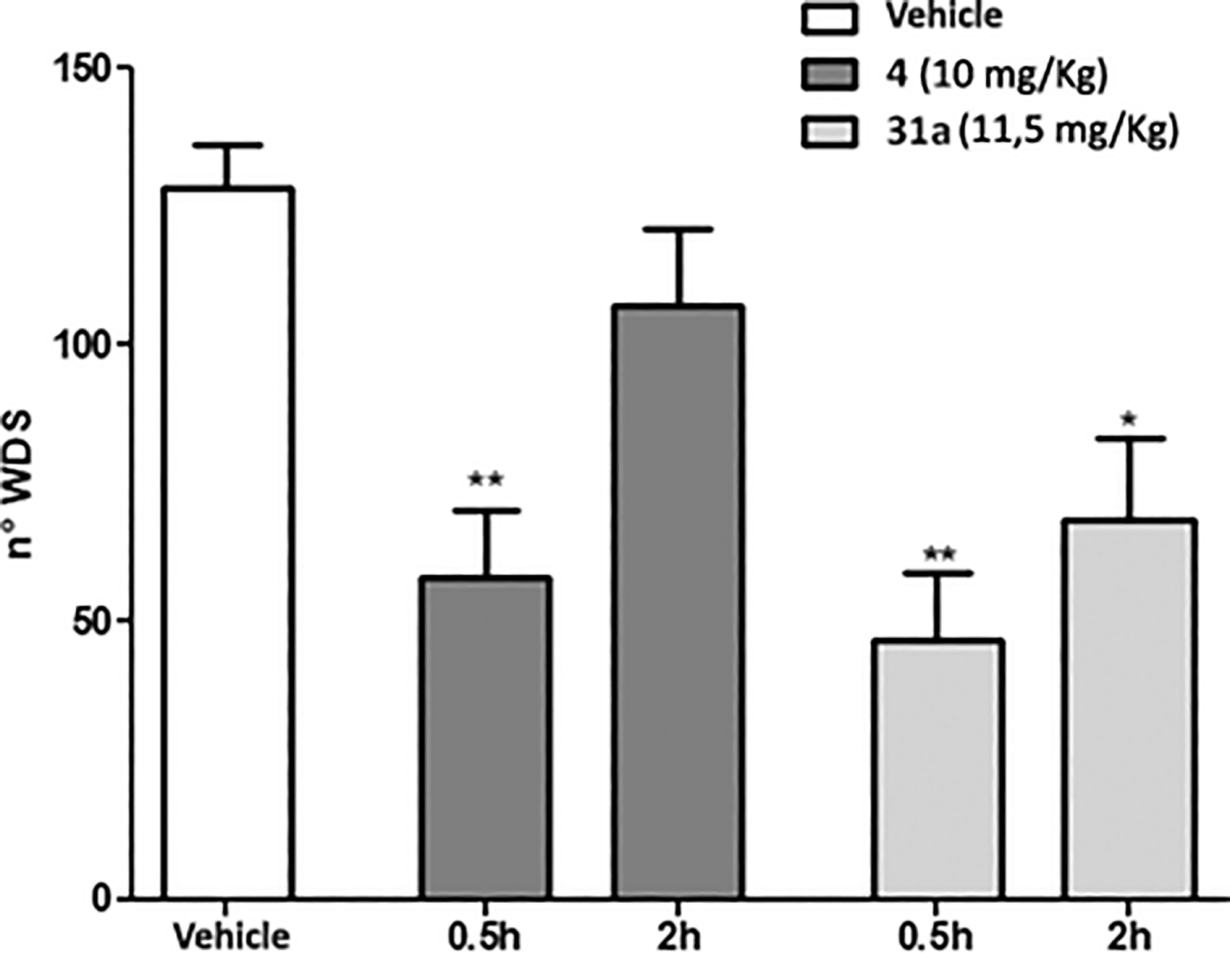
Comparative effect of **4** and **31a** on icilin-induced WDS in Swiss CD1 mice. After ip injection of icilin (10 mg/kg), the number of wet-dog shakes (WDS) were counted over 30 min. Data are given as the mean ± SEM *n* = 6, two-way ANOVA with Bonferroni post hoc test: *p < 0.05; **p < 0.01.

Initially, we have evaluated the capability of TRPM8 antagonist **31a** in blocking the spontaneous wet-dog shake (WDS) induced by icilin in comparison with its precursor derivative **4** at equimolar doses. Due to the difference in metabolic stability, a prolonged pharmacological effect of **31a** was expected. For this purpose, **4** and **31** were administrated 30 min before the challenge with icilin (1 mg/kg ip) and WDS was recorded for 30 min. In the vehicle-treated group, a mean of about 128 shakes were counted (Figure 5, white column). As expected, from the metabolic stability experiments, the pretreatment with **4** (10 mg/kg ip) significantly decreased the number of icilin-induced WDS 0.5 h after the injection (Figure 5; **p < 0.01 vs vehicle treated mice); no effect was observed at 2 h. On the contrary, **31a** (11.5 mg/kg ip) showed a significant effect at both 0.5 and 2 h after the injection (Figure 5; *p < 0.05 and **p < 0.01 vs vehicle treated mice).

#### Effect of **31a** in Neuropathic Pain Models

TRPM8 plays a critical role in mouse models of chemotherapy-induced neuropathic pain evoked by oxaliplatin (OXP), a condition mimicking cold hypersensitivity provoked by chemotherapy-induced peripheral neuropathy (CIPN). Both acute and chronic OXP-induced cold hypersensitivity has been reproduced in rats and correlated with TRPM8 expression and function. Mizoguchi et al.^33^ reported that in a rodent model, acute cold allodynia after OXP injection was alleviated by the TRPM8 blockers *N*-(2-aminoethyl)-*N*-[4-(benzyloxy)-3- methoxybenzyl]-*N*′-(1*S*)-1-(phenyl)ethyl]urea and TC-I 2014. According to these findings, we investigated the effect of our antagonist **31a** in an OXP-induced cold allodynia model, using acetone for cooling stimulation. Considering that the cold pain threshold is increased from ≈12 °C to ≈26 °C in OXP-treated patients, acetone stimulation is considered to evoke pain in OXP-treated mice.

**Figure 6:**
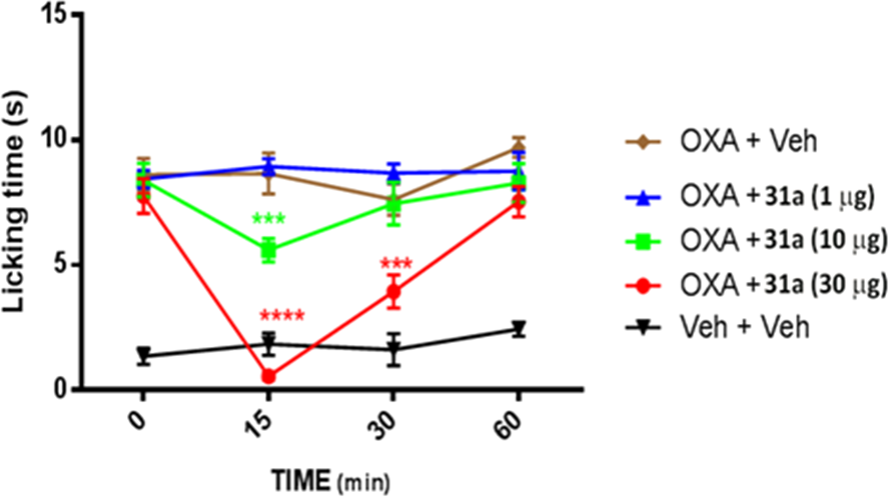
Dose-dependent inhibition of nocifensive paw licking given by compound **31a** (1, 10, and 30 μg, sc) in oxaliplatin-induced cold allodynia in C57/BL6 mice. Data are given as the mean ± SEM *n* = 6. Statistical analysis was two-way ANOVA followed by post hoc Bonferroni test by multiple comparison: ***p < 0.001, ****p < 0.0001.

The activity of compound **31a** was evaluated 7 days after three intraperitoneal injections of OXP (6 mg/kg) in C57/BL6 mice, when cold allodynia had developed. As shown in Figure 6, a single subcutaneous administration of 1 μg of **31a** was not effective in inhibiting the (OXP)-induced cold allodynia, whereas injections of 10 and 30 μg of our compound showed a remarkable inhibitory effect, which was maximum after 15 min. This effect was still evident 30 min after administration of a 30 μg dose (Figure 6). These data suggest that **31a** may be a viable therapeutic scaffold for the treatment of CIPN.

Further we investigated the efficacy of **31a** in a chronic constriction injury (CCI) model of neuropathic pain, using a thermal gradient ring assay. This assay deeply differs from the canonical reflexive measures of nociception, in which the end point is withdrawal to a noxious stimulus, a fact that has been questioned during the past years for their unsatisfactory translation.^34^ In particular, this test integrates information on temperature perception distinguishing exploratory behavior from thermal preference behavior.^35a^ Thus, we measured the thermal preference location of sham, CCI-mice, and CCI-mice treated intraperitoneally with **31a** in a thermal gradient assay equilibrated between 15 and 40 °C.

**Figure 7:**
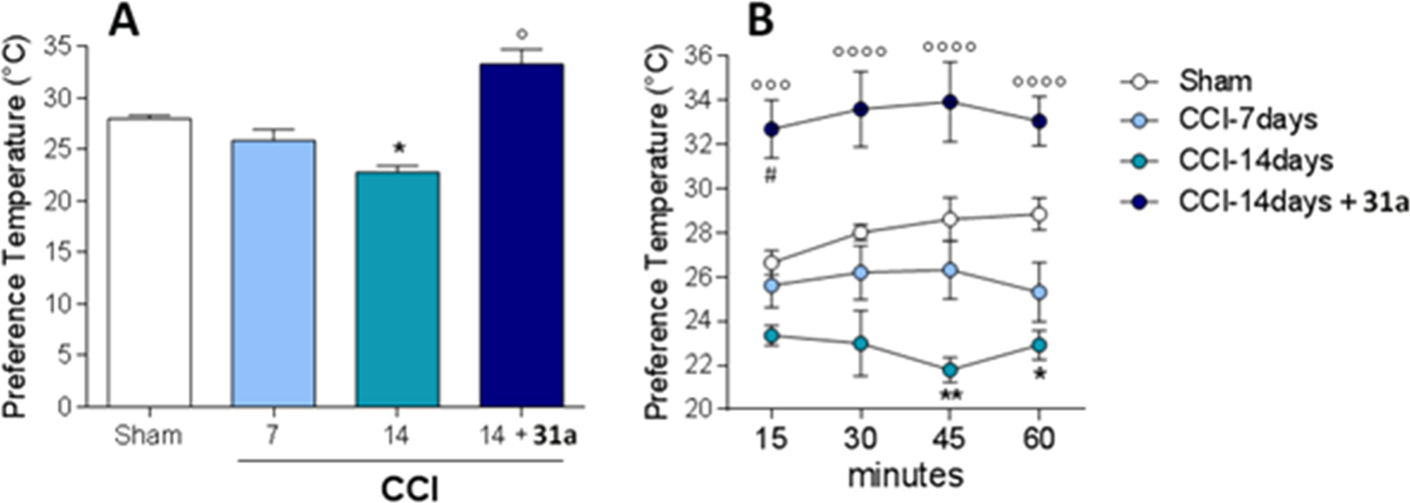
(A) Thermal preference behavior of Swiss CD1 mice 7 days (light blue bar) and 14 days (green bar) after CCI. The blue bar represents the effect of compound **31a** administration at 14 days of CCI. Data are given as the mean ± SEM, *n* = 6, two-way ANOVA with Bonferroni post hoc test: *p < 0,05 vs sham mice; °*p* < 0,05 vs 14 days CCI. (B) Time course thermal preference behavior of sham Swiss CD1 mice (white dots), CD1 mice 7 days (light blue dots), and 14 days (green dots) after CCI. The blue dots represent the time course effect of compound **31a** administration at 14 days of CCI. Data are given as the mean ± SEM *n* = 6, two-way ANOVA with Bonferroni post hoc test: *p < 0,05 and **p < 0.01 vs vehicle treated; °°°*p* < 0.001 and °°°°*p* < 0.0001 vs CCI-mice; ^#^*p* < 0.05 vs sham mice.

The mean temperature to which the sham animal located during the observation time was 27.9 °C ± 0.35 °C (Figure 7A), and no statistical differences were evidenced at the different time points (Figure 7B). No effects on temperature preferences were observed after **31a** administration in sham-mice (data not shown). This value slightly differs from the previously reported by Touska et al.^35a^ but is consistent with gender, age, and strain differences within animals used. The same temperature preference was observed in CCI-mice 7 days after ligation (mean preferred temperature 25.88 °C ± 1.08 °C, for CCI mice, *p* = 1.452 vs sham mice, Figure 7A and Figure 7B). However, 14 days after ligation, when the neuropathic pain and the related nociceptive disorders are well-known to occur,^35b,35c^ the CCI animals displayed a marked preference for colder areas (mean temperature = 22.80 °C ± 0.61 °C, *p < 0.05 vs sham mice, Figure 7A), which was most prominent during the first 45 min of exposure as shown in Figure 7B (**p < 0.01 vs sham mice) and extending to 60 min. This is in accordance with the cold-seeking behaviors reported during inflammatory states.^35d^ Considering that thermosensation is mediated by the primary afferent Aδ and C fibers,^35e^ where TRPM8 is particularly represented,^2^ its role in the cold-seeking behaviors of CCI animal seems evident. In fact, intraperitoneal administration of the TRPM8 antagonist **31a** (11.5 mg/kg) significantly reverted this behavior to 33.30 °C ± 1.44 °C (Figure 7A; °*p* < 0.05 vs CCI 14 days). Similar enhanced thermal tolerance has been recently reported when the antihyperalgesic drug clonidine was administered in a CCI mouse model.^34^ Moreover, the mice behavior is also in accordance with previous data that describe TRPM8 deficient mice (TRPM8^–/–^) as rather preferring warmer than colder areas.^35a^ It should be noted that mice treated with **31a** immediately recognized warmer zones as preferable to colder areas compared to vehicle CCI-mice (Figure 7B; °°°*p* < 0.001 and °°°°*p* < 0.0001 vs CCI-mice) also showing a preference for an even warmer temperature than sham animals during the first 15 min (Figure 7B; ^#^*p* < 0.05 vs sham mice). It is questionable why this transient effect was recorded, but it must be considered that TRPM8 antagonists are able to decrease the body temperature. This effect could probably account for the thermal preference expressed by animals treated with **31a** at 15 min.

The efficacy and the rapid onset of action further confirm the efficacy of compound **31a** as TRPM8 antagonists.

## Conclusions

Following our interest in the TRPM8 modulation and taking into account the in vivo promising results obtained with a tryptophan-based TRPM8 antagonist (**4**), in this work we describe the synthesis and the pharmacological characterization of different conformationally restricted analogues of this hit compound, designed with the dual objective of exploring the structural requirements for antagonizing TRPM8 at molecular level and improving the metabolic stability of our hit compound. Some of the synthesized compounds featuring tetrahydrocarboline, tetrahydropyrazino[1′,2′:1,6]pyrido[3,4-*b*]indole-1,4(6*H*,7*H*)-dione, and tetrahydro-1*H*-imidazo[1′,5′:1,6]pyrido[3,4-*b*]indole-1,3(2*H*)-dione chemical structures showed an efficient and potent TRPM8 antagonist activity in the nanomolar range. Using a new TRPM8 three-dimensional protein structure, we rationalized the SAR of this series of compounds by identifying the structural and stereochemical requirements that determine their competitive antagonist activity. One of the synthesized compounds, the (5*R*,11a*S*)-5-(4-chlorophenyl)-2-(4-fluorobenzyl)-5,6,11,11a-tetrahydro-1*H*-imidazo[1′,5′:1,6]pyrido[3,4-*b*]indole-1,3(2*H*)-dione, **31a**, has a slow metabolic turnover and both overcomes TRPM8-mediated cold hypersensitivity over time, as measured in the WDS assay, and displays acute antinociceptive response 15 min after its application in an oxaliplatin-induced cold allodynia model. In addition, **31a** also shows remarkable analgesic activity in an animal model of CCI-induced hyperalgesia. These last data are in agreement with the results obtained with **4** in other models of neuropathic pain^27d^ but differ with those obtained by other authors who demonstrate the efficacy of the TRPM8 agonists in animal models of injury-induced neuropathic pain.^27a−27c^ Our results confirm the validity of the indole nucleus in the design of potent TRPM8 modulators, adding one more piece to the puzzle that composes the TRPM8’s complex biology in the transmission and modulation of pain.

## Experimental Section

### General

All reagents and solvents used were purchased from Sigma-Aldrich (Milan, Italy) unless otherwise stated. Reactions were performed under magnetic stirring in round-bottomed flasks unless otherwise noted. Moisture-sensitive reactions were conducted in oven-dried glassware under nitrogen stream, using freshly distilled solvents. TLC analysis of reaction mixtures was performed on precoated glass silica gel plates (F254, 0.25 mm, VWR International), while crude products were purified by the Isolera Spektra One automated flash chromatography system (Biotage, Uppsala, Sweden), using commercial silica gel cartridges (SNAP KP-Sil, Biotage). NMR spectra were recorded on a Bruker Avance 400 MHz apparatus, at room temperature. Chemical shifts were reported in δ values (ppm) relative to internal Me_4_Si for ^1^H and ^13^C NMR and to CFCl_3_ for ^19^F NMR. *J* values were reported in hertz (Hz). ^1^H NMR and ^19^F NMR peaks were described using the following abbreviations: s (singlet), d (doublet), t (triplet), and m (multiplet). HR-MS spectra were recorded by LTQ-Orbitrap-XL-ETD mass spectrometer (Thermo Scientific, Bremen, Germany), equipped with an ESI source. Analytical RP-HPLC analysis of final products was performed through a Nexera UHPLC system (Shimadzu, Kyoto, Japan) consisting of a CBM-20A controller, two LC-30AD pumps, a DGU-20 A5R degasser, an SPD-M20A photodiode array detector, a CTO-20AC column oven, a SIL-30AC autosampler, and a Kinetex C18 150 mm × 2.1 mm × 2.6 μm (100 Å) column (Phenomenex, Bologna, Italy). The optimal mobile phase consisted of 0.1% HCOOH/H_2_O v/v (A) and 0.1% HCOOH/ACN v/v (B). Analysis was performed in gradient elution as follows: 0–13.00 min, 5–65% B; 13–14.00 min, 65–95% B; 14–15.00 min, isocratic to 95% B; then 3 min for column re-equilibration. Flow rate was 0.5 mL min^–1^. Column oven temperature was set to 40 °C. Injection volume was 5 μL of sample. The following PDA parameters were applied: sampling rate, 12.5 Hz; detector time constant, 0.160 s; cell temperature, 40 °C. Data acquisition was set in the range 190–800 nm, and chromatograms were monitored at 230 nm. Analytical RP-HPLC confirmed that all final compounds had a purity of >95%. For quantitative analysis, the calibration curve was obtained in a concentration range of 2.5–40 μM with five concentration levels and triplicate injections of each level were run. Peak areas were plotted against corresponding concentrations, and the linear regression was used to generate a calibration curve with *R*^2^ values of ≥0.999 (Table S1).

Stability analysis for **36b** was performed using the same chromatographic conditions reported above but with the following elution gradient: 0–13.00 min, 15–65% B; 13–14.00 min, 65–95% B; 14–15.00 min, isocratic to 95% B; then 3 min for column re-equilibration.

All circular dichroism spectra were recorded using a JASCO J810 spectropolarimeter at 25°C in the range λ = 260–190 nm (1 mm path length, 1 nm bandwidth, four accumulations, and a scanning speed of 10 nm min^–1^). Compounds were dissolved in methanol at a concentration of 0.100 mM. Spectra were corrected for the solvent contribution.

### General Procedure A: Pictet–Spengler Reaction

1 mmol of l-tryptophan methyl ester or (*S*)-2-amino-*N*-(4-fluorobenzyl)-3-(1*H*-indol-3-yl)propanamide (**8**) was dissolved in methanol and added with the proper aldehyde (1.5 equiv) and trifluoroacetic acid (1.5 equiv). The mixture was subjected to a microwave assisted closed vessel reaction for 45 min at 110 °C.^36^ The mixture was then evaporated *in vacuo*, and the residue was dissolved in dichloromethane and was washed three times with water. The organic phase was extracted, dried over Na_2_SO_4_, filtered, and concentrated under vacuum. The crude products were purified by flash chromatography using mixtures of *n*-hexane/ethyl acetate as mobile phase.

### General Procedure B: Coupling Reactions

1 mmol of the proper carboxylic acid was dissolved in dichloromethane/DMF (4:1 v:v) and added with HoBt (1.2 equiv), HBTU (1.2 equiv), DIPEA (2.4 equiv), and the corresponding amine (1.2 equiv) and stirred at room temperature overnight. Then, the solvent was evaporated in vacuum, and the residue was dissolved in dichloromethane and washed with water (3 times), a saturated solution of NaHCO_3_ (3 times), and a solution of citric acid (10% w:w). The organic phase was extracted, dried over Na_2_SO_4_, filtered, and concentrated under vacuum. The crude products were purified by flash chromatography using mixtures of *n*-hexane/ethyl acetate as mobile phase.

### General Procedure C: Boc Removal

The *N*-Boc protected intermediate (0.2 mmol) was dissolved in a mixture of TFA/DCM (1/3, v/v), and triisopropylsilane (TIS, 0.25 equiv) was added. Reaction was stirred at room temperature for 2 h. Then, a solution of NaOH (2 N) was added dropwise until pH 7. The mixture was diluted with water and dichloromethane, and the organic phase was extracted, dried over Na_2_SO_4_, filtered, and concentrated under vacuum. The crude products were purified by flash chromatography using mixtures of *n*-hexane/ethyl acetate as mobile phase.

### General Procedure D: Hydantoin Synthesis

Diastereoisomerically pure tetrahydro-β-carbolines (0.2 mmol) were dissolved in THF, and 0.4 equiv of triphosgene was added. The pH was adjusted to 8 by addition of TEA, and the mixture was stirred at room temperature for 10 min. Then, the proper amine (1.2 equiv) was added and the resulting mixture was refluxed for 1 h. After cooling to room temperature, the solvent was evaporated, the residue reconstituted in dichloromethane and washed with water (3 times). The organic phase was extracted, dried over Na_2_SO_4_, filtered, and concentrated under vacuum. The crude products were purified by flash chromatography using mixtures of *n*-hexane/ethyl acetate as mobile phase.

### General Procedure E: Hydantoin Synthesis

Tetrahydro-β-carboline **32a** or **32b** (0.2 mmol) was dissolved in THF, and 1.2 equiv of trimethylamine and 1.2 equiv of the proper isocyanate were added. The mixture was stirred at room temperature for 30 min. The solvent was evaporated, the residue reconstituted in dichloromethane and washed with water (3 times). The organic phase was extracted, dried over Na_2_SO_4_, filtered, and concentrated under vacuum. The crude products were purified by flash chromatography using mixtures of *n*-hexane/ethyl acetate as mobile phase.

### General Procedure F: Hydantoin Synthesis

Tetrahydro-β-carboline **32b** (0.2 mmol) was dissolved in THF, and 1.2 equiv of trimethylamine and 1.2 equiv of the proper isocyanate were added. The mixture was stirred at room temperature for 30 min and then refluxed for further 30 min. After cooling to room temperature, the solvent was evaporated, the residue reconstituted in dichloromethane and washed with water (3 times). The organic phase was extracted, dried over Na_2_SO_4_, filtered, and concentrated under vacuum. The crude products were purified by flash chromatography using mixtures of *n*-hexane/ethyl acetate as mobile phase.

### (1*R*,3*S*)-Methyl 1-Phenyl-2,3,4,9-tetrahydro-1*H*-pyrido[3,4-*b*]indole-3-carboxylate (**5a**)

Compound **7a** was obtained using general procedure A in 33% yield, using benzaldehyde as starting material. Spectral data were in accordance with literature.^37^ FC in *n*-hexane/ethyl acetate 1/1, *R_f_* = 0.37.

### (1*S*,3*S*)-Methyl 1-Phenyl-2,3,4,9-tetrahydro-1*H*-pyrido[3,4-*b*]indole-3-carboxylate (**5b**)

Compound **7b** was obtained using general procedure A in 41% yield, using benzaldehyde as starting material. Spectral data were in accordance with literature.^37^ FC in *n*-hexane/ethyl acetate 1/1, *R_f_* = 0.44.

### (1*R*,3*S*)-Methyl 2-Benzyl-1-phenyl-2,3,4,9-tetrahydro-1*H*-pyrido[3,4-*b*]indole-3-carboxylate (**6a**)

Intermediate **5a** (0.2 mmol) was dissolved in THF and added with NaI (1.1 equiv), Pd(CH_3_COO)_2_ (10% mol), TEA (1.2 equiv), and benzyl bromide (1.2 equiv). The mixture was subjected to a microwave assisted closed vessel reaction for 45 min at 110 °C. After removal of the solvent, the residue was reconstituted in dichloromethane and was washed three times with water. The organic phase was extracted, dried over Na_2_SO_4_, filtered, and concentrated under vacuum. The crude products were purified by flash chromatography using *n*-hexane/ethyl acetate 1/1, *R_f_* = 0.35. White powder (62% yield). [α]^25^_D_: −101.16 ± 0.17 (*c* = 0.10, MeOH). ^1^H NMR (400 MHz, CDCl_3_) δ: 3.14 (d, 2H, C*H*_2_, *J* = 4.8 Hz); 3.55 (s, 3H, C*H*_3_); 3.80 (d, 2H, C*H*_2_, *J* = 11.8 Hz) 3.85–3.88 (m, 1H, C*H*); 5.39 (s, 1H, C*H*); 6.99–7.02 (m, 2H, aryl); 7.04 (d, 1H, aryl, *J* = 7.2 Hz); 7.10–7.28 (m, 8H, aryl); 7.39 (d, 2H, aryl, *J* = 7.8 Hz); 7.43 (d, 1H, aryl, *J* = 8.0 Hz). ^13^C NMR (100 MHz, CDCl_3_) δ 24.5, 51.4, 54.4, 56.1, 60.9, 106.4, 110.8, 118.2, 119.3, 121.6, 127.0, 127.1, 128.1, 128.4, 128.6, 128.8, 128.9, 135.0, 136.5, 139.4, 142.2, 173.6. HR-MS *m*/*z* calcd for C_26_H_24_N_2_O_2_ [(M + H)]^+^: 397.1911; found 397.1918.

### (1*S*,3*S*)-Methyl 2-Benzyl-1-phenyl-2,3,4,9-tetrahydro-1*H*-pyrido[3,4-*b*]indole-3-carboxylate (**6b**)

Final product **6b** was synthesized starting from **5b** and following the procedure described above for **6a**. FC in *n*-hexane/ethyl acetate 2/1, *R_f_* = 0.40. White powder (59% yield). [α]^25^_D_: 135.18 ± 0.25 (*c* = 0.10, MeOH). ^1^H NMR (400 MHz, CDCl_3_) δ: 3.08 (dd, 1H, C*H*_2a_, *J*′ = 4.4, *J*″ = 15.6 Hz); 3.31 (s, 3H, C*H*_3_); 3.40 (dd, 1H, C*H*_2b_, *J*′ = 7.8, *J*″ = 15.6 Hz); 3.87 (t, 1H, *J* = 8.0 Hz, C*H*); 3.92 (d, 1H, C*H*_2a_, *J* = 16.0 Hz); 4.08 (d, 1H, C*H*_2b_, *J* = 16.0 Hz); 4.96 (s, 1H, C*H*); 7.13–7.23 (m, 2H, aryl); 7.25–7.36 (m, 11H, aryl); 7.56 (d, 1H, aryl, *J* = 7.6 Hz). ^13^C NMR (100 MHz, CDCl_3_) δ 22.7, 51.4, 57.2, 61.0, 61.8, 107.2, 110.8, 118.4, 119.5, 121.8, 126.8, 127.1, 128.0, 128.5, 129.3, 133.2, 136.4, 138.2, 140.2, 173.5. HR-MS *m*/*z* calcd for C_26_H_24_N_2_O_2_ [(M + H)]^+^: 397.1911; found 397.1920.

### *tert*-Butyl (*S*)-(1-((4-Fluorobenzyl)amino)-3-(1*H*-indol-3-yl)-1-oxopropan-2-yl)carbamate (**7**)

Synthesized according to the general procedure B, starting from *N*-Boc-l-tryptophan-OH and 4-fluorobenzylamine. FC in *n*-hexane/ethyl acetate 3/2, *R_f_* = 0.6. yellowish oil (75% yield). ^1^H NMR (400 MHz, CDCl_3_) δ 1.42 (s, 9H, C*H*_3_); 3.17–3.22 (m, 1H, C*H*_2a_); 3.30–3.35 (m, 1H, C*H*_2b_) 4.14–4.25 (m, 2H, C*H*_2_); 4.49 (bs, 1H, C*H*); 5.27 (bs, 1N*H*); 6.20 (s, 1H, C*H*); 6.89–6.95 (m, 4H, aryl); 7.13 (t, 1H, aryl, *J* = 7.2 Hz); 7.21 (t, 1H, aryl, *J* = 7.6 Hz); 7.37 (d, 1H, aryl, *J* = 8.0 Hz); 7.66 (d, 1H, aryl, *J* = 7.6 Hz); 8.45 (bs, 1N*H*). HR-MS *m*/*z* calcd for C_23_H_26_FN_3_O_3_ [(M + H)]^+^: 411.1958; found 411.1963.

### (*S*)-2-Amino-*N*-(4-fluorobenzyl)-3-(1*H*-indol-3-yl)propanamide (**8**)

Intermediate **8** was synthesized according to the general procedure C, starting from **7**. FC in ethyl acetate, *R_f_* = 0.3. White powder (94% yield). ^1^H NMR (400 MHz, CD_3_OD) δ 3.06 (dd, 1H, C*H*_2a_, *J*′ = 6.5, *J*″ = 14.1 Hz); 3.18 (dd, 1H, C*H*_2b_, *J*′ = 7.0, *J*″ = 14.1 Hz); 3.69 (t, 1H, C*H*, *J* = 6.8 Hz); 4.18 (d, 1H, C*H*_2a_, *J* = 14.9 Hz); 4.31 (d, 1H, C*H*_2b_, *J* = 14.9 Hz); 6.89–6.98 (m, 3H, aryl); 7.01–7.06 (m, 2H, aryl); 7.13 (t, 1H, aryl, *J* = 7.8 Hz); 7.39 (d, 1H, ar yl, *J* = 8.1 Hz); 7.63 (d, 1H, aryl, *J* = 7.9 Hz). HR-MS *m*/*z* calcd for C_18_H_18_FN_3_O [(M + H)]^+^: 312,1507; found 311.1512.

### (*S*)-*N*-(4-Fluorobenzyl)-2,3,4,9-tetrahydro-1*H*-pyrido[3,4-*b*]indole-3-carboxamide (**9**)

Compound **9** was obtained using general procedure A, starting from intermediate **8** which was reacted with formaldehyde. Compound FC in ethyl acetate/acetone 9.8/0.2, *R_f_* = 0.48. White powder (55% yield). ^1^H NMR (400 MHz, DMSO): δ: 2.69 (dd, 1H, C*H*2a, *J*′ = 9.7, *J*″ = 14.9 Hz); 2.90 (dd, 1H, C*H*2b, *J*′ = 4.5, *J*″ = 15.2 Hz); 3.48–3.53 (m, 1H, C*H*); 3.95 (dd, 2H, C*H2, J*′ = 17.4, *J*″ = 22.5 Hz); 4.32 (d, 2H, C*H*2, *J* = 5.6, Hz); 6.94 (t, 1H, aryl, *J* = 7.1 Hz); 7.01 (t, 1H, aryl, *J* = 7.3 Hz); 7.17 (t, 2H, aryl, *J* = 8.8 Hz); 7.26–7.39 (m, 3H, aryl); 8.46 (t, 1H, aryl, *J* = 6.1 Hz); 10.68 (s, 1H, N*H*). ^13^C NMR (100 MHz, DMSO) δ 25.4, 41.7, 42.3, 57.0, 106.9, 111.3, 115.3, 115.5, 117.7, 118.7, 120.9, 127.6, 129.6, 129.7, 134.3, 136.2, 136.32, 136.34, 160.4, 162.8, 173.2. ^19^F NMR (DMSO, 376.3 MHz) δ: −(116.37) (s, 1F, C*F*). HR-MS *m*/*z* calcd for C_19_H_18_FN_3_O [(M + H)]^+^: 324.1507; found 324.1516.

### (1*R*,3*S*)-*N*-(4-Fluorobenzyl)-1-isobutyl-2,3,4,9-tetrahydro-1*H*-pyrido[3,4-*b*]indole-3-carboxamide (**10a**)

Compound **10a** was obtained using general procedure A, starting from intermediate **8** which was reacted with isovaleraldehyde. FC in hexane/ethyl acetate 1/1, *R_f_* = 0.46. White powder (35% yield). ^1^H NMR (400 MHz, CDCl_3_): δ: 0.90 (d, 3H, C*H*_3_, *J* = 5.5 Hz); 0.93 (d, 3H, C*H*_3_, *J* = 5.7 Hz); 1.40–1.48 (m, 1H, C*H*_*2a*_); 1.56–1.68 (m, 1H, C*H*_*2b*_); 1.85–1.89 (m, 1H, C*H*); 2.78 (dd, 1H, C*H*_*2a*_, *J*′ = 8.3, *J*″= 17.2 Hz); 3.21 (dd, 1H, C*H*_*2b*_, *J*′ = 5.0, *J*″= 15.9 Hz); 3.71 (dd, 1H, C*H*, *J*′ = 5.0, *J*″ = 7.9 Hz); 4.05 (dd, 1H, C*H*, *J*′ = 4.2, *J*″= 10.0 Hz); 4.36 (dd, 1H, C*H*_2a_, *J*′ = 5.7, *J*″= 14.8 Hz); 4.43 (dd, 1H, C*H*_2b_, *J*′ = 5.7, *J*″= 14.8 Hz); 6.95 (t, 2H, aryl, *J* = 8.7 Hz); 7.01–7.11 (m, 2H, aryl); 7.18–7.24 (m, 2H, aryl); 7.39 (t, 1H, aryl, *J* = 5.8 Hz); 7.45 (d, 1H, aryl, *J* = 6.3 Hz); 7.62 (s, 1H, N*H*). ^13^C NMR (100 MHz, CDCl_3_) δ 22.0, 23.5, 24.6, 25.1, 42.5, 43.9, 49.5, 52.7, 108.4, 110.7, 115.4, 115.7, 118.3, 119.7, 121.9, 127.3, 129.7, 135.9, 136.4, 173.0. ^19^F NMR (CDCl_3_, 376.3 MHz) δ: −(115.17) (s, 1F, C*F*). HR-MS *m*/*z* calcd for C_23_H_26_FN_3_O [(M + H)]^+^: 380.2133; found 380.2139.

### (1*S*,3*S*)-*N*-(4-Fluorobenzyl)-1-isobutyl-2,3,4,9-tetrahydro-1*H*-pyrido[3,4-*b*]indole-3-carboxamide (**10b**)

Compound **10b** was obtained using general procedure A, starting from intermediate **8** which was reacted with isovaleraldehyde. FC in hexane/ethyl acetate 6/4, *R_f_* = 0.44. White powder (43% yield). ^1^H NMR (400 MHz, CDCl_3_): δ: 0.92 (d, 3H, C*H*_3_, *J* = 6.6 Hz); 0.96 (d, 3H, C*H*_3_, *J* = 6.5 Hz); 1.41–1.48 (m, 1H, C*H*_*2a*_); 1.61–1.68 (m, 1H, C*H*_*2b*_); 1.95–1.97 (m, 1H, C*H*); 2.66 (dd, 1H, C*H*_*2a*_, *J*′= 8.7, *J*″= 15.6 Hz); 3.29 (dd, 1H, C*H*_*2a*_, *J*′ = 4.4, *J*″= 17.6 Hz); 3.52 (dd, 1H, C*H*, *J*′= 4.5, *J*″= 11.3 Hz); 4.08 (d, 1H, C*H*, *J* = 8.7 Hz); 4.42 (d, 2H, C*H*_2_, *J* = 5.7, Hz); 6.96 (t, 2H, aryl, *J* = 8.6 Hz); 7.02–7.10 (m, 2H, aryl); 7.19–7.25 (m, 2H, aryl); 7.38 (t, 1H, aryl, *J* = 5.6 Hz); 7.43 (d, 1H, aryl, *J* = 7.5 Hz); 7.75 (s, 1H, N*H*). ^13^C NMR (100 MHz, CDCl_3_) δ 21.9, 23.8, 25.5, 30.9, 42.4, 44.2, 51.9, 57.9, 109.0, 110.8, 115.4, 115.7, 118.3, 119.7, 121.9, 127.4, 129.4, 134.4, 135.9, 136.5, 163.4, 172.9. ^19^F NMR (CDCl_3_, 376.3 MHz) δ: −(115.89) (s, 1F, C*F*). HR-MS *m*/*z* calcd for C_23_H_26_FN_3_O [(M + H)]^+^: 380.2133; found 380.2142.

### Methyl 3-((1*R*,3*S*)-3-((4-Fluorobenzyl)carbamoyl)-2,3,4,9-tetrahydro-1*H*-pyrido[3,4-*b*]indol-1-yl)propanoate (**11a**)

Compound **11a** was obtained using general procedure A, starting from intermediate **8** which was reacted with methyl-4-oxobutanoate. FC in dichloromethane/methanol 9.5/0.5, *R_f_* = 0.44. White powder (43% yield). ^1^H NMR (400 MHz, CDCl_3_) δ: 1.98–2.14 (m, 2H, C*H*_2_); 2.49–2.67 (m, 2H, C*H*_2_); 2.80 (dd, 1H, C*H*_*2a*_, *J*′= 15.6, *J*″= 19.8 Hz); 3.30 (dd, 1H, C*H*_*2b*_, *J*′ = 4.6, *J*″= 19.4 Hz); 3.67 (s, 3H, C*H*_3_); 3.73 (dd, 1H, C*H*, *J*′= 8.0, *J*″= 16.0 Hz); 4.06 (dd, 1H, C*H*, *J*′ = 4.4, *J*″= 12.2 Hz); 4.45 (dd, 1H, C*H*_2a_, *J*′ = 7.8, *J*″= 18.8 Hz); 4.54 (dd, 1H, C*H*_2b_, *J*′ = 7.8, *J*″= 18.8 Hz); 7.05 (t, 2H, aryl, *J* = 8.0 Hz); 7.09–7.18 (m, 2H, aryl); 7.21–7.39 (m, 3H, aryl); 7.53 (d, 1H, aryl, *J* = 8.0 Hz); 8.21 (s, 1H, N*H*). ^13^C NMR (100 MHz, CDCl_3_) δ: 24.7, 29.7, 31.4, 42.6, 51.3, 51.8, 52.6, 108.7, 110.8, 115.4, 115.7, 118.4, 119.6, 122.1, 127.1, 129.5, 134.3, 135.2, 136.0, 161.1, 163.4, 172.7, 174.4. HR-MS *m*/*z* calcd for C_23_H_24_FN_3_O_3_ [(M + H)]^+^: 410.1874; found 410.1888.

### Methyl 3-((1*S*,3*S*)-3-((4-Fluorobenzyl)carbamoyl)-2,3,4,9-tetrahydro-1*H*-pyrido[3,4-*b*]indol-1-yl)propanoate (**11b**)

Compound **11b** was obtained using general procedure A, starting from intermediate **8** which was reacted with methyl-4-oxobutanoate. FC in dichloromethane/methanol 9.5/0.5, *R_f_* = 0.41. White powder (37% yield). ^1^H NMR (400 MHz, CDCl_3_): δ: 1.83–1.88 (m, 1H, C*H*_*2a*_); 2.25–2.30 (m, 1H, C*H*_*2b*_); 2.44–2.49 (m, 2H, C*H*_2_); 2.64–2.72 (m, 1H, C*H*_*2a*_); 3.25 (dd, 1H, C*H*_*2b*_, *J*′ = 4.8, *J*″= 12.0 Hz); 3.54–3.58 (m, 4H, C*H*_3_ and C*H*); 4.12 (dd, 1H, C*H*, *J*′= 4.2, *J*″= 8.6 Hz); 4.36–4.45 (m, 2H, C*H*_2_); 6.97 (t, 2H, aryl, *J* = 8.0 Hz); 7.04 (t, 1H, aryl, *J* = 8.0 Hz); 7.10 (t, 1H, aryl, *J* = 8.0 Hz); 7.19–7.26 (m, 3H, aryl); 7.43 (d, 1H, aryl, *J* = 8.0 Hz); 8.00 (s, 1H, N*H*). ^13^C NMR (100 MHz, CDCl_3_) δ 25.4, 29.2, 30.3, 42.5, 51.8, 53.5, 57.8, 109.5, 111.0, 115.5, 115.7, 118.4, 119.8, 122.2, 127.2, 129.5, 134.2, 134.5, 136.1, 155.3, 161.0, 163.5, 172.5, 174.2. HR-MS *m*/*z* calcd for C_23_H_24_FN_3_O_3_ [(M + H)]^+^: 410.1874; found 410.1879.

### 3-((1*R*,3*S*)-3-((4-Fluorobenzyl)carbamoyl)-2,3,4,9-tetrahydro-1*H*-pyrido[3,4-*b*]indol-1-yl)propanoic Acid (**12a**)

Compound **11a** was dissolved in a mixture of NaOH 6 N/methanol (9/1 v/v) and stirred for 90 min at room temperature. The mixture was then buffered to pH 7 using HCl (6N) and extracted three times with ethyl acetate. The organic phases were collected, dried over Na_2_SO_4_, filtered, and concentrated *in vacuo.* The final product was purified by the use of reverse phase preparative HPLC using a Synergi fusion column (4 μm, 80A, 150 mm × 21.2 mm, Phenomenex Torrence, CA, USA) as stationary phase and a gradient elution with acetonitrile 0.1% TFA (A) and water 0.1% TFA (B) (from 5% to 90% of A in 22 min). Flow rate was set at 20 mL/min. Retention time was 9.10 min. White powder (42% yield). ^1^H NMR (400 MHz, CD_3_OD): δ: 2.01–2.08 (m, 1H, C*H*_*2a*_); 2.11–2.19 (m, 1H, C*H*_*2b*_); 2.47 (t, 2H, C*H*_2_, *J* = 7.0 Hz); 2.88 (dd, 1H, C*H*_*2a*_, *J*′=9.2, *J*″= 15.2 Hz); 3.10 (dd, 1H, C*H*_*2b*_, *J*′ = 4.6, *J*″= 15.3 Hz); 3.90 (dd, 1H, C*H*, *J*′= 4.7, *J*″= 9.3 Hz); 4.20 (dd, 1H, C*H*, *J*′ = 3.6, *J*″= 9.0 Hz); 4.45 (s, 2H, C*H*_2_); 6.96–7.09 (m, 4H, aryl); 7.29–7.35 (m, 3H, aryl); 7.42 (d, aryl, 1H, *J* = 7.7 Hz). ^13^C NMR (100 MHz, CD_3_OD) δ: 24.4, 31.0, 34.8, 41.8, 51.2, 52.5, 106.1, 110.4, 114.6, 114.8, 117.1, 118.2, 120.7, 126.9, 128.9, 129.0, 134.7, 135.6, 136.5, 163.2, 174.1. HR-MS *m*/*z* calcd for C_22_H_22_FN_3_O_3_ [(M + H)]^+^: 396.1718; found 396.1725.

### 3-((1*S*,3*S*)-3-((4-Fluorobenzyl)carbamoyl)-2,3,4,9-tetrahydro-1*H*-pyrido[3,4-*b*]indol-1-yl)propanoic Acid (**12b**)

Compound **12b** was synthesized and purified according to the procedure described for **12a**, using **11b** as starting material. Retention time in RP-HPLC was 8.98 min. The product was isolated as white powder (33% yield). ^1^H NMR (400 MHz, CD_3_OD): δ: 1.92–2.02 (m, 1H, C*H*_*2a*_); 2.35–2.44 (m, 3H, C*H*_2_); 2.71–2.78 (m, 1H, C*H*_*2a*_); 3.09 (dd, 1H, C*H*_*2b*_, *J*′ = 4.4, *J*″ = 15.0 Hz); 3.61 (dd, 1H, C*H*, *J*′= 4.2, *J*″= 11.2 Hz); 4.18 (d, 1H, C*H*, *J* = 8.2 Hz); 4.47 (s, 2H, C*H*_2a_); 6.98 (t, 1H, aryl, *J* = 7.9 Hz); 7.04–7.09 (m, 3H, aryl); 7.31 (d, 1H, aryl, *J* = 8.0 Hz); 7.38–7.41 (m, 3H, aryl). ^13^C NMR (100 MHz, CD_3_OD): δ: 25.6, 30.8, 33.7, 41.8, 53.1, 57.8, 107.1, 110.6, 114.6, 114.8, 117.0, 118.3, 120.6, 127.1, 129.1, 134.8, 135.8, 136.6, 160.9, 163.3, 174.6, 181.2. ^19^F NMR (CDCl_3_, 376.3 MHz) δ: -(118.05) (s, 1F, C*F*).HR-MS *m*/*z* calcd for C_22_H_22_FN_3_O_3_ [(M + H)]^+^: 396.1718; found 396.1729.

### (*S*)-Methyl 2,3,4,9-Tetrahydro-1*H*-pyrido[3,4-*b*]indole-3-carboxylate (**13**)

Intermediate **13** was synthesized in 89% yield according to the general procedure A starting from l-tryptophan methyl ester and formaldehyde. The product was isolated by filtration from the reaction mixture. Spectral data were in accordance with literature.^37^

### (1*R*,3*S*)-Methyl 1-iIsobutyl-2,3,4,9-tetrahydro-1*H*-pyrido[3,4-*b*]indole-3-carboxylate (**14a**)

Synthesized in 36% yield according to the general procedure A starting from tryptophan methyl ester and isovaleraldehyde. FC in hexane/ethyl acetate 1/1, *R_f_* = 0.40. Spectral data were in accordance with literature.^38^

### (1*S*,3*S*)-Methyl 1-Isobutyl-2,3,4,9-tetrahydro-1*H*-pyrido[3,4-*b*]indole-3-carboxylate (**14b**)

Synthesized in 40% yield according to the general procedure A starting from tryptophan methyl ester and isovaleraldehyde. FC in hexane/ethyl acetate 1/1, *R_f_* = 0.44. Spectral data were in accordance with literature.^38^

### (1*R*,3*S*)-Methyl 1-(4-Chlorophenyl)-2,3,4,9-tetrahydro-1*H*-pyrido[3,4-*b*]indole-3-carboxylate (**15a**)

Synthesized in 33% yield according to the general procedure A starting from tryptophan methyl ester and 4-chlorobenzaldehyde. FC in hexane/ethyl acetate 1/1, *R_f_* = 0.45. Spectral data were in accordance with literature.^39^

### (1*S*,3*S*)-Methyl 1-(4-Chlorophenyl)-2,3,4,9-tetrahydro-1*H*-pyrido[3,4-*b*]indole-3-carboxylate (**15b**)

Synthesized in 46% yield according to the general procedure A starting from tryptophan methyl ester and 4-chlorobenzaldehyde. FC in hexane/ethyl acetate 1/1, *R_f_* = 0.51. Spectral data were in accordance with literature.^39^

### (*S*)-Methyl 2-(3-((*tert*-Butoxycarbonyl)amino)propanoyl)-2,3,4,9-tetrahydro-1*H*-pyrido[3,4-*b*]indole-3-carboxylate (**16**)

Synthesized in 71% yield according to the general procedure B starting from intermediate **13** and *N*-Boc-β-Ala-OH. FC ethyl acetate/*n*-hexane 1/2. *R_f_* = 0.65. Spectral data were in accordance with literature.^36^

### (*S*)-Methyl 2-(3-Aminopropanoyl)-2,3,4,9-tetrahydro-1*H*-pyrido[3,4-*b*]indole-3-carboxylate (**17**)

Synthesized from intermediate **16** using the general procedure C. FC in ethyl acetate, *R_f_* = 0.60. White powder (91% yield). [α]^25^_D_: +101.70 ± 0.03. ^1^H NMR (CD_3_OD, 400 MHz): δ: (A) 2.62–2.72 (m, 2H, C*H*_2_); 2.86–2.92 (m, 3H, C*H*_2_ and C*H*_2a_); 3.35 (d, 1H, C*H*_2b_, *J* = 15.6 Hz); 3.50 (s, 3H, C*H*_3_); 4.64 (d, 1H, C*H*_2a_, *J* = 15.4 Hz); 4.82 (d, 1H, C*H*_2b_, *J* = 15.4 Hz); 5.15 (d, 1H, C*H*, *J* = 4.5 Hz); 6.90 (t, 1H, aryl, *J* = 7.2 Hz); 6.95–7.00 (m, 1H, aryl); 7.18 (t, 1H, aryl, *J* = 6.8 Hz); 7.31 (d, 1H, aryl, *J* = 7.8 Hz); ^13^C NMR (CD_3_OD, 100 MHz) δ: ^1^H NMR (CD_3_OD, 400 MHz). δ: (B) 2.49–2.56 (m, 2H, C*H*_2_); 2.86–2.92 (m, 2H, C*H*_2_); 3.01 (dd, 1H, C*H*_2a_, *J*′ = 5.8 and *J*″ = 15.6 Hz); 3.42 (d, 1H, C*H*_2a_, *J* = 15.3 Hz); 3.52 (s, 3H, C*H*_3_); 4.24 (d, 1H, C*H*_2a_, *J* = 17.1 Hz); 5.04 (d, 1H, C*H*_2b_, *J* = 17.1 Hz); 5.74 (d, 1H, C*H*, *J* = 7.5 Hz); 6.90 (t, 1H, aryl, *J* = 7.2 Hz); 6.95–7.00 (m, 1H, aryl); 7.18 (t, 1H, aryl, *J* = 6.8 Hz); 7.31 (d, 1H, aryl, *J* = 7.8 Hz); ^13^C NMR (CD_3_OD, 100 MHz) (A + B) δ: 22.4, 23.2, 29.4, 35.4, 36.8, 37.0, 38.9, 41.2, 51.0, 51.6, 51.8, 55.2, 104.4, 105.0, 110.6, 117.2, 118.7, 121.1, 126.5, 128.6, 129.2, 137.0, 171.3, 171.7, 173.3, 173.5. HR-MS *m*/*z* calcd for C_16_H_19_N_3_O_3_, [(M + H)^+^]: 302.1499; found 302.1503.

### (*S*)-Methyl 2-(2-((*tert*-Butoxycarbonyl)amino)acetyl)-2,3,4,9-tetrahydro-1*H*-pyrido[3,4-*b*]indole-3-carboxylate (**18**)

Compound **18** was synthesized in 71% yield starting from intermediate **13** and *N*-Boc-Gly-OH following the general procedure B. FC in ethyl acetate/*n*-hexane 1/1, *R_f_* = 0.25. Spectral data were in accordance with literature.^36^

### (*S*)-Methyl 2-((*S*)-2-((*tert*-Butoxycarbonyl)amino)-3-phenylpropanoyl)-2,3,4,9-tetrahydro-1*H*-pyrido[3,4-*b*]indole-3-carboxylate (**19**)

Compound **19** was synthesized in 68% yield starting from intermediate **13** and *N*-Boc-*L*-Phe-OH following the general procedure B. FC in ethyl acetate/*n*-hexane 2/3, *R_f_* = 0.30. Spectral data were in accordance with literature.^36^

### (*S*)-Methyl 2-((*R*)-2-((*tert*-Butoxycarbonyl)amino)-3-phenylpropanoyl)-2,3,4,9-tetrahydro-1*H*-pyrido[3,4-*b*]indole-3-carboxylate (**20**)

Compound **20** was synthesized in 65% yield starting from intermediate **13** and *N*-Boc-*D*-Phe-OH following the general procedure B. FC in ethyl acetate/*n*-hexane 2/3, *R_f_* = 0.30. Spectral data were in accordance with literature.^36^

### (*S*)-2,3,6,7,12,12a-Hexahydropyrazino[1′,2′:1,6]pyrido[3,4-*b*]indole-1,4-dione (**21**)

Synthesized from **18** following the general procedure C. FC in ethyl acetate/*n*-hexane 3/1, *R_f_* = 0.32. White powder (85% yield). [α]^25^_D_: +37.40 ± 0.01. ^1^H NMR (CD_3_OD, 400 MHz): δ: 2.99 (t, 1H, C*H*_2a_, *J* = 13.1 Hz); 3.32–3.41 (m, 1H, C*H*_2b_,); 4.05 (d, 1H, C*H*_2a_, *J* = 18.0 Hz); 4.17 (d, 1H, C*H*_2b_, *J* = 18.0 Hz); 4.27 (d, 1H, C*H*_2a_, *J* = 16.6 Hz); 4.37 (dd, 1H, C*H*, *J*′ = 7.6, *J*″ = 11.8 Hz); 5.55 (d, 1H, C*H*_2b_, *J* = 16.6 Hz); 7.03 (t, 1H, aryl, *J* = 7.8 Hz); 7.11 (t, 1H, aryl, *J* = 8.1 Hz); 7.33 (d, 1H, aryl, *J* = 8.1 Hz); 7.45 (d, 1H, aryl, *J* = 7.8 Hz). ^13^C NMR (CD_3_OD, 100 MHz) δ: 26.3, 39.7, 43.9, 56.5, 105.6, 110.6, 117.2, 118.8, 121.2, 128. HR-MS *m*/*z*: calcd. for C_14_H_13_N_3_O_2_, [(M + H)^+^]: 256,1081; found 256.1087.

### (3*S*,12a*S*)-3-Benzyl-2,3,12,12a-tetrahydropyrazino[1′,2′:1,6]pyrido[3,4-*b*]indole-1,4(6*H*,7*H*)-dione (**22**)

Synthesized from **19** following the general procedure C. FC in ethyl acetate, *R_f_* = 0.25. White powder (81% yield). [α]^25^_D_: −68.000 ± 0.00108 (*c* = 0.10, MeOH). ^1^H NMR (CD_3_OD, 400 MHz): δ: 0.89 (t, 1H, C*H*_2a_, *J* = 12.8 Hz); 2.73 (dd, 1H, C*H*_2b_, *J*′ = 5.8, *J*″ = 15.1 Hz); 2.99 (dd, 1H, C*H*_2a_, *J*′ = 4.8, *J*″ = 13.7 Hz); 3.37 (dd, 1H, C*H*_2b_, *J*′ = 5.3, *J*″ = 13.7 Hz); 4.06–4.15 (m, 2H, C*H* and C*H*_2a_); 4.49 (t, 1H, C*H*, *J* = 4.0 Hz); 5.51 (d, 1H, C*H*_2b_, *J* = 16.5 Hz); 6.98 (t, 1H, aryl, *J* = 7.0 Hz); 7.01–7.12 (m, 6H, aryl); 7.19 (d, 1H, aryl, *J* = 7.8 Hz); 7.29 (d, 1H, aryl, *J* = 8.1 Hz). ^13^C NMR (CD_3_OD, 100 MHz) δ: 25.8, 39.6, 56.1, 105.8, 110.5, 117.1, 118.6, 121.0, 126.9, 126.9, 127.9, 128.2, 130.1, 135.0, 136.5, 164.9, 167.9. HR-MS *m*/*z*: calcd. for C_21_H_19_N_3_O_2_, [(M + H)^+^]: 346.1550; found 346.1556.

### (3*R*,12a*S*)-3-Benzyl-2,3,12,12a-tetrahydropyrazino[1′,2′:1,6]pyrido[3,4-*b*]indole-1,4(6*H*,7*H*)-dione (**23**)

Synthesized from **20** following the general procedure C. FC in ethyl acetate, *R_f_* = 0.20. White powder (80% yield). [α]^25^_D_: −102.7 ± 0.2 (*c* = 0.10, MeOH). ^1^H NMR (CDCl_3_, 400 MHz): δ: 2.78 (t, 1H, C*H*_2a_, *J* = 15.3 Hz); 3.02 (dd, 1H, C*H*_2b_, *J*′ = 8.1, *J*″ = 13.8 Hz); 3.32–3.36 (m, 2H, C*H*_2a_ and C*H*_2a_); 3.67 (dd, 1H, C*H*, *J*′ = 4.3, *J*″ = 11.2 Hz); 4.04 (d, 1H, C*H*_2a_, *J* = 16.8 Hz); 4.31 (dd, 1H, C*H*, *J*′ = 3.5, *J*″ = 7.9 Hz); 5.48 (d, 1H, C*H*_2b_, *J* = 16.8 Hz); 5.79 (s, 1NH); 7.05 (t, 1H, aryl, *J* = 7.0 Hz); 7.12 (t, 1H, aryl, *J* = 7.1 Hz); 7.16–7.30 (m, 6H, aryl); 7.38 (d, 1H, aryl, *J* = 7.7 Hz); 7.81 (s, 1NH); ^13^C NMR (CDCl_3_, 100 MHz) δ: 28.0, 41.4, 42.1, 56.8, 56.9, 108.4, 112.0, 119.3, 121.0, 123.4, 127.3, 128.7, 129.2, 130.2, 130.77, 136.1, 137.3, 163.9, 166.1. HR-MS *m*/*z*: calcd for C_21_H_19_N_3_O_2_, [(M + H)^+^]: 346.1550; found 346.1541.

### (1*R*,3*S*)-Methyl 2-((*S*)-2-((*tert*-Butoxycarbonyl)amino)-3-phenylpropanoyl)-1-isobutyl-2,3,4,9-tetrahydro-1*H*-pyrido[3,4-*b*]indole-3-carboxylate (**24a**)

Compound **24a** was synthesized in 25% yield starting from intermediate **14a** and *N*-Boc-*L*-Phe-OH following the general procedure B. FC in ethyl acetate/*n*-hexane 1/2, *R_f_* = 0.60. Spectral data were in accordance with literature.^36^

### (1*S*,3*S*)-Methyl 2-((*S*)-2-((*tert*-Butoxycarbonyl)amino)-3-phenylpropanoyl)-1-isobutyl-2,3,4,9-tetrahydro-1*H*-pyrido[3,4-*b*]indole-3-carboxylate (**24b**)

Compound **24b** was synthesized in 29% yield starting from intermediate **14b** and *N*-Boc-*L*-Phe-OH following the general procedure B. FC in ethyl acetate/*n*-hexane 1/2, *R_f_* = 0.65. Spectral data were in accordance with literature.^36^

### (3*S*,6*R*,12a*S*)-3-Benzyl-6-isobutyl-2,3,12,12a-tetrahydropyrazino[1′,2′:1,6]pyrido[3,4-*b*]indole-1,4(6*H*,7*H*)-dione (**25a**)

Obtained from **24a** following the general procedure C. FC in ethyl acetate/*n*-hexane 1/1, *R_f_* = 0.32. White powder (78% yield). [α]^25^_D_: −201.40 ± 0.01. ^1^H NMR (400 MHz, CDCl_3_): δ: 1.02 (d, 3H, C*H*_3_, *J* = 6.4 Hz); 1.14 (d, 3H, C*H*_3_, *J* = 6.3 Hz); 1.62–1.84 (m, 4H, C*H,* C*H*_2a_ and C*H*_2_); 3.12 (dd, 1H, C*H*_2b_, *J*′ = 4.6, *J*″ = 15.4 Hz); 3.18 (d, 2H, C*H*_2_, *J* = 5.5 Hz); 4.30 (dd, 1H, C*H*, *J*′ = 4.5, *J*″ = 11.8 Hz); 4.43–4.46 (m, 1H, C*H*); 5.97–6.00 (m, 1H, C*H*); 6.26 (s, 1NH); 7.12–7.24 (m, 7H, aryl); 7.36 (t, 2H, aryl, *J* = 7.8 Hz); 7.84 (s, 1NH). ^13^C NMR (100 MHz, CDCl_3_) δ 18.5, 19.3, 21.3, 23.3, 37.7, 39.7, 43.9, 48.4, 53.0, 102.7, 106.9, 114.2, 116.0, 118.4, 122.5, 123.6, 125.1, 125.9, 128.8, 131.1, 132.0, 160.3, 163.6. HR-MS *m*/*z* calcd for C_25_H_27_N_3_O_2_ [(M + H)]^+^: 402.2176; found 402.2189.

### (3*S*,6*S*,12a*S*)-3-Benzyl-6-isobutyl-2,3,12,12a-tetrahydropyrazino[1′,2′:1,6]pyrido[3,4-*b*]indole-1,4(6*H*,7*H*)-dione (**25b**)

Obtained from **24b** following the general procedure C. FC in ethyl acetate/*n*-hexane 1/1, *R_f_* = 0.38. White powder (73% yield). [α]^25^_D_: −122.36 ± 0.01. ^1^H NMR (400 MHz, CDCl_3_): δ: 0.78 (d, 3H, C*H*_3_, *J* = 6.3 Hz); 1.02 (d, 3H, C*H*_3_, *J* = 6.4 Hz); 1.49–1.55 (m, 2H, C*H*_2_); 1.72–1.79 (m, 1H, C*H*); 2.81 (dd, 1H, C*H*_2a_, *J*′ = 10.8, *J*″ = 14.8 Hz); 2.94 (dd, 1H, C*H*_2a_, *J*′ = 11.7, *J*″ = 15.6 Hz); 3.51 (dd, 1H, C*H*_2b_, *J*′ = 4.7, *J*″ = 15.7 Hz,); 3.63 (dd, 1H, C*H*_2b_, *J*′ = 3.4, *J*″ = 14.4 Hz,); 3.98 (dd, 1H, C*H*, *J*′ = 4.5, *J*″ = 11.6 Hz); 4.14 (dd, 1H, C*H*, *J*′ = 3.4, *J*″ = 10.6 Hz); 5.45 (dd, 1H, C*H*, *J*′ = 4.0, *J*″= 9.2 Hz); 5.67 (s, 1NH); 7.07–7.16 (m, 2H, aryl); 7.19 (d, 2H, aryl, *J* = 5.6 Hz); 7.25 (d, 1H, aryl, *J* = 6.9 Hz); 7.31 (t, 3H, aryl, *J* = 7.9 Hz); 7.50 (d, 1H, aryl, *J* = 7.6 Hz); 7.95 (s, 1NH). ^13^C NMR (100 MHz, CDCl_3_) δ 17.7, 18.1, 19.9, 27.0, 33.3, 42.0, 47.3, 51.1, 52.2, 103.0, 107.2, 114.3, 116.2, 118.3, 122.2, 125.2, 125.4, 130.3, 131.8, 164.3, 165.2. HR-MS *m*/*z* calcd for C_25_H_27_N_3_O_2_ [(M + H)]^+^: 402.2176; found 402.2190.

### (1*R*,3*S*)-Methyl 2-(2-((*tert*-Butoxycarbonyl)amino)acetyl)-1-(4-chlorophenyl)-2,3,4,9-tetrahydro-1*H*-pyrido[3,4-*b*]indole-3-carboxylate (**26a**)

Compound **26a** was synthesized in 62% yield starting from intermediate **15a** and *N*-Boc-Gly-OH following the general procedure B. FC in ethyl acetate/*n*-hexane 1/2, *R_f_* = 0.35. Spectral data were in accordance with literature.^36^

### (1*S*,3*S*)-Methyl 2-(2-((*tert*-Butoxycarbonyl)amino)acetyl)-1-(4-chlorophenyl)-2,3,4,9-tetrahydro-1*H*-pyrido[3,4-*b*]indole-3-carboxylate (**26b**)

Compound **26b** was synthesized in 52% yield starting from intermediate **15b** and *N*-Boc-Gly-OH following the general procedure B. FC in ethyl acetate/*n*-hexane 1/2, *R_f_* = 0.45. Spectral data were in accordance with literature.^36^

### (6*R*,12a*S*)-6-(4-Chlorophenyl)-2,3,12,12a-tetrahydropyrazino[1′,2′:1,6]pyrido[3,4-*b*]indole-1,4(6*H*,7*H*)-dione (**27a**)

Obtained from **26a** following the general procedure C. FC in ethyl acetate/*n*-hexane 4/1, *R_f_* = 0.33. White powder (82% yield). [α]^25^_D_: −244.0 ± 0.2. ^1^H NMR (CDCl_3_, 400 MHz): δ: 2.96 (dd, 1H, C*H*_2a_, *J*′ = 12.0, *J*″ = 16.6 Hz); 3.46 (dd, 1H, C*H*_2a_, *J*′ = 4.2, *J*″ = 15.5 Hz); 4.02 (d, 1H, C*H*_2a_, *J* = 17.7 Hz); 4.12 (d, 1H, C*H*_2b_, *J* = 17.7 Hz); 4.18 (dd, 1H, C*H*, *J*′ = 4.2, *J*″ = 12.0 Hz); 6.67 (s, 1H, C*H*); 6.97 (s, 1NH); 7.08–7.26 (m, 7H, aryl); 7.48 (d, 1H, aryl, *J* = 7.7 Hz); 8.00 (s, 1NH). ^13^C NMR (CDCl_3_, 100 MHz) δ: 27.2, 44.8, 51.5, 52.3, 109.1, 111.2, 118.5, 120.3, 123.0, 126.2, 129.1, 129.2, 130.1, 135.0, 136.4, 136.7, 161.8, 167.5. HR-MS *m*/*z* calcd for C_20_H_16_ClN_3_O_2_, 366.1004; found 366.1011.

### (6*S*,12a*S*)-6-(4-Chlorophenyl)-2,3,12,12a-tetrahydropyrazino[1′,2′:1,6]pyrido[3,4-*b*]indole-1,4(6*H*,7*H*)-dione (**27b**)

Obtained from **26b** following the general procedure C. FC in ethyl acetate/*n*-hexane 4/1, *R_f_* = 0.37. White powder (76% yield). [α]^25^_D_: −79 ± 0.01. ^1^H NMR (CDCl_3_, 400 MHz): δ: 3.17 (dd, 1H, C*H*_2a_, *J*′ = 10.3, *J*″ = 16.0 Hz); 3.67 (dd, 1H, C*H*_2b_, *J*′ = 4.6, *J*″ = 16.0 Hz); 3.93–4.06 (m, 2H, C*H*_2_); 4.30 (dd, 1H, C*H*, *J*′ = 4.5, *J*″ = 11.5 Hz); 6.16 (s, 1H, C*H*); 6.26 (s, 1NH); 7.07–7.22 (m, 7H, aryl); 7.53 (d, 1H, aryl, *J* = 8.4 Hz); 7.80 (s, 1NH). ^13^C NMR (CDCl_3_, 100 MHz) δ: 23.4, 45.3, 55.8, 56.3, 106.6, 111.3, 118.6, 120.3, 122.8, 126.1, 128.6, 128.9, 132.2, 133.7, 136.6, 139.8, 167.1, 168.5. HR-MS *m*/*z* calcd for C_20_H_16_ClN_3_O_2_ [(M + H)]^+^: 366.1004; found 366.1009.

### (*S*)-2-(4-Fluorobenzyl)-5,6,11,11a-tetrahydro-1*H*-imidazo[1′,5′:1,6]pyrido[3,4-*b*]indole-1,3(2*H*)-dione (**28**)

Obtained from **13** and 4-fluorobenzylamine following the general procedure D. FC in hexane/ethyl acetate 3/2, *R_f_* = 0.45. White powder (65% yield). ^1^H NMR (400 MHz, CDCl_3_): δ: 2.78 (dd, 1H, C*H*_*2a*_, *J*′ = 12.8, *J*″ = 13.1 Hz); 3.41 (dd, 1H, C*H*_*2b*_, *J*′= 5.3, *J*″= 15.1 Hz,); 4.25 (dd, 1H, C*H*, *J*′ = 5.5, *J*″ = 11.0 Hz); 4.40 (d, 1H, C*H*_*2a*_, *J* = 16.1 Hz); 4.73 (s, 2H, C*H*_2_); 5.10 (d, 1H, C*H*_*2b*_, *J* = 16.1 Hz); 7.03 (t, 2H, aryl, *J* = 8.6 Hz); 7.17 (t, 1H, aryl, *J* = 7.4 Hz); 7.23 (t, 1H, aryl, *J* = 7.1 Hz); 7.34 (d, 1H, aryl, *J* = 8.0 Hz); 7.43–7.46 (m, 2H, aryl); 7.50 (d, 1H, aryl, *J* = 7.7 Hz); 8.15 (s, 1H, N*H*). ^13^C NMR (100 MHz, CDCl_3_) δ: 23.1, 37.8, 41.7, 55.3, 106.3, 111.0, 115.5, 115.7, 118.1, 120.2, 122.7, 126.4, 128.3, 130.6, 131.9, 136.5, 155.1, 161.3, 163.7, 172.5. HR-MS *m*/*z* calcd for C_20_H_16_FN_3_O_2_ [(M + H)]^+^: 350.1299; found 350.1307.

### (5*R*,11a*S*)-5-Isobutyl-2-(4-methoxybenzyl)-5,6,11,11a-tetrahydro-1*H*-imidazo[1′,5′:1,6]pyrido[3,4-*b*]indole-1,3(2*H*)-dione (**29a**)

Obtained from **14a** and 4-methoxybenzylamine following the general procedure D. FC in hexane/ethyl acetate 1/1, *R_f_* = 0.38. White powder (32% yield). [α]^25^_D_: −92.360 ± 0.179 (*c* = 0.10, MeOH). ^1^H NMR (400 MHz, CDCl_3_) δ 0.98 (d, 3H, C*H*_3_, *J* = 8.2 Hz); 1.17 (d, 3H, C*H*_3_, *J* = 8.8 Hz); 1.68–1.77 (m, 2H, C*H*_2_); 1.79–1.88 (m, 1H, C*H*); 2.75 (dd, 1H, C*H*_2a_, *J*′ = 12.3, *J*″ = 17.4 Hz); 3.37 (dd, 1H, C*H*_2b_, *J*′ = 6.8, *J*″= 19.4 Hz); 3.79 (s, 3H, C*H*_3_); 4.30 (dd, 1H, C*H, J*′ = 7.8, *J*″ = 14.8 Hz); 4.62 (d, 1H, C*H*_2a_, *J* = 18.5 Hz,); 4.75 (d, 1H, C*H*_2b_, *J* = 18.5 Hz); 5.29–5.34 (m, 1H, C*H*); 6.87 (d, 2H, aryl, *J* = 10.0 Hz); 7.14 (t, 1H, aryl, *J* = 9.0 Hz); 7.24 (t, 1H, aryl, *J* = 9.0 Hz); 7.33–7.40 (m, 3H, aryl); 7.47 (d, 1H, aryl, *J* = 8.8 Hz); 7.89 (s, 1NH). ^13^C NMR (100 MHz, CDCl_3_) δ 22.2, 23.5, 23.6, 25.0, 41.8, 45.8, 46.9, 52.9, 55.2, 105.7, 111.0, 114.1, 118.2, 120.1, 122.6, 126.3, 128.5, 130.2, 133.2, 136.2, 155.3, 159.2, 172.9. HR-MS *m*/*z* calcd for C_25_H_27_N_3_O_3_ [(M + H)]^+^: 418.2125; found 418.2139.

### (5*S*,11a*R*)-5-Isobutyl-2-(4-methoxybenzyl)-5,6,11,11a-tetrahydro-1*H*-imidazo[1′,5′:1,6]pyrido[3,4-*b*]indole-1,3(2*H*)-dione (**29a′**)

Obtained from **14b** and 4-methoxybenzylamine following the general procedure D. FC in hexane/ethyl acetate 1/1, *R_f_* = 0.44. White powder (43% yield). [α]^25^_D_: +98.563 ± 0.158 (*c* = 0.10, MeOH). ^1^H NMR (400 MHz, CDCl_3_) δ 0.98 (d, 3H, C*H*_3_, *J* = 8.7 Hz); 1.17 (d, 3H, C*H*_3_, *J* = 8.7 Hz); 1.65–1.77 (m, 2H, C*H*_2_); 1.80–1.88 (m, 1H, C*H*); 2.76 (dd, 1H, C*H*_2a_, *J*′ = 14.5, *J*″ = 18.8 Hz); 3.37 (dd, 1H, C*H*_2b_*J*′ = 7.8, *J*″ = 20.4 Hz); 3.79 (s, 3H, C*H*_3_); 4.30 (dd, 1H, C*H, J*′ = 7.7, *J*″ = 14.4 Hz); 4.62 (d, 1H, C*H*_2a_, *J* = 19.2 Hz); 4.75 (d, 1H, C*H*_2b_, *J* = 19.2 Hz); 5.30–5.34 (m, 1H, C*H*); 6.86 (d, 2H, aryl, *J* = 11.5 Hz); 7.15 (t, 1H, aryl, *J* = 9.3 Hz); 7.22 (t, 1H, aryl, *J* = 9.1 Hz); 7.28–7.40 (m, 3H, aryl); 7.47 (d, 1H, aryl, *J* = 10.4 Hz); 7.89 (s, 1NH). ^13^C NMR (100 MHz, CDCl_3_) δ 22.2, 23.5, 23.6, 25.0, 41.8, 45.8, 46.9, 52.9, 55.2, 105.7, 111.0, 114.1, 118.2, 120.1, 122.6, 126.3, 128.5, 130.0, 133.1, 136.2, 155.3, 159.3, 172.9. HR-MS *m*/*z* calcd for C_25_H_27_N_3_O_3_ [(M + H)]^+^: 418.2125; found 418.2132.

### (5*R*,11a*S*)-5-(4-Chlorophenyl)-2-(4-methylbenzyl)-5,6,11,11a-tetrahydro-1*H*-imidazo[1′,5′:1,6]pyrido[3,4-*b*]indole-1,3(2*H*)-dione (**30a**)

Obtained from **15a** and 4-methylbenzylamine following the general procedure D. FC in dichloromethane/ethyl acetate 9.8/0.2, *R_f_* = 0.42. White powder (38% yield). [α]^25^_D_: −181.00 ± 0.10 (*c* = 0.10, MeOH). ^1^H NMR (400 MHz, CDCl_3_): δ: 2.32 (s, 3H, C*H*_3_); 2.83 (dd, 1H, C*H*_*2a*_, *J*′ = 11.3, *J*″ = 14.8 Hz); 3.49 (dd, 1H, C*H*_*2b*_, *J*′= 5.3, *J*″ = 15.3 Hz); 4.30 (dd, 1H, C*H, J*′ = 5.3, *J*″ = 10.8 Hz); 4.61 (d, 1H, C*H*_2a_, *J* = 14.4 Hz); 4.71 (d, 1H, C*H*_2b_, *J* = 14.4 Hz,); 6.29 (s, 1H, C*H*); 7.13 (d, 2H, aryl, *J* = 7.6 Hz); 7.18–7.36 (m, 9H, aryl); 7.55 (d, 1H, aryl, *J* = 7.6 Hz); 7.72 (s, 1NH). ^13^C NMR (100 MHz, CDCl_3_) δ 21.1, 23.3, 42.2, 51.4, 53.2, 108.4, 111.2, 118.5, 120.3, 123.1, 126.1, 128.7, 129.4, 129.8, 133.0, 135.0, 136.6, 137.5, 137.8, 154.7, 172.2. HR-MS *m*/*z* calcd for C_27_H_22_ClN_3_O_2_ [(M + H)]^+^: 456.1473; found 456.1480.

### (5*S*,11a*R*)-5-(4-Chlorophenyl)-2-(4-methylbenzyl)-5,6,11,11a-tetrahydro-1*H*-imidazo[1′,5′:1,6]pyrido[3,4-*b*]indole-1,3(2*H*)-dione (**30a′**)

Obtained from **15b** and 4-methylbenzylamine following the general procedure D. FC in dichloromethane/ethyl acetate 9.8/0.2, *R_f_* = 0.42. White powder (41% yield). [α]^25^_D_: +175.00 ± 0.02 (*c* = 0.10, MeOH). ^1^H NMR (400 MHz, CDCl_3_): δ: 2.32 (s, 3H, C*H*_3_); 2.84 (dd, 1H, C*H*_*2a*_, *J*′ = 11.3, *J*″ = 14.8 Hz); 3.49 (dd, 1H, C*H*_*2b*_, *J*′= 5.3, *J*″ = 15.3 Hz); 4.30 (dd, 1H, C*H, J*′ = 5.3, *J*″ = 10.8 Hz); 4.61 (d, 1H, C*H*_2a_, *J* = 14.4 Hz); 4.71 (d, 1H, C*H*_2b_, *J* = 14.4 Hz,); 6.29 (s, 1H, C*H*); 7.13 (d, 2H, aryl, *J* = 7.6 Hz); 7.20–7.35 (m, 9H, aryl); 7.54 (d, 1H, aryl, *J* = 7.6 Hz); 7.72 (s, 1NH). ^13^C NMR (100 MHz, CD_3_OD) δ 21.1, 23.3, 42.2, 51.4, 53.2, 108.4, 111.2, 118.5, 120.3, 123.1, 126.0, 128.7, 129.8, 133.0, 135.0, 136.6, 137.5, 137.8, 154.7, 172.2. HR-MS *m*/*z* calcd for C_27_H_22_ClN_3_O_2_ [(M + H)]^+^: 456.1473; found 456.1478.

### (5*R*,11a*S*)-5-(4-Chlorophenyl)-2-(4-fluorobenzyl)-5,6,11,11a-tetrahydro-1*H*-imidazo[1′,5′:1,6]pyrido[3,4-*b*]indole-1,3(2*H*)-dione (**31a**)

Obtained from **15a** and 4-fluorobenzylamine following the general procedure D. FC in dichloromethane/ethyl acetate 8/2, *R_f_* = 0.40. White powder (33% yield). [α]^25^_D_: −105.00 ± 0.10 (*c* = 0.10, MeOH). ^1^H NMR (400 MHz, CDCl_3_): δ: 2.87 (t, 1H, C*H*_*2a*_, *J* = 12.6 Hz); 3.51 (dd, 1H, C*H*_*2b*_, *J*′= 4.2, *J*″ = 14.2 Hz); 4.31 (dd, 1H, C*H, J*′ = 4.9, *J*″ = 10.4 Hz); 4.60 (d, 1H, C*H*_2a_, *J* = 14.5 Hz); 4.70 (d, 1H, C*H*_2b_, *J* = 14.5 Hz,); 6.28 (s, 1H, C*H*); 7.00 (t, 2H, aryl, *J* = 8.1 Hz); 7.18–7.41 (m, 9H, aryl); 7.57 (d, 1H, aryl, *J* = 7.4 Hz); 7.84 (s, 1NH). ^13^C NMR (100 MHz, CDCl_3_) δ 23.3, 41.7, 51.4, 53.3, 108.4, 111.3, 115.5, 115.7, 118.5, 120.4, 123.2, 126.0, 129.4, 129.6, 129.7, 130.6, 131.8, 135.0, 136.6, 137.4, 154.6, 161.3, 163.7, 172.2. HR-MS *m*/*z* calcd for C_26_H_19_ClFN_3_O_2_ [(M + H)]^+^: 460.1223; found 460.1218.

### (5*S*,11a*R*)-5-(4-Chlorophenyl)-2-(4-fluorobenzyl)-5,6,11,11a-tetrahydro-1*H*-imidazo[1′,5′:1,6]pyrido[3,4-*b*]indole-1,3(2*H*)-dione (**31a′**)

Obtained from **15b** and 4-fluorobenzylamine following the general procedure D. FC in dichloromethane/ethyl acetate 8/2, *R_f_* = 0.40. White powder (41% yield). [α]^25^_D_: +97.00 ± 0.02 (*c* = 0.10, MeOH). ^1^H NMR (400 MHz, CDCl_3_): δ: 2.86 (dd, 1H, C*H*_*2a*_, *J*′ = 11.1, *J*″ = 12.9 Hz); 3.50 (dd, 1H, C*H*_*2b*_, *J*′= 5.5, *J*″ = 15.4 Hz); 4.30 (dd, 1H, C*H, J*′ = 5.5, *J*″ = 11.0 Hz); 4.60 (d, 1H, C*H*_2a_, *J* = 14.5 Hz); 4.69 (d, 1H, C*H*_2b_, *J* = 14.5 Hz,); 6.28 (s, 1H, C*H*); 6.99 (t, 2H, aryl, *J* = 8.6 Hz); 7.16–7.41 (m, 9H, aryl); 7.55 (d, 1H, aryl, *J* = 7.6 Hz); 7.75 (s, 1NH). ^13^C NMR (100 MHz, CDCl_3_) δ 23.3, 41.7, 51.5, 53.3, 108.4, 111.2, 115.5, 115.7, 118.5, 120.4, 123.2, 126.0, 129.4, 129.6, 129.7, 130.6, 131.8, 135.0, 136.6, 137.4, 154.6, 161.3, 163.7, 172.2. HR-MS *m*/*z* calcd for C_26_H_19_ClFN_3_O_2_ [(M + H)]^+^: 460.1223; found 460.1215.

### (1*R*,3*S*)-Methyl 1-(4-Fluorophenyl)-2,3,4,9-tetrahydro-1*H*-pyrido[3,4-*b*]indole-3-carboxylate (**32a**)

Synthesized in 35% yield from l-tryptophan methyl ester and 4-fluorobenzaldehyde following the general procedure A, as previously described.^39^ FC in ethyl acetate/*n*-hexane 1/2, *R_f_* = 0.36. Spectral data were in accordance with literature.

### (1*S*,3*S*)-Methyl 1-(4-Fluorophenyl)-2,3,4,9-tetrahydro-1*H*-pyrido[3,4-*b*]indole-3-carboxylate (**32b**)

Synthesized in 44% yield from l-tryptophan methyl ester and 4-fluorobenzaldehyde following the general procedure A, as previously described.^39^ FC in ethyl acetate/*n*-hexane 1/2, *R_f_* = 0.41. Spectral data were in accordance with literature.

### (5*R*,11a*S*)-2-Benzyl-5-(4-fluorophenyl)-5,6,11,11a-tetrahydro-1*H*-imidazo[1′,5′:1,6]pyrido[3,4-*b*]indole-1,3(2*H*)-dione (**33a**)

Obtained from **32a** and benzylamine following the general procedure D. FC in hexane/ethyl acetate 7/3, *R_f_* = 0.48. White powder (36% yield). [α]^25^_D_: −113.529 ± 0.182 (*c* = 0.10, MeOH). ^1^H NMR (400 MHz, CDCl_3_): δ: 2.75 (dd, 1H, C*H*_*2a*_, *J*′ = 11.8, *J*″ = 16.0 Hz); 3.40 (dd, 1H, C*H*_*2b*_, *J*′ = 5.5, *J*″ = 11.8 Hz); 4.22 (dd, 1H, C*H, J*′ = 5.5, *J*″ = 11.0 Hz); 4.61 (d, 1H, C*H*_2a_, *J* = 14.5 Hz); 4.67 (d, 1H, C*H*_2b_, *J* = 14.5 Hz,); 6.22 (s, 1H, C*H*); 6.96 (t, 2H, aryl, *J* = 8.6 Hz); 7.10–7.25 (m, 8H, aryl); 7.32 (d, 2H, aryl, *J* = 8.1 Hz); 7.46 (d, 1H, aryl, *J* = 7.7 Hz); 7.68 (s, 1NH). ^13^C NMR (100 MHz, CDCl_3_) δ 23.4, 42.4, 51.4, 53.2, 108.3, 111.2, 116.0, 116.2, 118.5, 120.3, 123.1, 126.1, 128.0, 128.7, 130.1, 134.9, 136.0, 136.6, 154.7, 161.7, 164.2, 172.3. HR-MS *m*/*z* calcd for C_26_H_20_FN_3_O_2_ [(M + H)]^+^: 426.1612; found 426.1619.

### (5*S*,11a*R*)-2-Benzyl-5-(4-fluorophenyl)-5,6,11,11a-tetrahydro-1*H*-imidazo[1′,5′:1,6]pyrido[3,4-*b*]indole-1,3(2*H*)-dione (**33a′**)

Obtained from **32b** and benzylamine following the general procedure D. FC in hexane/ethyl acetate 7/3, *R_f_* = 0.48. White powder (40% yield). [α]^25^_D_: +124.615 ± 0.162. ^1^H NMR (400 MHz, CDCl_3_): δ: 2.79 (dd, 1H, C*H*_*2a*_, *J*′ = 11.1, *J*″ = 15.3 Hz); 3.47 (dd, 1H, C*H*_*2b*_, *J*′ = 5.4, *J*″ = 11.4 Hz); 4.30 (dd, 1H, C*H, J*′ = 5.5, *J*″ = 11.0 Hz); 4.63 (d, 1H, C*H*_2a_, *J* = 14.5 Hz); 4.75 (d, 1H, C*H*_2b_, *J* = 14.6 Hz); 6.30 (s, 1H, C*H*); 7.04 (t, 2H, aryl, *J* = 8.6 Hz); 7.17–7.35 (m, 8H, aryl); 7.41–7.44 (m, 2H, aryl); 7.54 (d, 1H, aryl, *J* = 7.7 Hz); 7.78 (s, 1NH). ^13^C NMR (100 MHz, CDCl_3_) δ 23.3, 42.4, 51.3, 53.2, 108.3, 111.2, 116.0, 116.2, 118.5, 120.3, 123.1, 126.0, 128.0, 128.7, 130.1, 134.9, 136.0, 136.6, 154.6, 161.7, 164.1, 172.3. HR-MS *m*/*z* calcd for C_26_H_20_FN_3_O_2_ [(M + H)]^+^: 426.1612; found 426.1621.

### *tert*-Butyl (3-((5*R*,11a*S*)-5-(4-Fluorophenyl)-1,3-dioxo-11,11a-dihydro-1*H*-imidazo[1′,5′:1,6]pyrido[3,4-*b*]indol-2(3*H*,5*H*,6*H*)-yl)propyl)carbamate (**34a**)

Synthesized from **32a** and *N*-Boc-diaminopropane following the general procedure D. FC in *n*-hexane/ethyl acetate 2/1, *R_f_* = 0.55. White powder (49% yield). [α]^25^_D_: −166.70 ± 0.35 (*c* = 0.10, MeOH). ^1^H NMR (400 MHz, CDCl_3_) δ 1.40 (s, 9H, C*H*_3_); 1.73–1.80 (m, 2H, C*H*_2_); 2.89 (dd, 1H, C*H*_2a_, *J*′ = 11.5, *J*″ = 14.2 Hz,) 3.09–3.12 (m, 2H, C*H*_2_); 3.51 (dd, 1H, C*H*_*2b*_, *J*′ = 5.5, *J*″ = 15.5 Hz); 3.57–3.66 (m, 2H, C*H*_2_); 4.28 (dd, 1H, C*H, J*′ = 5.4, *J*″ = 11.0 Hz); 5.19 (bs, 1N*H*); 6.31 (s, 1H, C*H*); 7.02 (t, 2H, aryl, *J* = 8.4 Hz); 7.19 (t, 1H, aryl, *J* = 7.1 Hz); 7.25 (t, 1H, aryl, *J* = 7.1 Hz); 7.31–7.35 (m, 3H, aryl); 7.55 (d, 1H, aryl, *J* = 7.7 Hz); 8.22 (bs, 1N*H*). HR-MS *m*/*z* calcd for C_27_H_29_FN_4_O_4_ [(M + H)]^+^: 493.2246; found 493.2252.

### *tert*-Butyl (3-((5*S*,11a*R*)-5-(4-Fluorophenyl)-1,3-dioxo-11,11a-dihydro-1*H*-imidazo[1′,5′:1,6]pyrido[3,4-*b*]indol-2(3*H*,5*H*,6*H*)-yl)propyl)carbamate (**34a′**)

Synthesized from **32b** and *N*-Boc-diaminopropane following the general procedure D. FC in *n*-hexane/ethyl acetate 2/1, *R_f_* = 0.55. White powder (57% yield). [α]^25^_D_: +125.36 ± 0.40 (*c* = 0.10, MeOH). ^1^H NMR (400 MHz, CDCl_3_) δ 1.43 (s, 9H, C*H*_3_); 1.75–1.81 (m, 2H, C*H*_2_); 2.88 (dd, 1H, C*H*_2a_, *J*′ = 11.3, *J*″ = 14.0 Hz) 3.08–3.11 (m, 2H, C*H*_2_); 3.51 (dd, 1H, C*H*_*2b*_, *J*′ = 5.6, *J*″ = 15.4 Hz); 3.55–3.67 (m, 2H, C*H*_2_); 4.29 (dd, 1H, C*H, J*′ = 5.5, *J*″ = 11.1 Hz); 5.15 (bs, 1N*H*); 6.31 (s, 1H, C*H*); 7.04 (t, 2H, aryl, *J* = 8.6 Hz); 7.19 (t, 1H, aryl, *J* = 6.9 Hz); 7.24 (t, 1H, aryl, *J* = 7.0 Hz); 7.31–7.34 (m, 3H, aryl); 7.56 (d, 1H, aryl, *J* = 7.7 Hz); 8.24 (bs, 1N*H*). HR-MS *m*/*z* calcd for C_27_H_29_FN_4_O_4_ [(M + H)]^+^: 493.2246; found 493.2250.

### (5*R*,11a*S*)-2-(3-Aminopropyl)-5-(4-fluorophenyl)-5,6,11,11a-tetrahydro-1*H*-imidazo[1′,5′:1,6]pyrido[3,4-*b*]indole-1,3(2*H*)-dione (**35a**)

Synthesized according to the general procedure C starting from intermediate **34**. FC in dichloromethane/methanol 9/1, *R_f_* = 0.47. White powder (35% yield). [α]^25^_D_: −102.500 ± 0.075 (*c* = 0.10, MeOH). ^1^H NMR (400 MHz, CD_3_OD) δ 1.83–1.92 (m, 2H, C*H*_2_); 2.81 (t, 2H, C*H*_2_, *J* = 5.7 Hz) 2.90 (dd, 1H, C*H*_*2a*_, *J*′ = 18.3, *J*″ = 20.0 Hz); 3.50 (dd, 1H, C*H*_*2b*_, *J*′ = 7.4, *J*″= 20.0 Hz); 3.64 (t, 2H, *J* = 8.9 Hz, C*H*_2_); 4.54 (dd, 1H, C*H, J*′ = 7.6, *J*″ = 14.7 Hz); 6.34 (s, 1H, C*H*); 7.05–7.17 (m, 4H, aryl); 7.29 (d, 1H, aryl, *J* = 10.0 Hz); 7.38–7.42 (m, 2H, aryl); 7.55 (d, 1H, aryl, *J* = 10.3 Hz). ^13^C NMR (100 MHz, CD_3_OD) δ 22.7, 30.7, 35.5, 38.1, 51.4, 53.2, 106.6, 110.9, 115.1, 115.3, 117.7, 119.0, 121.9, 126.0, 129.9, 130.3, 135.9, 137.2, 155.1, 161.5, 163.9, 173.6. HR-MS *m*/*z* calcd for C_22_H_21_FN_4_O_2_ [(M + H)]^+^: 393.1721; found 393.1733.

### (5*S*,11a*R*)-2-(3-Aminopropyl)-5-(4-fluorophenyl)-5,6,11,11a-tetrahydro-1*H*-imidazo[1′,5′:1,6]pyrido[3,4-*b*]indole-1,3(2*H*)-dione (**35a′**)

Synthesized according to the general procedure C starting from intermediate **34′**. FC in dichloromethane/methanol 9/1, *R_f_* = 0.47. White powder (38% yield). [α]^25^_D_: +107.83 ± 0.21 (*c* = 0.10, MeOH). ^1^H NMR (400 MHz, CD_3_OD) δ 1.71–1.80 (m, 2H, C*H*_2_); 2.61 (t, 2H, C*H*_2_, *J* = 9.0 Hz,) 2.81 (dd, 1H, C*H*_*2a*_, *J*′ = 14.9, *J*″ = 19.8 Hz,); 3.43 (dd, 1H, C*H*_*2b*_, *J*′ = 7.4, *J*″ = 20.1 Hz); 3.60 (t, 2H, C*H*_2_, *J* = 8.9 Hz,) 4.46 (dd, 1H, C*H, J*′ = 7.5, *J*″ = 14.7 Hz); 6.30 (s, 1H, C*H*); 7.03–7.15 (m, 4H, aryl); 7.28 (d, 1H, aryl, *J* = 10.7 Hz); 7.34–7.39 (m, 2H, aryl); 7.52 (d, 1H, aryl, *J* = 10.1 Hz). ^13^C NMR (100 MHz, CD_3_OD) δ: 22.7, 30.7, 35.5, 38.0, 51.4, 53.2, 106.6, 110.9, 115.1, 115.3, 117.7, 119.0, 121.9, 126.0, 129.9, 130.3, 135.9, 137.2, 155.1, 161.5, 163.9, 173.6. HR-MS *m*/*z* calcd for C_22_H_21_FN_4_O_2_ [(M + H)]^+^: 393.1721; found 393.1729.

### (5*R*,11a*S*)-5-(4-Fluorophenyl)-2-(3-(trifluoromethyl)phenyl)-5,6,11,11a-tetrahydro-1*H*-imidazo[1′,5′:1,6]pyrido[3,4-*b*]indole-1,3(2*H*)-dione (**36a**)

Synthesized form **32a** and 3-trifluoromethylphenyl isocyanate following the general procedure E. FC in dichloromethane/*n*-hexane 8/2, *R_f_* = 0.40. Yellowish powder (39% yield). [α]^25^_D_: −159.00 ± 10.00 (*c* = 0.10, MeOH). ^1^H NMR (CDCl_3_, 400 MHz): δ: 3.01 (dd, 1H, C*H*_*2a*_, *J*′ = 11.1, *J*″ = 15.4 Hz); 3.54 (dd, 1H, C*H*_2b_, *J*′ = 5.5, *J*″ = 15.4 Hz); 4.41 (dd, 1H, C*H, J*′ = 5.5, *J*″ = 11.0 Hz); 6.32 (s, 1H, C*H*); 7.14 (t, 1H, aryl, *J* = 6.9 Hz); 7.18 (t, 1H, aryl, *J* = 6.8 Hz); 7.24–7.30 (m, 5H, aryl); 7.49–7.56 (m, 3H, aryl); 7.61 (d, 1H, aryl, *J* = 7.6 Hz); 7.71 (s, 1H, aryl); 7.78 (s, 1H, N*H*). ^13^C NMR (CDCl_3_, 100 MHz) δ: 23.6, 51.7, 53.1, 108.4, 111.3, 118.6, 120.5, 122.9, 123.3, 124.8, 126.0, 129.0, 129.5, 129.6, 129.8, 131.4, 131.8, 132.1, 135.3, 136.7, 137.1, 153.2, 170.9. ^19^F NMR (CDCl_3_, 376.3 MHz) δ: −(62.59) (s, 3F, C*F*_3_); −(111.96) (s, 1F, C*F*). HR-MS *m*/*z*: calcd. for C_26_H_17_F_4_N_3_O_2_, [(M + H)^+^]: 480.1330; found 480.1338.

### (5*S*,11a*R*)-5-(4-Fluorophenyl)-2-(3-(trifluoromethyl)phenyl)-5,6,11,11a-tetrahydro-1*H*-imidazo[1′,5′:1,6]pyrido[3,4-*b*]indole-1,3(2*H*)-dione (**36a′**)

Synthesized form **32b** and 3-trifluoromethylphenyl isocyanate following the general procedure F. FC in dichloromethane/*n*-hexane 8/2, *R_f_* = 0.40. Yellowish powder (40% yield). [α]^25^_D_: +172.00 ± 10.00 (*c* = 0.10, MeOH) ^1^H NMR (CDCl_3_, 400 MHz): δ: 3.02 (dd, 1H, C*H*_*2a*_, *J*′ = 11.3, *J*″ = 13.7 Hz); 3.56 (dd, 1H, C*H*_2b_, *J*′ = 5.4, *J*″ = 15.3 Hz); 4.43 (dd, 1H, C*H, J*′ = 5.4, *J*″ = 10.8 Hz); 6.35 (s, 1H, C*H*); 7.01 (t, 2H, aryl, *J* = 8.5 Hz); 7.14–7.33 (m, 5H, aryl); 7.51–7.56 (m, 3H, aryl); 7.63 (d, 1H, aryl, *J* = 7.3 Hz); 7.74 (s, 1H, aryl); 7.80 (s, 1H, N*H*). ^13^C NMR (CDCl_3_, 100 MHz) δ: 23.6, 51.7, 53.0, 108.3, 111.3, 116.15, 116.36, 118.6, 120.5, 122.82, 122.86, 123.3, 124.8, 126.1, 129.0, 129.6, 129.8, 130.22, 130.31, 132.1, 134.5, 136.7, 153.2, 161.8, 164.3, 170.9. ^19^F NMR (CDCl_3_, 376.3 MHz) δ: −(62.67) (s, 3F, C*F*_3_); −(111.84) (s, 1F, C*F*). HR-MS *m*/*z*: calcd. for C_26_H_17_F_4_N_3_O_2_, [(M + H)^+^]: 480.1330; found 480.1335.

### (5*S*,11a*S*)-5-(4-Fluorophenyl)-2-(3-(trifluoromethyl)phenyl)-5,6,11,11a-tetrahydro-1*H*-imidazo[1′,5′:1,6]pyrido[3,4-*b*]indole-1,3(2*H*)-dione (**36b**)

Synthesized form **32b** and 3-trifluoromethylphenyl isocyanate following the general procedure E. FC in dichloromethane/*n*-hexane 8/2, *R_f_* = 0.35. Yellowish powder (42% yield). [α]^25^_D_: −1.00 ± 0.01 (*c* = 0.10, MeOH). ^1^H NMR (CDCl_3_, 400 MHz): δ: 3.15 (t, 1H, C*H*_*2a*_, *J* = 13.5 Hz); 3.56 (dd, 1H, C*H*_2b_, *J*′ = 4.4, *J*″ = 15.1 Hz); 4.54 (dd, 1H, C*H, J*′ = 4.5, *J*″ = 13.3 Hz); 5.86 (s, 1H, C*H*); 6.97 (t, 2H, aryl, *J* = 6.9 Hz); 7.11–7.20 (m, 3H, aryl); 7.26 (t, 2H, aryl, *J* = 6.8 Hz); 7.45–7.58 (m, 5H, aryl); 7.67 (s, 1H, N*H*). ^13^C NMR (CDCl_3_, 100 MHz) δ: 23.6, 51.7, 53.0, 108.3, 111.3, 116.1, 116.4, 118.6, 120.5, 122.8, 122.9, 123.3, 124.8, 126.1, 129.0, 129.6, 129.8, 130.2, 130.3, 132.1, 134.5, 136.7, 153.2, 161.8, 164.3, 170.9. ^19^F NMR (CDCl_3_, 376.3 MHz) δ: −(62.44) (s, 3F, C*F*_3_); −(112.08) (s, 1F, C*F*). HR-MS *m*/*z*: calcd for C_26_H_17_F_4_N_3_O_2_, [(M + H)^+^]: 480.1330; found 480.1341.

### (5*R*,11a*S*)-2-(2-Fluorophenyl)-5-(4-fluorophenyl)-5,6,11,11a-tetrahydro-1*H*-imidazo[1′,5′:1,6]pyrido[3,4-*b*]indole-1,3(2*H*)-dione (**37a**)

Synthesized from **32a** and 2-fluorophenyl isocyanate following the general procedure E. FC in dichloromethane/ethyl acetate 9.5/0.5, *R_f_* = 0.52. Yellowish powder (42% yield). [α]^25^_D_: −154.00 ± 2.53 (*c* = 0.10, MeOH). ^1^H NMR (CDCl_3_, 400 MHz): δ: 3.11 (t, 1H, C*H*_2a_, *J* = 12.7 Hz); 3.63 (dd, 1H, C*H*_2b_, *J*′ = 5.4, *J*″ = 15.4 Hz); 4.52 (dd, 1H, C*H, J*′ = 5.2, *J*″ = 10.7 Hz); 6.41 (s, 1H, C*H*); 7.08 (t, 2H, aryl, *J* = 8.4 Hz); 7.23–7.45 (m, 9H, aryl); 7.62 (d, 1H, aryl, *J* = 7.4 Hz); 7.96 (s, 1H, NH). ^13^C NMR (CDCl_3_, 100 MHz) δ: 23.7, 51.6, 53.4, 108.3, 111.3, 116.1, 116.3, 116.7, 118.5, 119.1, 120.4, 123.2, 124.7, 126.1, 129.6, 129.9, 130.2, 131.0, 134.8, 136.7, 153.2, 156.5, 159.0, 161.8, 164.2, 170.9. ^19^F NMR (CDCl_3_, 376.3 MHz) δ: −(112.10) (s, 1F, C*F*). HR-MS *m*/*z*: calcd. for C_25_H_17_F_2_N_3_O_2_, [(M + H)^+^]: 430.1362; found 430.1370.

### (5*S*,11a*R*)-2-(2-Fluorophenyl)-5-(4-fluorophenyl)-5,6,11,11a-tetrahydro-1*H*-imidazo[1′,5′:1,6]pyrido[3,4-*b*]indole-1,3(2*H*)-dione (**37a′**)

Synthesized from **32b** and 2-fluorophenyl isocyanate following the general procedure F. FC in dichloromethane/ethyl acetate 9.5/0.5, *R_f_* = 0.52. Yellowish powder (39% yield). [α]^25^_D_: +166.00 ± 2.53 (*c* = 0.10, MeOH). ^1^H NMR (CDCl_3_, 400 MHz): δ: 3.13 (t, 1H, C*H*_2a_, *J* = 15.0 Hz); 3.64 (dd, 1H, C*H*_2b_, *J*′ = 5.4, *J*″ = 15.4 Hz); 4.53 (dd, 1H, C*H, J*′ = 5.4, *J*″ = 11.0 Hz); 6.44 (s, 1H, C*H*); 7.10 (t, 2H, aryl, *J* = 8.5 Hz); 7.22–7.48 (m, 9H, aryl); 7.63 (d, 1H, aryl, *J* = 7.7 Hz); 7.88 (s, 1H, NH). ^13^C NMR (CDCl_3_, 100 MHz) δ: 23.7, 51.6, 53.4, 108.4, 111.3, 116.1, 116.3, 116.7, 116.9, 118.6, 120.4, 123.2, 124.7, 126.1, 129.6, 129.9, 130.2, 130.3, 130.87, 130.95, 134.7, 136.7, 153.2, 156.5, 159.0, 161.8, 164.3, 170.8. ^19^F NMR (CDCl_3_, 376.3 MHz) δ: −(119.22) (s, 1F, C*F*); −(112.05) (s, 1F, C*F*). HR-MS *m*/*z*: calcd for C_25_H_17_F_2_N_3_O_2_, [(M + H)^+^]: 430.1362; found 430.1355.

### (5*S*,11a*S*)-2-(2-Fluorophenyl)-5-(4-fluorophenyl)-5,6,11,11a-tetrahydro-1*H*-imidazo[1′,5′:1,6]pyrido[3,4-*b*]indole-1,3(2*H*)-dione (**37b**)

Synthesized from **32b** and 2-fluorophenyl isocyanate following the general procedure E. FC in dichloromethane/ethyl acetate 9.5/0.5, *R_f_* = 0.48. Yellowish powder (40% yield). [α]^25^_D_: −14.00 ± 0.03 (*c* = 0.10, MeOH). ^1^H NMR (CDCl_3_, 400 MHz): δ: 3.15 (t, 1H, C*H*_2a_, *J* = 13.1 Hz); 3.54 (dd, 1H, C*H*_2b_, *J*′ = 4.2, *J*″ = 15.0 Hz); 4.55 (dd, 1H, C*H, J*′ = 3.8, *J*″ = 11.0 Hz); 5.83 (s, 1H, C*H*); 6.94 (t, 2H, aryl, *J* = 8.4 Hz); 7.11–7.32 (m, 8H, aryl); 7.53 (s, 2H, aryl). ^13^C NMR (CDCl_3_, 100 MHz) δ: 22.7, 56.4, 58.3, 107.3, 11.3, 115.8, 116.1, 116.6, 116.8, 118.6, 119.0, 120.4, 123.1, 124.5, 126.2, 129.8, 129.9, 130.8, 132.9, 134.0, 136.8, 153.1, 156.4, 158.9, 161.6, 164.0, 169.8. ^19^F NMR (CDCl_3_, 376.3 MHz) δ: −(119.00) (s, 1F, C*F*); −(112.68) (s, 1F, C*F*). HR-MS *m*/*z*: calcd. for C_25_H_17_F_2_N_3_O_2_, [(M + H)^+^]: 430.1362; found 430.1373.

### (5*R*,11a*S*)-2,5-Bis(4-fluorophenyl)-5,6,11,11a-tetrahydro-1*H*-imidazo[1′,5′:1,6]pyrido[3,4-*b*]indole-1,3(2*H*)-dione (**38a**)

Synthesized from **32a** and 4-fluorophenyl isocyanate following the general procedure E. FC in dichloromethane/*n*-hexane 8/2, *R_f_* = 0.40. Yellowish powder (42% yield). [α]^25^_D_: −164.00 ± 10.00 (*c* = 0.10, MeOH). ^1^H NMR (CDCl_3_, 400 MHz): δ: 2.99 (dd, 1H, C*H*_*2a*_, *J*′ = 13.2, *J*″ = 16.9 Hz); 3.52 (dd, 1H, C*H*_2b_, *J*′ = 5.5, *J*″ = 15.3 Hz); 4.39 (dd, 1H, C*H, J*′ = 5.5, *J*″ = 11.0 Hz); 6.33 (s, 1H, C*H*); 6.99 (t, 2H, aryl, *J* = 8.6 Hz); 7.07 (t, 2H, aryl, *J* = 8.7 Hz); 7.14–7.21 (m, 3H, aryl); 7.24–7.36 (m, 4H, aryl); 7.53 (d, 1H, aryl, *J* = 7.6 Hz); 7.76 (s, 1H, N*H*). ^13^C NMR (CDCl_3_, 100 MHz) δ: 23.6, 51.6, 53.0, 108.4, 111.3, 115.9, 116.3, 118.6, 120.4, 123.2, 126.1, 127.4, 127.9, 129.9, 130.9, 134.7, 136.7, 153.7, 160.7, 161.8, 163.2, 164.3, 171.3. ^19^F NMR (CDCl_3_, 376.3 MHz) δ: −(111.98) (s, 1F, C*F*); −(112.80) (s, 1F, C*F*). HR-MS *m*/*z*: calcd for C_25_H_17_F_2_N_3_O_2_, [(M + H)^+^]: 430.1362; found 430.1371.

### (5*S*,11a*R*)-2,5-Bis(4-fluorophenyl)-5,6,11,11a-tetrahydro-1*H*-imidazo[1′,5′:1,6]pyrido[3,4-*b*]indole-1,3(2*H*)-dione (**38a′**)

Synthesized from **32b** and 4-fluorophenyl isocyanate following the general procedure F. FC in dichloromethane/*n*-hexane 8/2, *R_f_* = 0.40. Yellowish powder (45% yield). [α]^25^_D_: +185.00 ± 10.00 (*c* = 0.10, MeOH). ^1^H NMR (CDCl_3_, 400 MHz): δ: 2.99 (dd, 1H, C*H*_*2a*_, *J*′ = 11.5, *J*″ = 14.6 Hz); 3.53 (dd, 1H, C*H*_2b_, *J*′ = 5.5, *J*″ = 15.4 Hz); 4.39 (dd, 1H, C*H, J*′ = 5.5, *J*″ = 11.0 Hz); 6.33 (s, 1H, C*H*); 6.99 (t, 2H, aryl, *J* = 8.5 Hz); 7.07 (t, 2H, aryl, *J* = 8.6 Hz); 7.12–7.19 (m, 3H, aryl); 7.24–7.36 (m, 4H, aryl); 7.53 (d, 1H, aryl, *J* = 7.8 Hz); 7.75 (s, 1H, N*H*). ^13^C NMR (CDCl_3_, 100 MHz) δ: 23.6, 51.6, 53.0, 108.4, 111.3, 115.9, 116.2, 118.6, 120.4, 123.3, 126.1, 127.4, 127.8, 129.9, 130.3, 134.7, 136.7, 153.7, 160.7, 161.8, 163.2, 164.3, 171.3. ^19^F NMR (CDCl_3_, 376.3 MHz) δ: −(111.97) (s, 1F, C*F*); −(112.80) (s, 1F, C*F*). HR-MS *m*/*z*: calcd. for C_25_H_17_F_2_N_3_O_2_, [(M + H)^+^]: 430.1362; found 430.1357.

### (5*S*,11a*S*)-2,5-Bis(4-fluorophenyl)-5,6,11,11a-tetrahydro-1*H*-imidazo[1′,5′:1,6]pyrido[3,4-*b*]indole-1,3(2*H*)-dione (**38b**)

Synthesized form **32b** and 4-fluorophenyl isocyanate following the general procedure E. FC in dichloromethane/*n*-hexane 8/2, *R_f_* = 0.45. Yellowish powder (48% yield). [α]^25^_D_: −8.03 ± 0.02 (*c* = 0.10, MeOH). ^1^H NMR (CDCl_3_, 400 MHz): δ: 3.22 (t, 1H, C*H*_*2a*_, *J* = 11.7 Hz); 3.64 (dd, 1H, C*H*_2b_, *J*′ = 3.2, *J*″ = 15.0 Hz); 4.59 (dd, 1H, C*H, J*′ = 4.4, *J*″ = 11.3 Hz); 5.92 (s, 1H, C*H*); 7.05 (t, 2H, aryl, *J* = 8.5 Hz); 7.12 (t, 2H, aryl, *J* = 8.6 Hz); 7.21–7.43 (m, 6H, aryl); 7.62–7.65 (m, 2H, aryl). ^13^C NMR (CDCl_3_, 100 MHz) δ: 22.7, 56.4, 57.9, 107.3, 111.3, 115.8, 116.1, 118.6, 120.4, 123.1, 126.2, 127.3, 127.8, 129.9, 132.9, 134.1, 136.8, 153.7, 160.0, 161.6, 163.1, 164.0, 170.2. ^19^F NMR (CDCl_3_, 376.3 MHz) δ: −(112.80) (s, 1F, C*F*); −(111.98) (s, 1F, C*F*). HR-MS *m*/*z*: calcd. for C_25_H_17_F_2_N_3_O_2_, [(M + H)^+^]: 430.1362; found 430.1370.

### In Vitro Biological Assays. Cell Cultures

For measurement of the potency of the compounds, fluorimetric experiments were performed using HEK-293 cells (CRL-1573TM, American Type Culture Collection, LGC Promochem, Molsheim, France) that stably express rat TRPM8. The cells were seeded in 96-well plates (Corning Incorporated, Corning, NY) at a cell density of 40 000 cells 2 days before treatment. On the day of treatment, the medium was replaced with 100 μL of the dye loading solution Fluo-4 NW supplemented with probenecid 2.5 mM.

For the assessment of selectivity of target compounds, fluorimetric experiments were performed using HEK-293 cells lines stably transfected with either hTRPA1 or hNav1.7 and CHO-K1 stably transfected with hTRPV1. HEK-293 cells were cultured in EMEM (MEM Eagle Earl’s salts balanced salt solution, Lonza, Walkersville, USA), 5 mL of 200 mM Ultraglutamine1 (Lonza), 5 mL of 100× penicillin/streptomycin (Lonza), 50 mL of fetal bovine serum (Euroclone, Milan, Italy), 2 mL of 100 mg/mL G418 (InvivoGen, San Diego, USA). CHO-K1 cells were grown in DMEM F-12 (1:1) mixture (Lonza), 5 mL of 100 mM sodium pyruvate (Lonza), 25 mL of 7.5% sodium bicarbonate (Lonza), 6.5 mL of 1 M HEPES (Lonza), 5 mL of 100× penicillin/streptomycin (Lonza), 50 mL of fetal bovine serum (Euroclone), 0.25 mL of 10 mg/mL puromycin (InvivoGen), and 0.5 mL of 100 mg/mL zeocin (InvivoGen).

For patch-clamp experiments, HEK-293/TRPM8 exon1 K3 cells were cultured in minimum essential medium with Earle’s salts, without l-glutamine (Euroclone) supplemented with 5 mL of 200 mM Ultraglutamine 1 in 0.85% NaCl solution (Lonza), 5 mL of 100× penicillin/streptomycin (Lonza), 0.2 mL of 10 mg/mL puromycin (InvivoGen; final concentration 0.4 μg/mL), and 50 mL of fetal bovine serum (Sigma-Aldrich, Milan, Italy).

### Fluorimetric Assays

The tested molecules dissolved in DMSO were added at the desired concentrations, and the plates were incubated in darkness at 37 °C in a humidified atmosphere of 5% CO_2_ for 60 min. The fluorescence was measured using instrument settings appropriate for excitation at 485 nm and emission at 535 nm (POLARstar Omega BMG LABtech). A baseline recording of four cycles was recorded prior to stimulation with the agonist (100 μM menthol for TRPM8). The TRPM8 antagonist, 10 μM AMTB, was added to the medium containing the corresponding agonist to induce channel blockade. The changes in fluorescence intensity were recorded during 15 cycles more. The higher concentration of DMSO used in the experiment was added to the control wells. The cells’ fluorescence was measured before and after the addition of various concentrations of test compounds. The fluorescence values obtained are normalized to that prompted by the corresponding agonist (for channel activating compounds) or upon agonist and antagonist coexposure (for channel blocker compounds).

### Selectivity Assays

The analysis was performed in 384-well clear bottom black walled polystyrene plates, (Thermo Scientific, Waltham, USA) for CHO-K1 cells and in 384-well clear bottom black polystyrene walled poly-d-Lys coated plates (TwinHelix, Rho, Italy) for HEK-293 cells. Compound dilution was performed in 96-well U bottom plates (Thermo Scientific), and then compounds were transferred into 384-well V bottom polypropylene barcoded plates (Thermo Scientific). To assess the activity of the selected compound over TRPA1 and TRPV1, cells were seeded in 384 MTP in complete medium (25 μL/well) at 10 000 cells/well concentration. 24 h after seeding, the culture medium was removed and cells were loaded with 20 μL/well of 0.5× calcium sensitive dye (Fluo-8 NW, AAT Bioquest, Sunnyvale, USA) in assay buffer. To assess the activity of the selected compound over Nav1.7, cells were seeded at 15 000 cells/well in 384 MTP in complete medium (25 μL/well). 24 h after seeding, the culture medium was removed and cells were loaded with 20 μL/well of 0.5× membrane potential dye (FLIPR membrane potential assay kits Blue, Molecular Devices LLC, San Jose, USA) in assay buffer. Plates were incubated for 1 h at room temperature in the dark. Then, 10 μL/well of test compounds and controls were injected at 3× concentration, and the signal of the emitted fluorescence was recorded using FLIPRTETRA apparatus (FortèBio, Fremont, USA). Then, a second injection of 15 μL/well of 3× reference activator (at ∼EC_80_) was performed analyzing the signal of the emitted fluorescence. Allyl isothiocyanate (AITC, Sigma-Aldrich), capsaicine (Sigma-Aldrich), and veratridine (Sigma-Aldrich) were used as reference agonists, while HC-030031 (Sigma-Aldrich), capasazepine (Sigma-Aldrich), and tetrodotoxine (Tocris bioscience, Bristol, U.K.) were used as reference antagonists for TRPA1, TRPV1, and Nav1.7 assaying, respectively.

### Patch-Clamp Experiments

HEK-293/TRPM8exon 1 cells are seeded 72 or 96 h before experiment at a concentration of 4 and 2.5 million cells, respectively, onto a T225 flask. Just before the experiments, cells are washed twice with D-PBS without Ca^2+^/Mg^2+^ (Euroclone, Milan, Italy) and detached from the flask with trypsin–EDTA (Sigma-Aldrich, Milan, Italy; diluted 1/10). Cells are then resuspended in the suspension solution, 25 mL of EX-CELL ACF CHO medium (Sigma-Aldrich, Milan, Italy); 0.625 mL of HEPES (Lonza, Walkersville, USA); 0.25 mL of 100× penicillin/streptomycin (Lonza, Walkersville, USA), 0.1 mL of soybean trypsin inhibitor 10 mg/mL (Sigma-Aldrich, Milan, Italy), and placed on an automated patch-clamp platform (QPatch 16X, Sophion Bioscience, Ballerup, Denmark).

Menthol was used as reference agonist, and a stock solution (1 M, 100% DMSO) was prepared the day of the experiment from the powder; an intermediate stock of 300 mM was prepared from the 1 M stock in 100% DMSO, and the final dilution was performed in the extracellular solution to obtain a working concentration of 300 μM (1:1000, 0.1% final DMSO concentration). Stock solutions of the testing compounds (10 mM; 100% DMSO; stored at −20 °C) were prepared the day of the experiment; an intermediate stock for each compound (300 μM) was prepared from the 10 mM stock in 100% DMSO, and the working dilutions were performed just before the experiments in the extracellular solution containing 300 μM menthol. The highest concentration tested was 300 nM, with serial dilutions (1:10) in the extracellular solution. DMSO was balanced to keep it constant throughout all the solutions in the same experiment (0.2% final DMSO concentration). Standard whole-cell voltage clamp experiments are performed at room temperature using the multihole technology. For the voltage clamp experiments on human TRPM8, data are sampled at 2 kHz. After establishment of the seal and the passage in the whole cell configuration, the cells are challenged by a voltage ramp (20 ms step at −60 mV; 100 ms ramp −60/+100 mV; 20 ms step at +100 mV; return to −60 mV) every 4 s. The potential antagonistic effect on human TRPM8 current of target compounds was evaluated after application of the agonist (menthol, 300 μM) alone and in the presence of the compound under investigation at increasing concentrations. Output: outward current evoked by the voltage ramp, measured in the step at +100 mV. The intracellular solution contained (mM) 135 CsCl, 10 BAPTA, 10 HEPES, 4 Na_2_ATP (pH 7.2 with CsOH). The extracellular solution contained (mM) 145 NaCl, 4 KCl, 1 MgCl_2_, 2 CaCl_2_, 10 HEPES, 10 glucose (pH 7.4 with NaOH).

### Computational Details

3D structures of TRPM8 in complex with TC-I 2014 antagonist (PDB code: 6O72)^28c^ were prepared using the Schrödinger Protein Preparation Wizard workflow.^40^ Specifically, water molecules were deleted, cap termini were included, all hydrogen atoms were added, and bond orders were assigned. Finally, the .pdb files were converted to the .mae file.

The grids for the subsequent molecular docking calculations were generated accounting the related position of TC-I 2014 on the receptor binding sites. In this way, the cocrystallized ligands were also automatically removed from the original binding sites.

The library of investigated compounds (see Results and Discussion) was prepared using LigPrep software (Schrodinger Suite).^41^ Specifically, all the possible tautomers and protonation states at pH = 7.4 ± 1.0 were generated for each compound, and finally the structures were minimized using the OPLS 2005 force field.

Molecular docking experiments were performed using Glide software (Schrödinger Suite),^42^ setting the Extra Precision [XP] mode. For this step, 20 000 poses were kept in the starting phase of docking, and 1200 poses for energy minimization were selected. The scoring window for keeping the initial poses was set to 400.0, and a scaling factor of 0.8 related to van der Waals radii with a partial charge cutoff of 0.15, based on a 0.5 kcal/mol rejection cutoff for the obtained minimized poses, was considered. In the output file, 10 poses for each compound were saved.

### In Vitro Metabolic Stability Using Liver Microsomes

#### Protocol I

Each sample (2.5 mM) was incubated with 100 mM phosphate buffer (pH 7.4) and 20 mg/mL of liver microsomes (Thermo Fisher Scientific, Bremen, Germany). After preincubation in water bath for 5 min, the mixture was incubated with 20 mM NADPH (protocol I) at 37 °C for 60 min in a Thermomixer comfort (Eppendorf, Hamburg, Germany).

#### Protocol II

For the measurement of UGT activity the microsomes were preincubated with alamethicin, which forms pores in microsomal membranes, promoting access of substrate and cofactor to UGT enzymes. Subsequently, each sample was incubated with 100 mM phosphate buffer, 500 mM magnesium chloride, 10 mM NADPH, and 20 mM UDP-GlcUA at 37 °C for 60 min.

Finally, the reactions from both protocols (protocols I and II) were stopped by the addition of 200 μL of ice-cold methanol, and then samples were centrifuged at 10 000 rpm at 25 °C for 5 min (Eppendorf microcentrifuge 5424, Hamburg, Germany). The supernatants were collected and injected in UHPLC-PDA.

The control at 0 min was obtained by addition of the organic solvent immediately after incubation with microsomes. As the positive control, testosterone was used, while the negative controls were prepared by incubation up to 60 min without NADPH and UDP-GlcUA/NADPH for protocols I and II, respectively. The negative control is essential to detect problems such as nonspecific protein binding or heat instability. The extent of metabolism is expressed as a percentage of the parent compound turnover using the following equation, as previously described:^43^
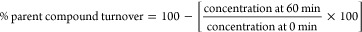


### Animals

C57-mice (males, 5 week old, ∼30 g) (Harlan, The Netherlands) were used for the oxaliplatin-induced neuropathic pain study. All experiments were approved by the Institutional Animal and Ethical Committee of the Universidad Miguel Hernandez where experiments were conducted, and they were in accordance with the guidelines of the Economic European Community and the Committee for Research and Ethical Issues of the International Association for the Study of Pain. All parts of the study concerning animal care were performed under the control of veterinarians.

The WDS was performed in Wistar male rats (300–350 g), and the thermal ring experiment was performed on male Swiss CD1 mice (30–35 g) purchased from Charles Rivers (Calco-Lecco-Italy) and then housed in the animal care facility of the Department Experimental of Pharmacology, University of Naples. The animals were acclimated to their environment for 1 week, and food and water were available ad libitum. All behavioral tests were performed between 9:00 am and 1:00 pm, and animals were used only once. Procedures involving animals and their care were conducted in conformity with international and national law and policies (EU Directive 2010/63/EU for animal experiments, ARRIVE guidelines, and the Basel declaration including the 3R concept). All procedures reported here were approved by the Institutional Committee on the Ethics of Animal Experiments (CVS) of the University of Naples Federico II and by “Ministero della Salute” under Protocol No. 851/2016. All efforts were made to minimize animal suffering, and at the end of all experiments, the animals were euthanized by CO_2_ overdose.

### Drug Treatment

For the oxaliplatin-induced neuropathic pain assay, oxaliplatin (Tocris) was dissolved in water with gentle warming and was subcutaneously (sc) injected on days 1, 3, and 5 at a 6 mg/kg dose. The day 7 after administration, experiments were performed. Together with oxaliplatin injection, saline and a 5% mannitol solution were intraperitoneally injected to prevent kidney damage and dehydration. **31a** stock was prepared in DMSO (Sigma-Aldrich) and diluted in saline for injections. Compound **31a** at different doses (1 to 30 μg) was injected into the plantar surface (25 μL) of the right hind paw of mice.

For the other in vivo assays, compound **4** and **31a** were dissolved in PEG 400 10% v/v, Tween 80 5% v/v, and sterile saline 85% v/v and injected once intraperitoneally at the equimolar doses of 10 mg/kg for **4** and 6.7 mg/kg for **31a**. Control group was only treated with vehicle.

### Icilin-Induced “Wet-Dog” Shaking in Rats

Icilin, a TRPM8 agonist, was used to induce shaking in mice.^44^ Animals were first habituated to the testing room for 30 min. After that they were randomized into treatment groups and treated with vehicle or TRPM8 antagonists. Icilin was administered intraperitoneally (ip) at 1 mg/kg dissolved in 1% Tween 80/H_2_O 30 or 120 min after drugs. The number of intermittent but rhythmic “wet-dog-like” shakes (WDS) of neck, head, and trunk in each animal was counted for a period of 30 min following icilin administration.

### Oxaliplatin-Induced Neuropatic Pain Model

Cold chemical thermal sensitivity was assessed using acetone drop method.^18b^ Mice were placed in a metal mesh cage and allowed to habituate for approximately 30 min in order to acclimatize them. Freshly dispensed acetone drop (10 μL) was applied gently onto the mid-plantar surface of the hind paw. Cold chemical sensitive reaction with respect to paw licking was recorded as a positive response (nociceptive pain response). The responses were measured for 20 s with a digital stopwatch. For each measurement, the paw was sampled twice and the mean was calculated. The interval between each application of acetone was approximately 5 min.

### Chronic Constriction Injury (CCI) Model of Neuropathic Pain

Neuropathic pain behavior was induced by ligation of the sciatic nerve as described previously.^27d^ Briefly, mice were first anesthetized with xylazine (10 mg/kg ip) and ketamine (100 mg/kg ip), and the left thigh was shaved and scrubbed with betadine, and then a small incision in the middle left thigh (2 cm in length) was performed to expose the sciatic nerve. The nerve was loosely ligated at two distinct sites (spaced at a 2 mm interval) around the entire diameter of the nerve using silk sutures (7–0). The surgical area was closed and finally scrubbed with betadine. In sham-operated animals, the nerve was exposed but not ligated. Drug effects were evaluated 7 and 14 days after ligation.

### Thermal Gradient Ring

We utilized the thermal gradient ring from Ugo-Basile previously using a modified protocol from Touska et al., 2016.^35^ The apparatus consists of a circular running track where each side of the ring is divided into 12 zones, in which the temperature is proportionally distributed from 15 to 40 °C, and each sector represents an increment of 2.27 °C. Before the experiment, on day 1, all mice were habituated to the apparatus for 30 min with the aluminum floor acclimatized to room temperature (22–24 °C). On day 2, mice were injected and 30 min after were placed in the apparatus and measured for 60 min using 15–40 °C. Data on preference temperature in time course were collected from the video-tracking software Any-Maze connected to the apparatus.

### Data Analysis

Data are reported as the mean ± standard error of the mean (sem) values of at least three independent experiments each in triplicate. Statistical analysis was performed by analysis of variance test, and multiple comparisons were made by Bonferroni’s test by using Prism 5 (GraphPad Software, San Diego, CA, USA). *p*-values smaller than 0.05 were considered significant.

## Supporting Information Available

Figure S1 showing time-course trans (**36a′**–**38a′**)/cis (**36b**,**38b**) HPLC peak area ratio in physiological buffer and methanol; Figure S2 showing effects of selected compounds over NaV_1.7_, TRPA1, and TRPV1 channels; Figures S3–S88 showing NMR spectra of synthesized compounds; Figure S89 showing attribution of the absolute configuration for derivatives **30a** and **30a′**; Figures S90–S97 showing HPLC chromatograms of derivatives **6a**, **9**, **11a**, **12a**, **23**, **31a**, **31a′**, and **36b**; Table S1 listing regression curves and *R*^2^ for quantitative UHPLC determination of selected compounds (PDF)
Atomic coordinates for PDB code 6O72 (PDB)
Atomic coordinates for complex **4**-6O72 (PDB)
Atomic coordinates for complex **6a**-6O72 (PDB)
Atomic coordinates for complex **9**-6O72 (PDB)
Atomic coordinates for complex **11a**-6O72 (PDB)
Atomic coordinates for complex **11b**-6O72 (PDB)
Atomic coordinates for complex **12a**-6O72 (PDB)
Atomic coordinates for complex **12b**-6O72 (PDB)
Atomic coordinates for complex **23**-6O72 (PDB)
Atomic coordinates for complex **31a**-6O72 (PDB)
Atomic coordinates for complex **31a′**-6O72 (PDB)
Atomic coordinates for complex **36a**-6O72 (PDB)
Atomic coordinates for complex **36a′**-6O72 (PDB)
Atomic coordinates for complex **36b**-6O72 (PDB)
Molecular formula strings and some data (XLSX)

## Supplementary Material

jm0c00816_si_001.pdf

jm0c00816_si_002.pdb

jm0c00816_si_003.pdb

jm0c00816_si_004.pdb

jm0c00816_si_005.pdb

jm0c00816_si_006.pdb

jm0c00816_si_007.pdb

jm0c00816_si_008.pdb

jm0c00816_si_009.pdb

jm0c00816_si_010.pdb

jm0c00816_si_011.pdb

jm0c00816_si_012.pdb

jm0c00816_si_013.pdb

jm0c00816_si_014.pdb

jm0c00816_si_015.pdb

## Abbreviations Used

WDS: wet-dog shake
*L*-Trp-OMe: l-tryptophan methyl ester
NBoc-*L*-Trp-OH: *N*-Boc-l-tryptophan
HoBT: hydroxybenzotriazole
HBTU: (2-(1*H*-benzotriazol-1-yl)-1,1,3,3-tetramethyluronium hexafluorophosphate
DIPEA: diisopropylethylamine
DMF: dimethylformamide
NHBoc-β-Ala-OH: *N*-Boc-β-alanine
NHBoc-Gly-OH: *N*-Boc-glycine
NHBoc-*L*-Phe-OH: *N*-Boc-l-phenylalanine
NHBoc-*D*-Phe-OH: *N*-Boc-d-phenylalanine
TEA: triethylamine
TIS: triisopropylsilane
UDP-GlcUA: UDP-glucuronic acid dehydrogenase
Veh: vehicle
CCI: chronic constriction injury
