# Supplementary material for: Exploration of TRPM8 Binding Sites by β-Carboline-Based Antagonists and Their In Vitro Characterization and In Vivo Analgesic Activities
Source: J Med Chem. 2020 Jul 29;63(17):9672–94. doi: 10.1021/acs.jmedchem.0c00816 (PMC8009520; doi:10.1021/acs.jmedchem.0c00816)
Supplement: Supplementary file 1 — jm0c00816_si_001.pdf [file jm0c00816_si_001.pdf]

Supporting Informations

Exploration of TRPM8 Binding Sites by β–Carboline-based Antagonists: In Vitro Characterization and in Vivo Analgesic Activity

Alessia Bertamino,[†,#] Carmine Ostacolo,[‡,#] Veronica Di Sarno,[†] Gianluigi Lauro,[†] Tania Ciaglia,[†] Vincenzo Vestuto,[†] Giacomo Pepe,[†] Manuela Giovanna Basilicata,[†] Simona Musella,[§] Gerardina Smaldone,[†] Claudia Cristiano,[‡] Sara Gonzalez-Rodriguez,[‖] Alicia Medina,[‖] Asia Fernandez-Carvajal,[‖] Giuseppe Bifulco,[†] Pietro Campiglia,[*,†,§] Isabel Gomez-Monterrey,[*,‡] and Roberto Russo.[‡]

[†]Department of Pharmacy, University of Salerno, Via G. Paolo II 132, 84084, Fisciano, Salerno, Italy

[‡]Department of Pharmacy, University Federico II of Naples, Via D. Montesano 49, 80131, Naples, Italy

[§]European Biomedical Research Institute (EBRIS), Via S.De Renzi 50, 84125, Salerno, Italy

[‖]Institute of Molecular and Cellular Biology, Universitas Miguel Hernández, Avda de la Universidad , 032020, Elche, Spain

# These authors contributed equally to this work

Table of content:

Page S3 – Figure S1: Time-course trans (36a'-38a') / cis (36b-38b) HPLC peak area ratio in physiological buffer (PBS, panel A) and methanol (Panel B)

Page S4 - Figure S2: Effects of selected compounds over $NaV_{1.7}$, TRPA1 and TRPV1 channels

Page S5-S90 - NMR spectra of synthesized compounds

Page S91 - Figure S90: Attribution of the absolute configuration for derivatives **30a** and **30a'**

Page S92-S99 – HPLC chromatograms of derivatives **6a**, **9**, **11a**, **12a**, **23**, **31a**, **31a'** and **36b**

Page S100 - Table S1: Regression curves and R^2 for quantitative UHPLC determination of selected compounds

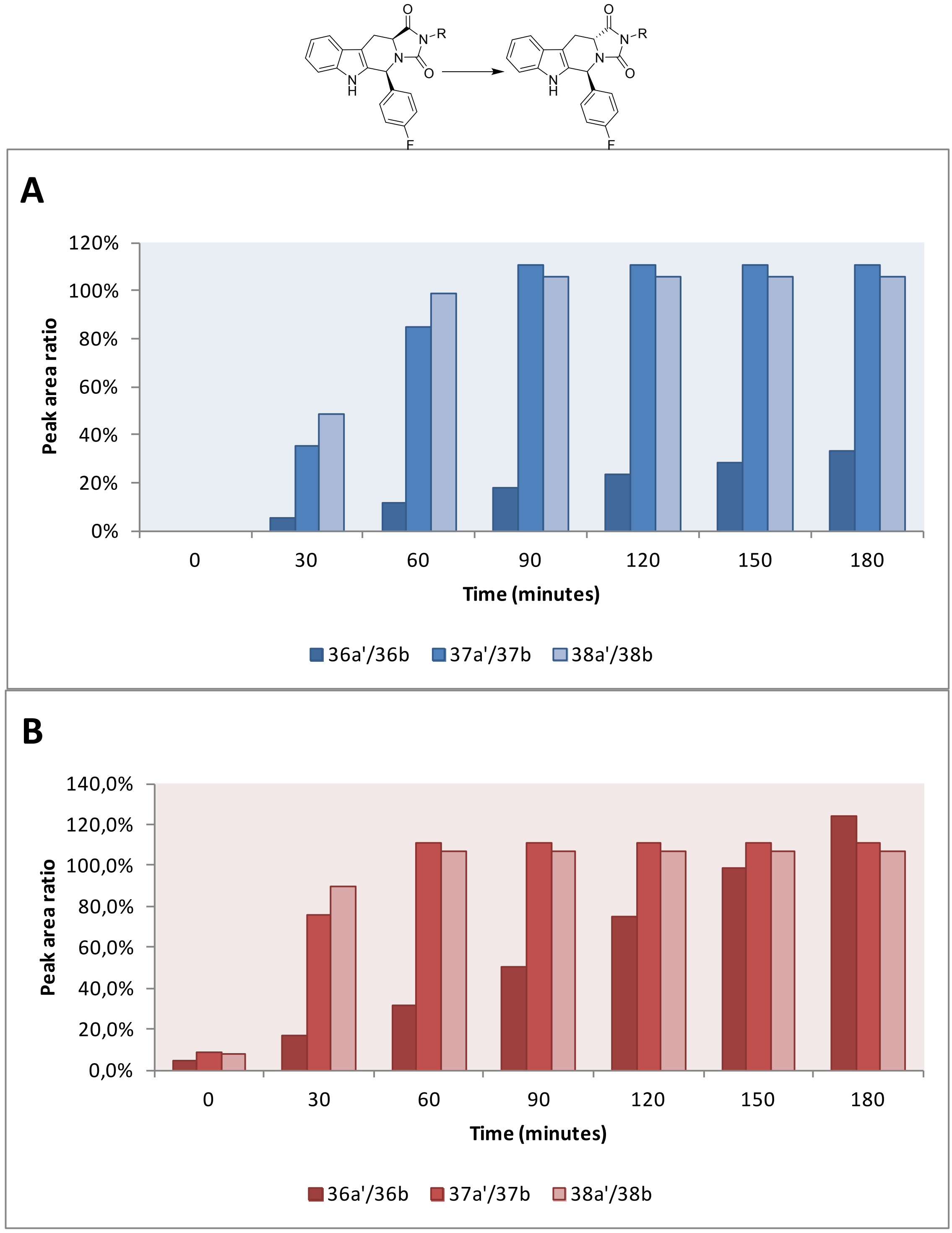

Figure S1: Time-course trans (**36a'-38a'**) / cis (**36b-38b**) HPLC peak area ratio in physiological buffer (PBS, panel A) and methanol (Panel B).

	Compound	Nav17	TRPA1	TRPV1	Compound	Nav17	TRPA1	TRPV1
Compound ID	Overlay_Agonist Mode	EC50	EC50	EC50	Overlay_Antagonist Mode	IC50	IC50	IC50
**6a**	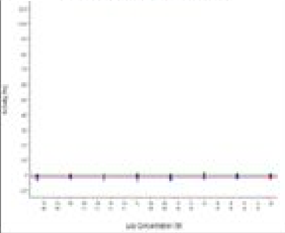				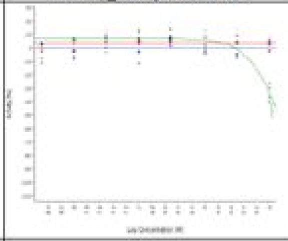	IC50>10μM		
**9**	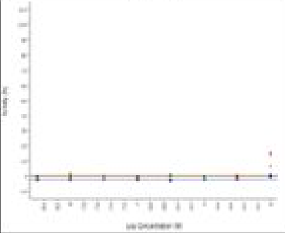				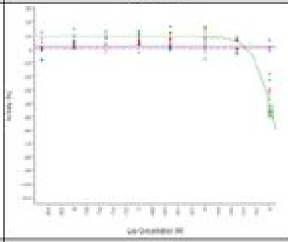	IC50>10μM		
**11a**	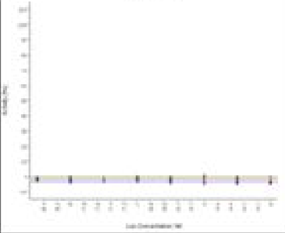				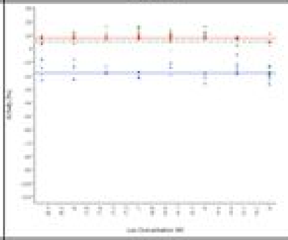			
**23**	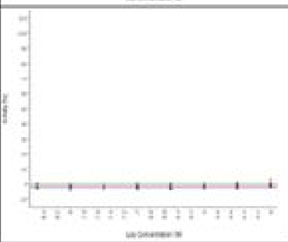				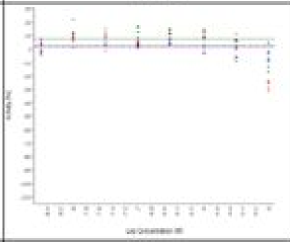			
**31a**	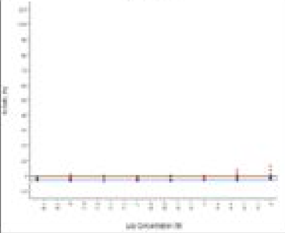				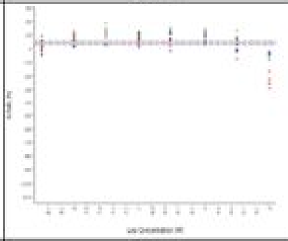			
**36b**	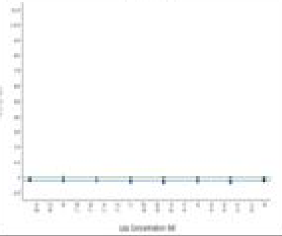				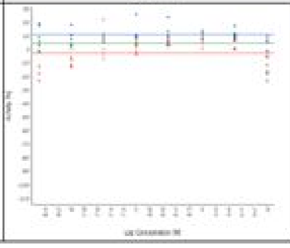			

■ Nav17
■ TRPA1
■ TRPV1

Figure S2: Effects of selected compounds over $NaV_{1.7}$, TRPA1 and TRPV1 channels

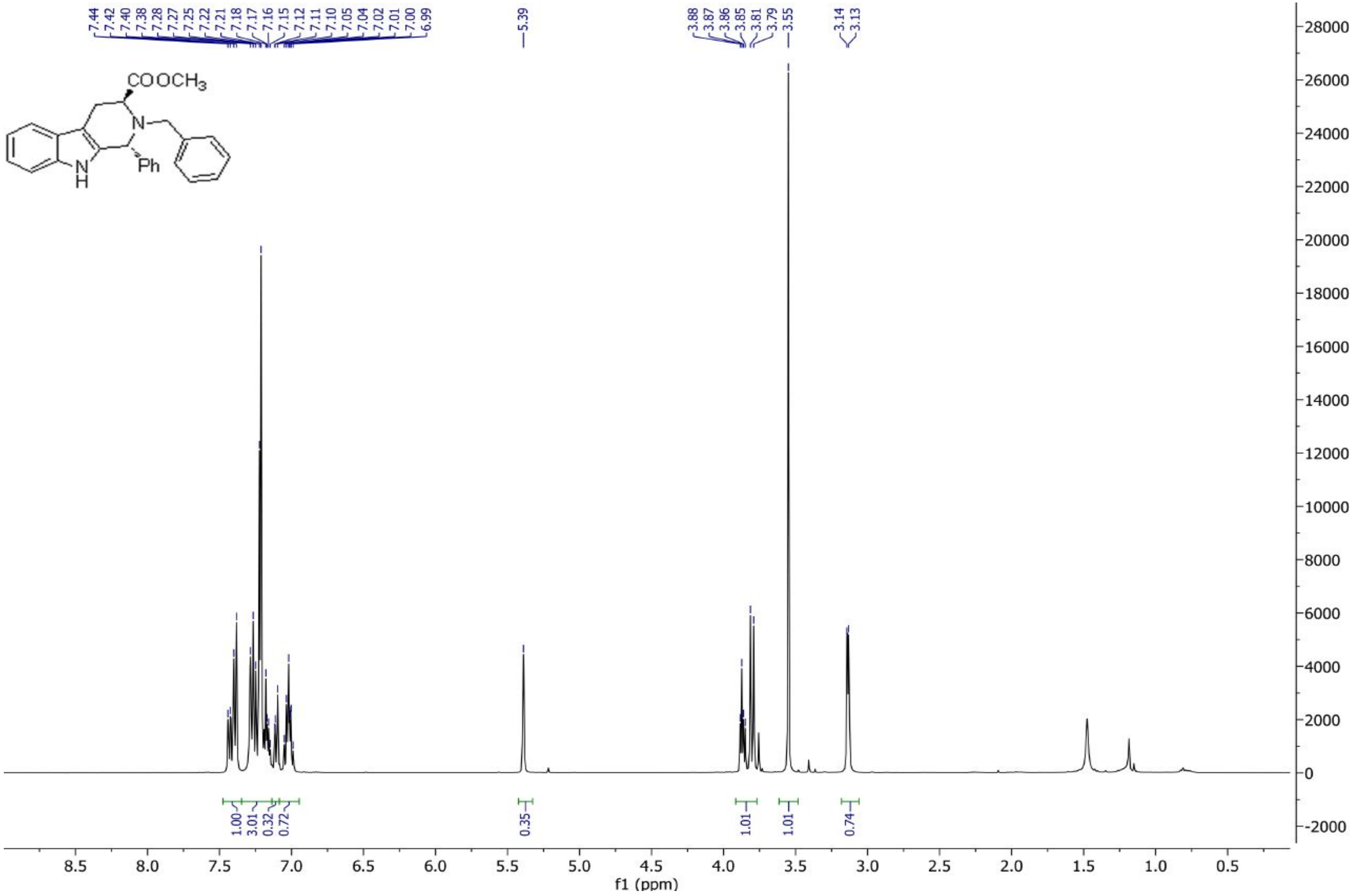

Figure S3: ^{1}H spectra of **6a**

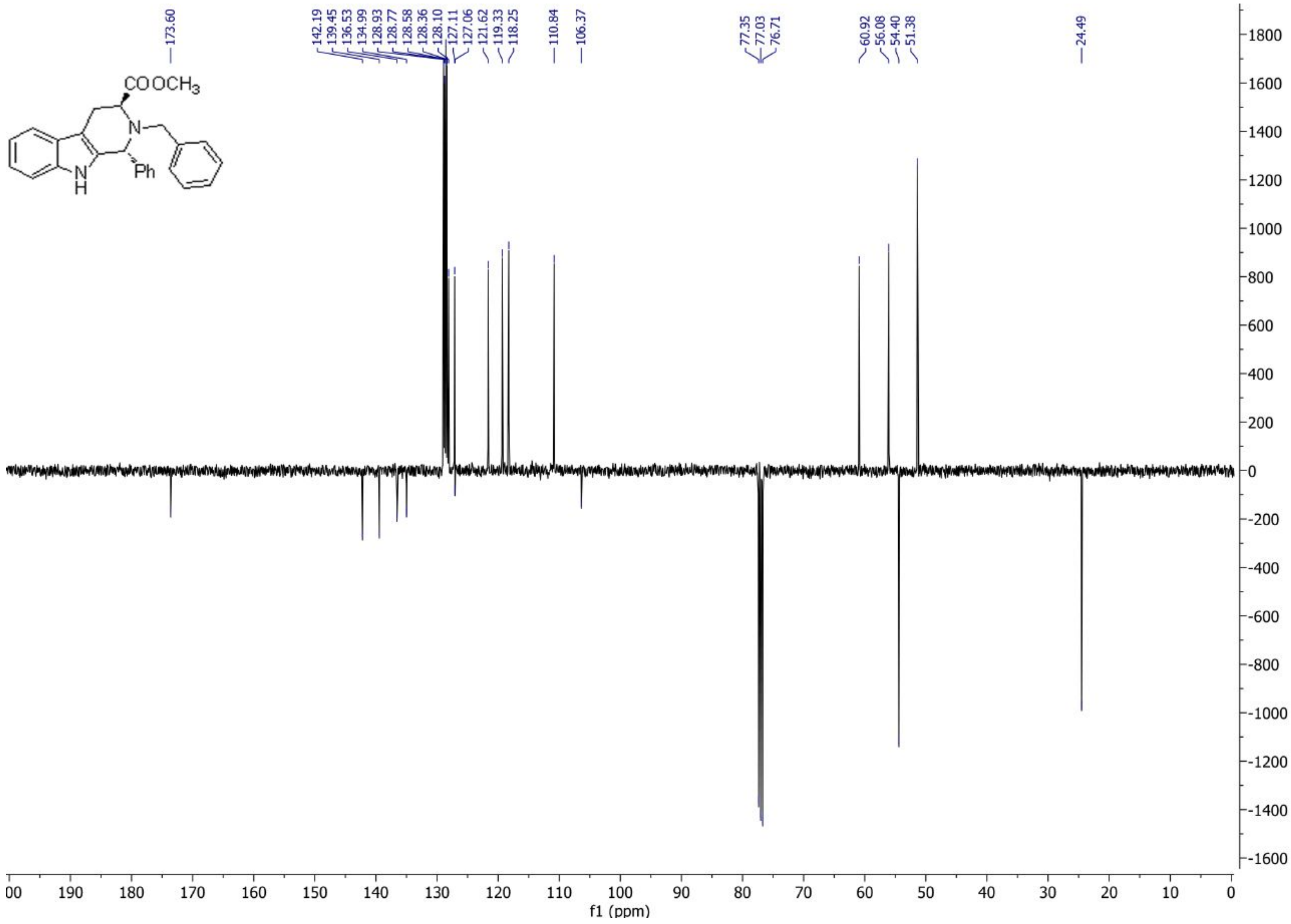

Figure S4: qDEPT spectra of **6a**

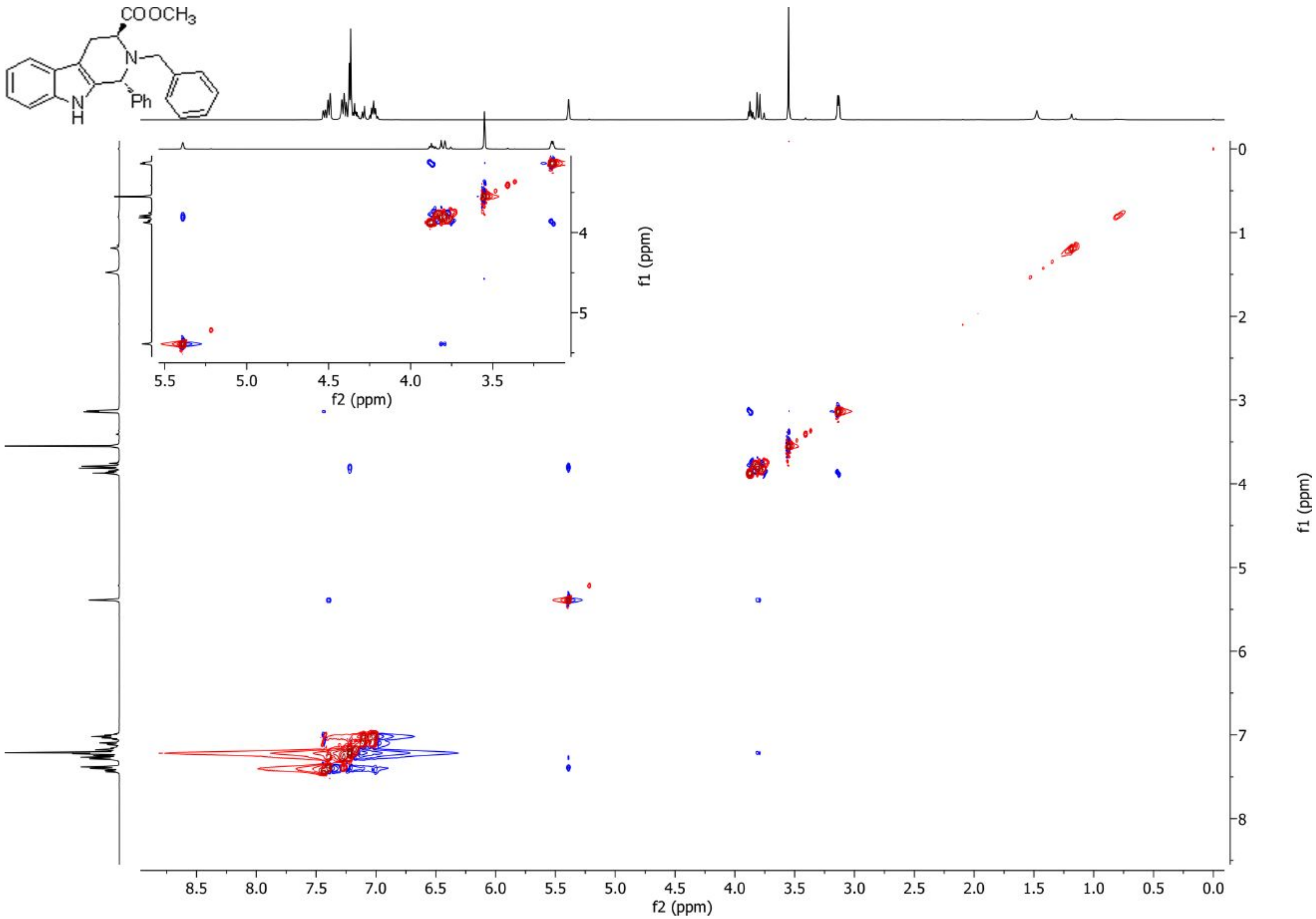

Figure S5: ROESY spectra of **6a**

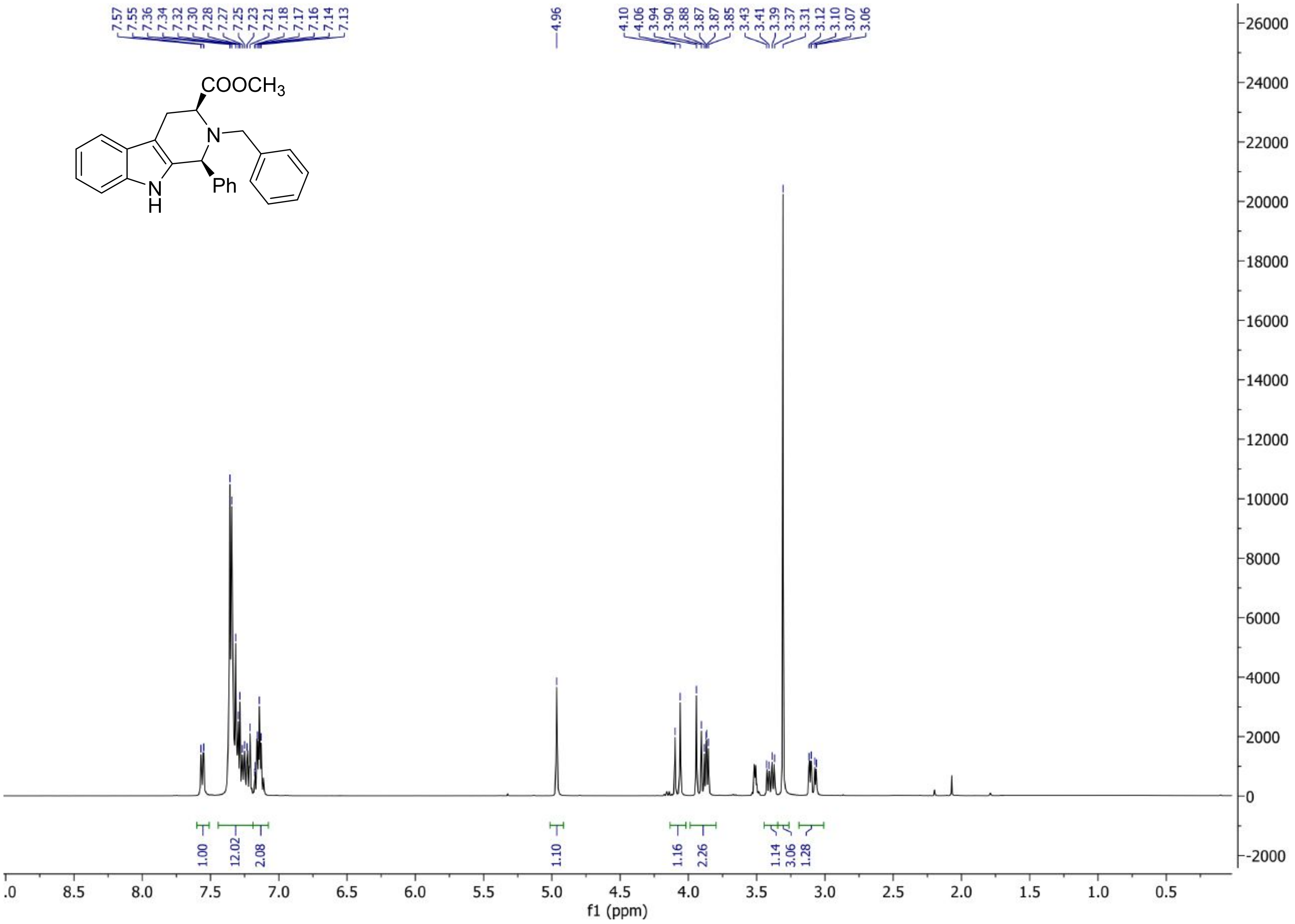

Figure S6: ^{1}H spectra of **6b**

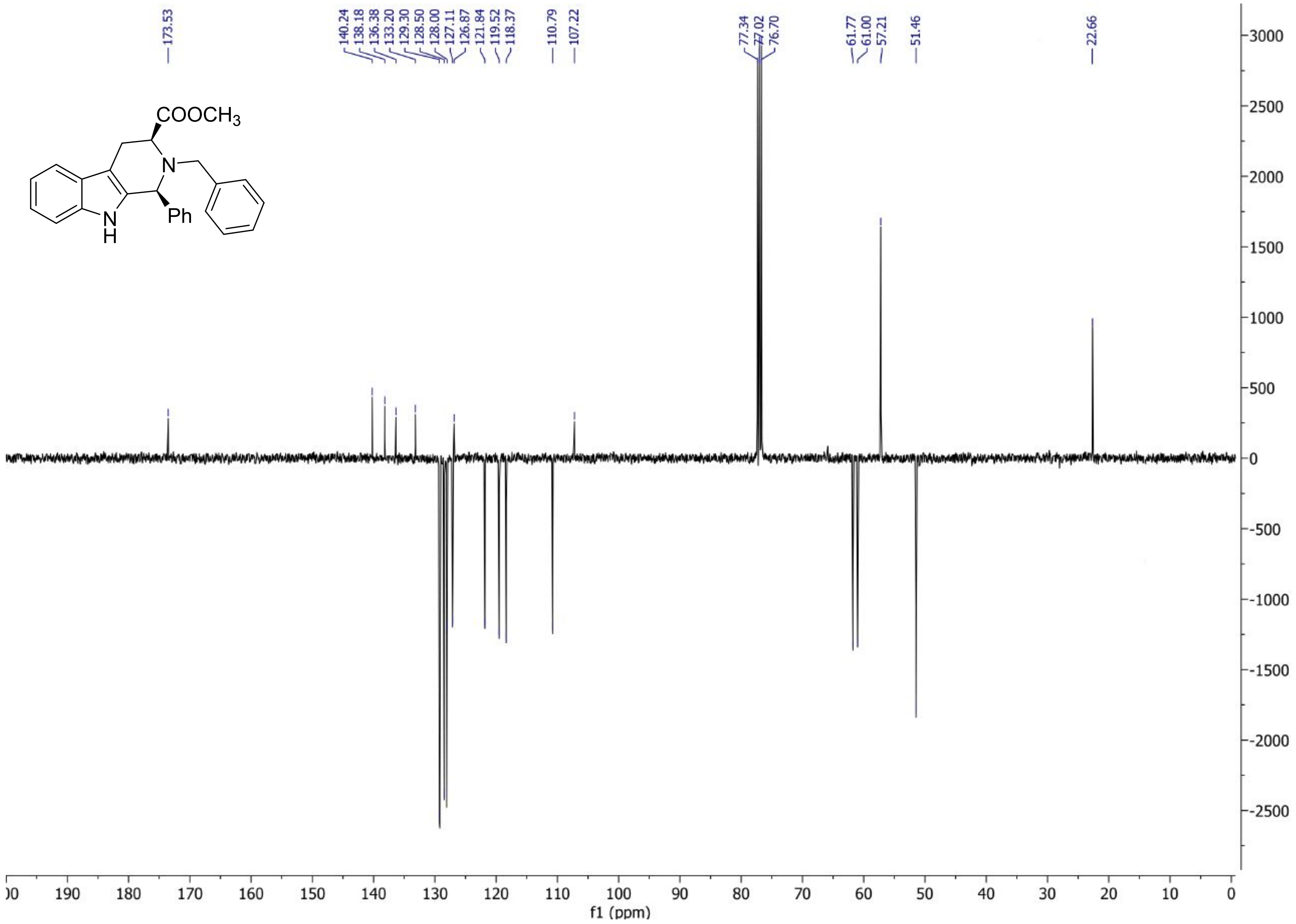

Figure S7: qDEPT spectra of **6b**

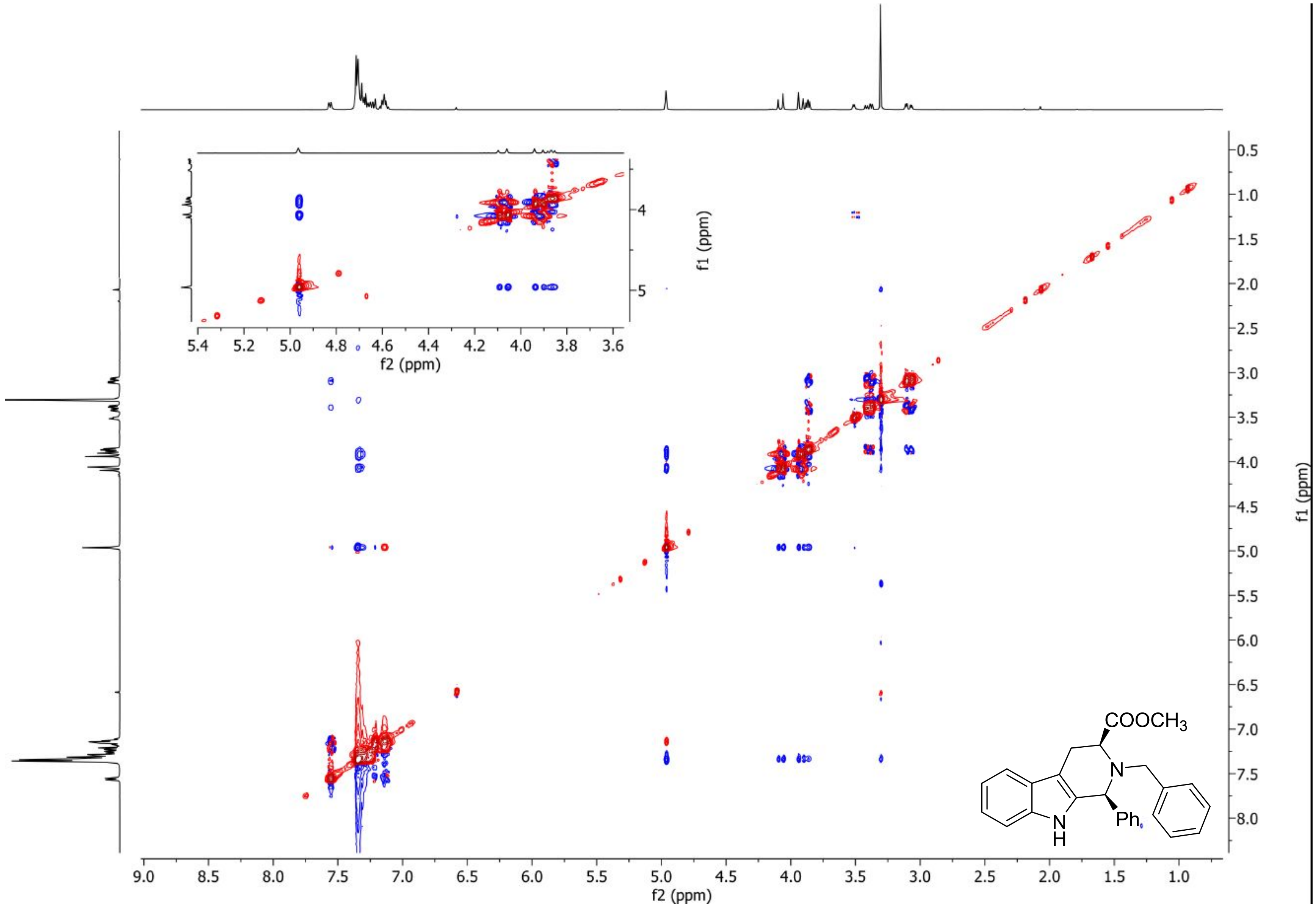

Figure S8: ROESY spectra of **6b**

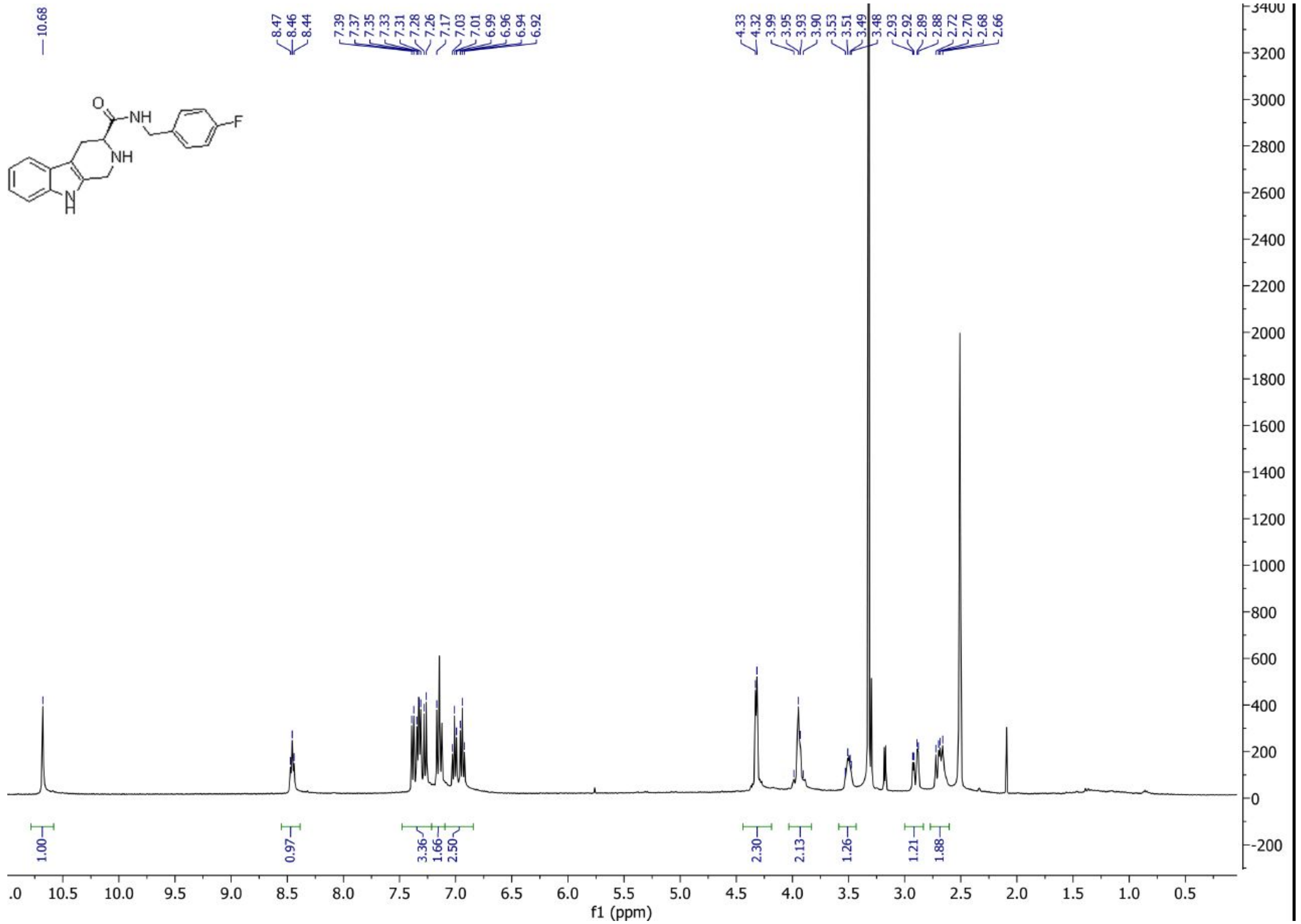

Figure S9: ^{1}H spectra of **9**

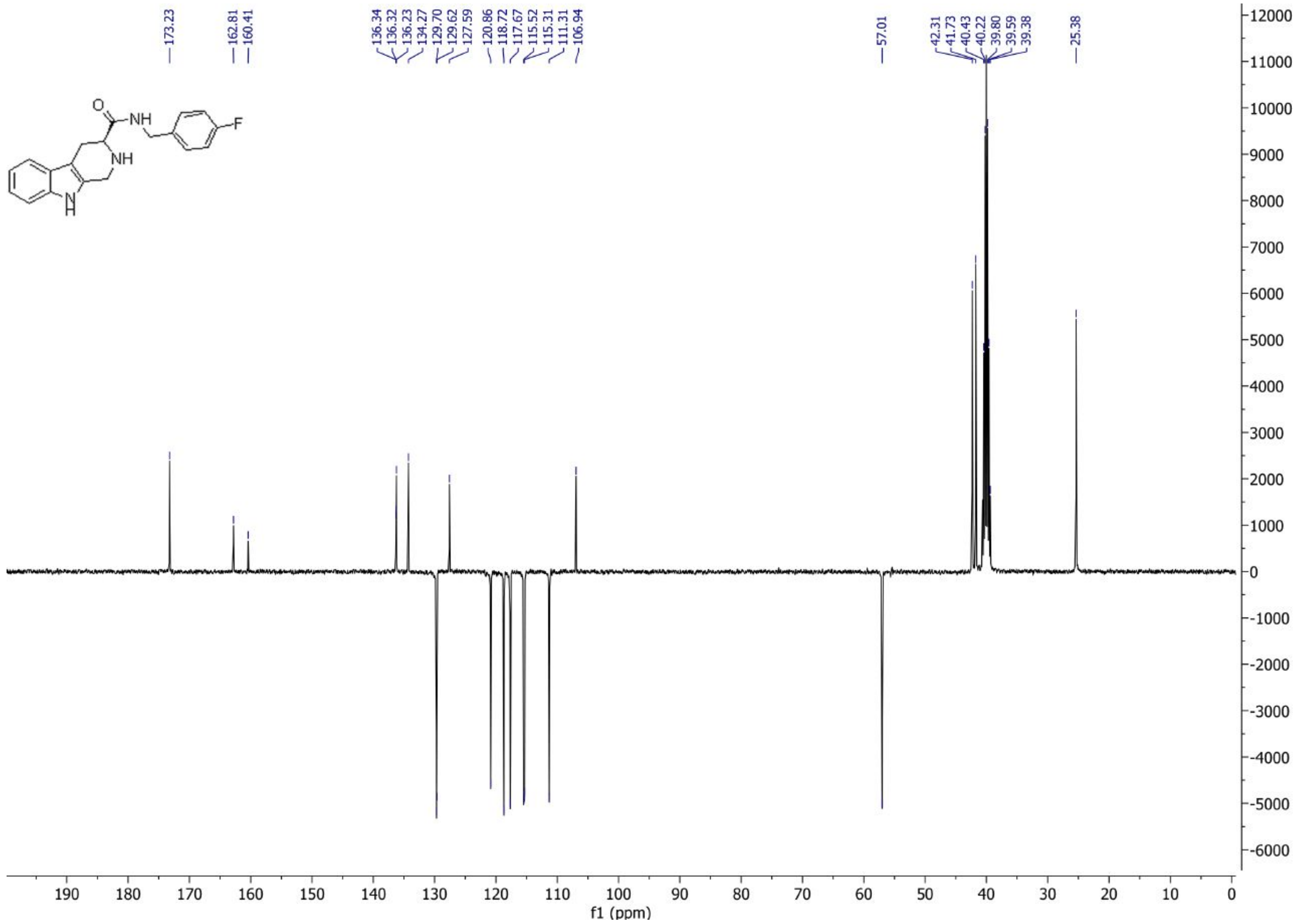

Figure S10: qDEPT spectra of **9**

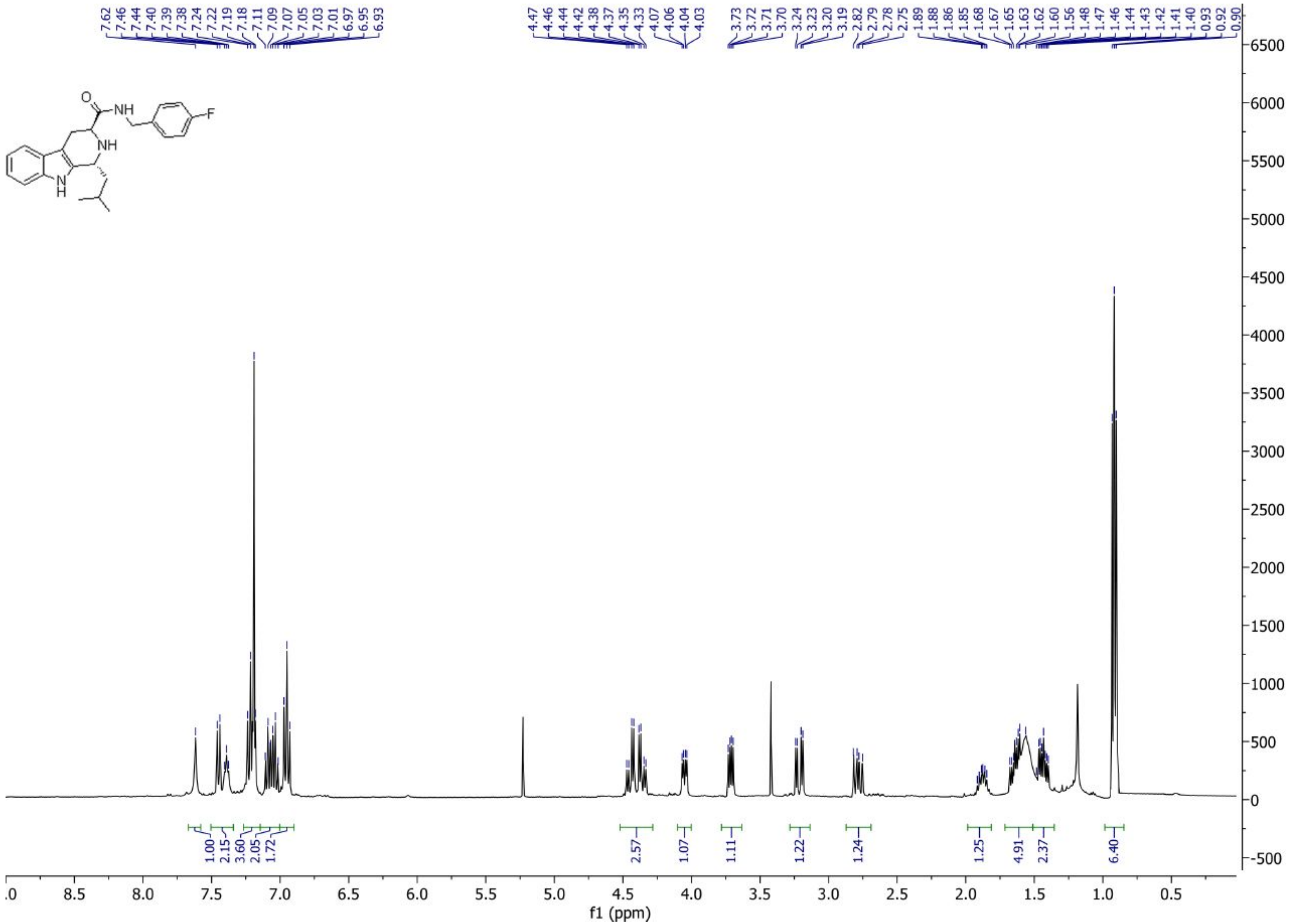

Figure S11: ^{1}H spectra of **10a**

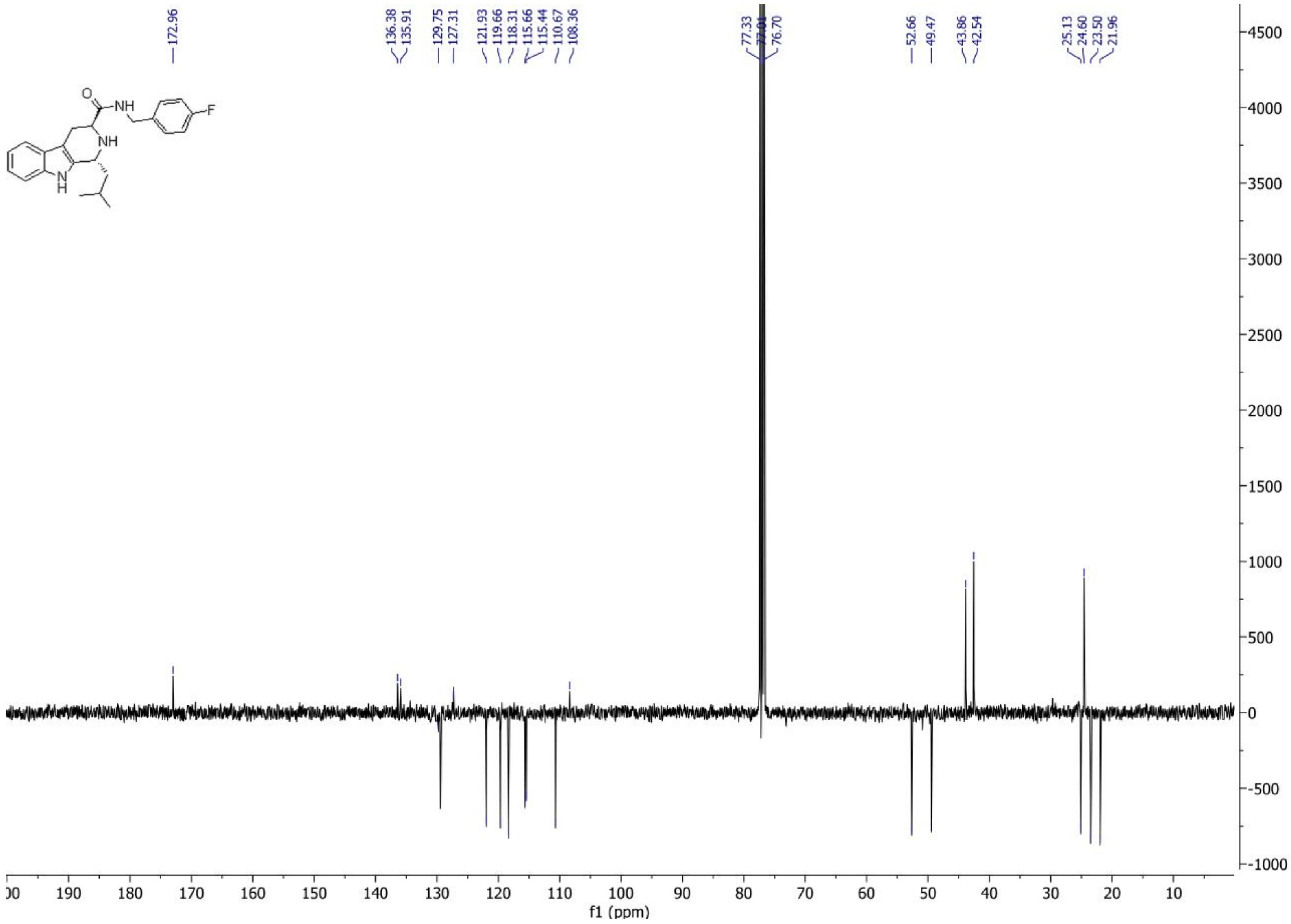

Figure S12: qDEPT spectra of **10a**

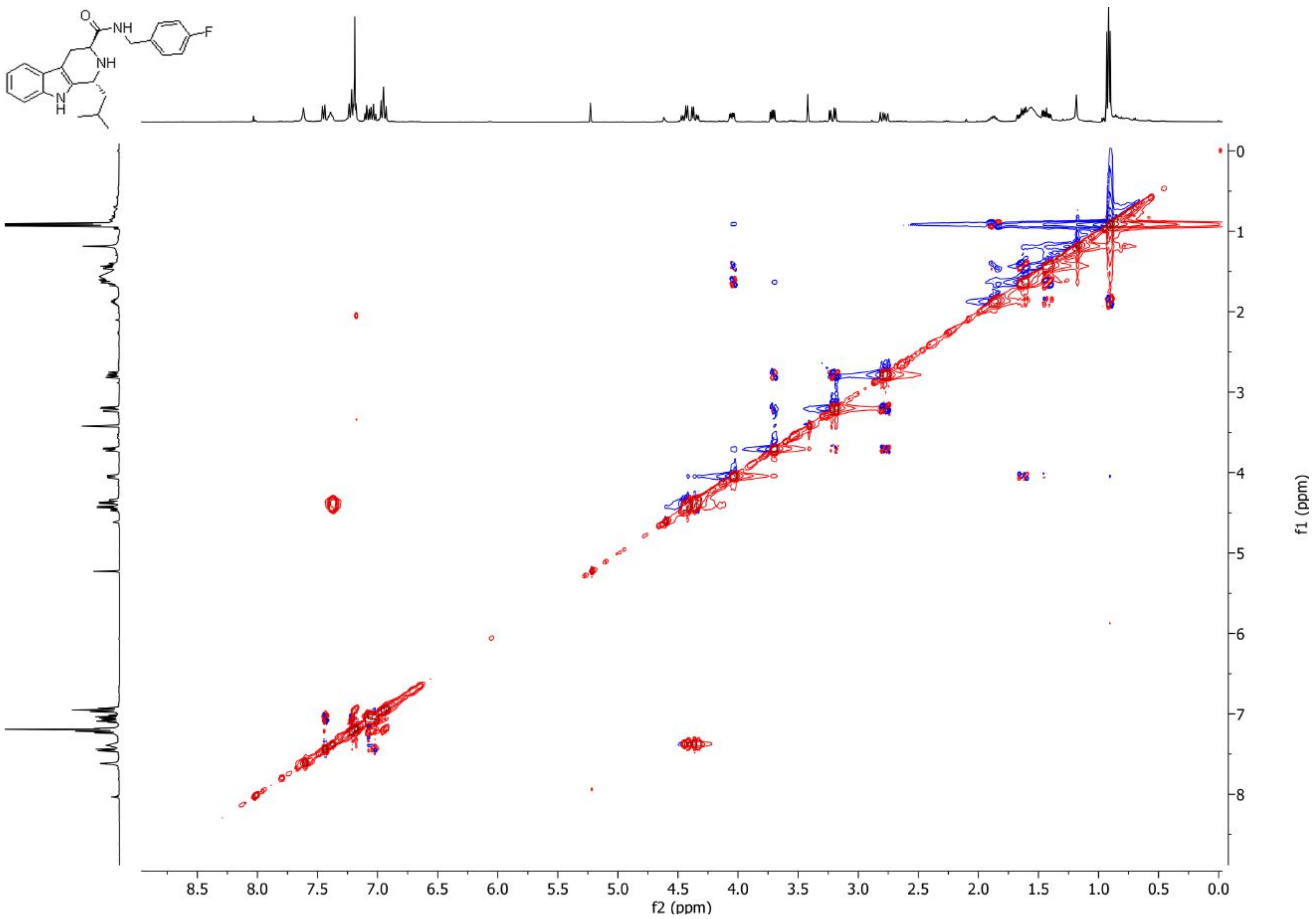

Figure S13: ROESY spectra of **10a**

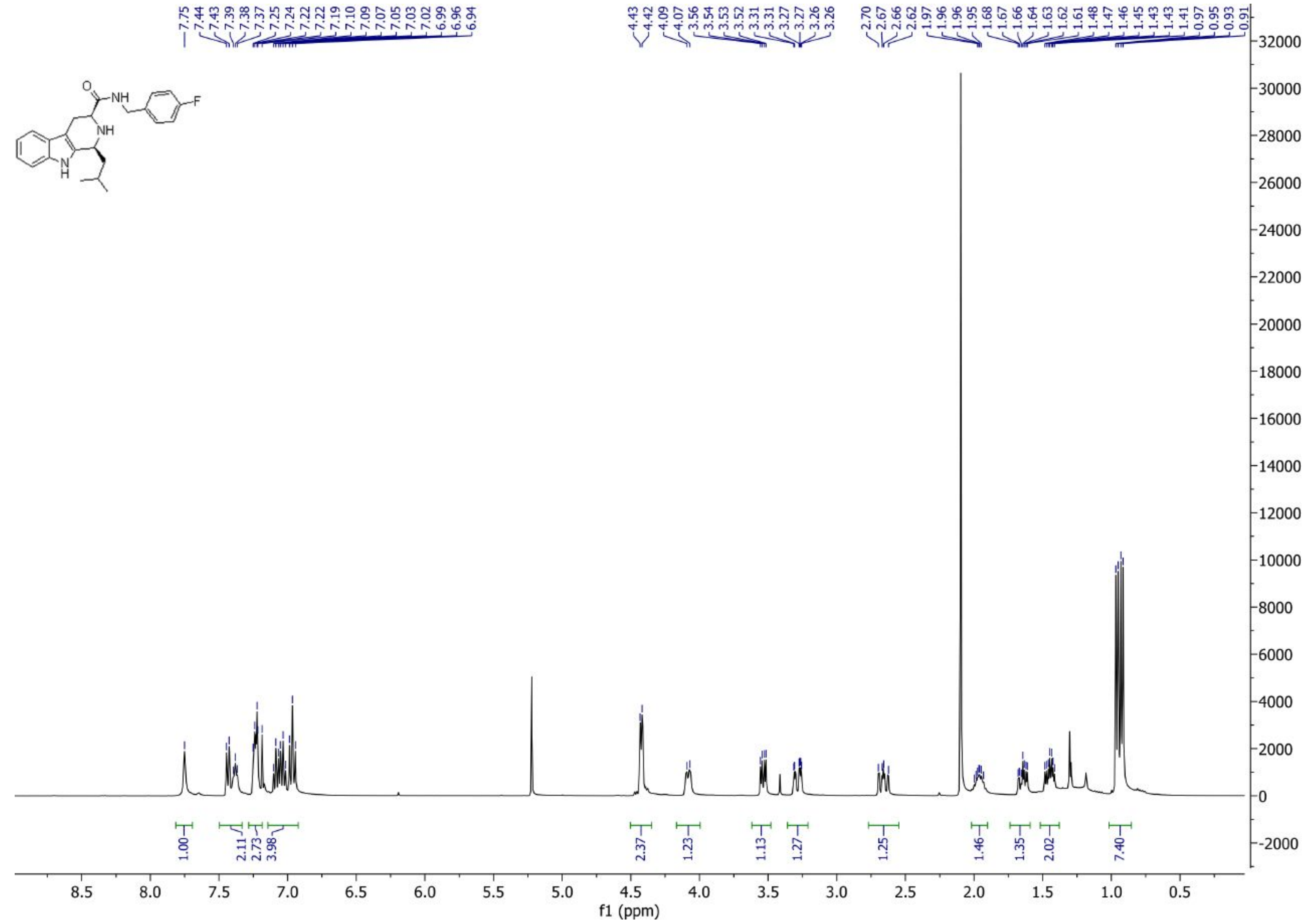

Figure S14: ^{1}H spectra of **10b**

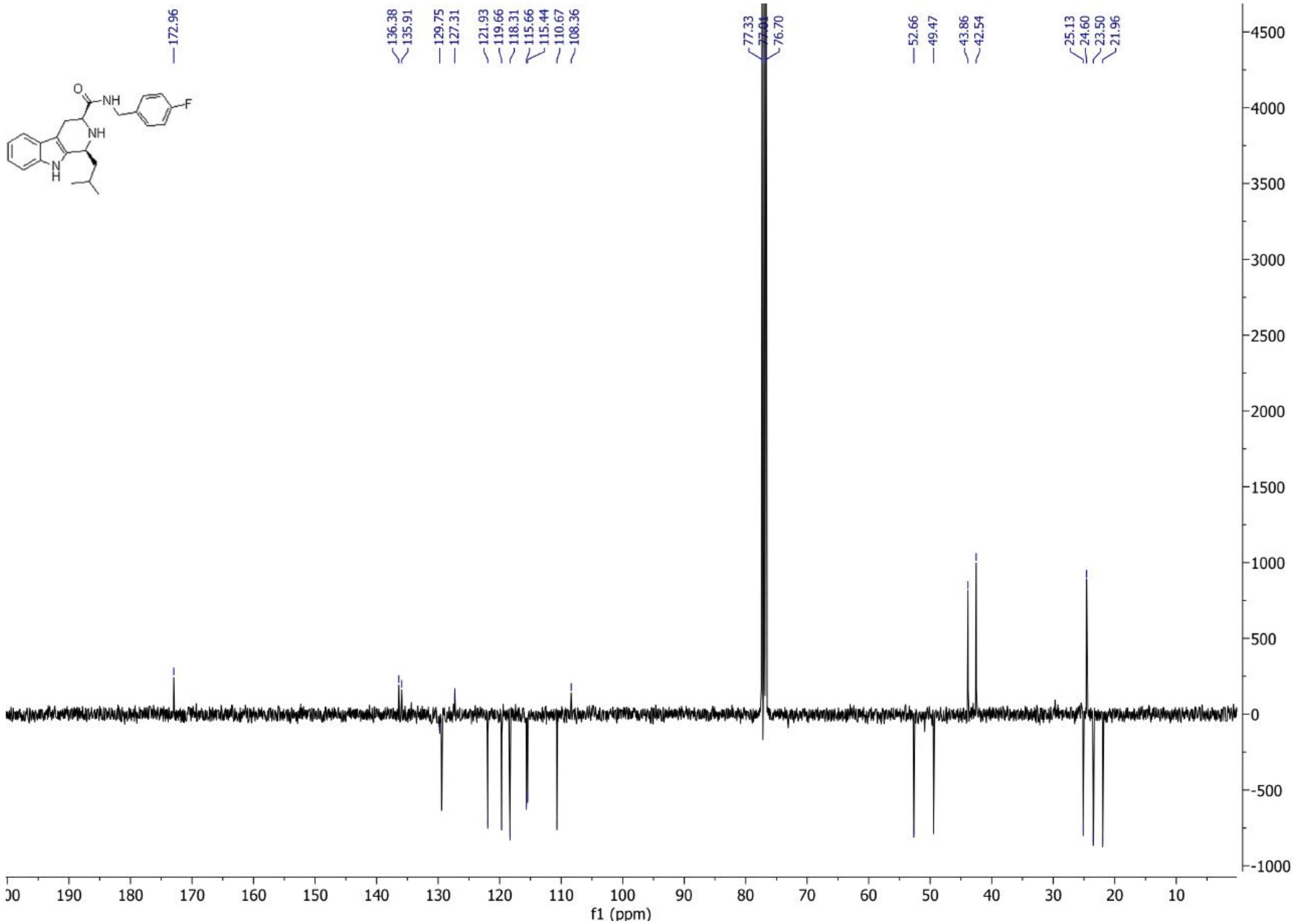

Figure S15: qDEPT spectra of **10b**

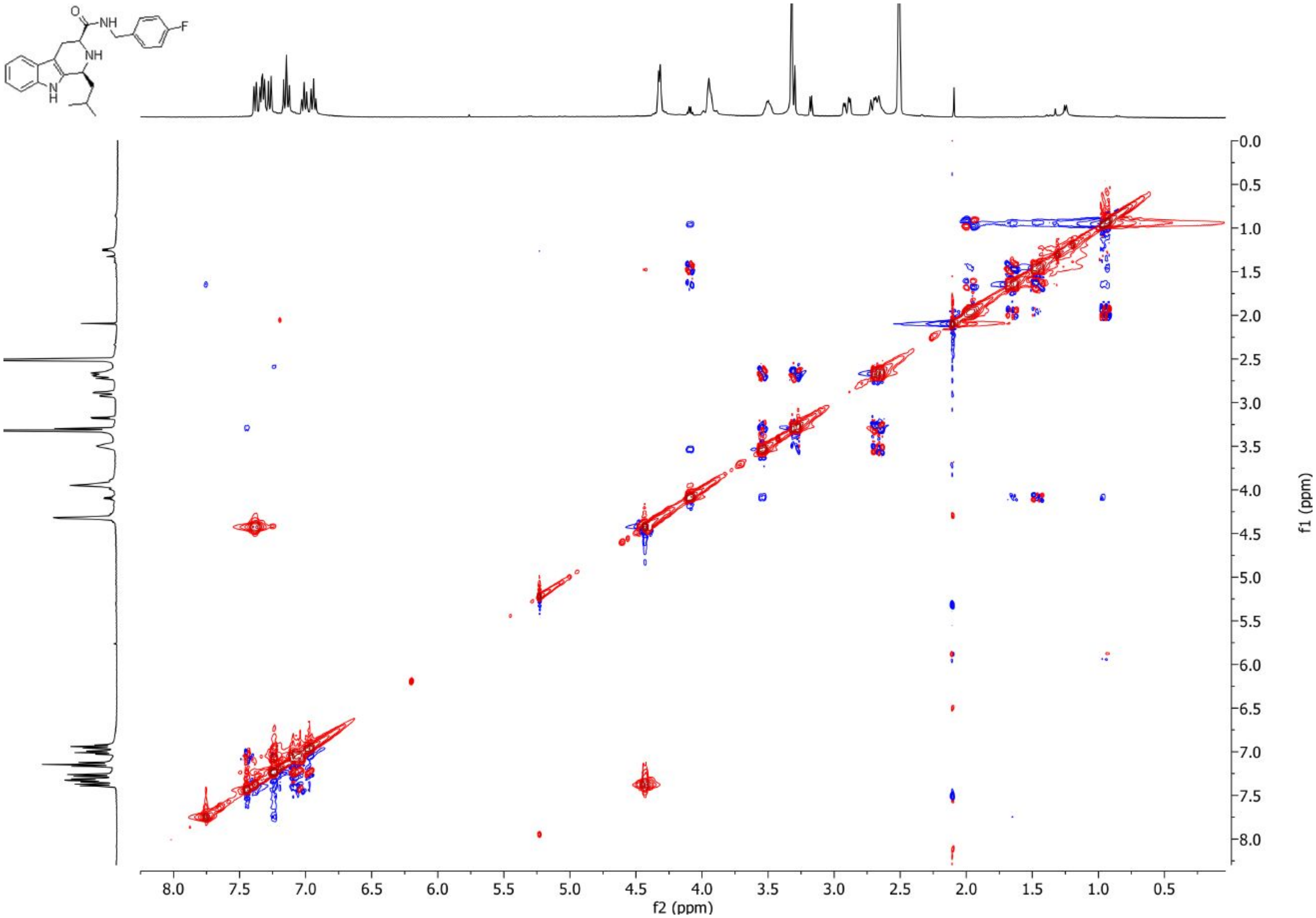

Figure S16: ROESY spectra of **10b**

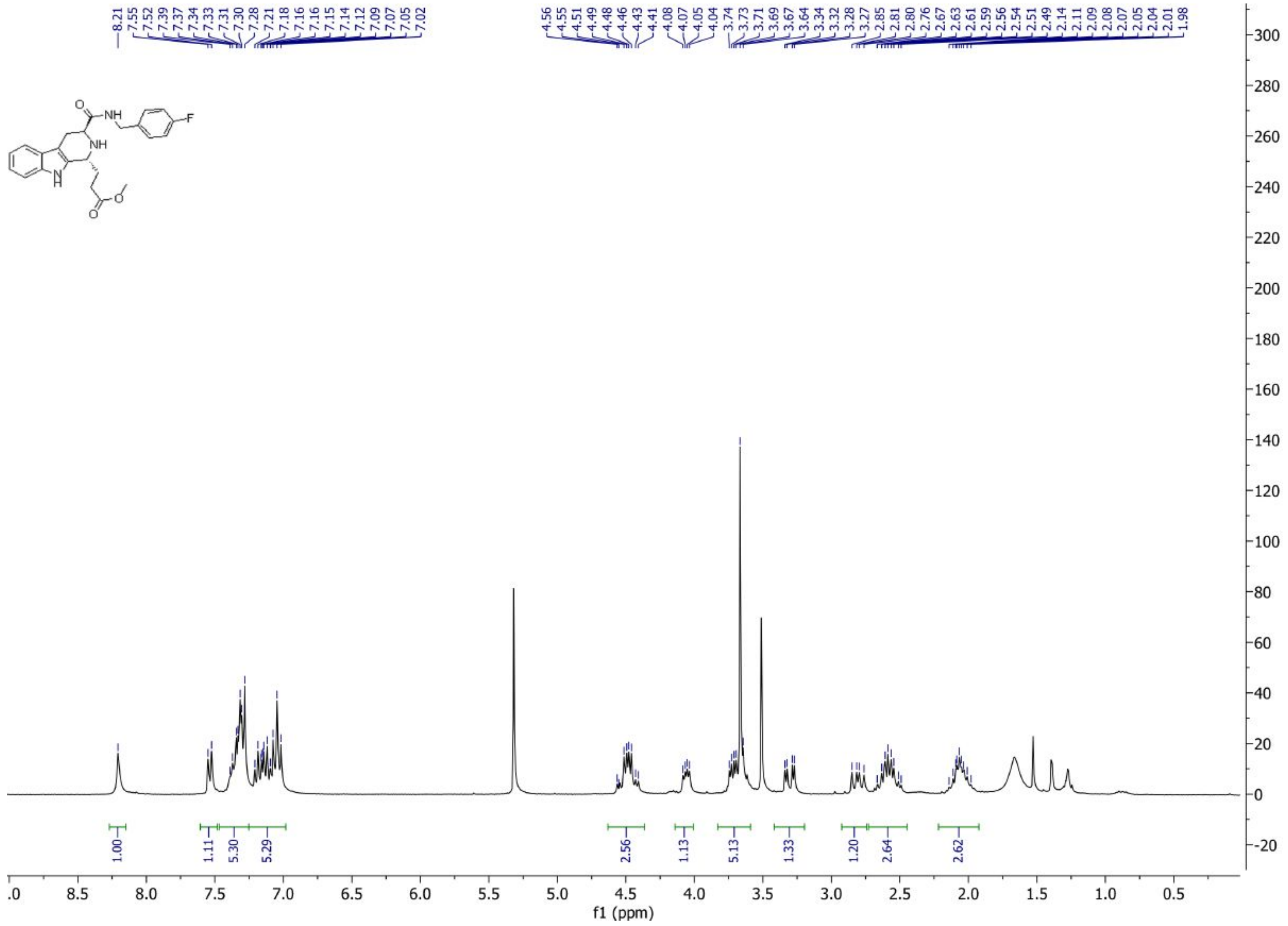

Figure S17: ^{1}H spectra of **11a**

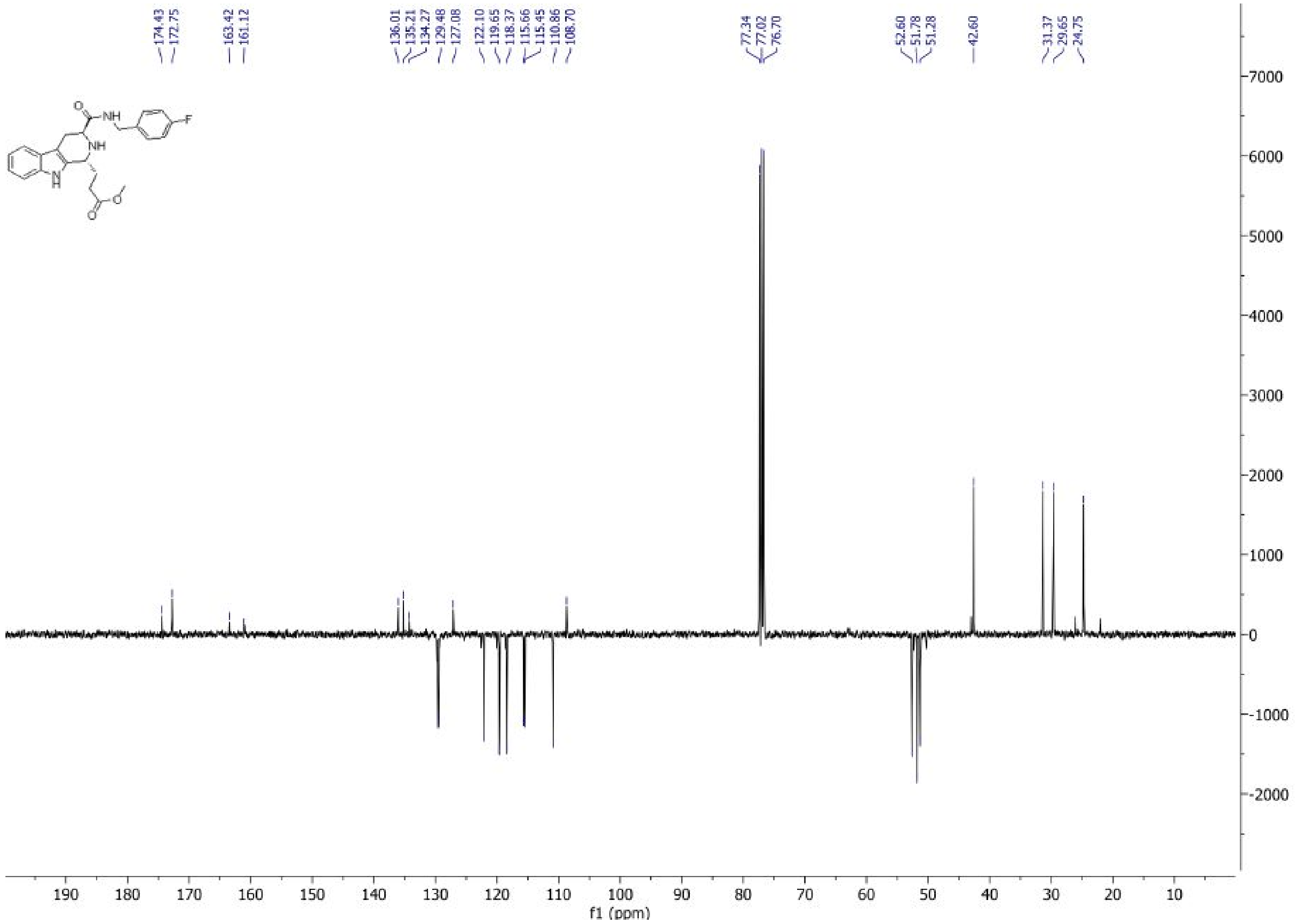

Figure S18: qDEPT spectra of **11a**

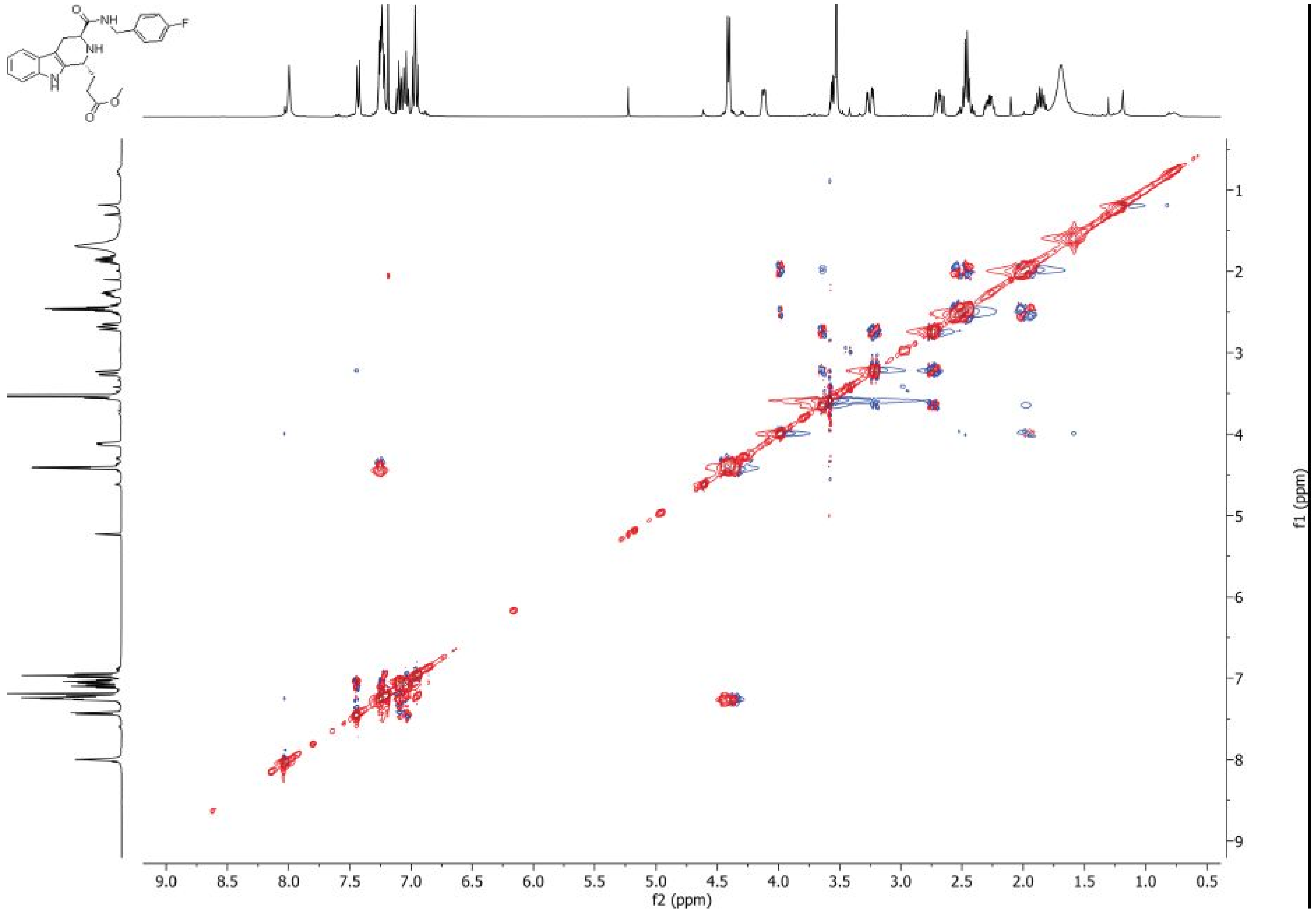

Figure S19: ROESY spectra of **11a**

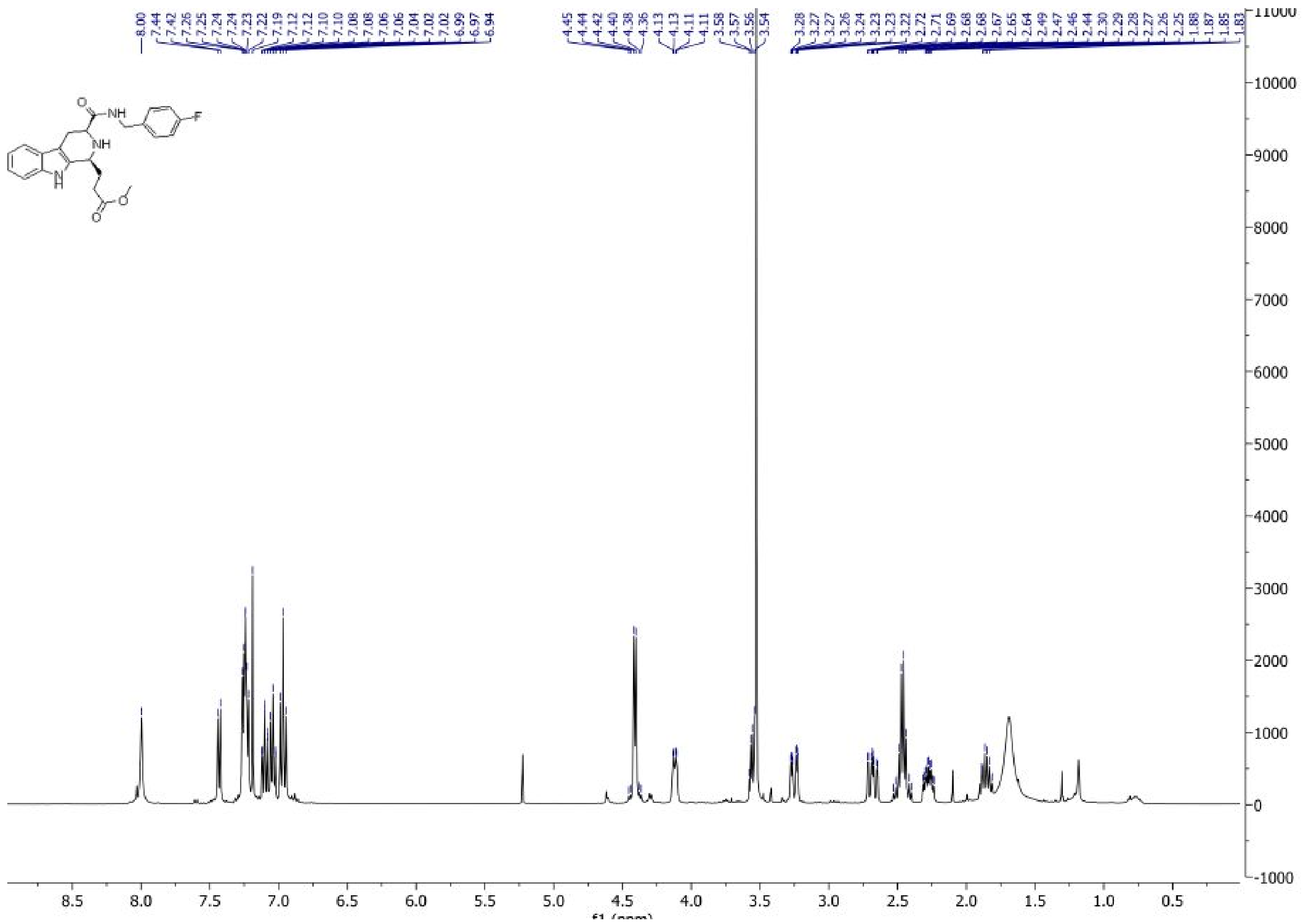

Figure S20: ^{1}H spectra of **11b**

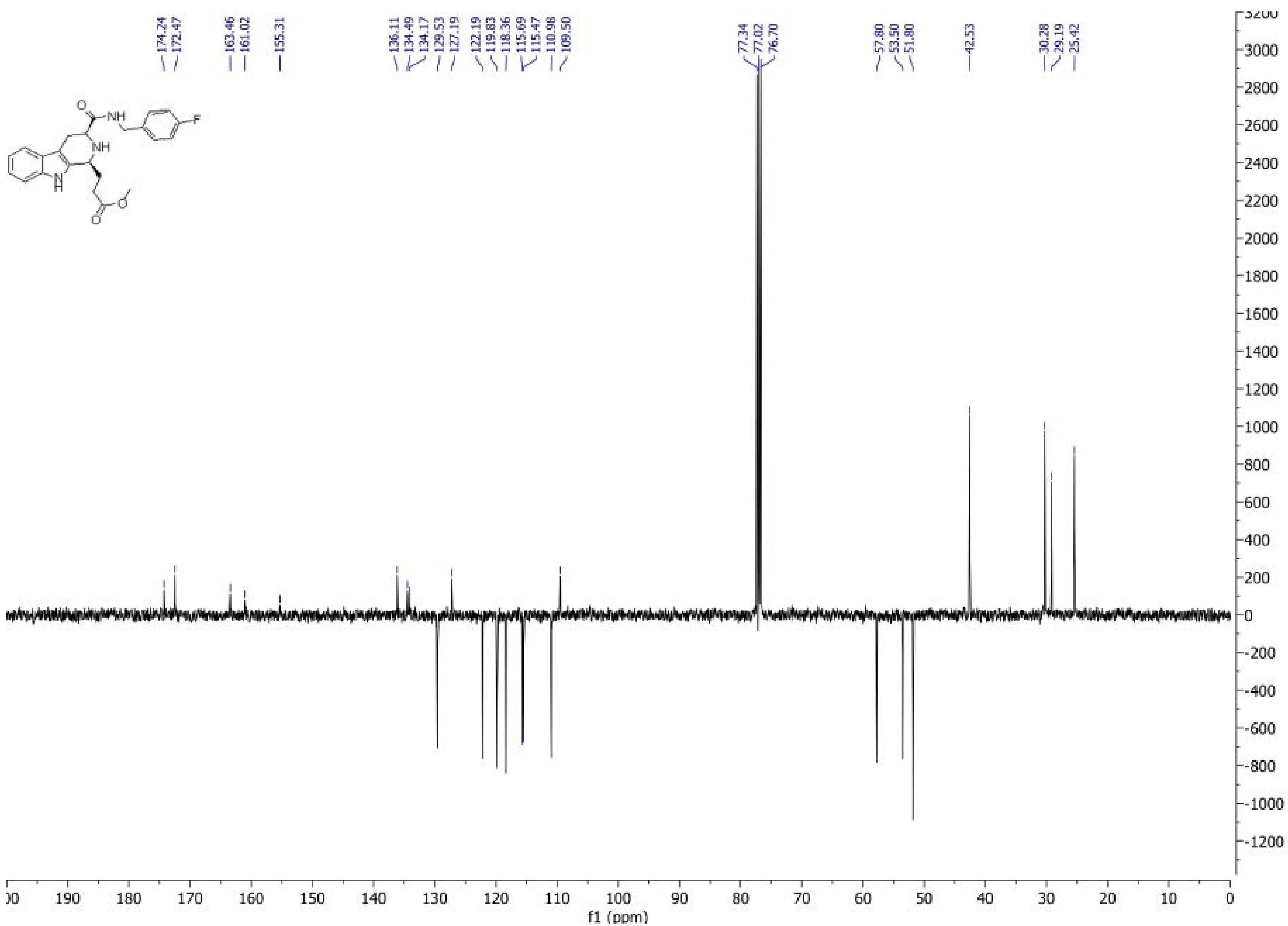

Figure S21: qDEPT spectra of **11b**

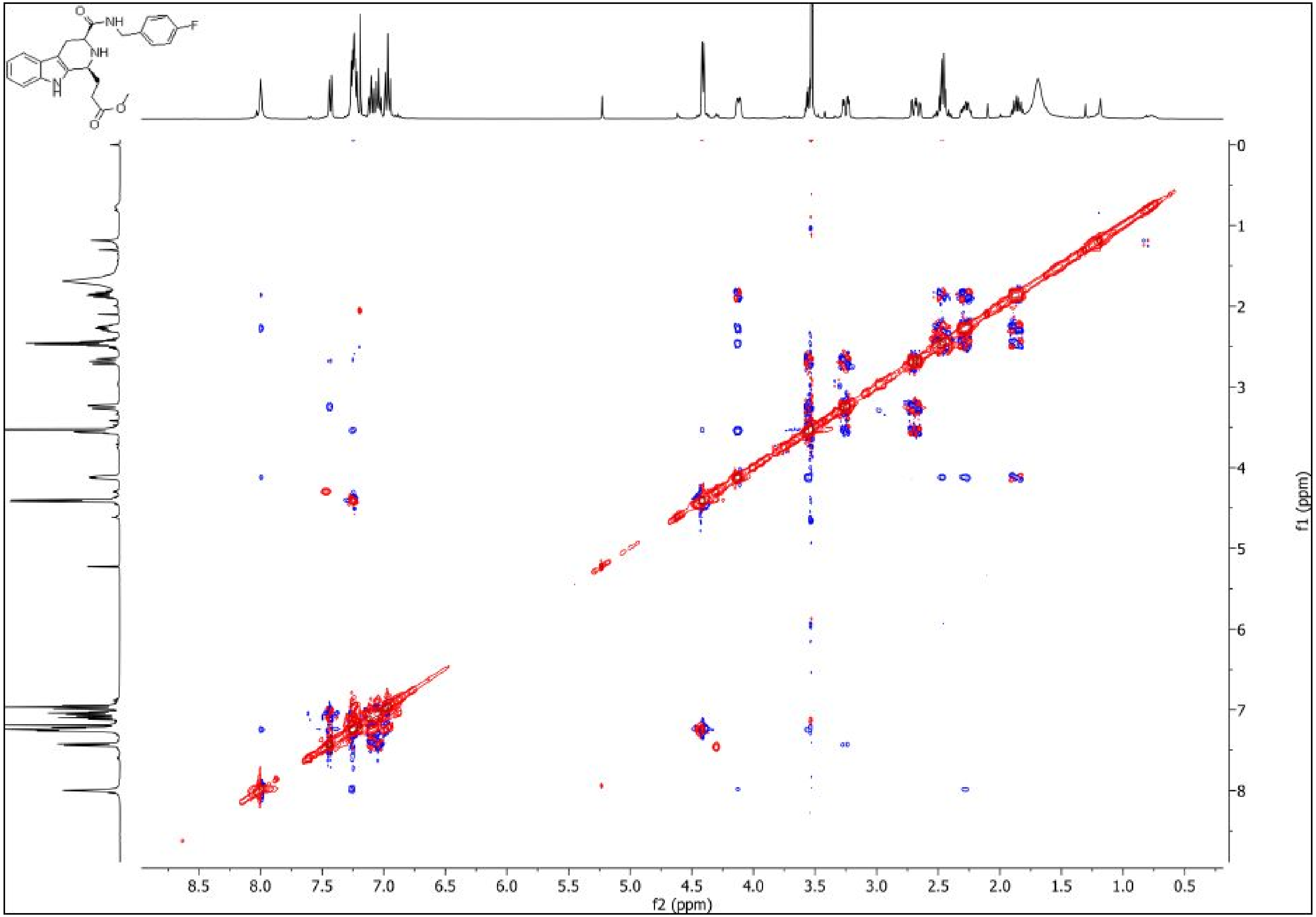

Figure S22: ROESY spectra of **11b**

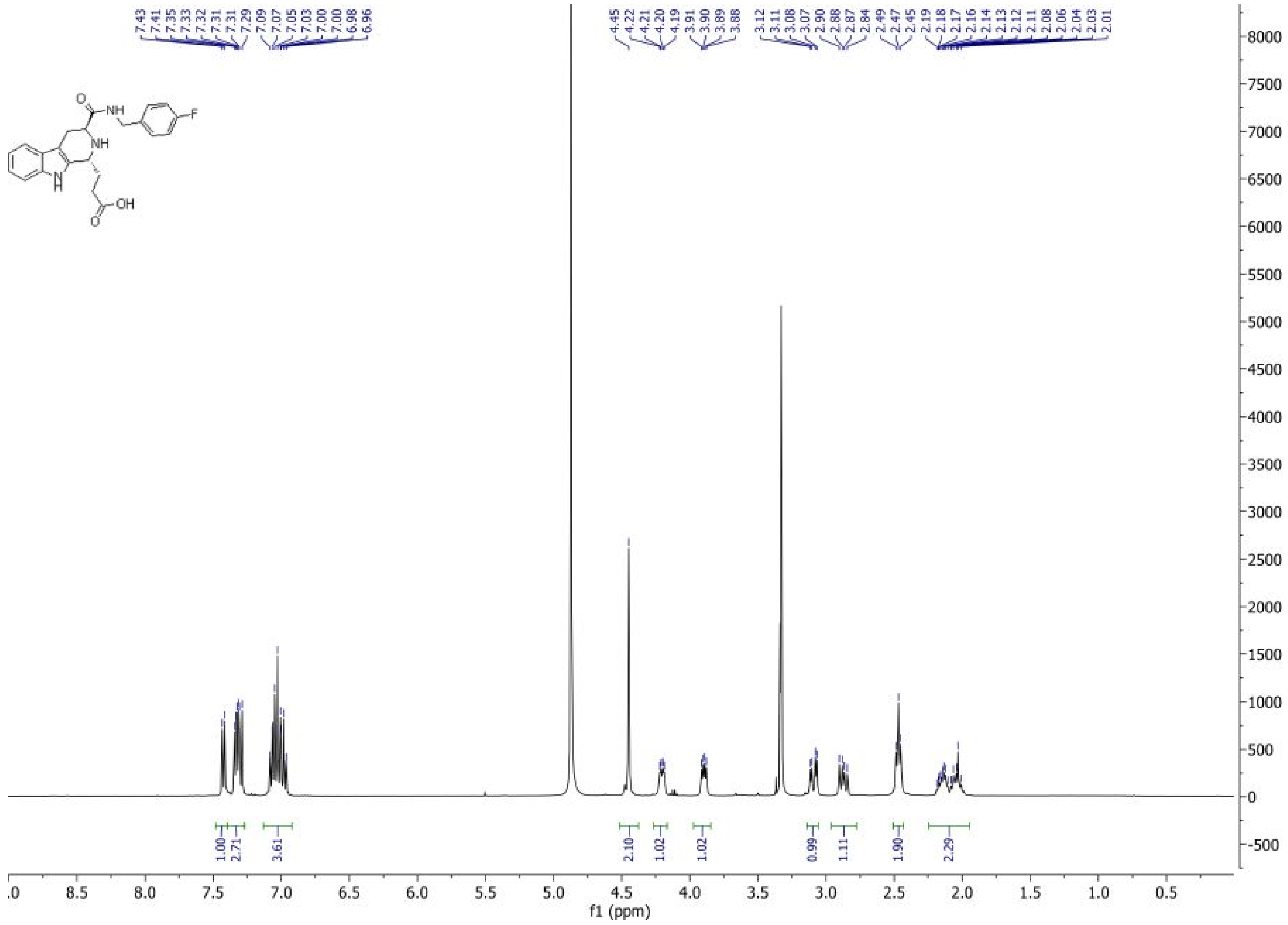

Figure S23: ^{1}H spectra of **12a**

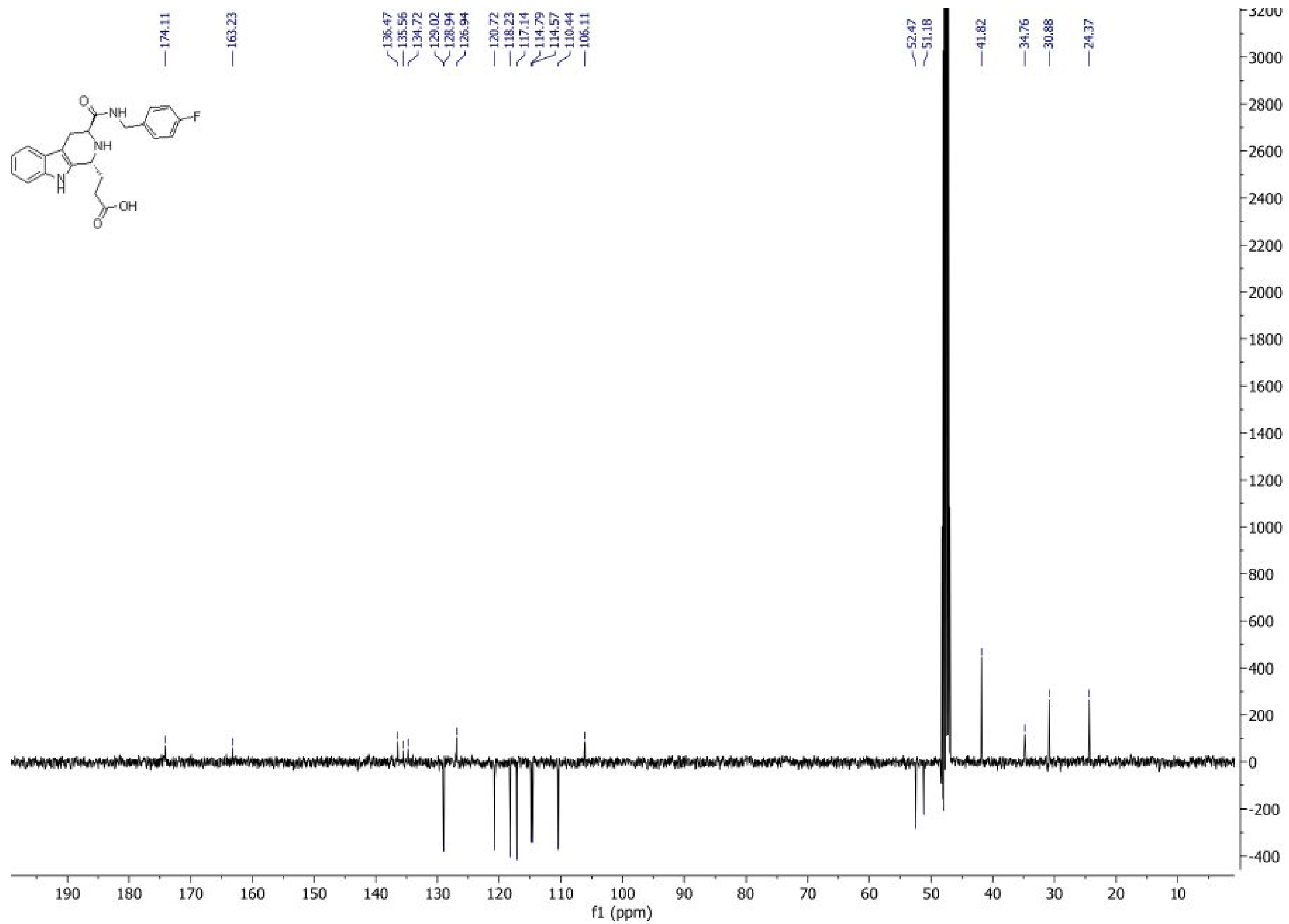

Figure S24: qDEPT spectra of **12a**

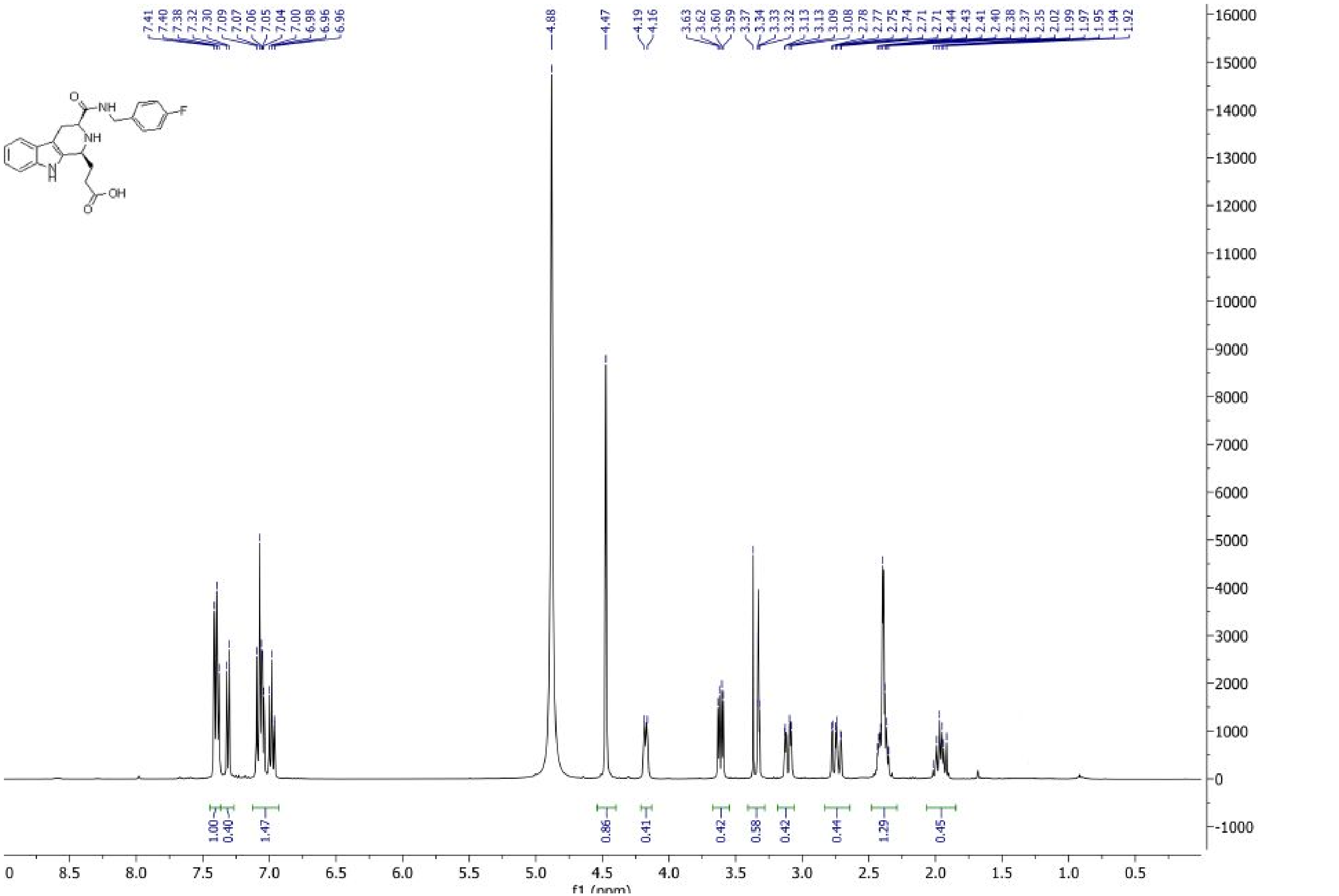

Figure S25: ^{1}H spectra of **12b**

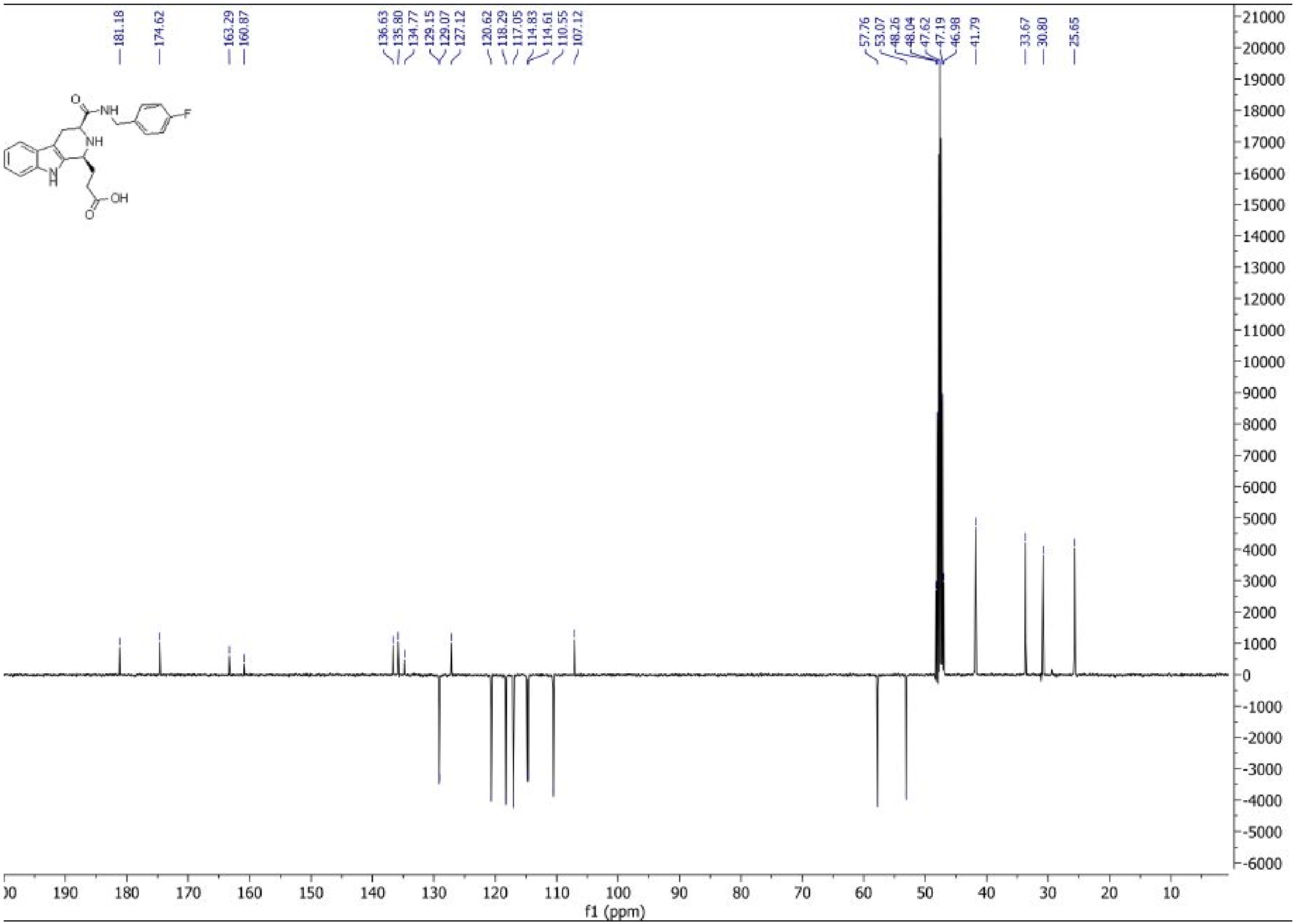

Figure S26: qDEPT spectra of **12b**

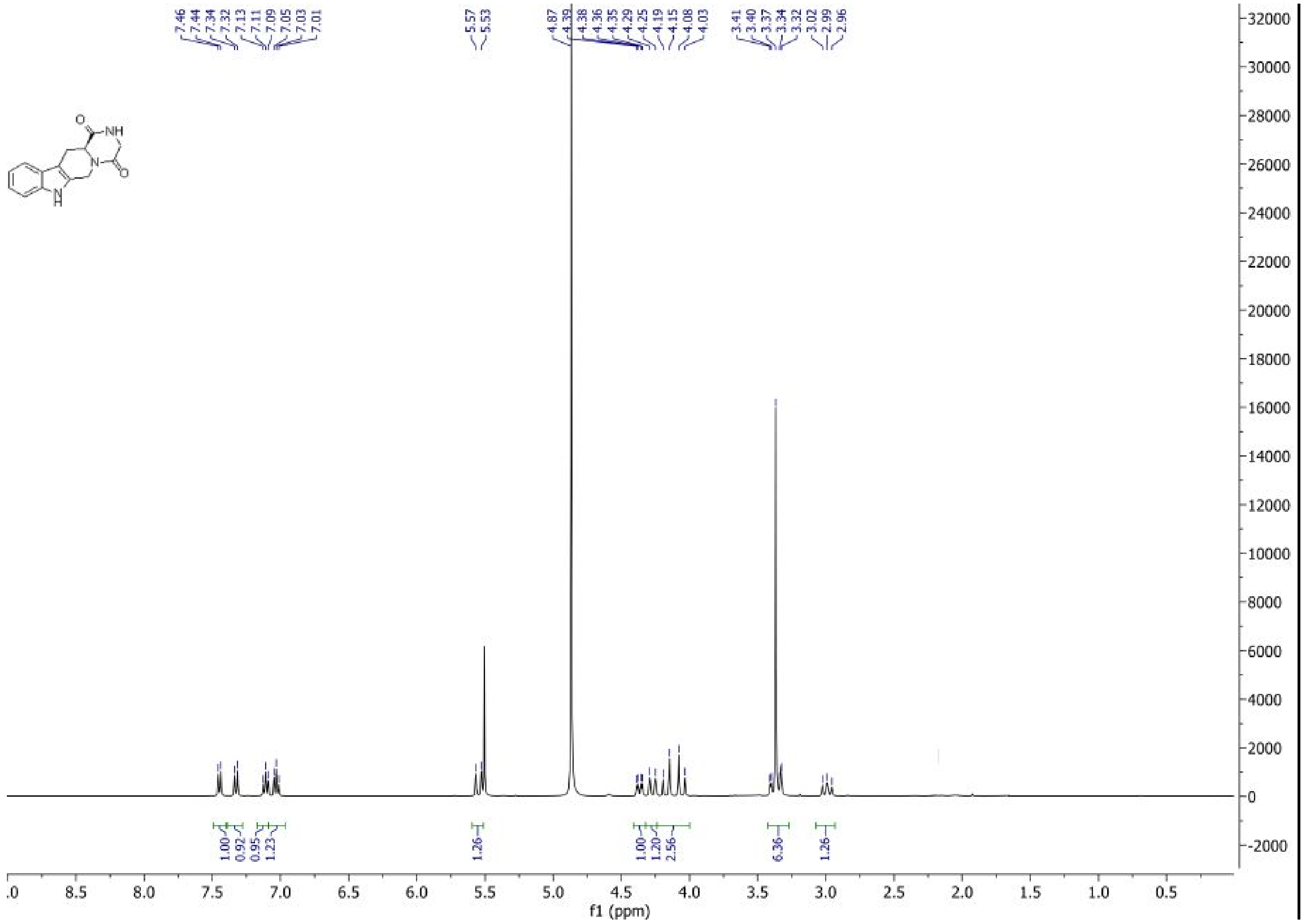

Figure S27: ^{1}H spectra of **21**

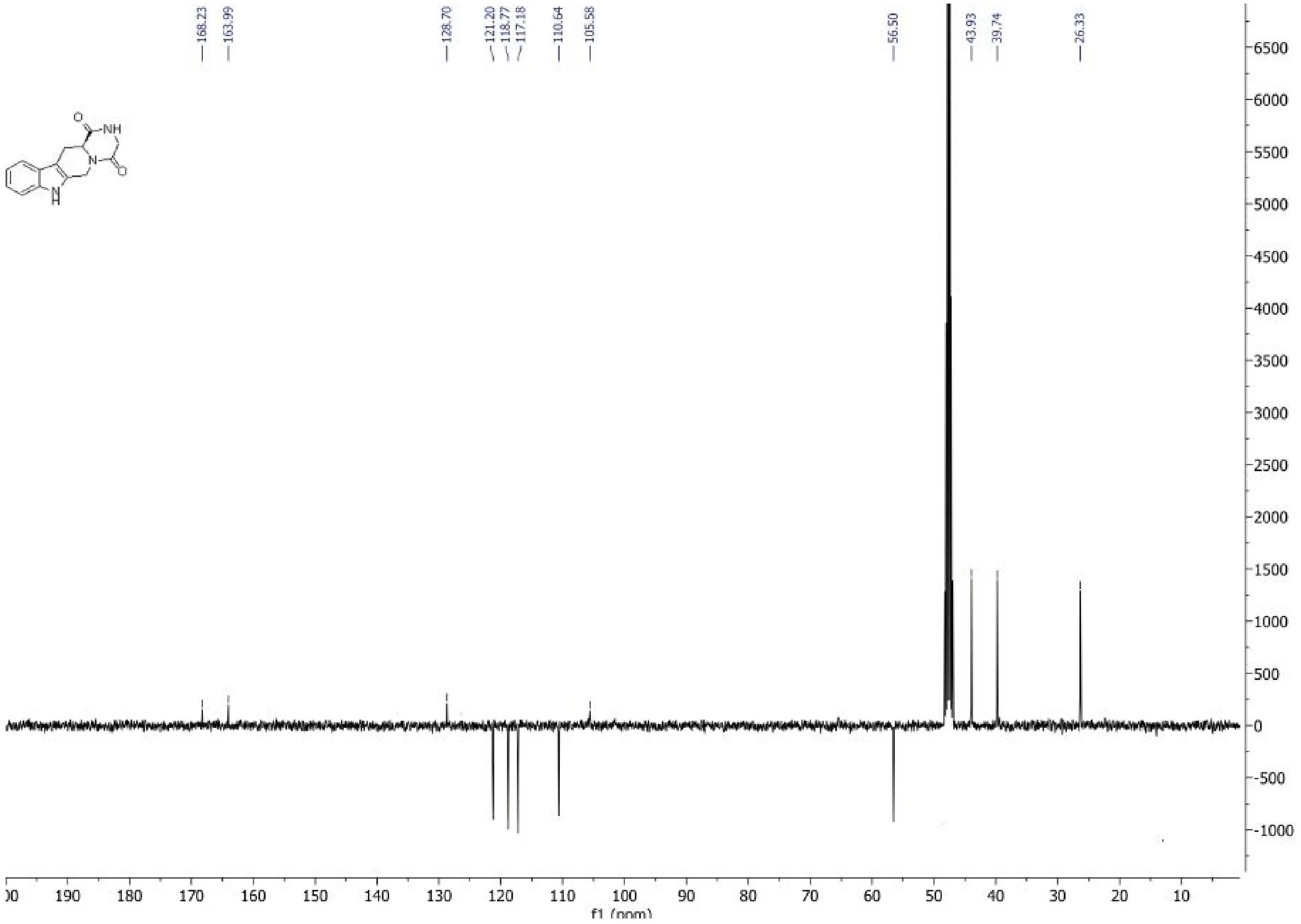

Figure S28: qDEPT spectra of **21**

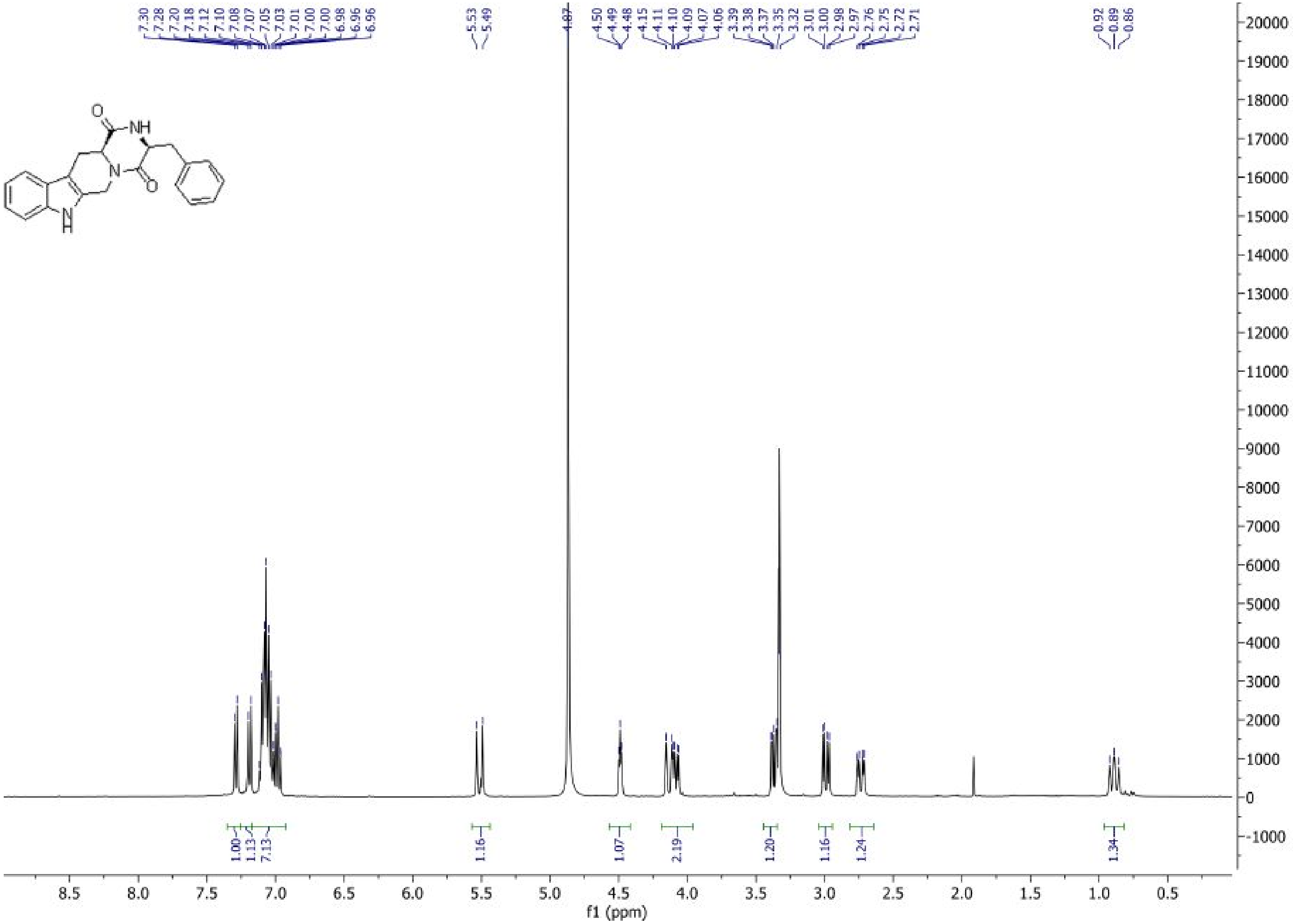

Figure S29: ^{1}H spectra of **22**

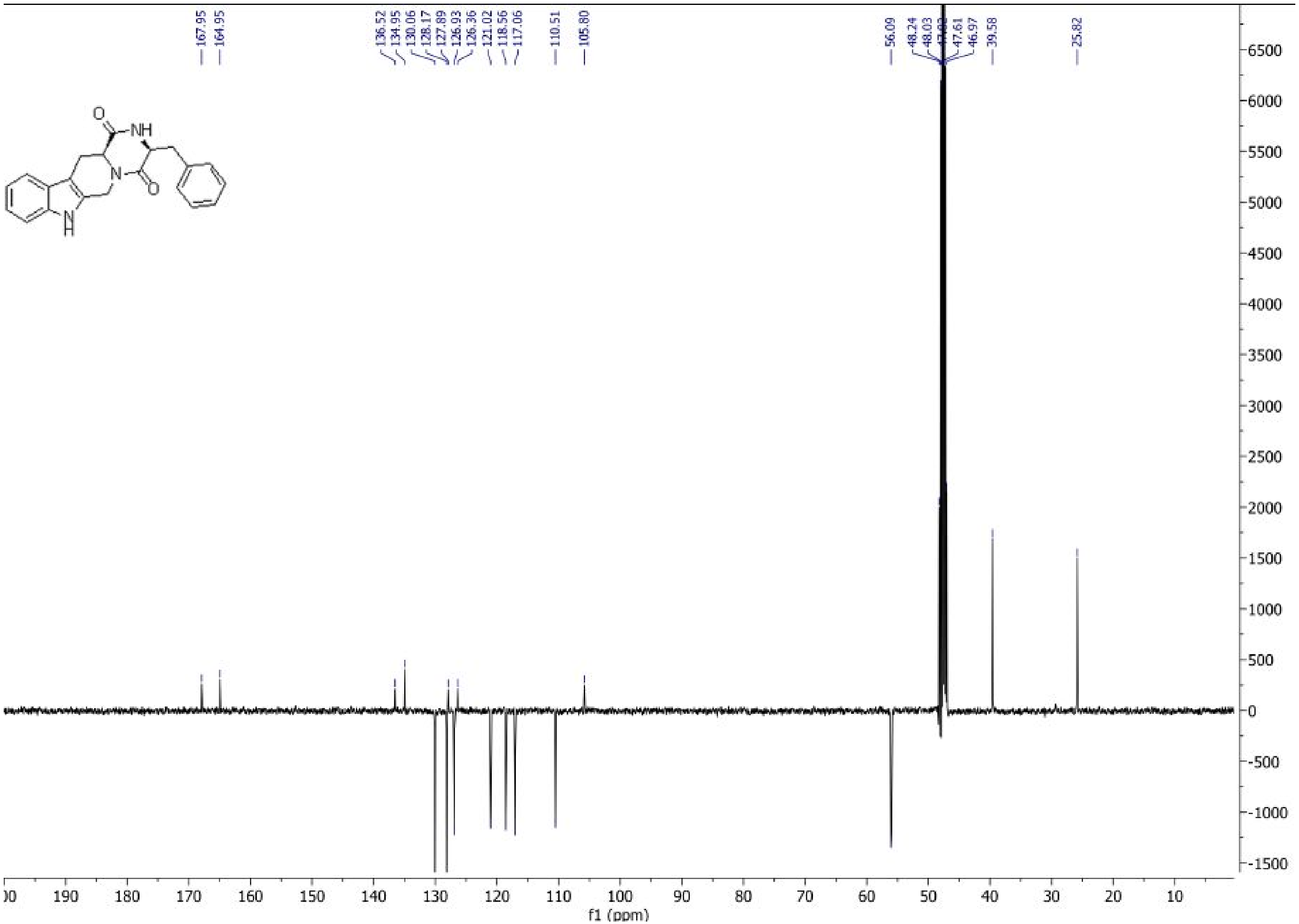

Figure S30: qDEPT spectra of **22**

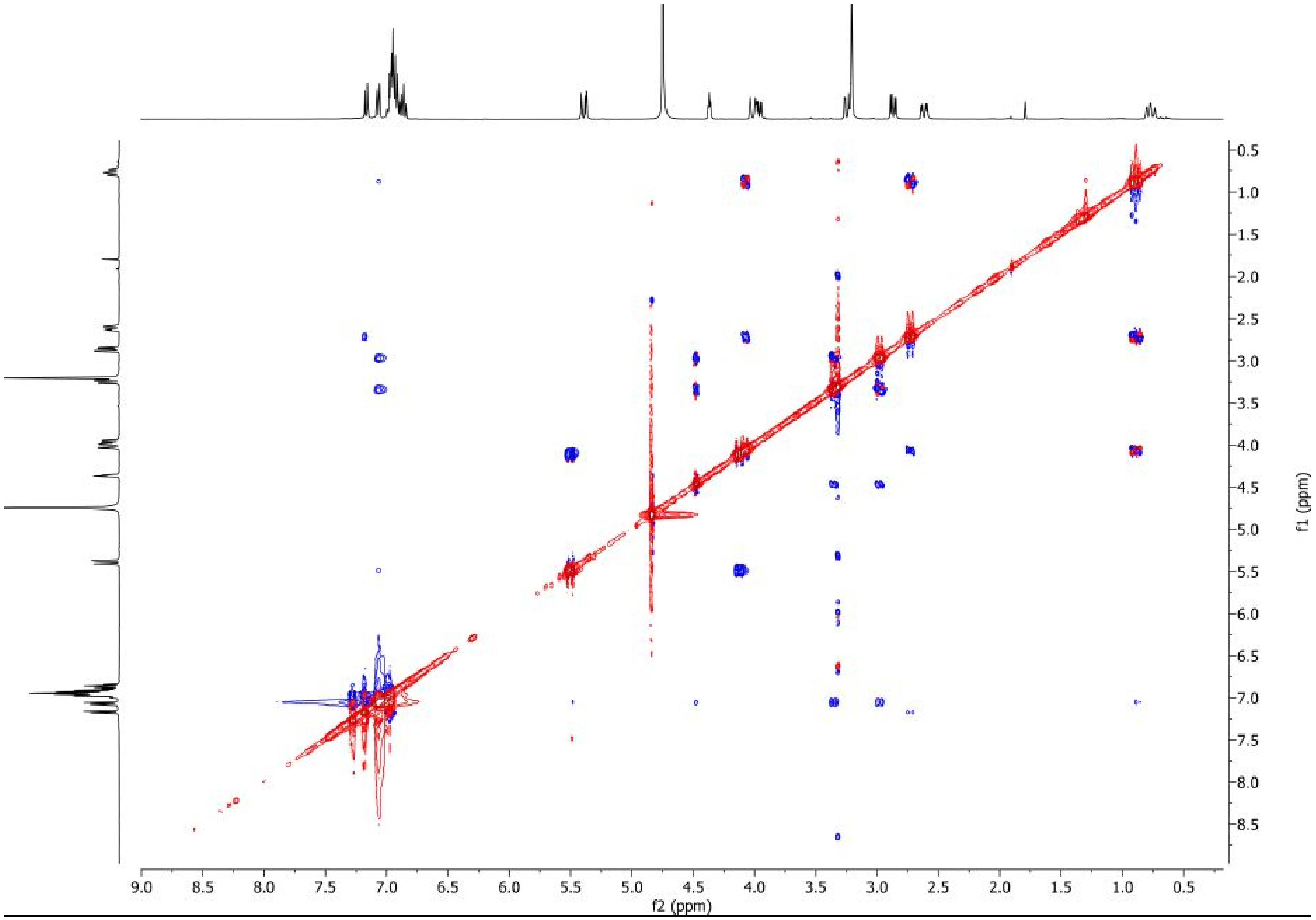

Figure S31: ROESY spectra of **22**

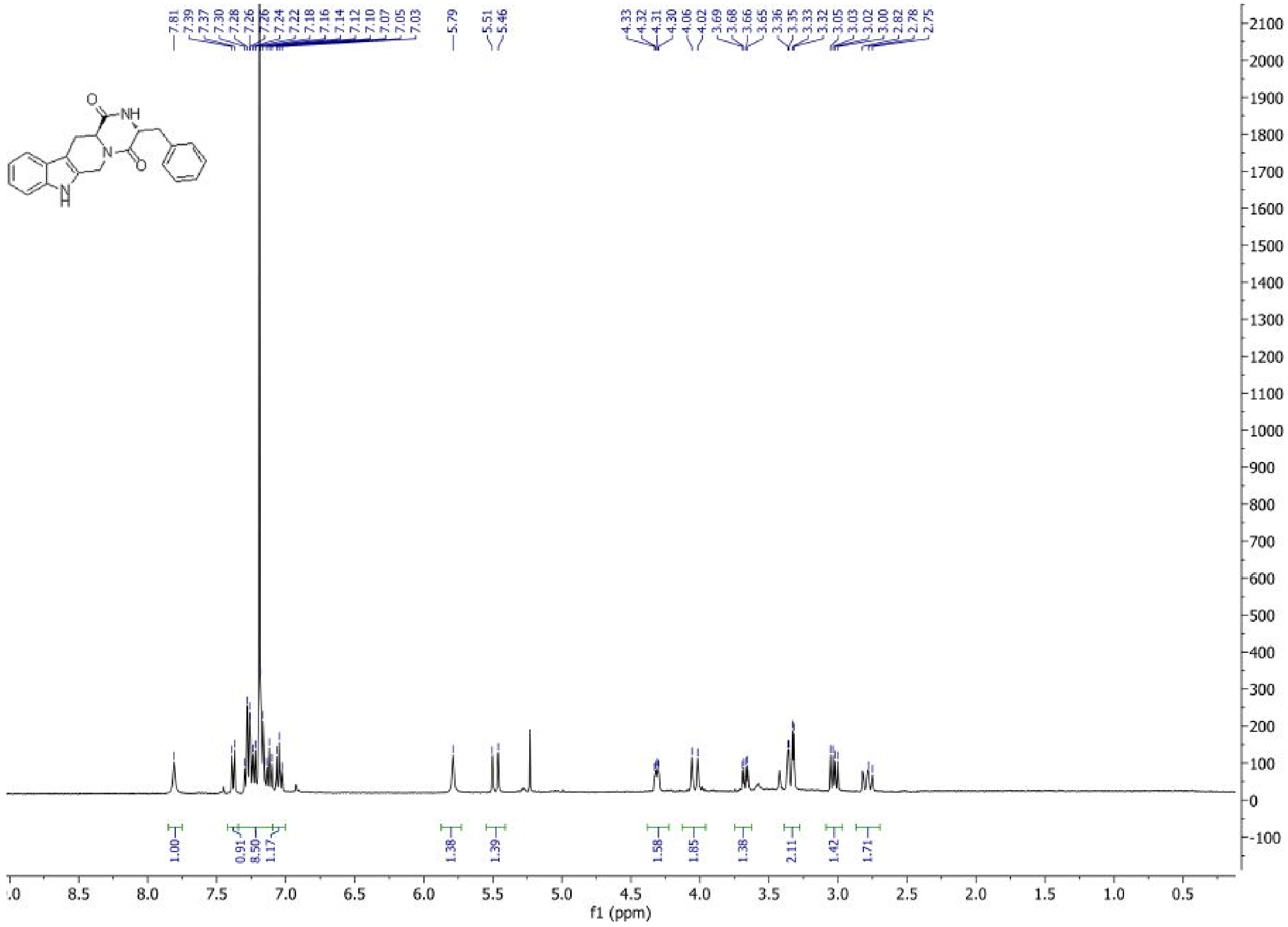

Figure S32: ^{1}H spectra of **23**

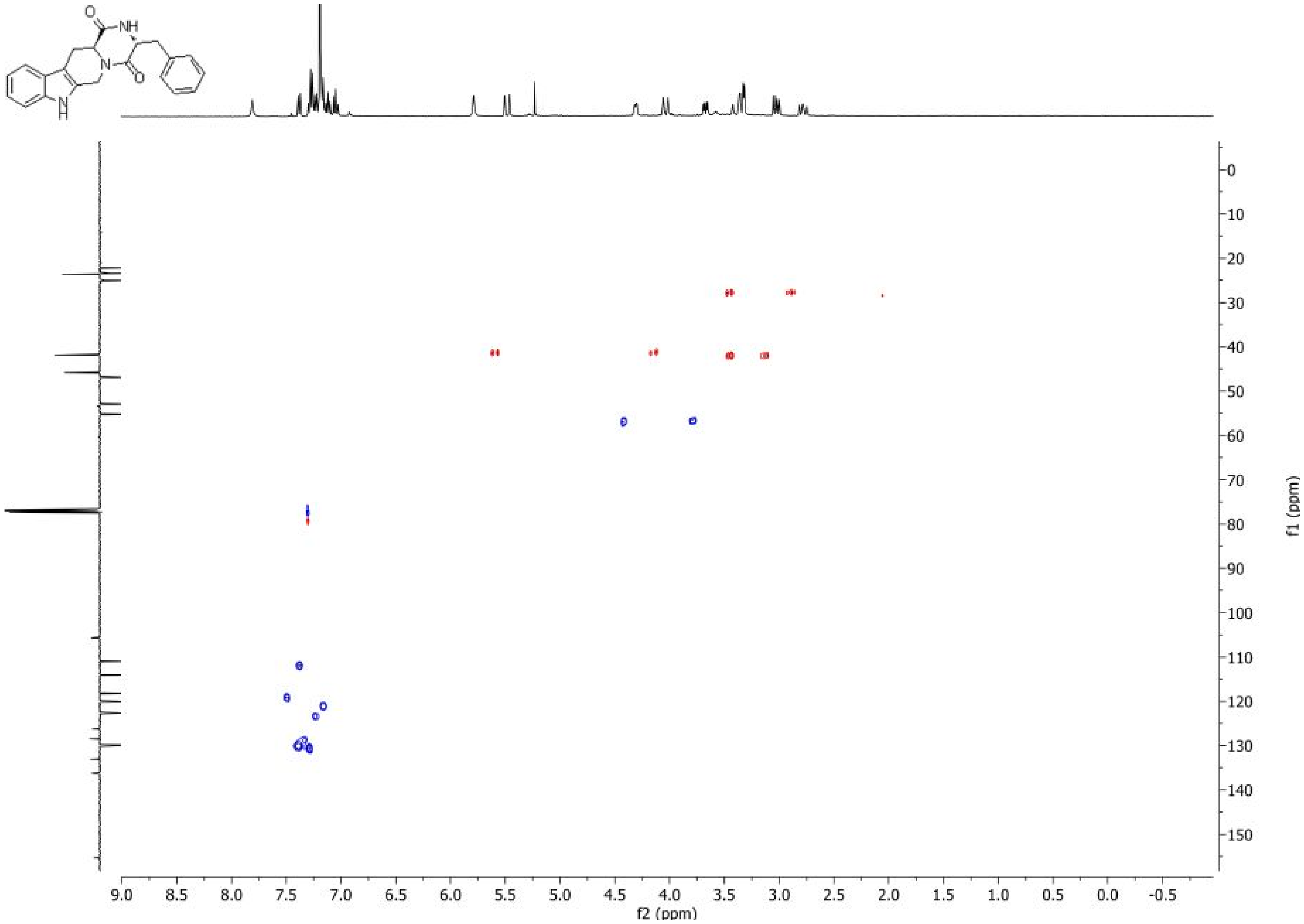

Figure S33: HMBC spectra of **23**

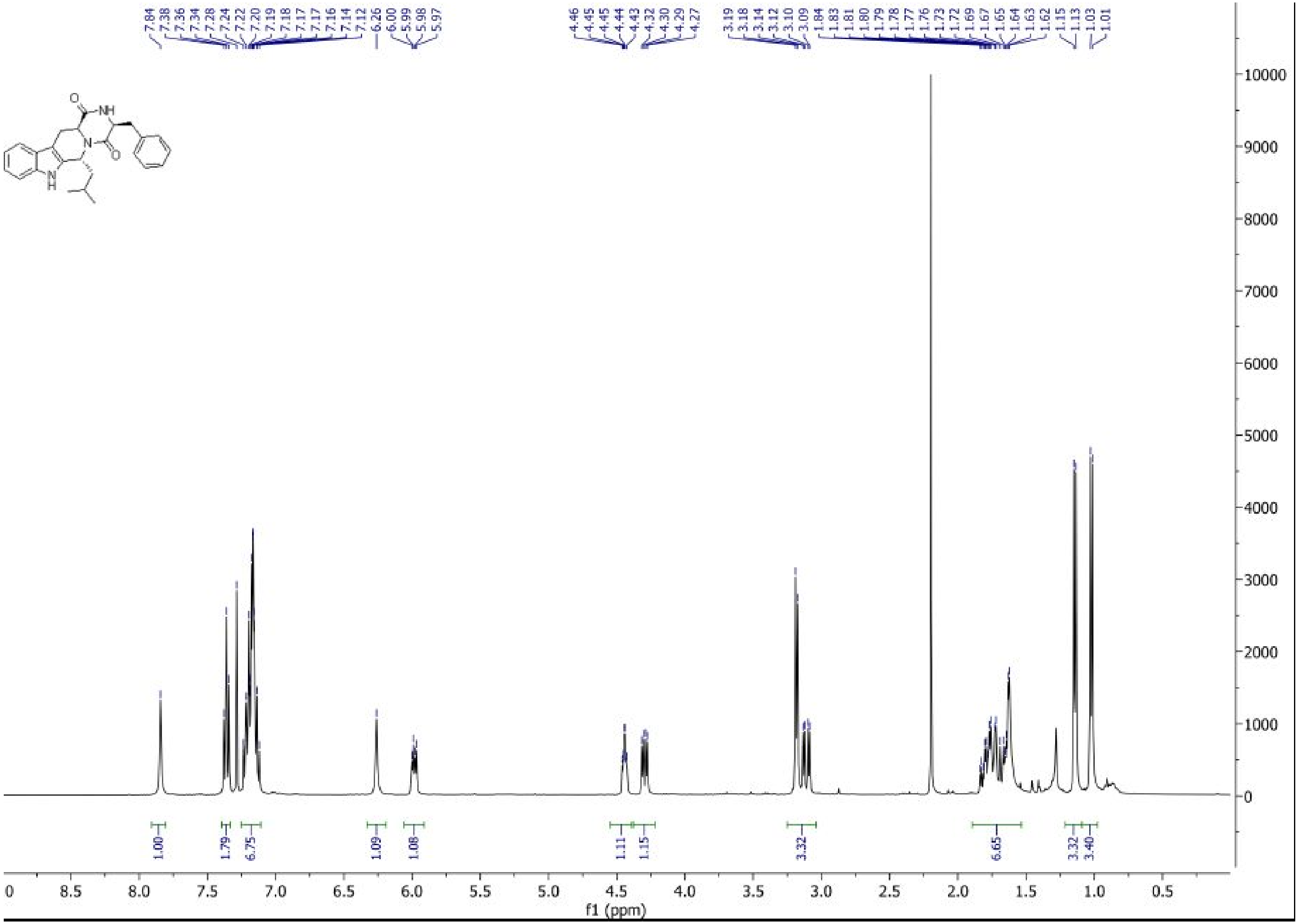

Figure S34: ^{1}H spectra of **25a**

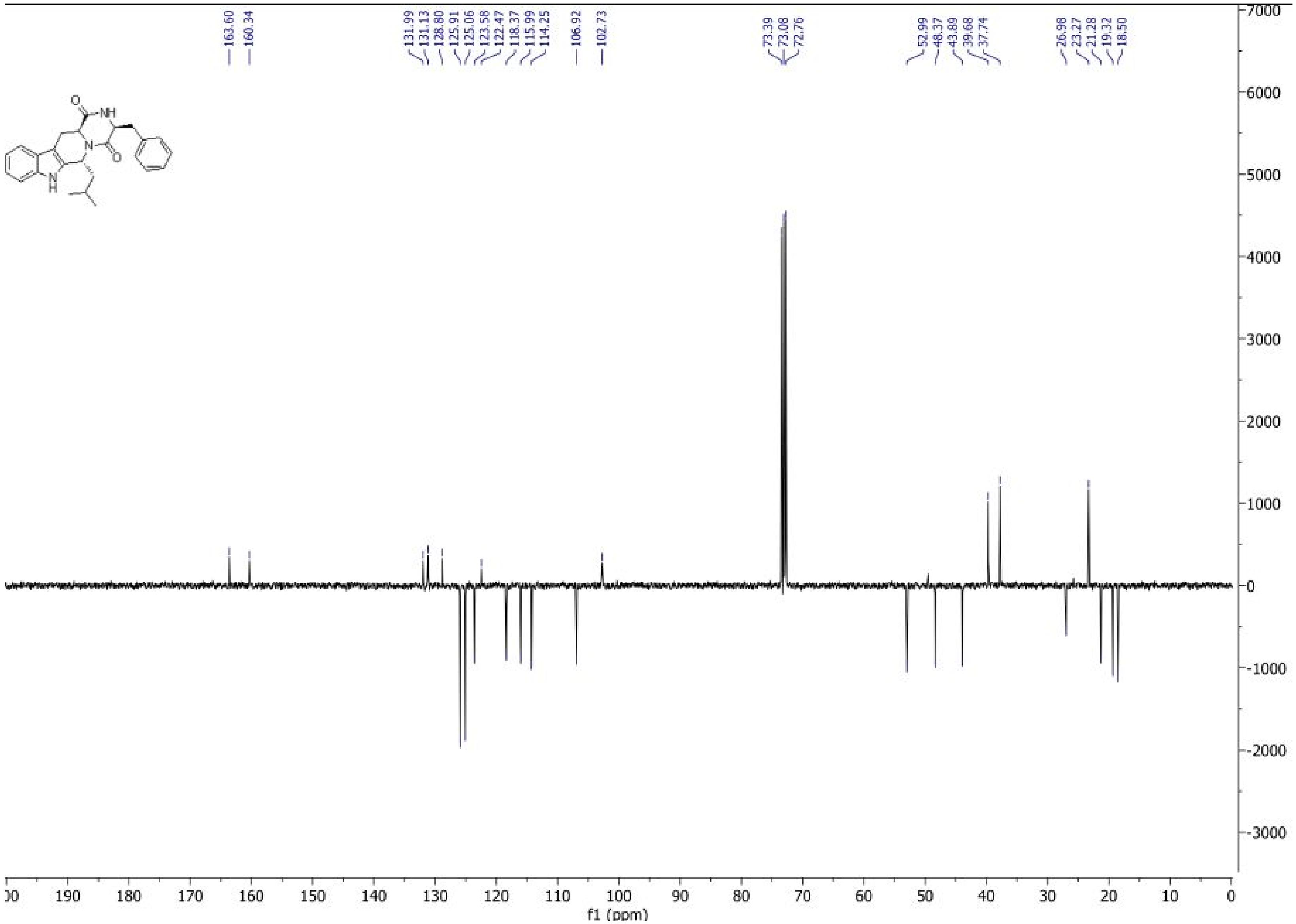

Figure S35: qDEPT spectra of **25a**

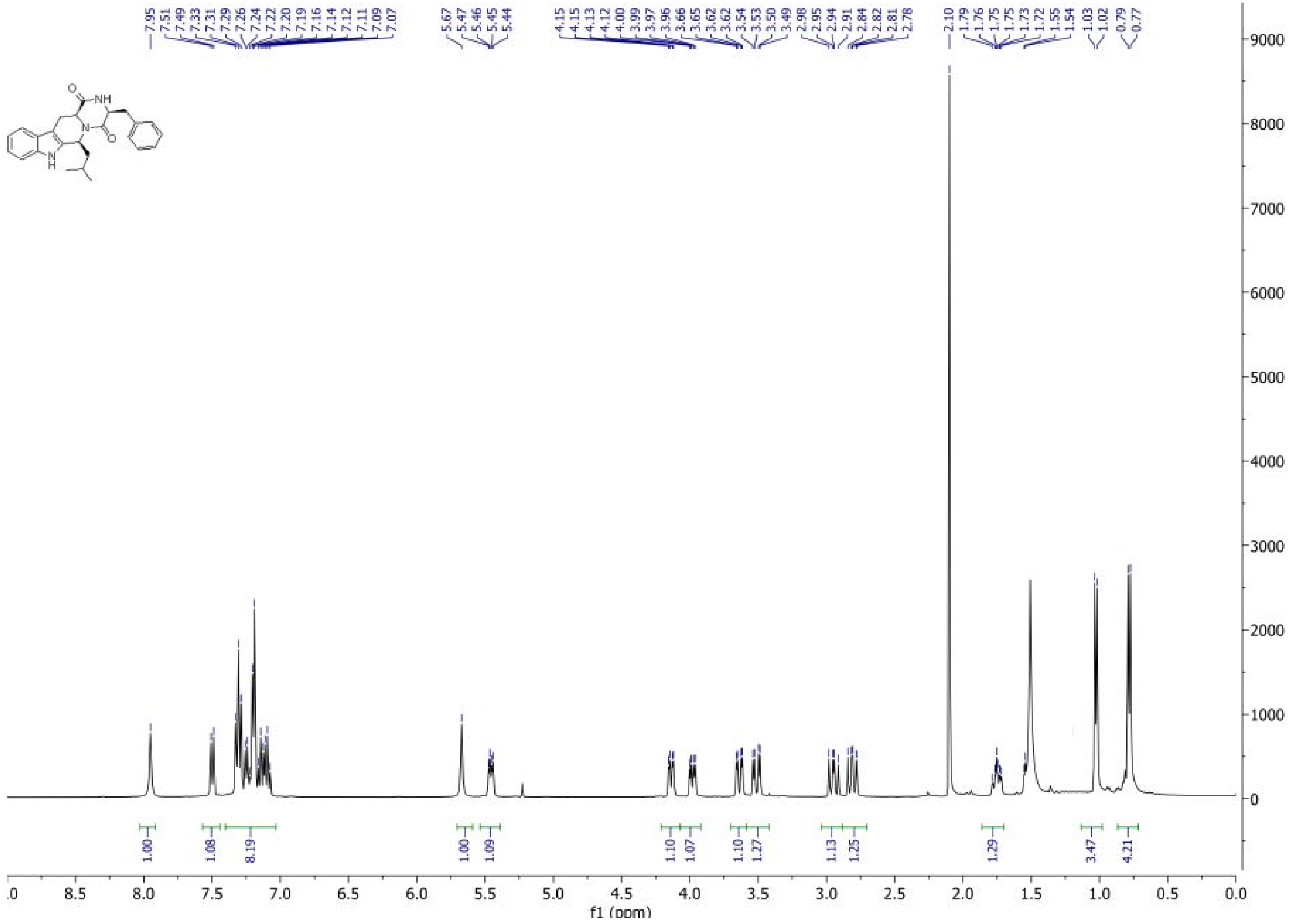

Figure S36: ¹H spectra of **25b**

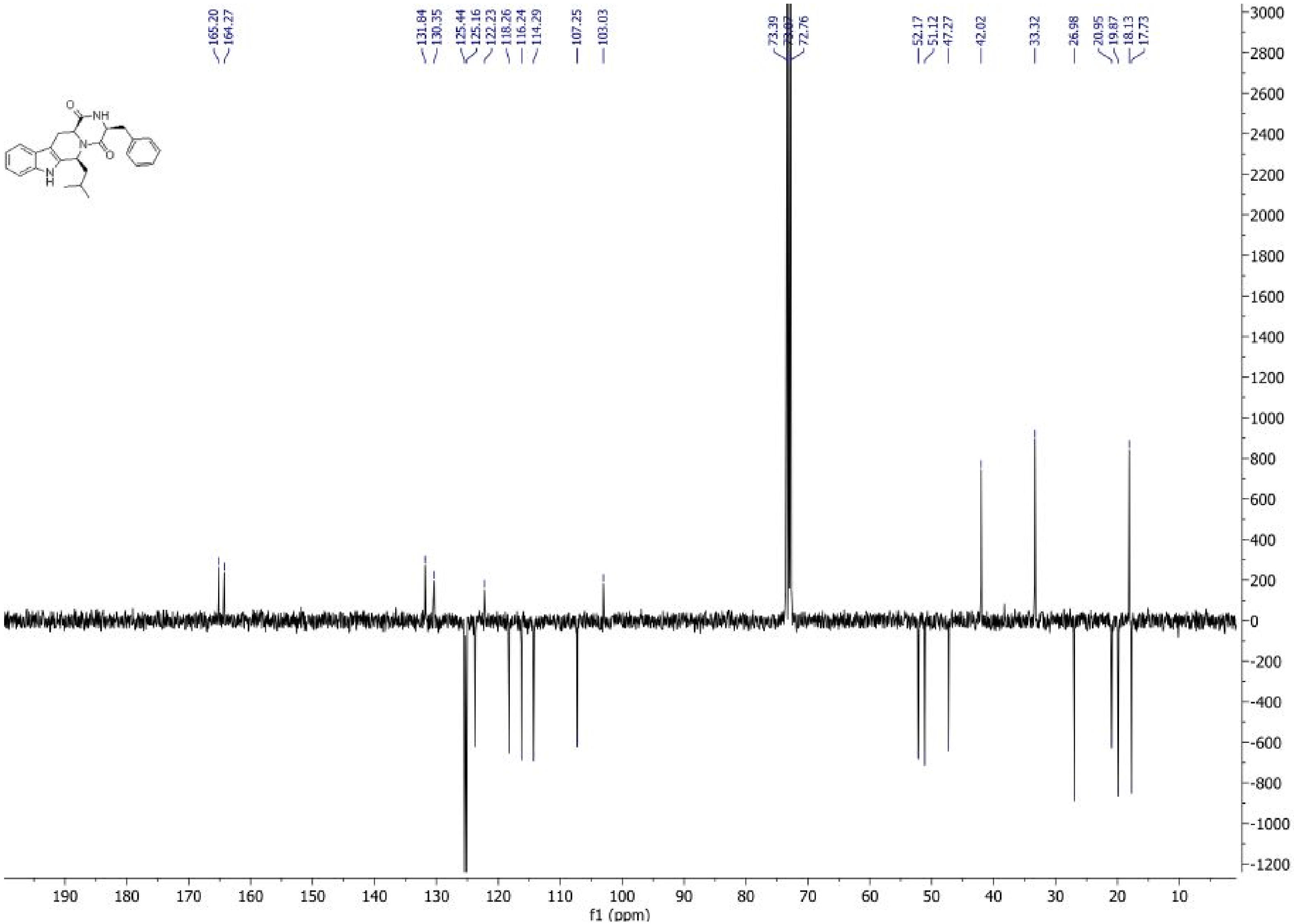

Figure S37: qDEPT spectra of **25b**

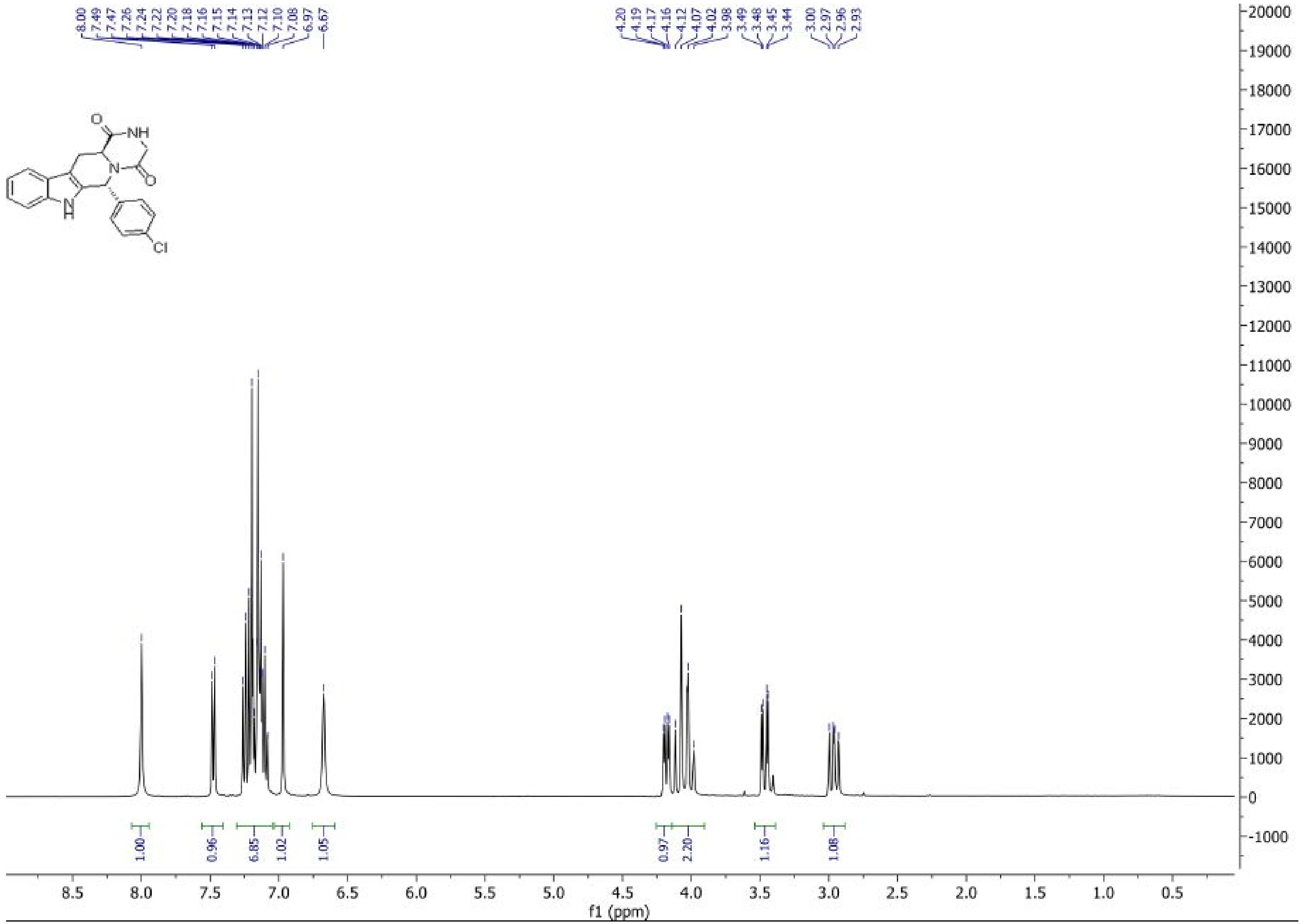

Figure S38: ^{1}H spectra of **27a**

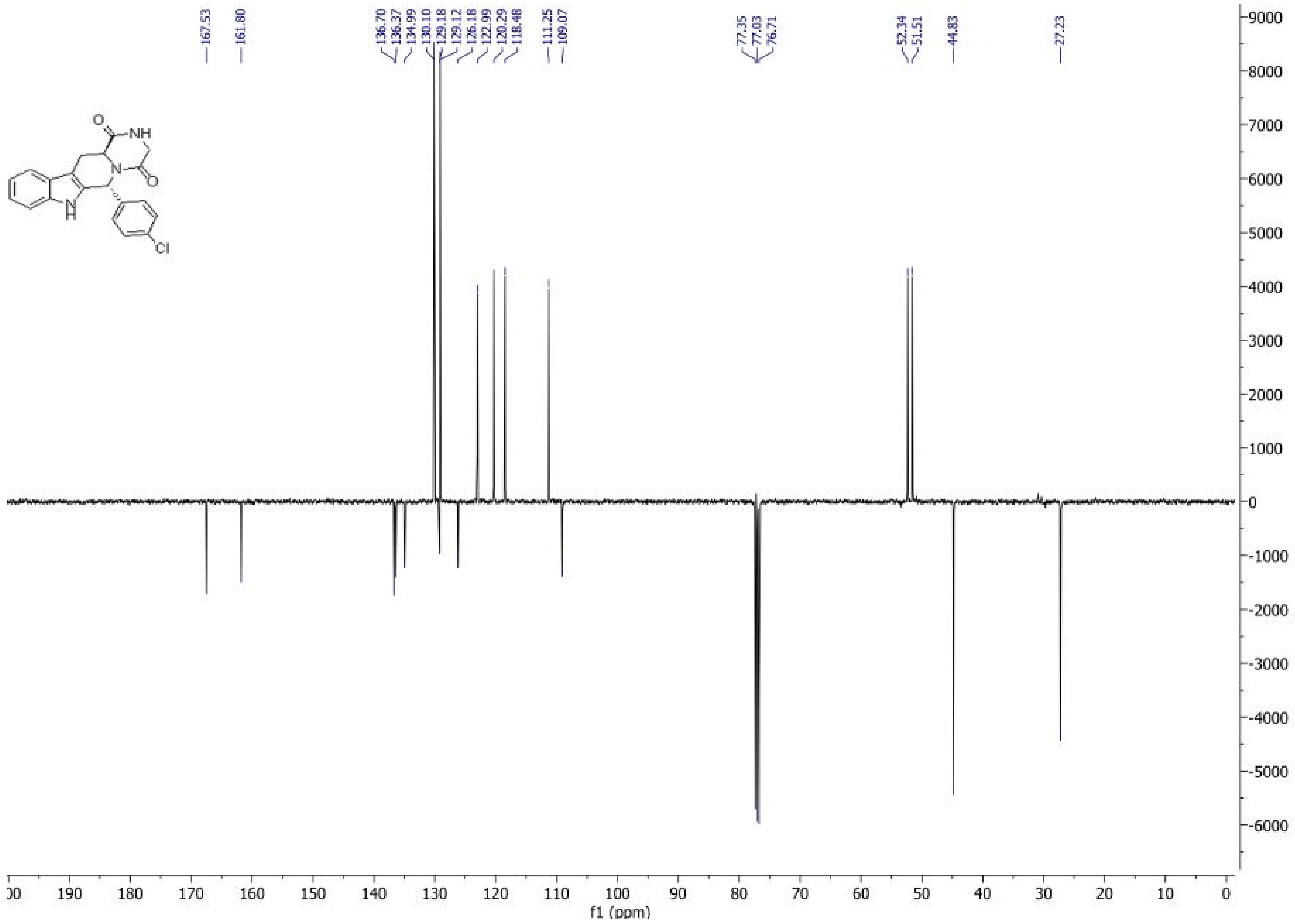

Figure S39: qDEPT spectra of **27a**

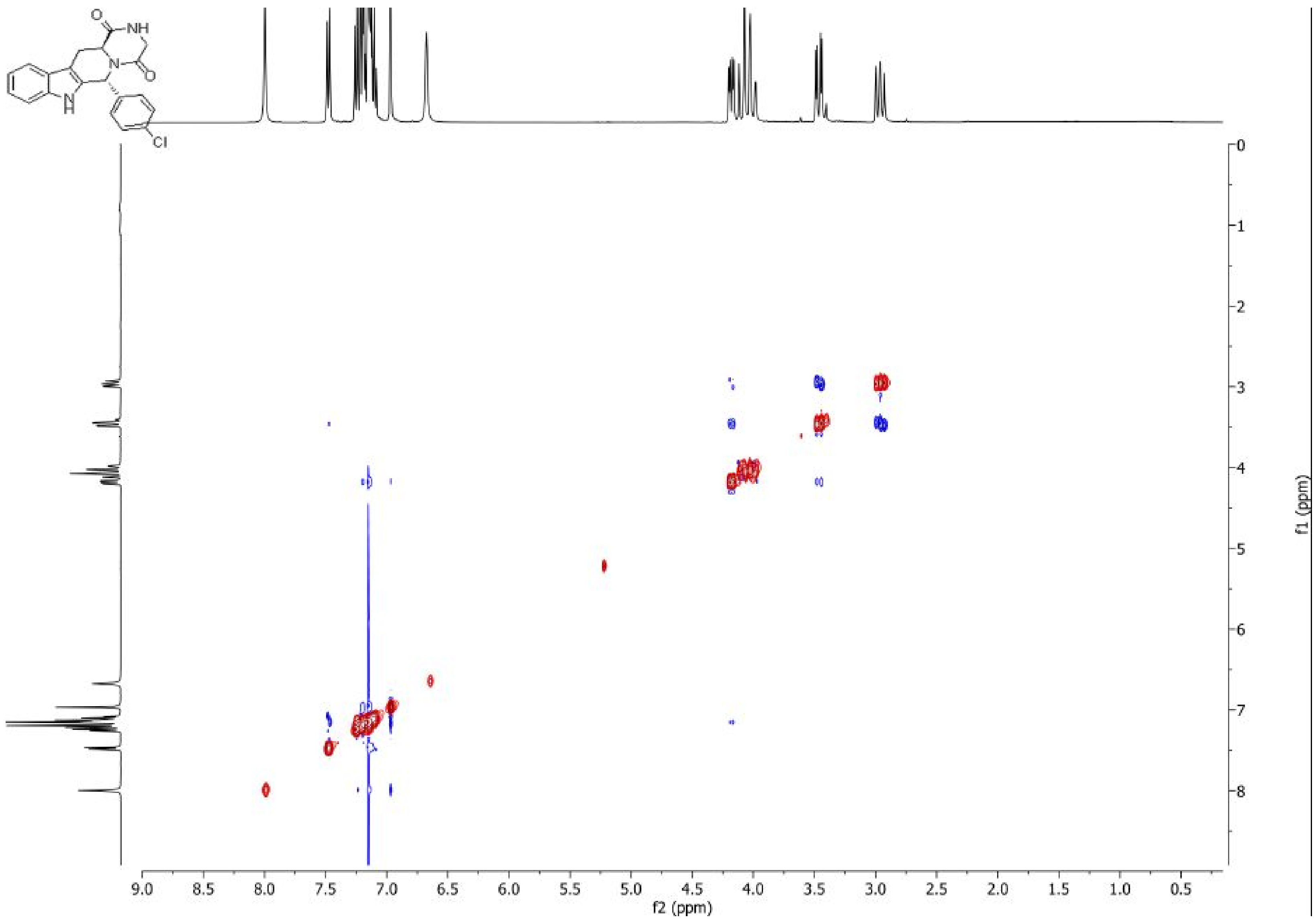

Figure S40: ROESY spectra of **27a**

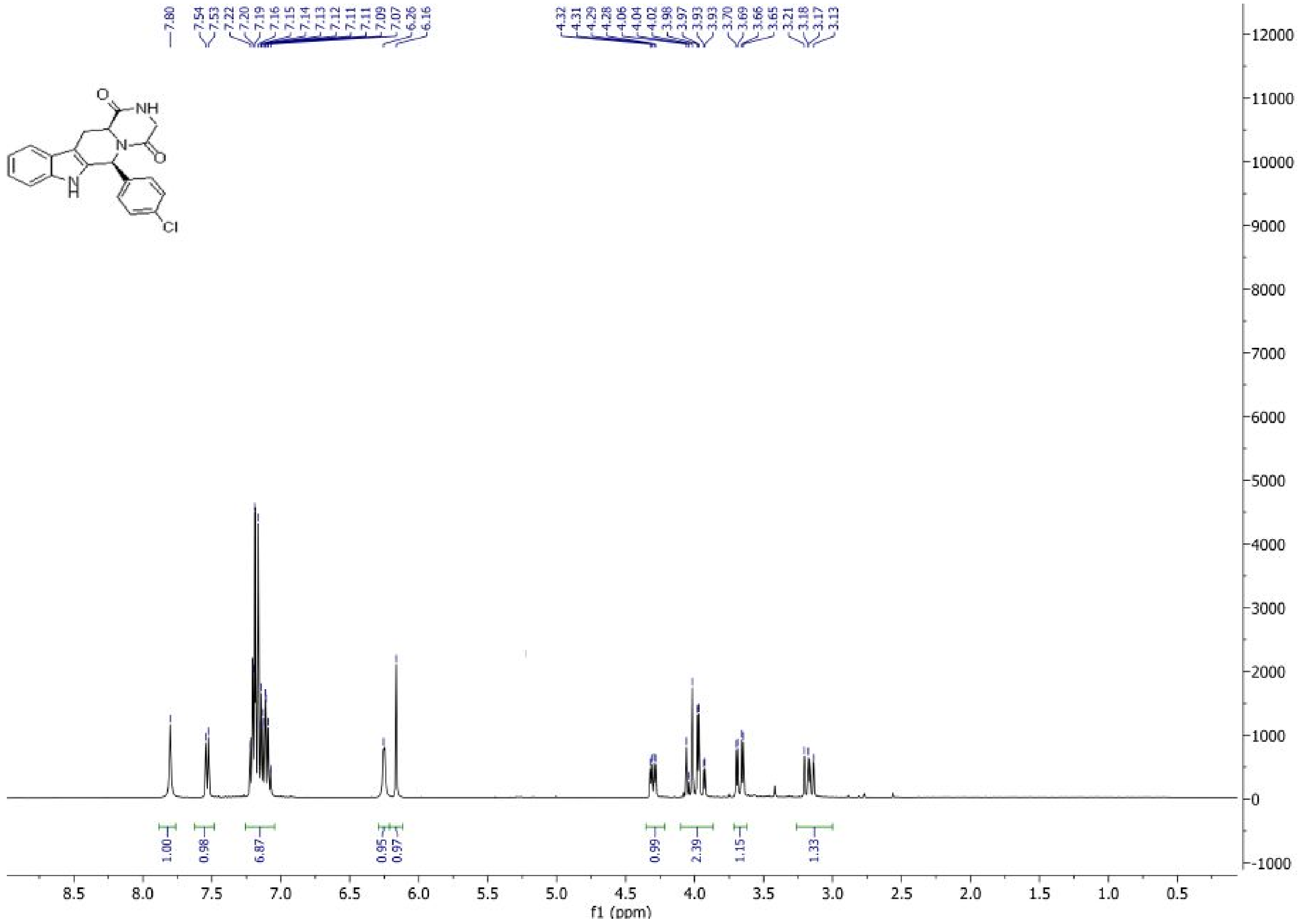

Figure S41: ^{1}H spectra of **27b**

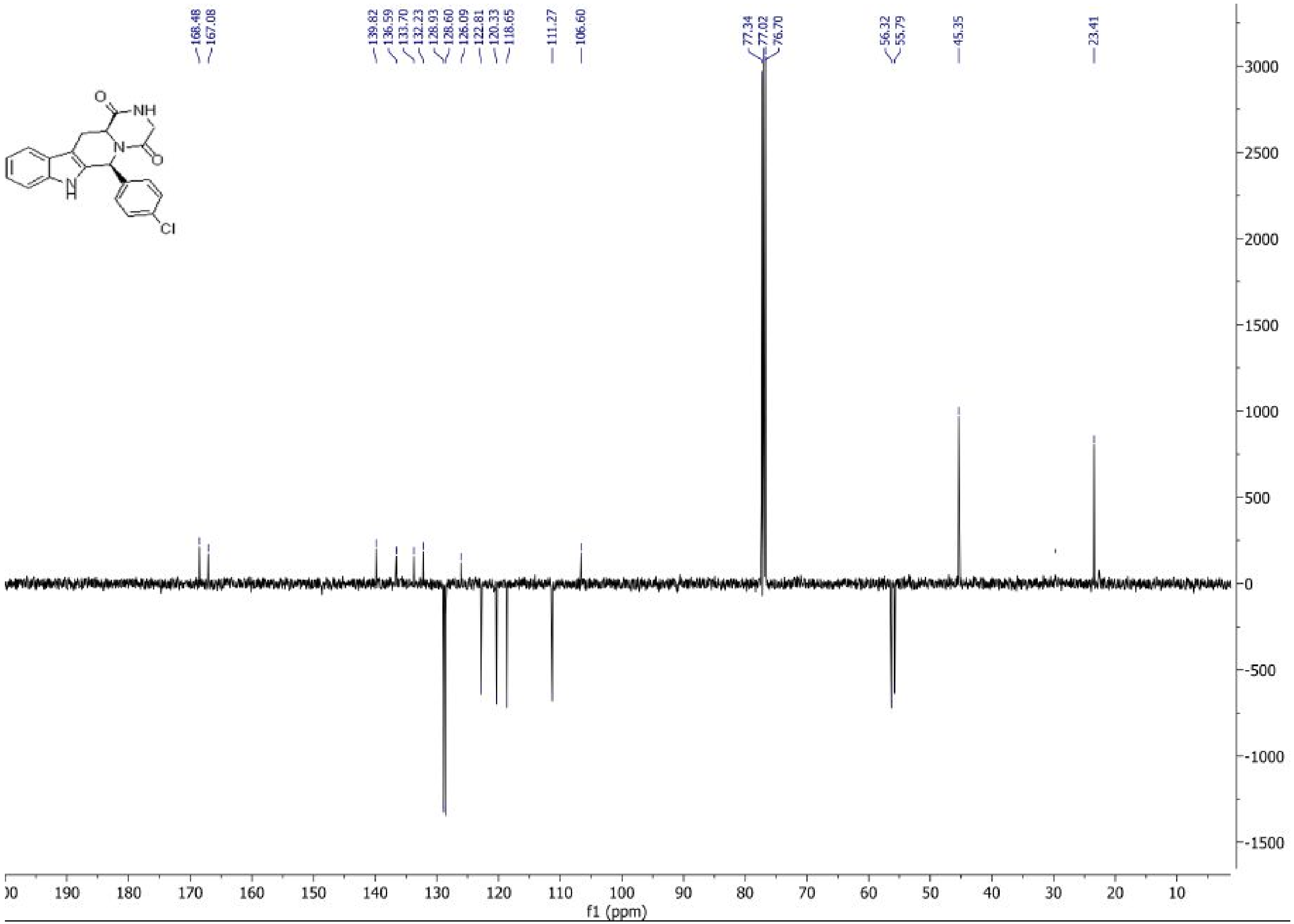

Figure S42: qDEPT spectra of **27b**

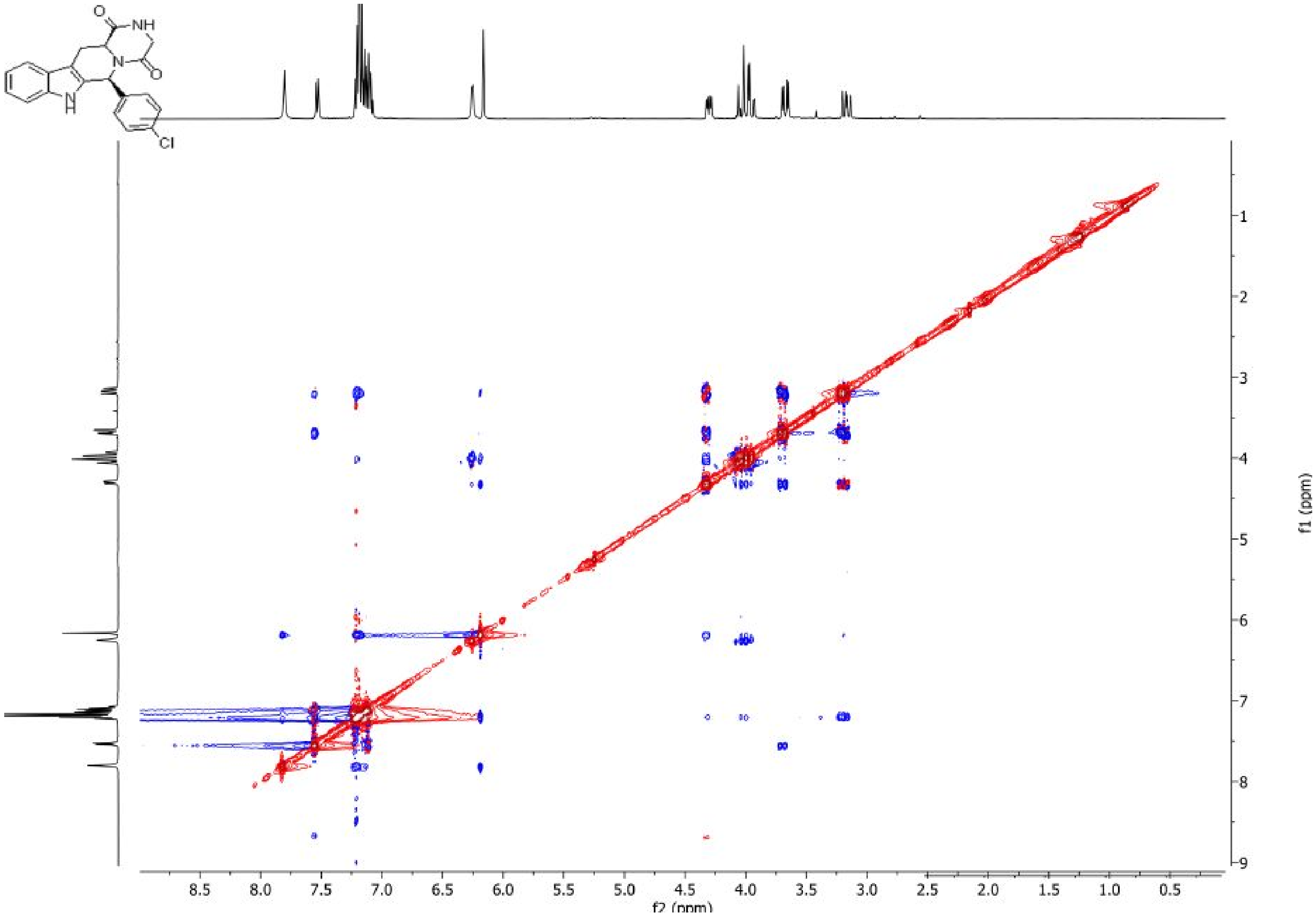

Figure S43: ROESY spectra of **27b**

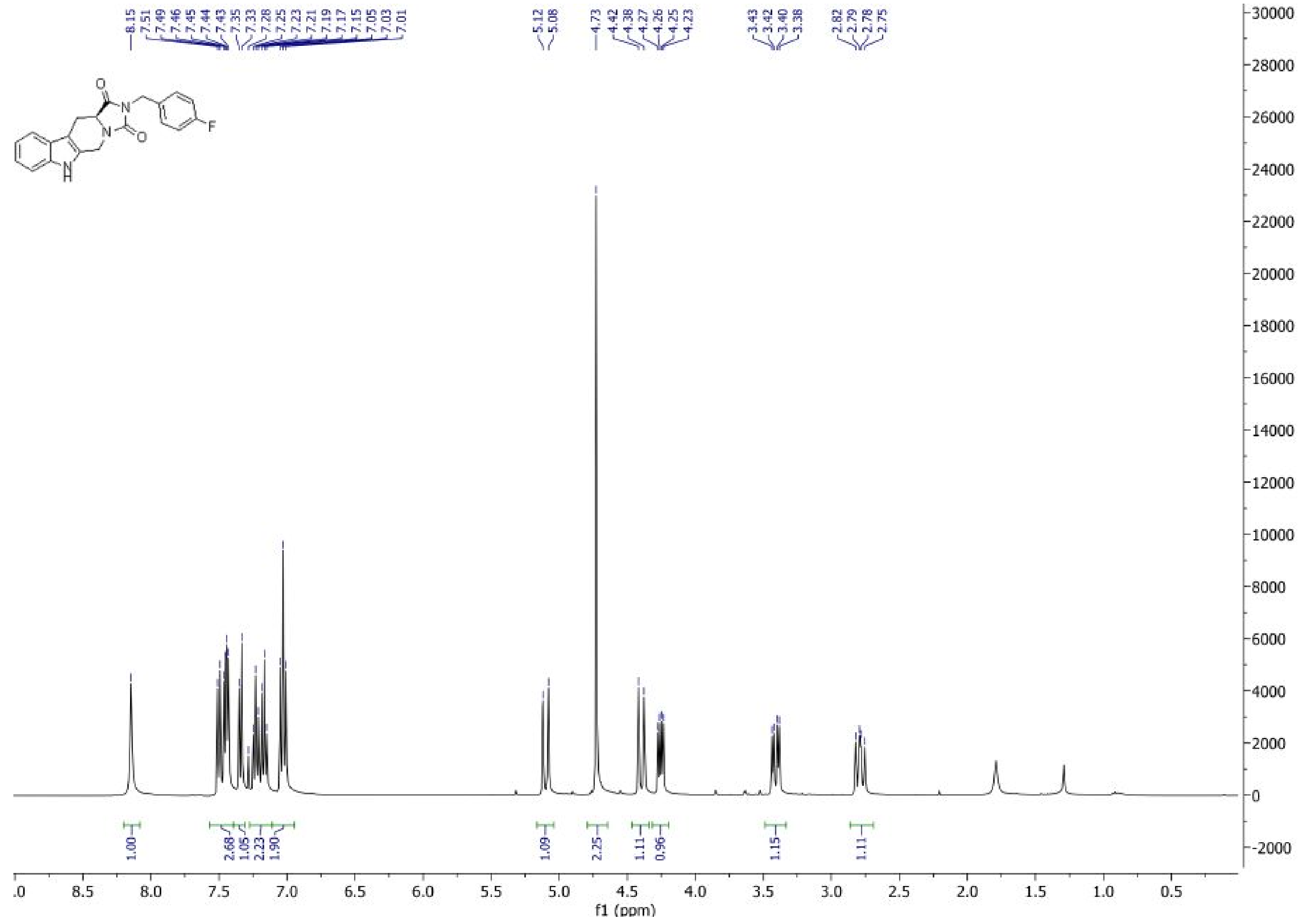

Figure S44: ^{1}H spectra of **28**

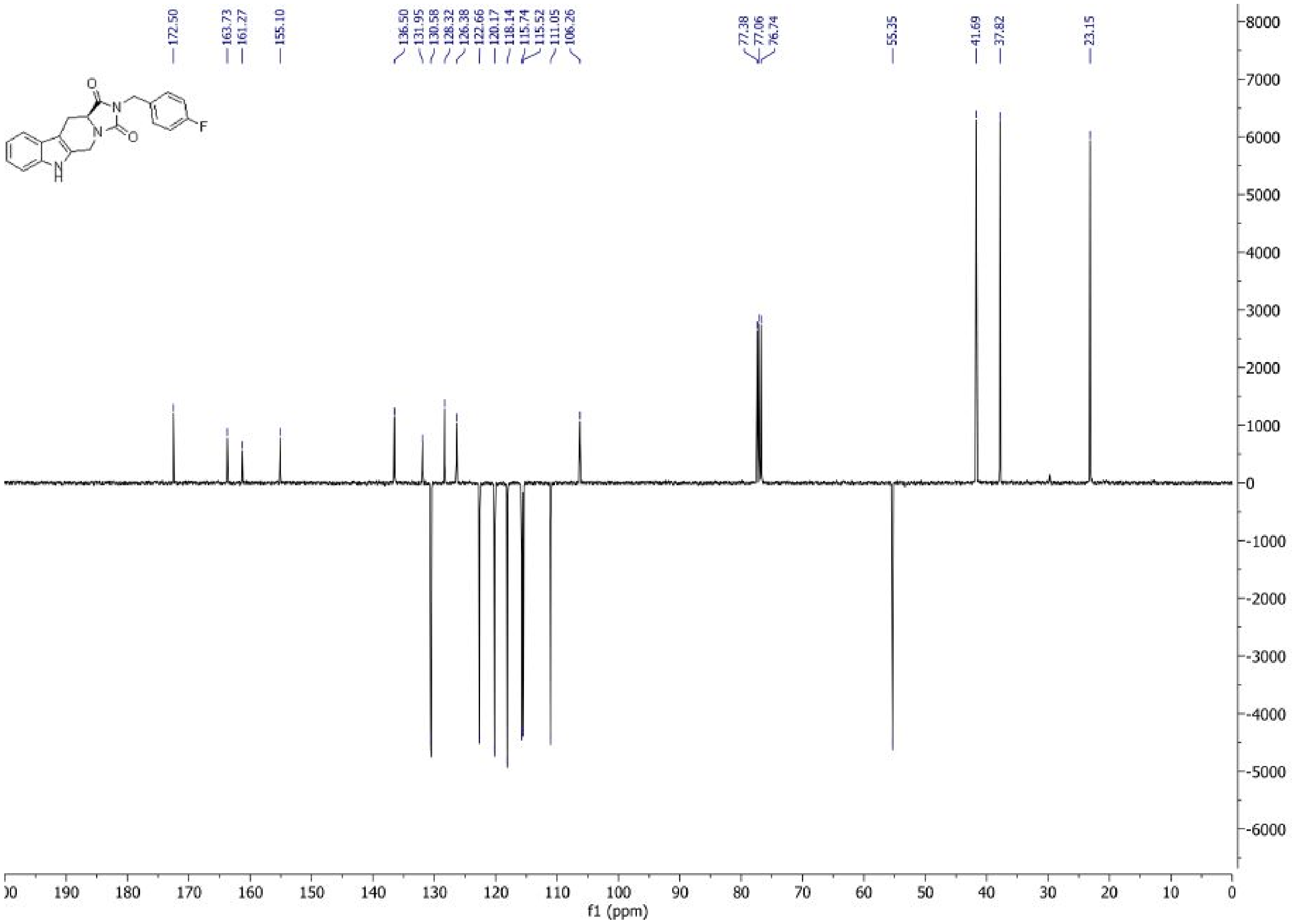

Figure S45: qDEPT spectra of **28**

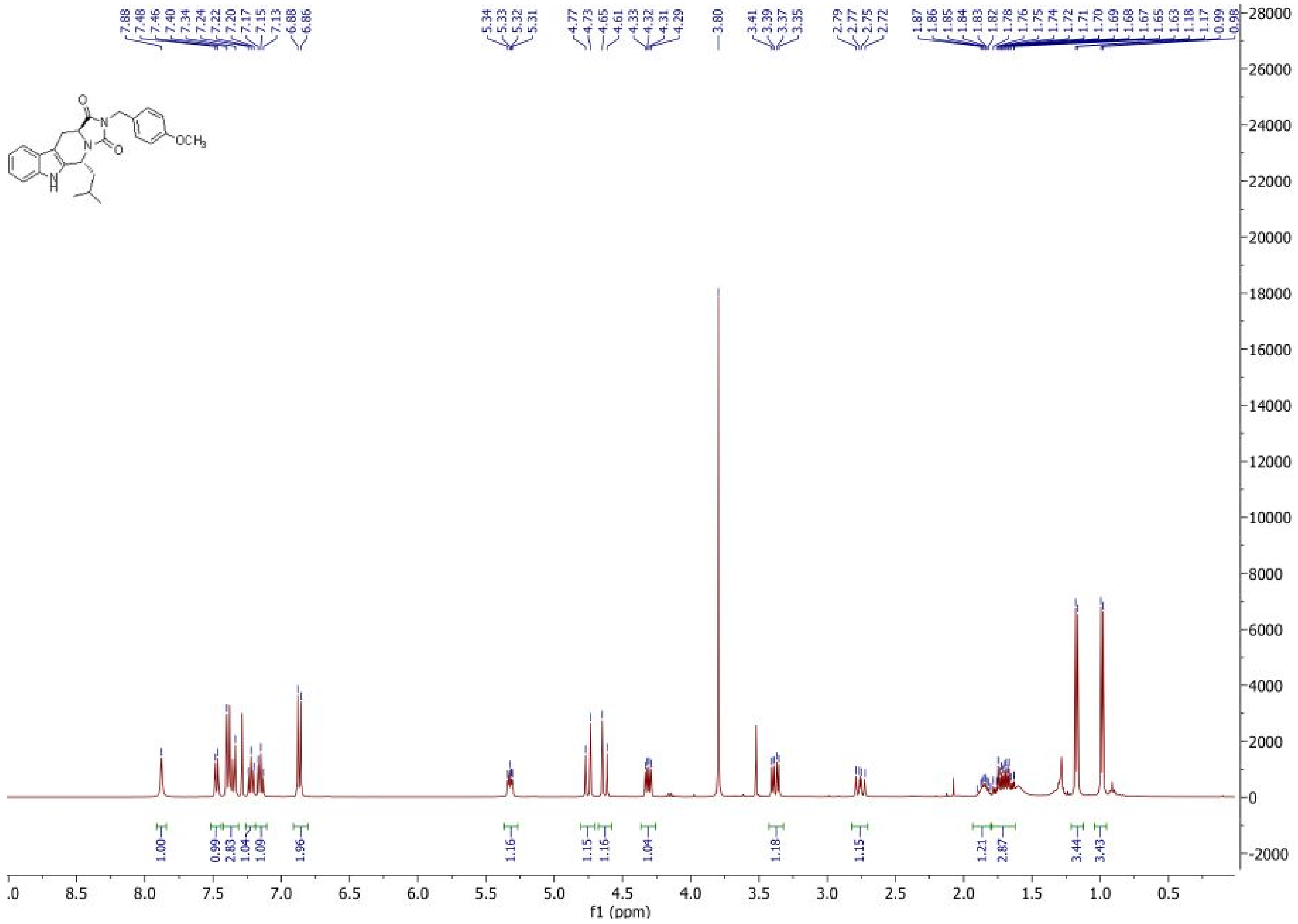

Figure S46: ^{1}H spectra of **29a**

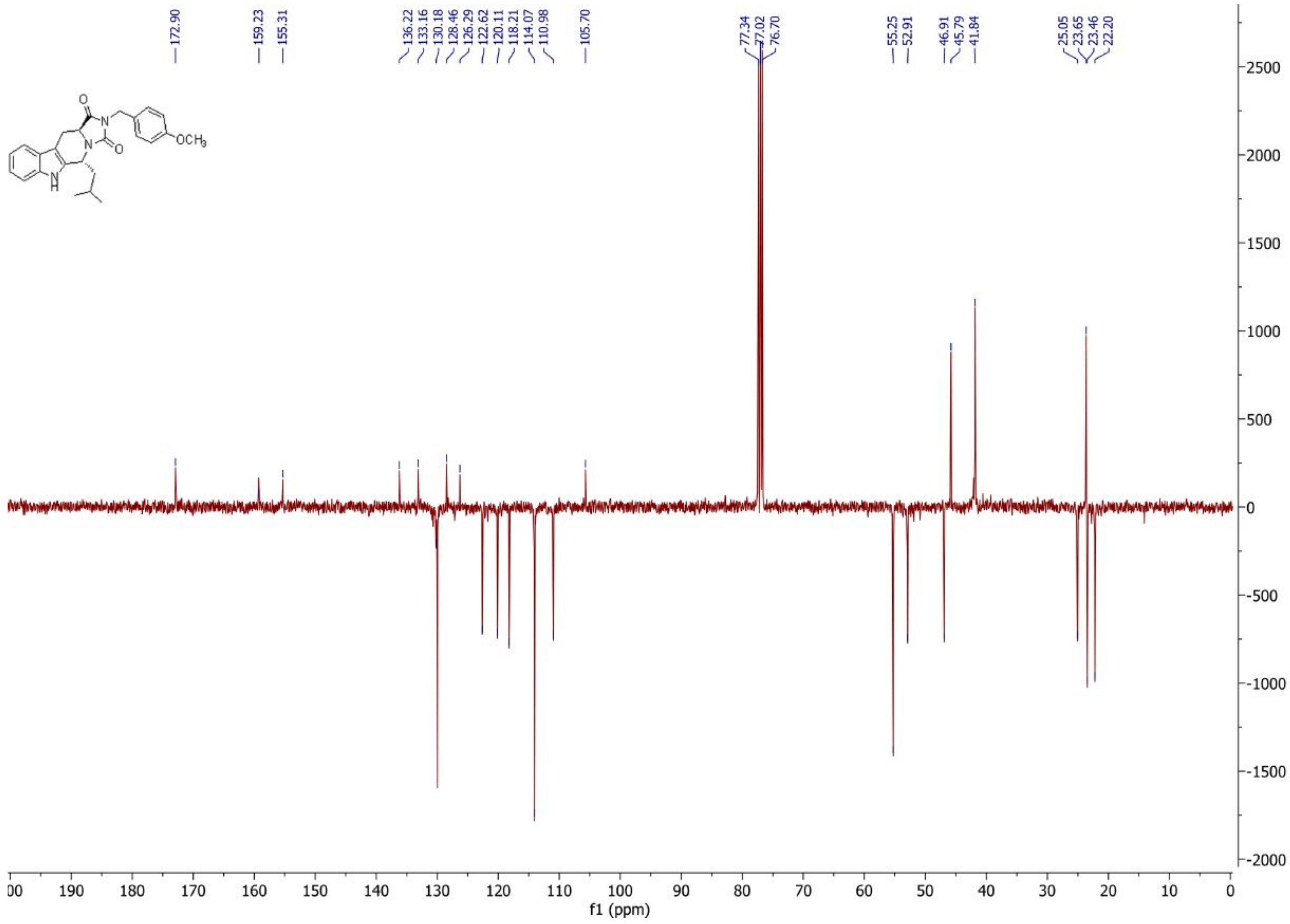

Figure S47: qDEPT spectra of **29a**

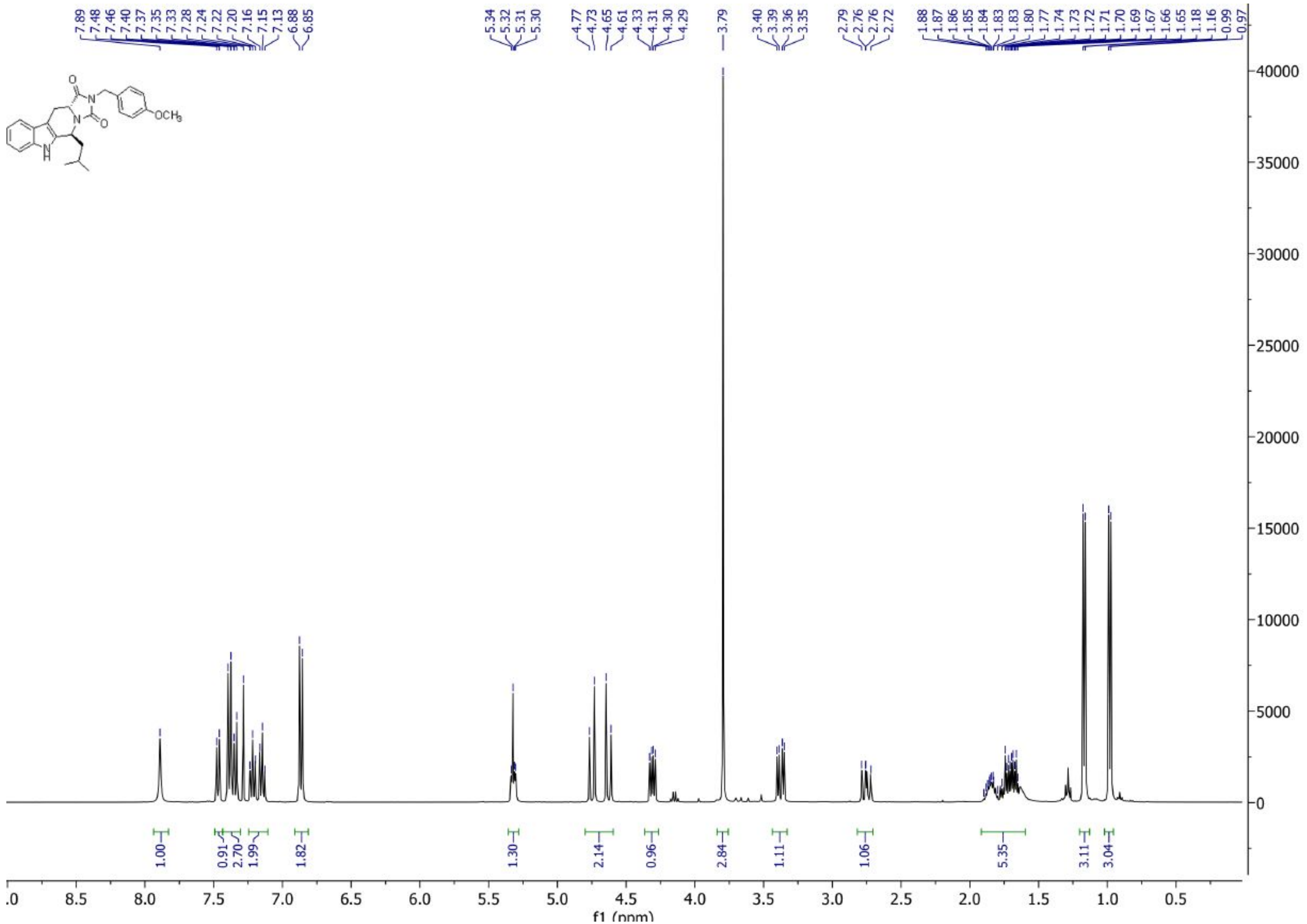

Figure S48: ^{1}H spectra of **29a'**

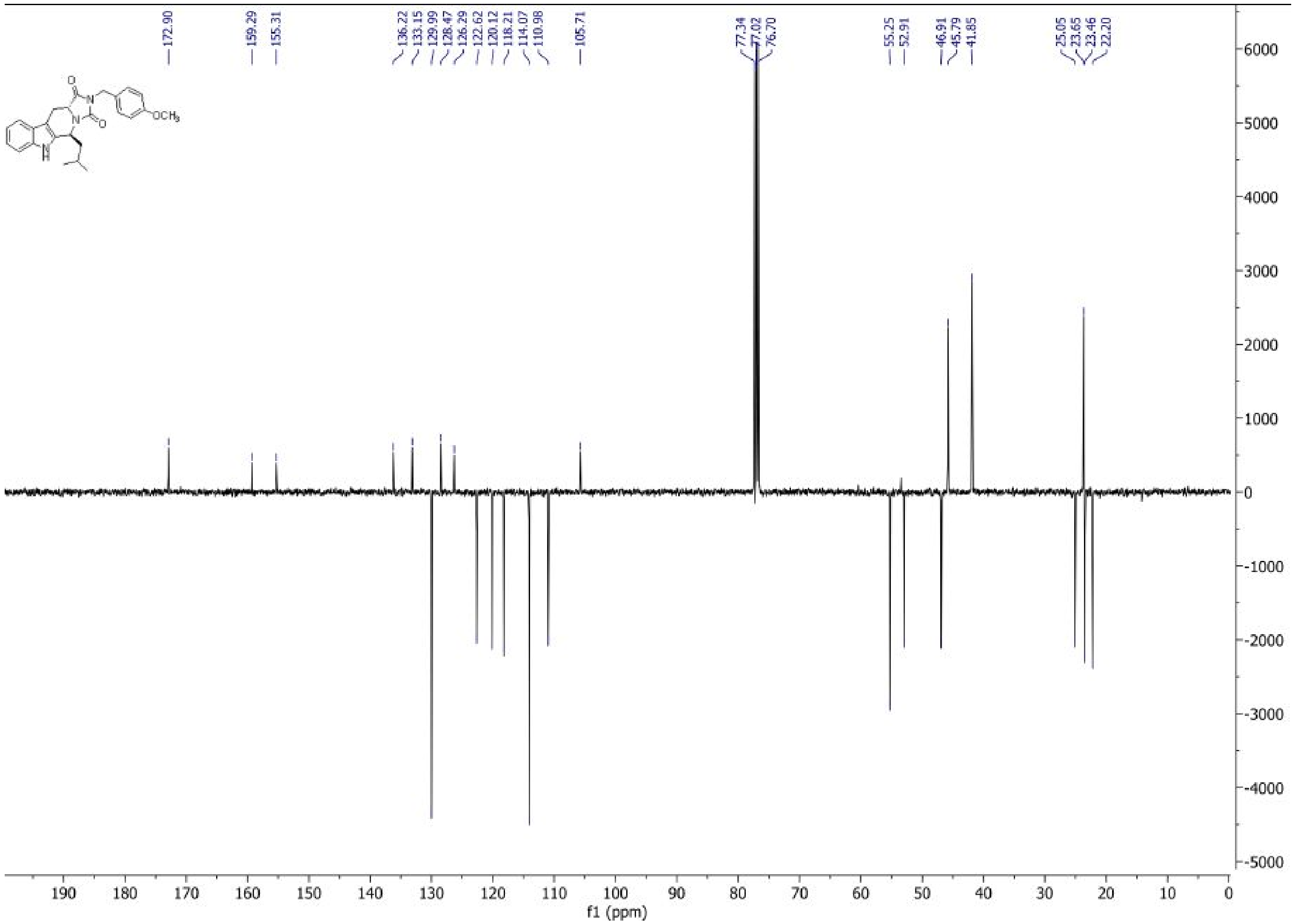

Figure S49: qDEPT spectra of **29a'**

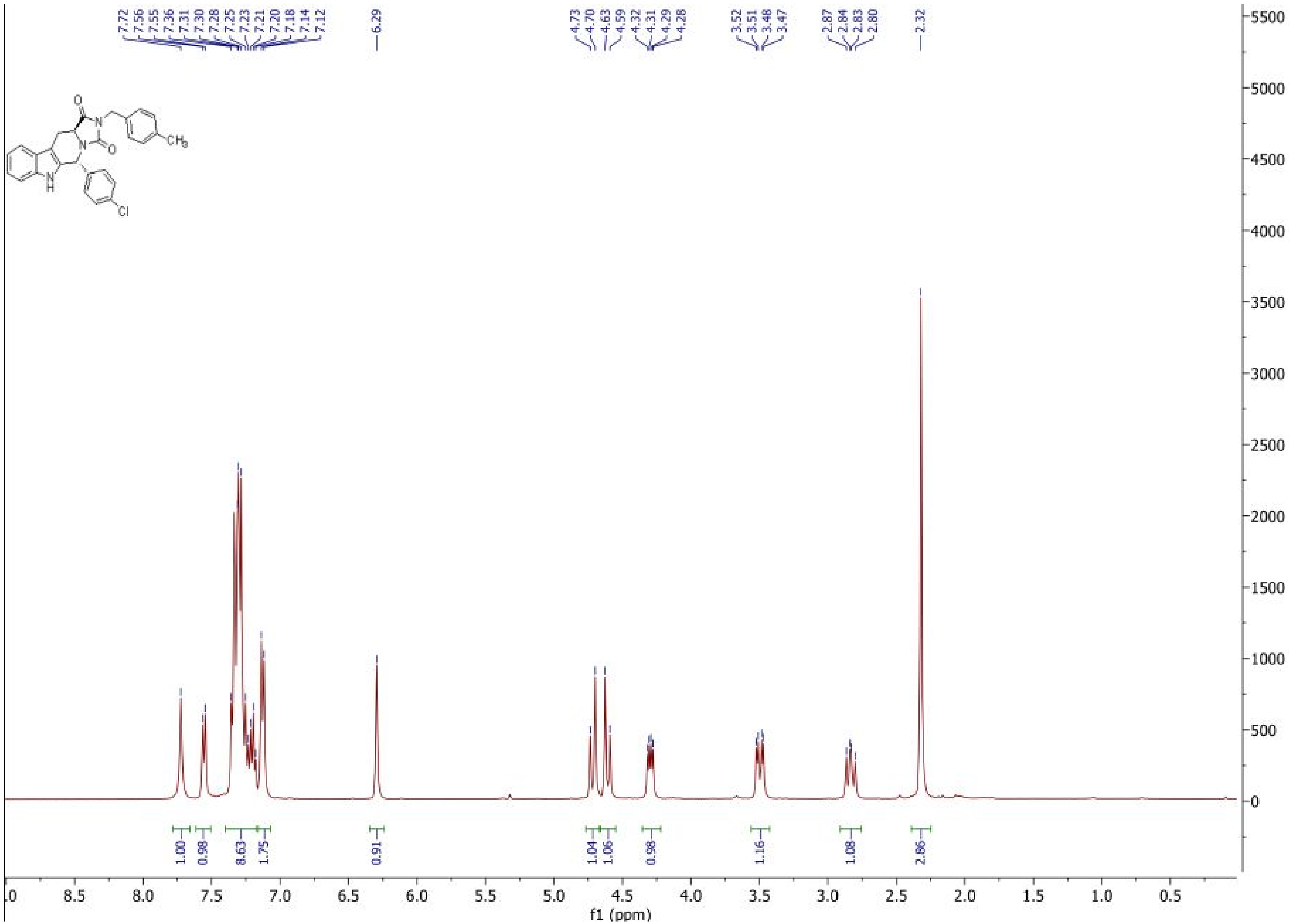

Figure S50: ^{1}H spectra of **30a**

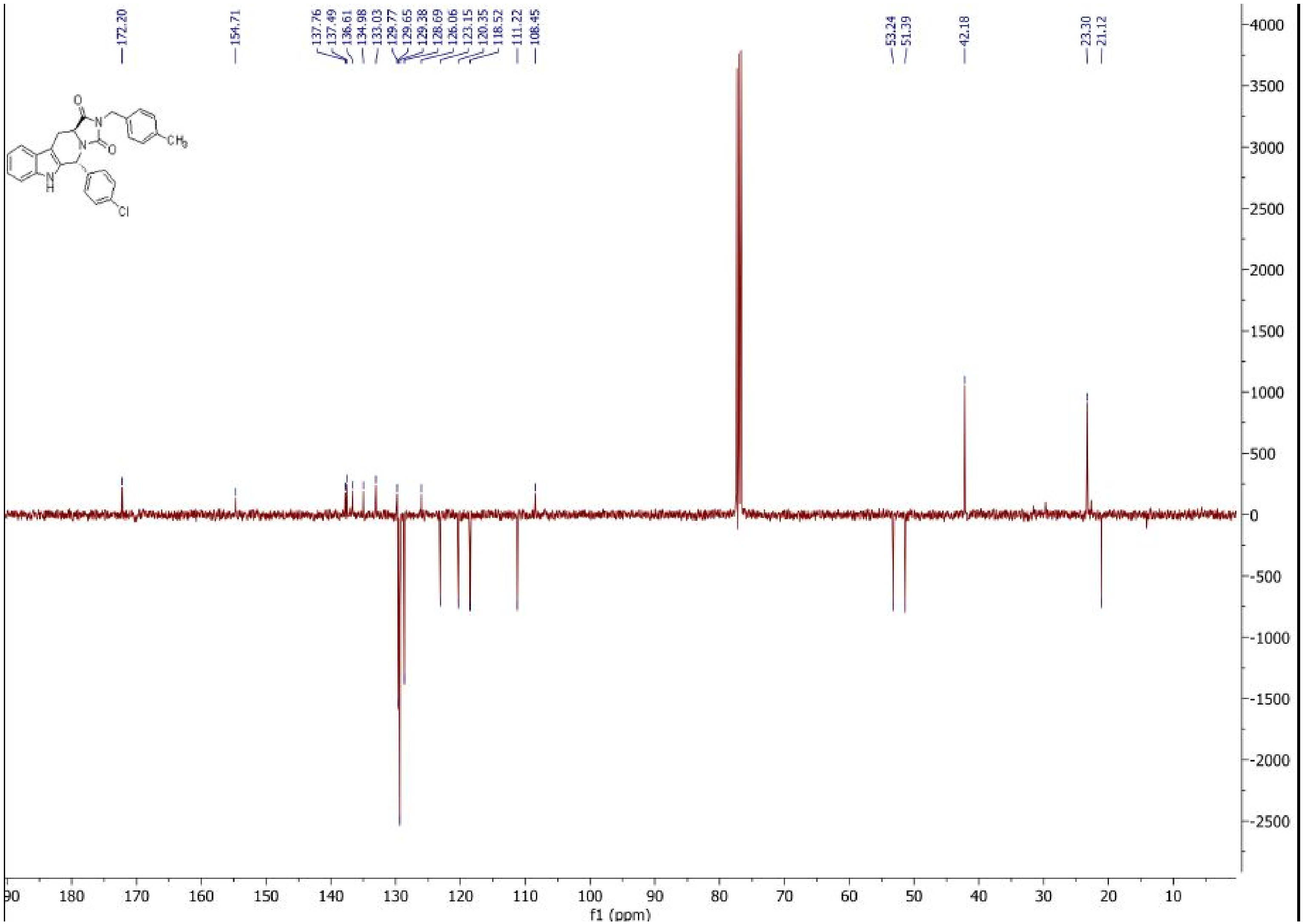

Figure S51: qDEPT spectra of **30a**

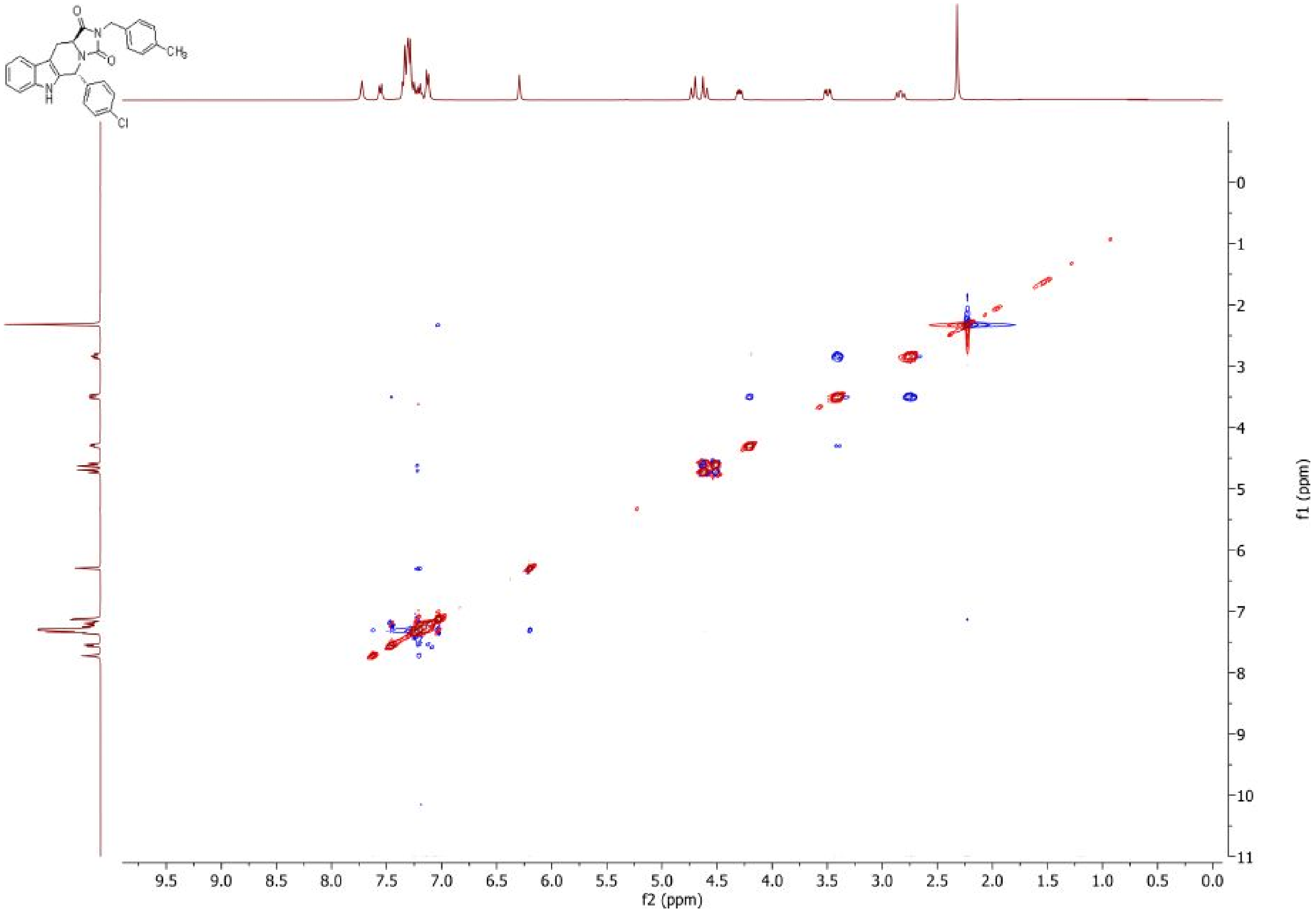

Figure S52: ROESY spectra of **30a**

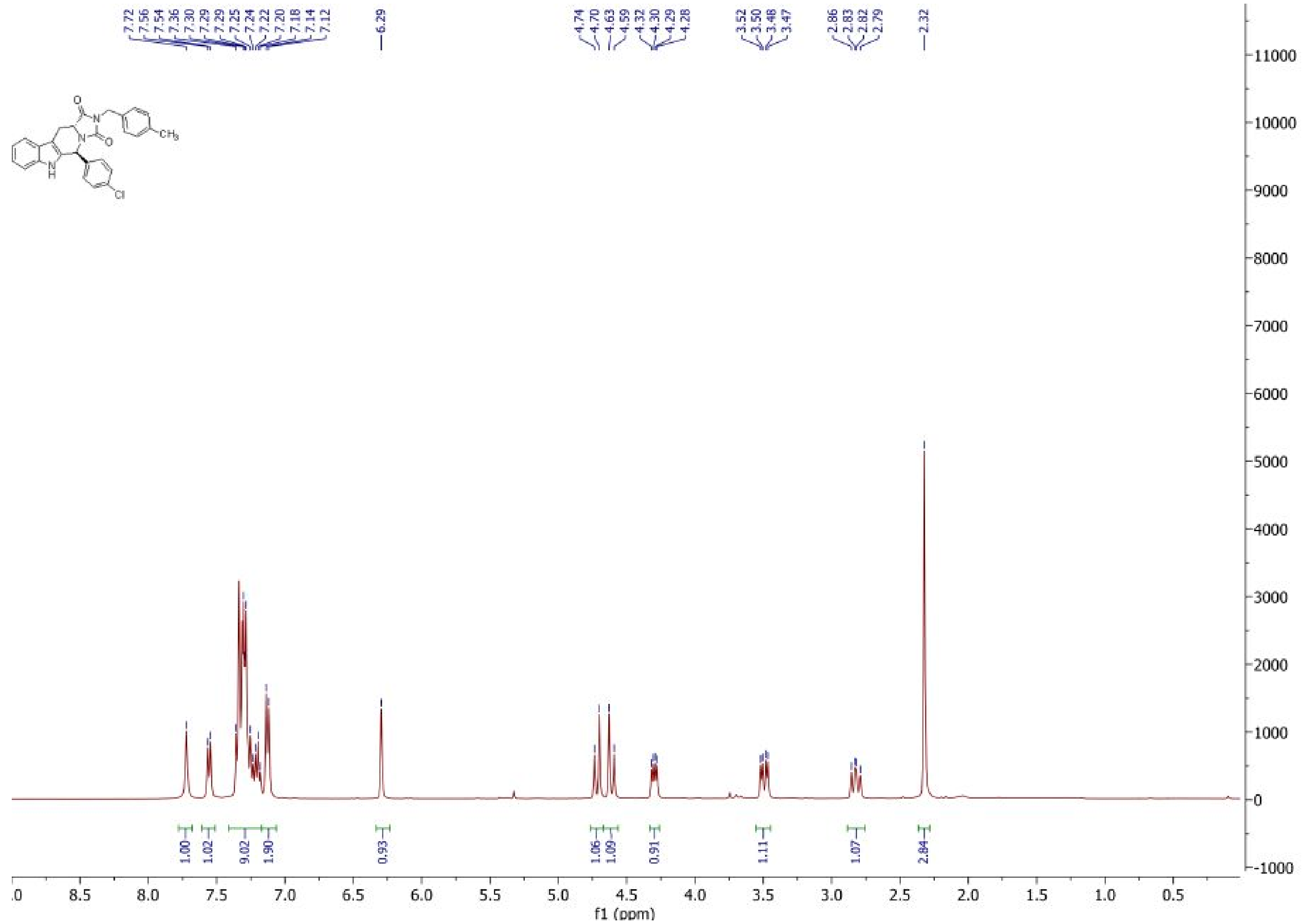

Figure S53: ^{1}H spectra of **30a'**

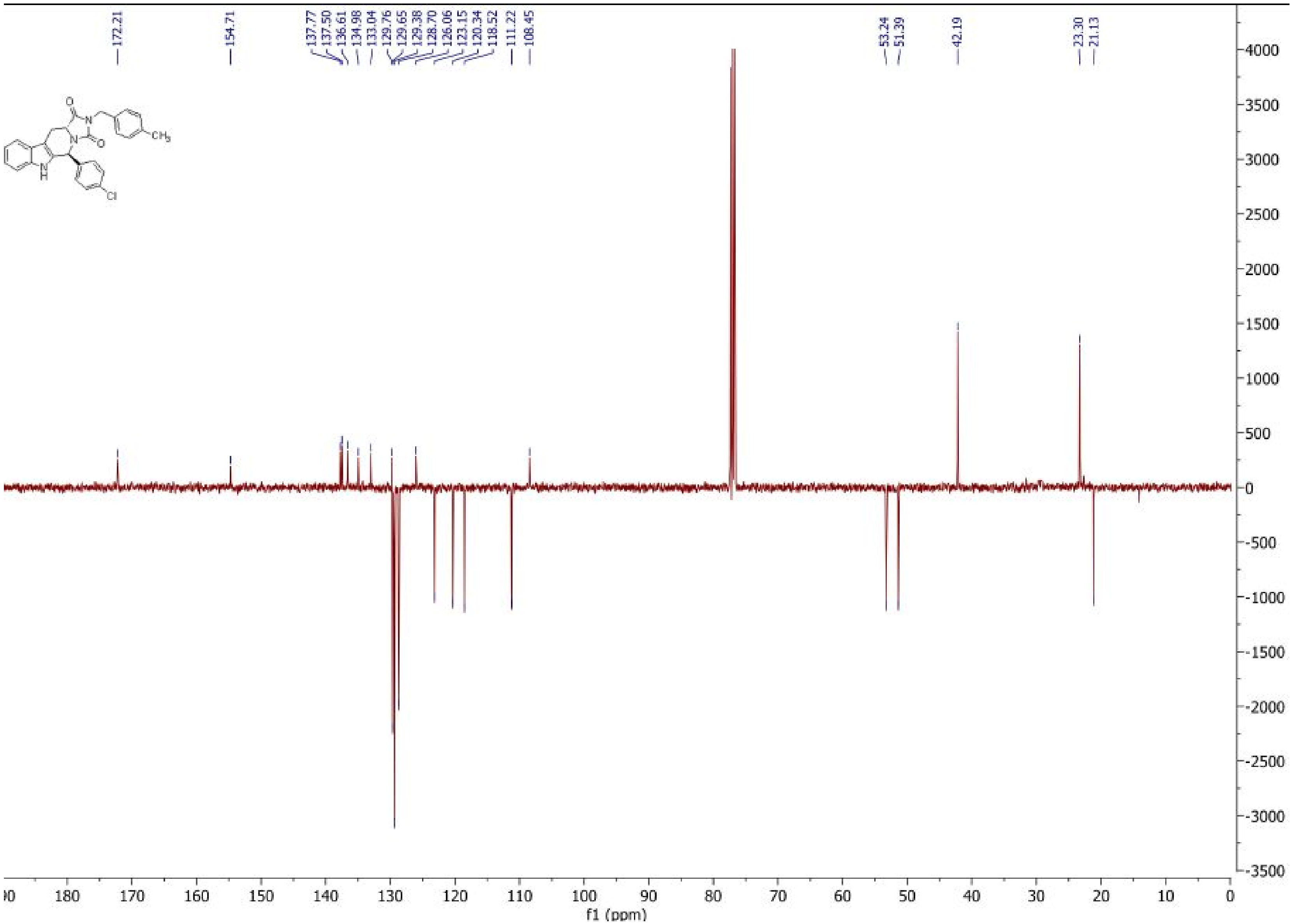

Figure S54: qDEPT spectra of **30a'**

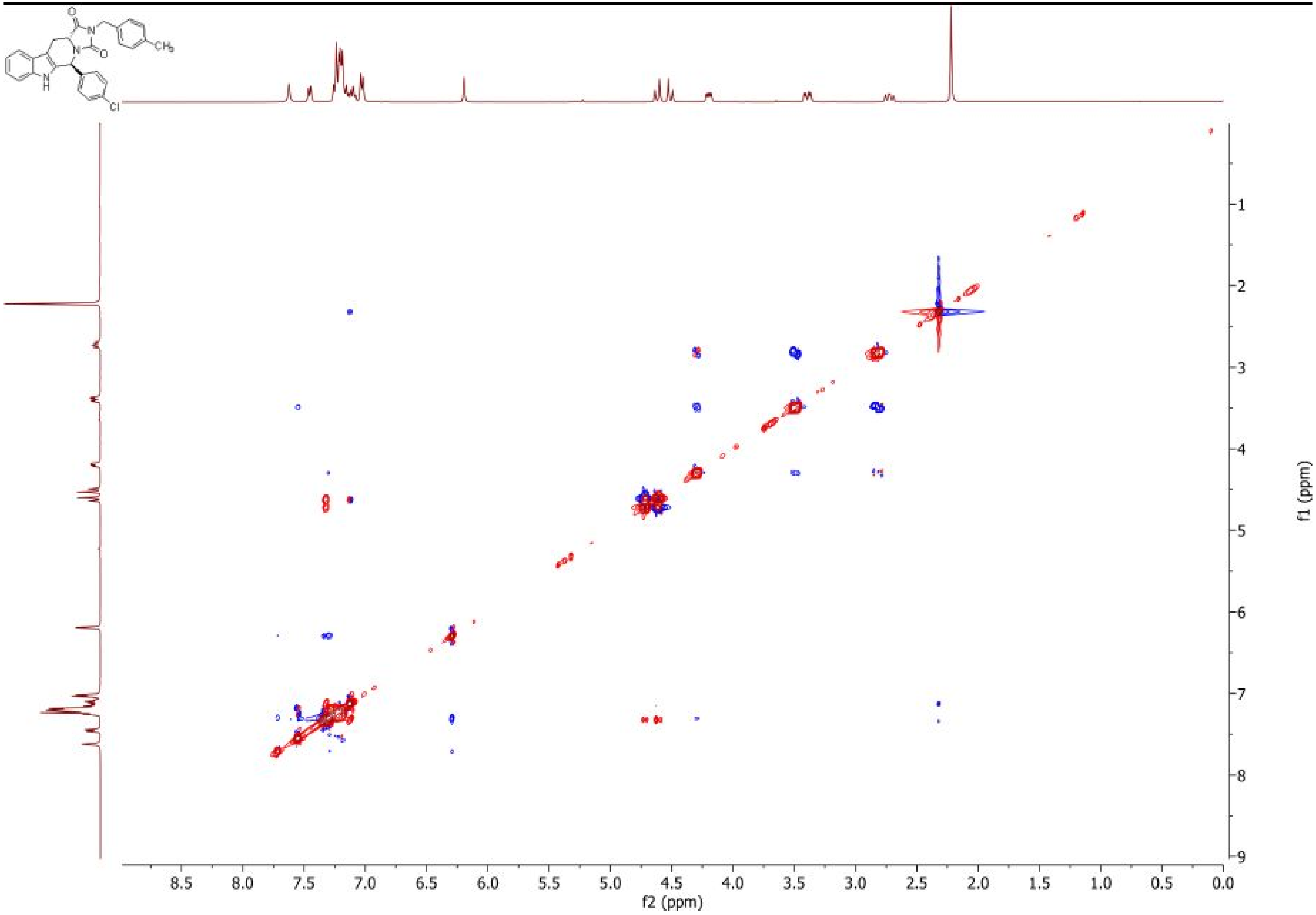

Figure S55: ROESY spectra of **30a'**

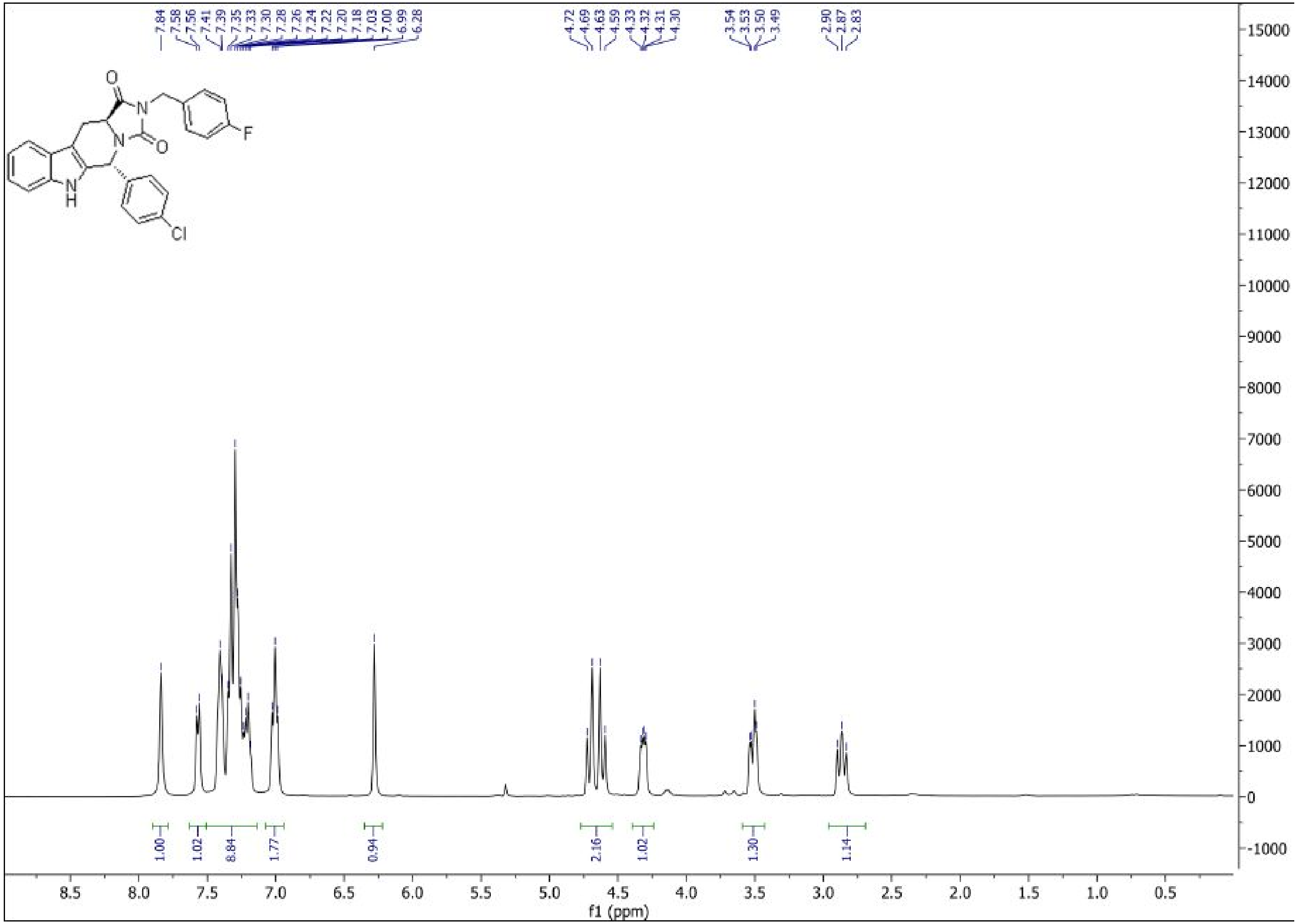

Figure S56: ^{1}H spectra of **31a**

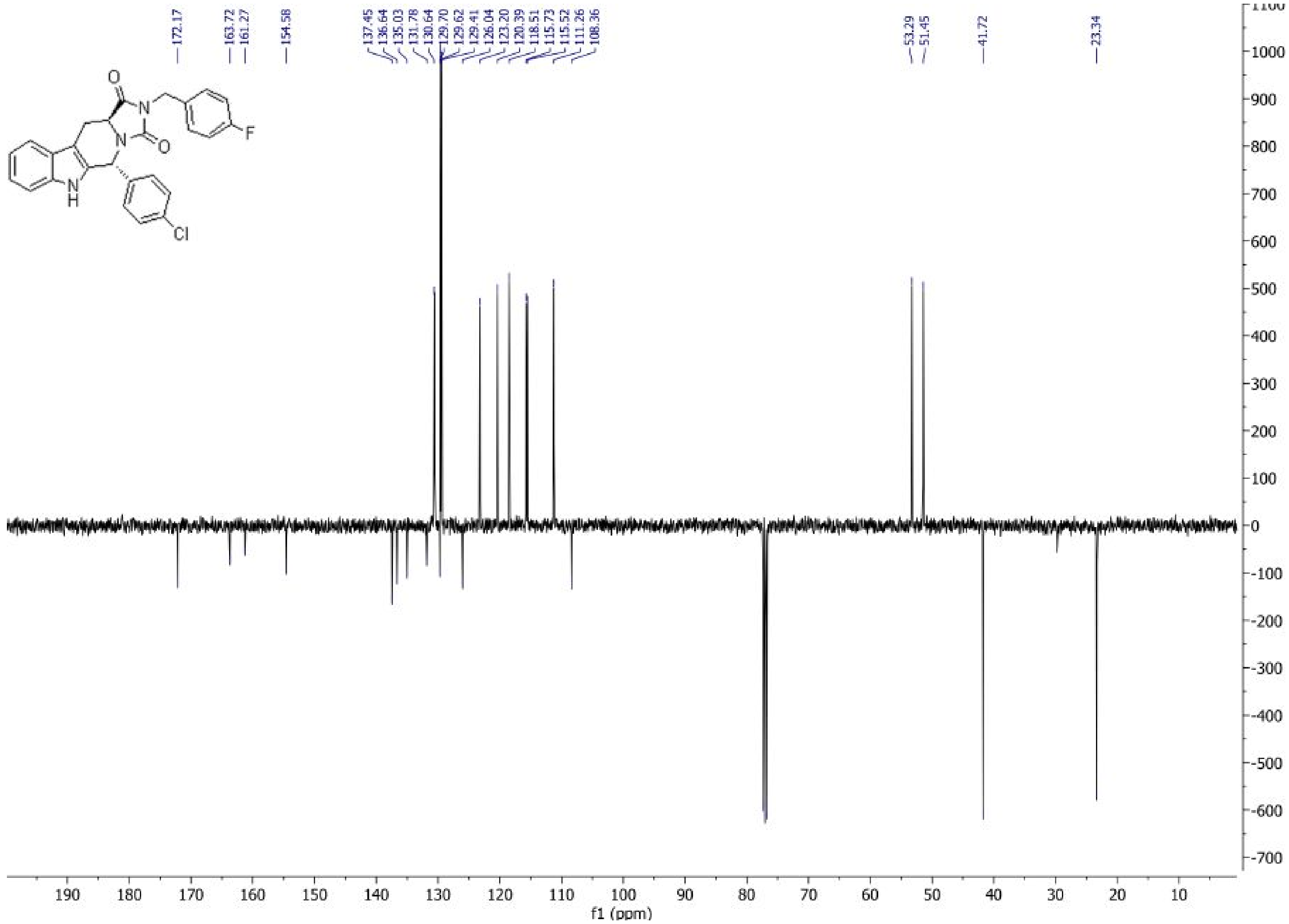

Figure S57: qDEPT spectra of **31a**

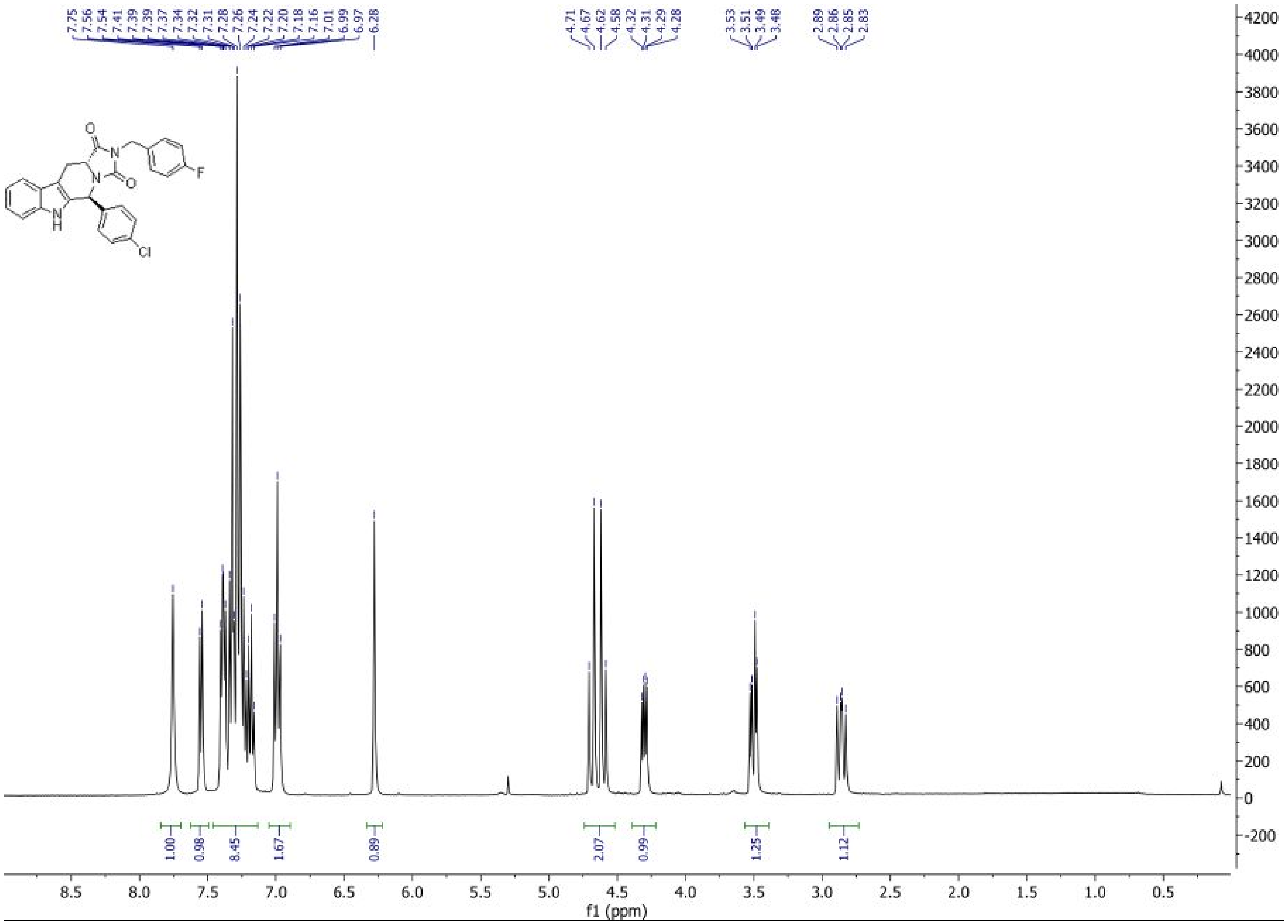

Figure S58: ^{1}H spectra of **31a'**

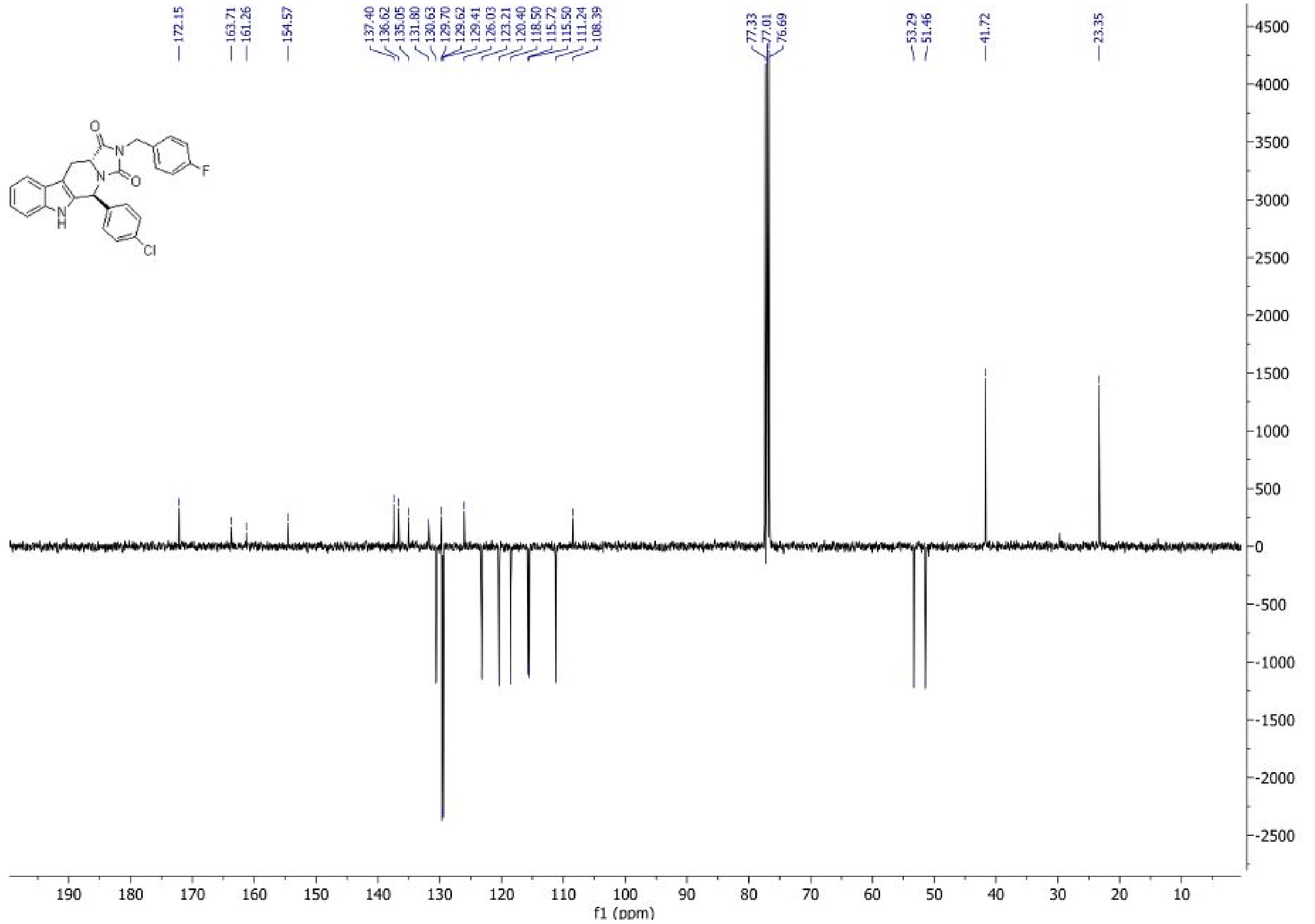

Figure S59: qDEPT spectra of **31a'**

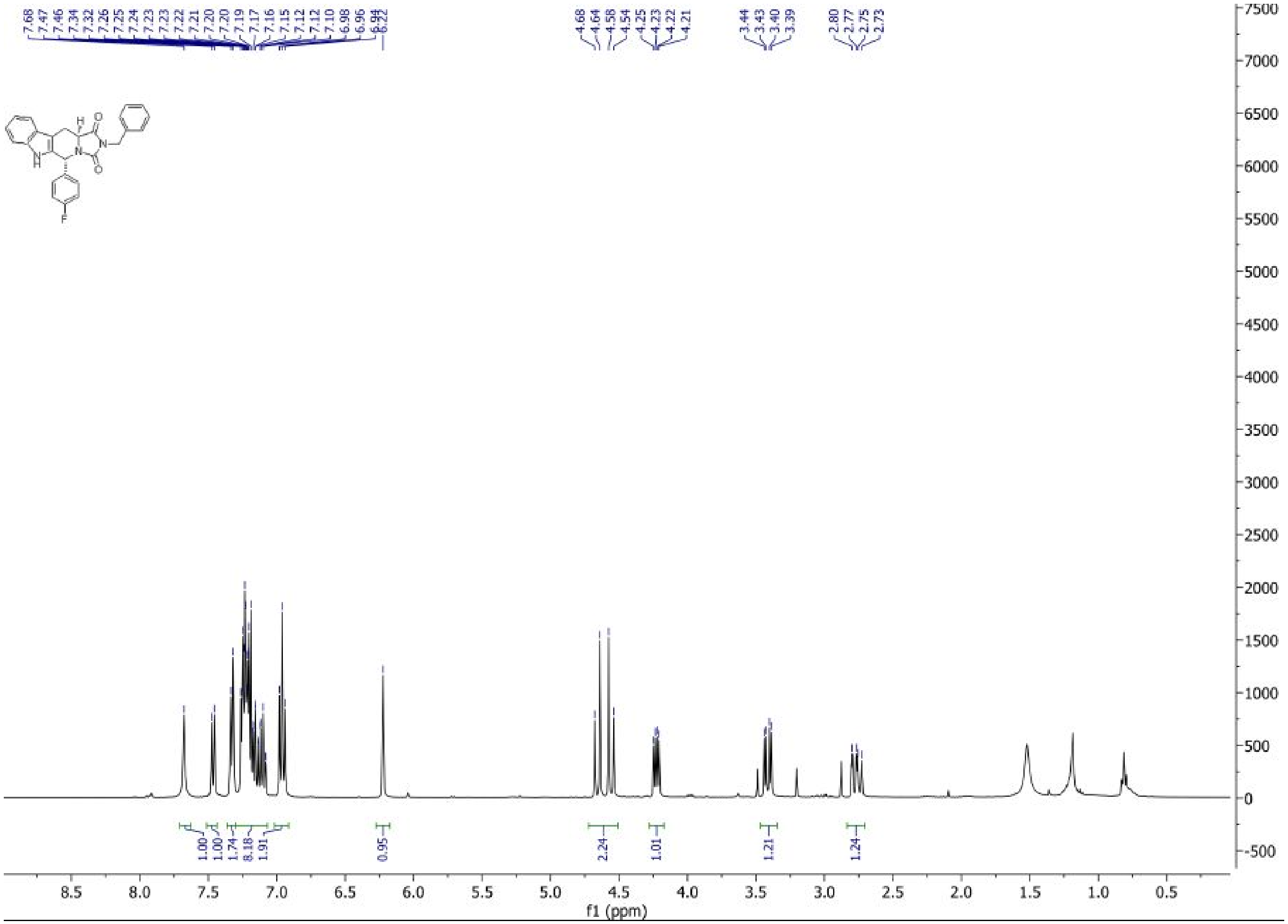

Figure S60: ^{1}H spectra of **33a**

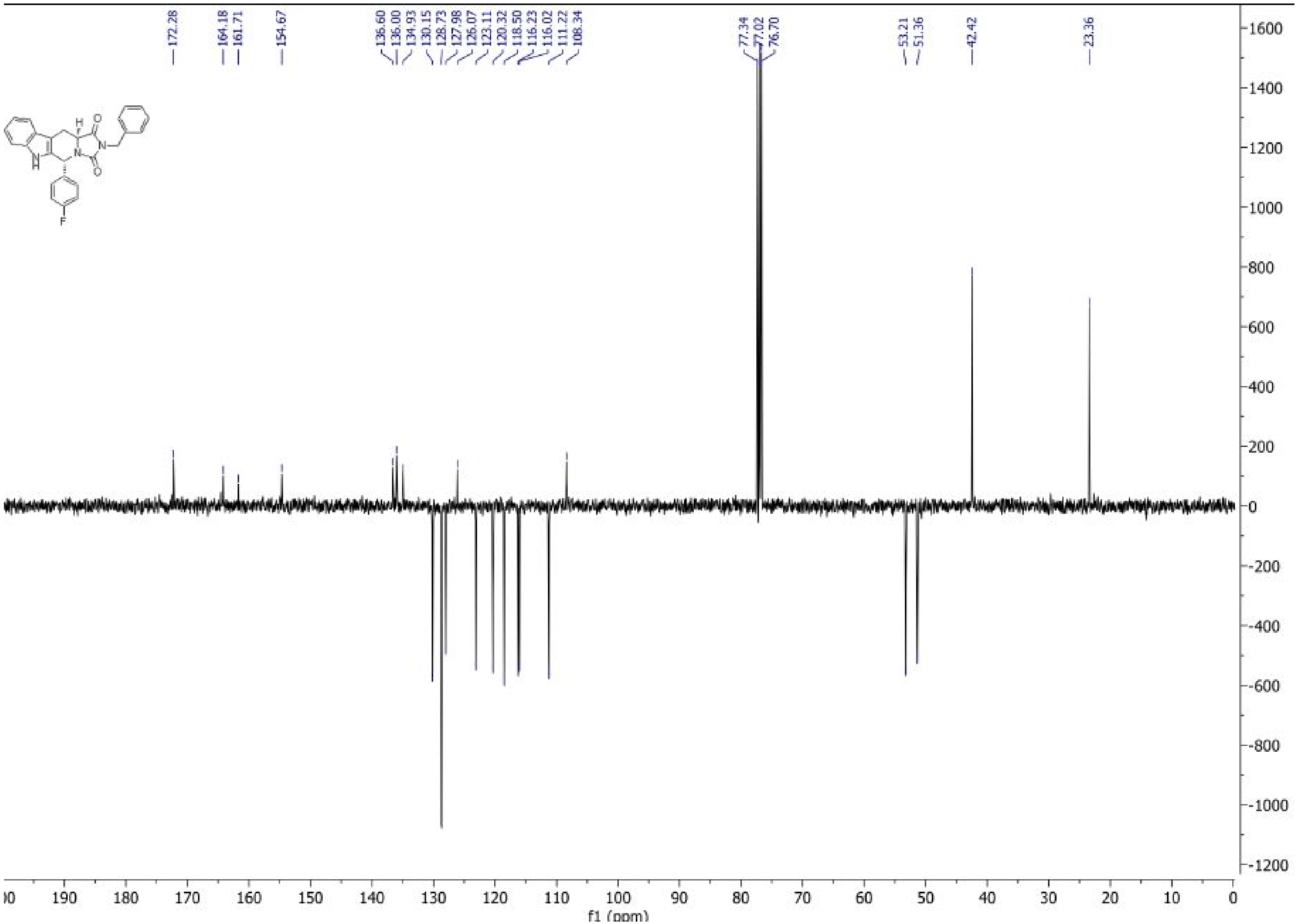

Figure S61: qDEPT spectra of **33a**

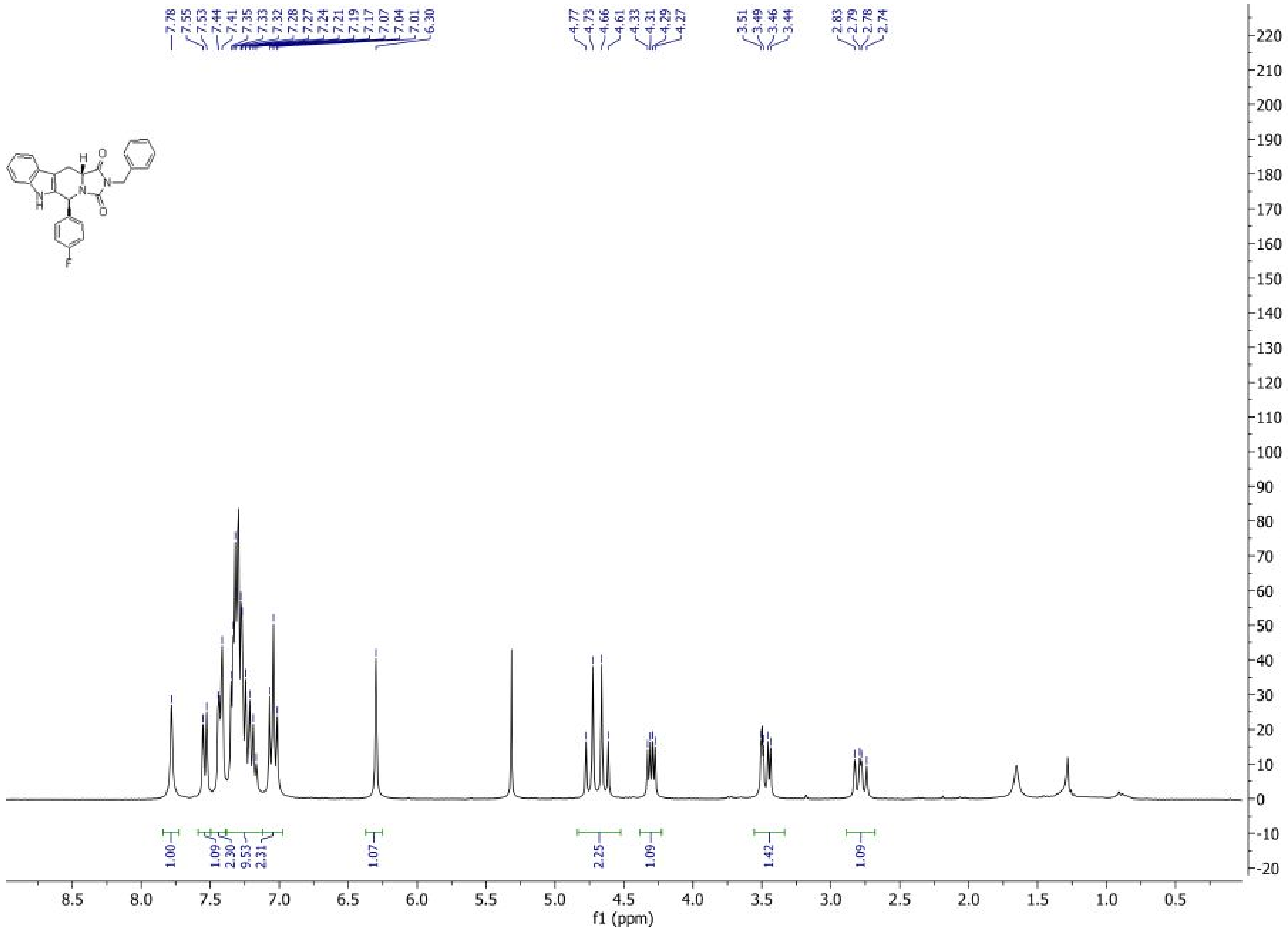

Figure S62: ^{1}H spectra of **33a'**

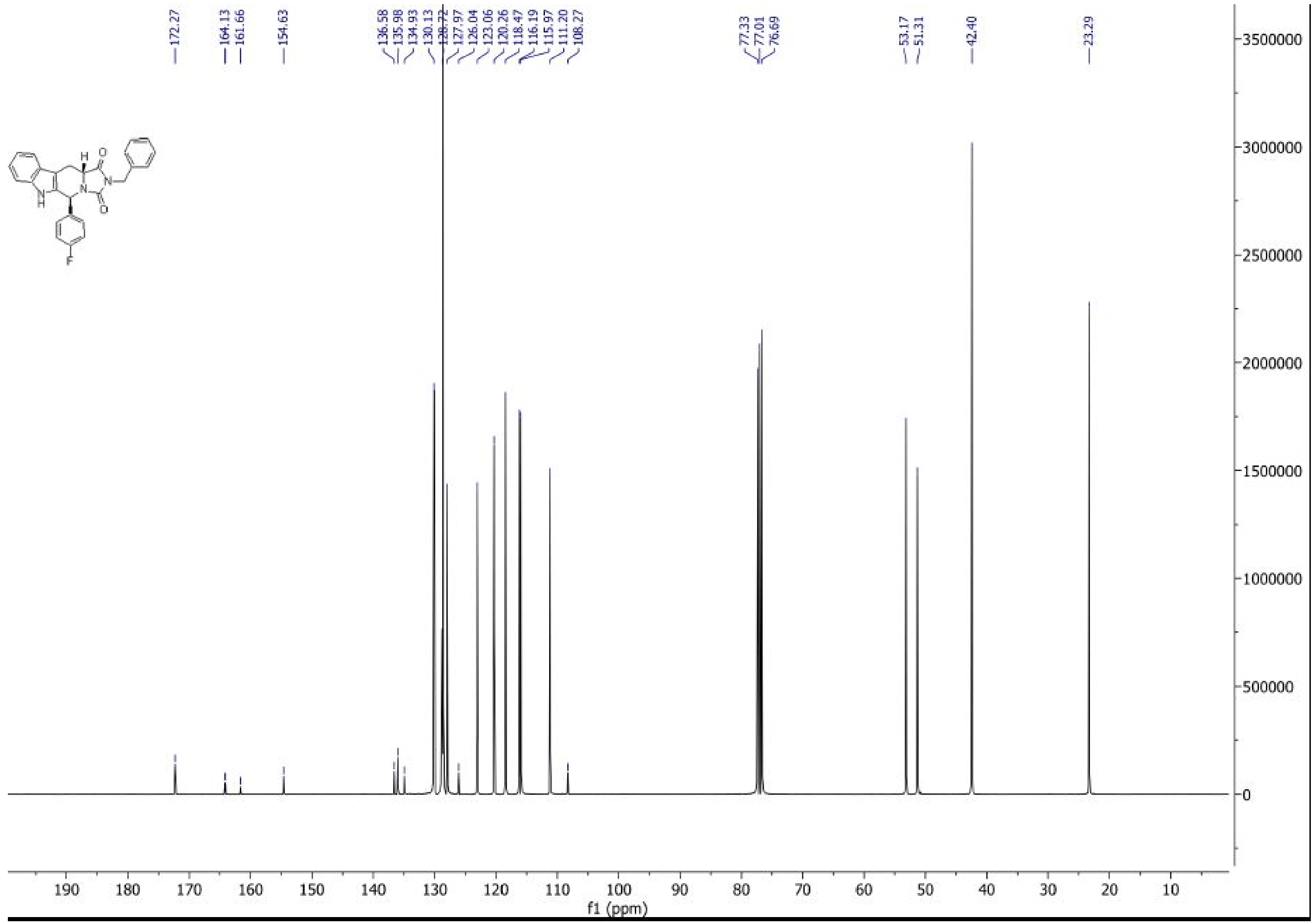

Figure S63: qDEPT spectra of **33a'**

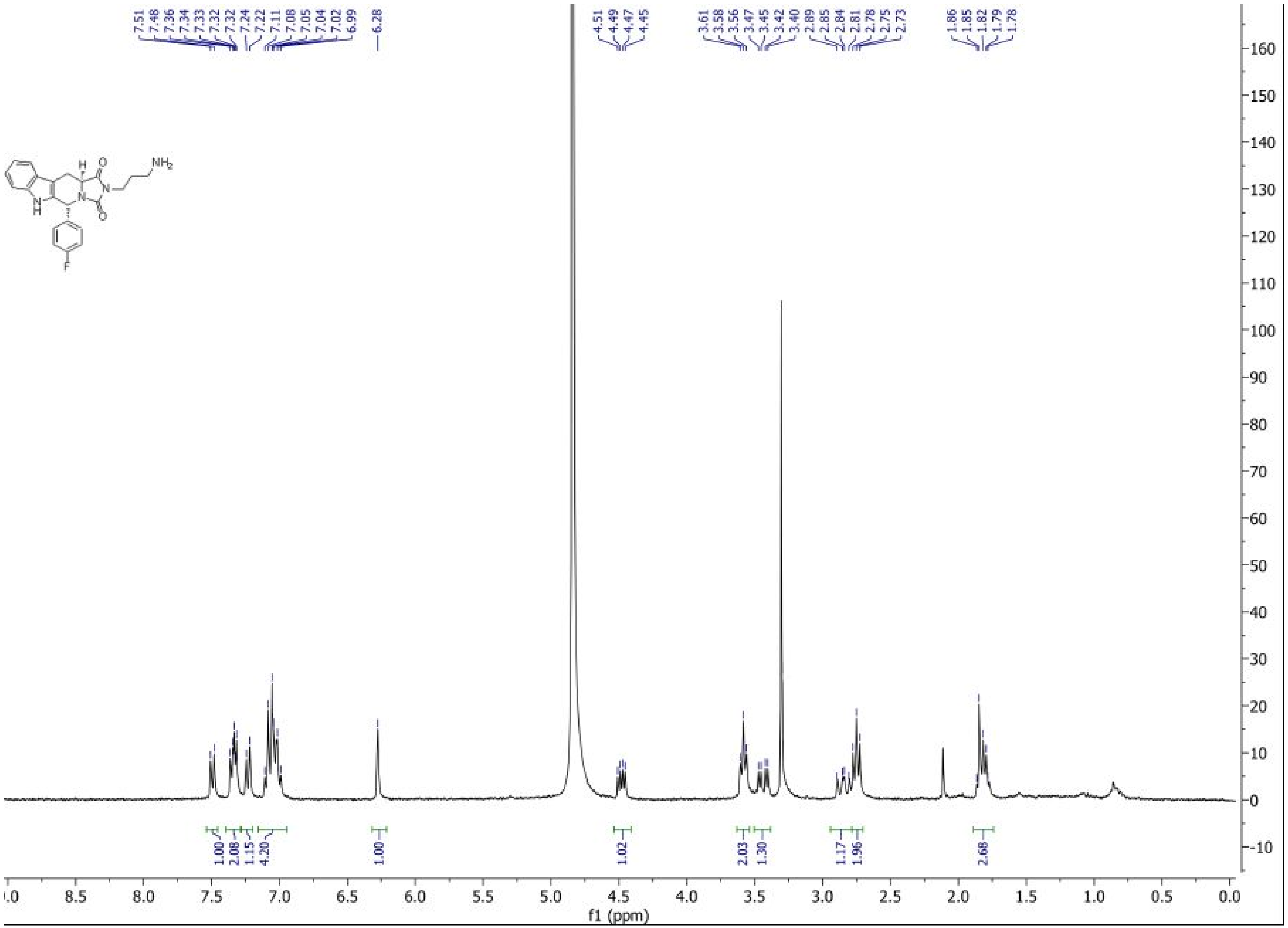

Figure S64: ^{1}H spectra of **35a**

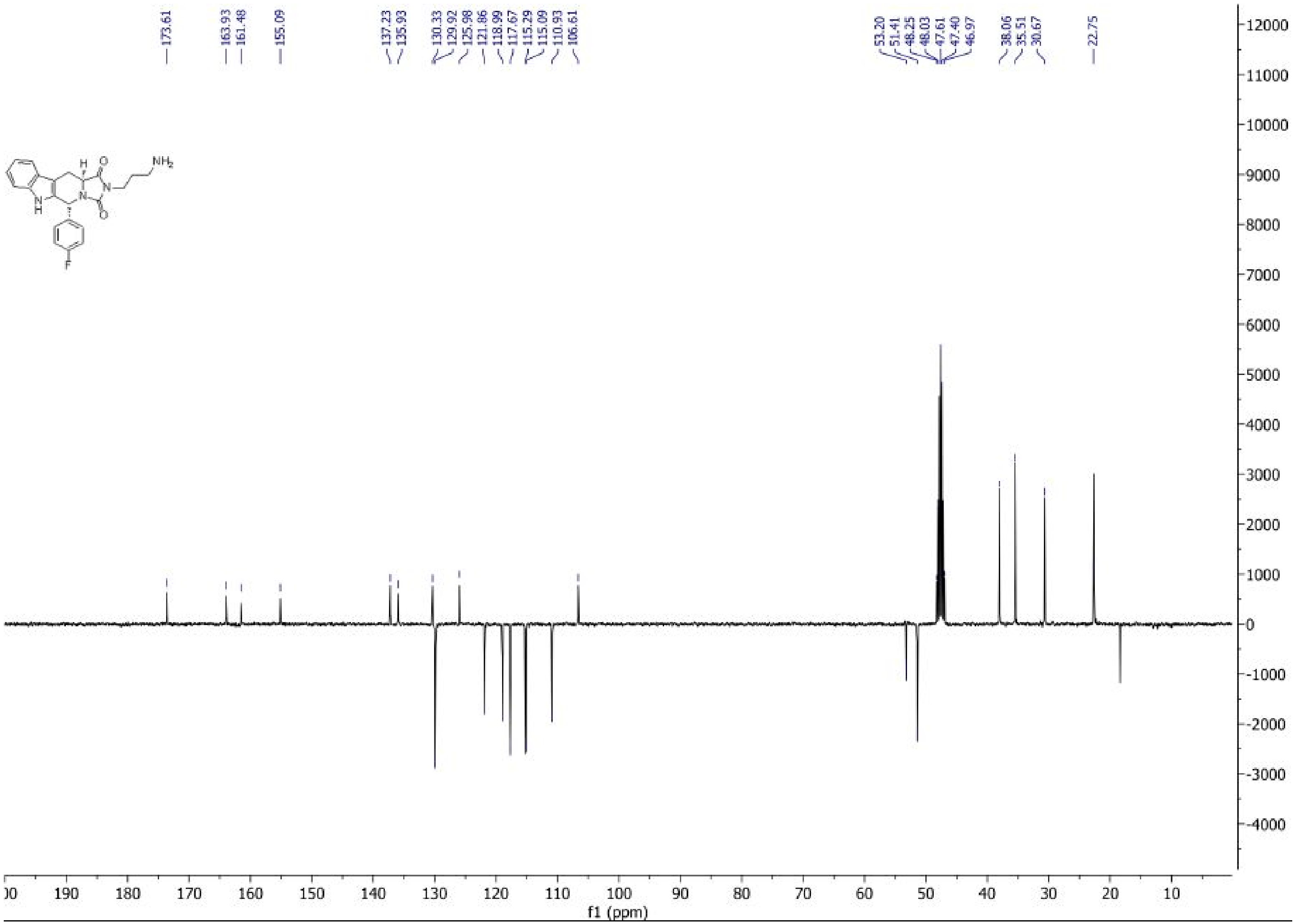

Figure S65: qDEPT spectra of **35a**

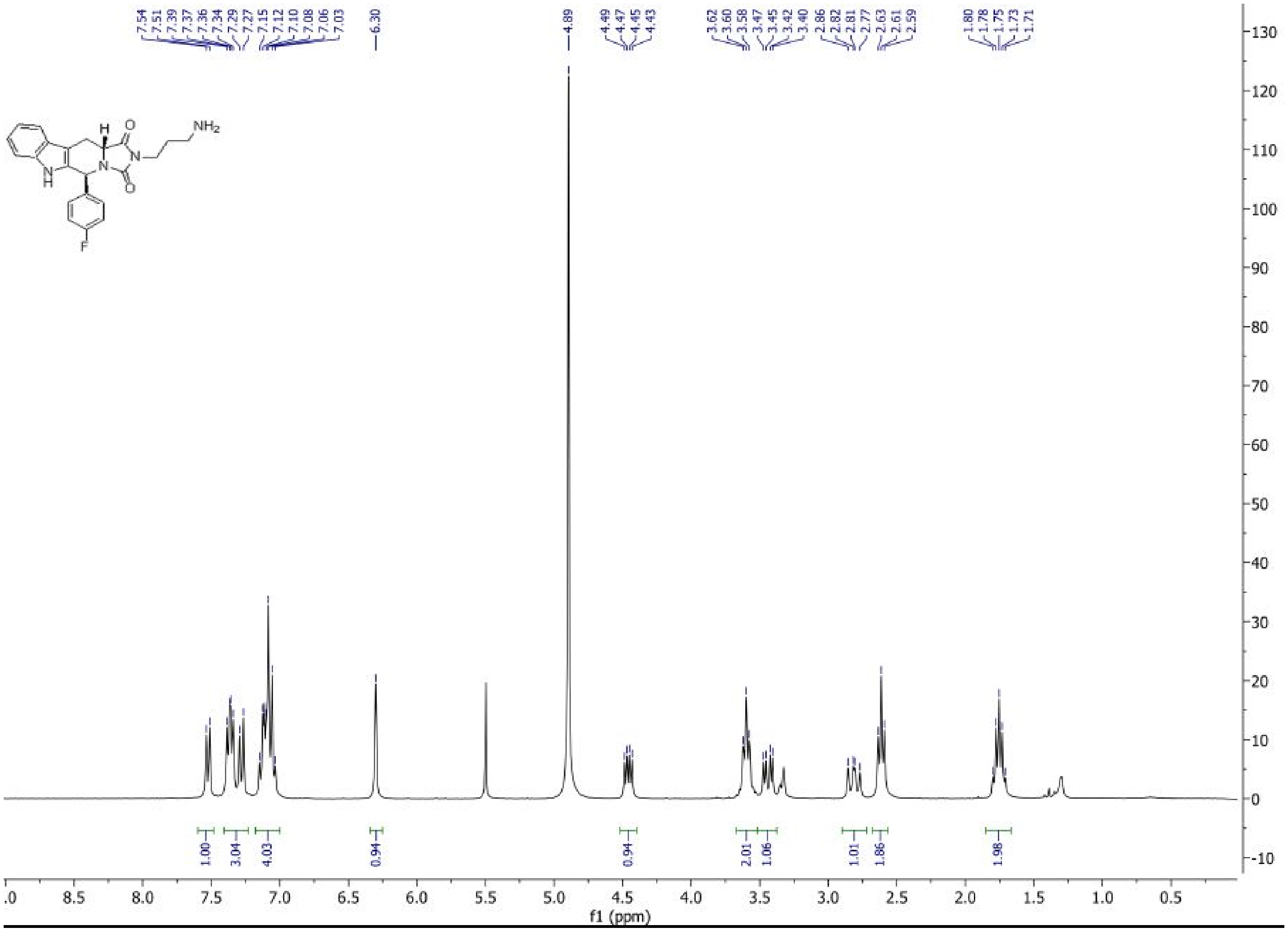

Figure S66: ^{1}H spectra of **35a'**

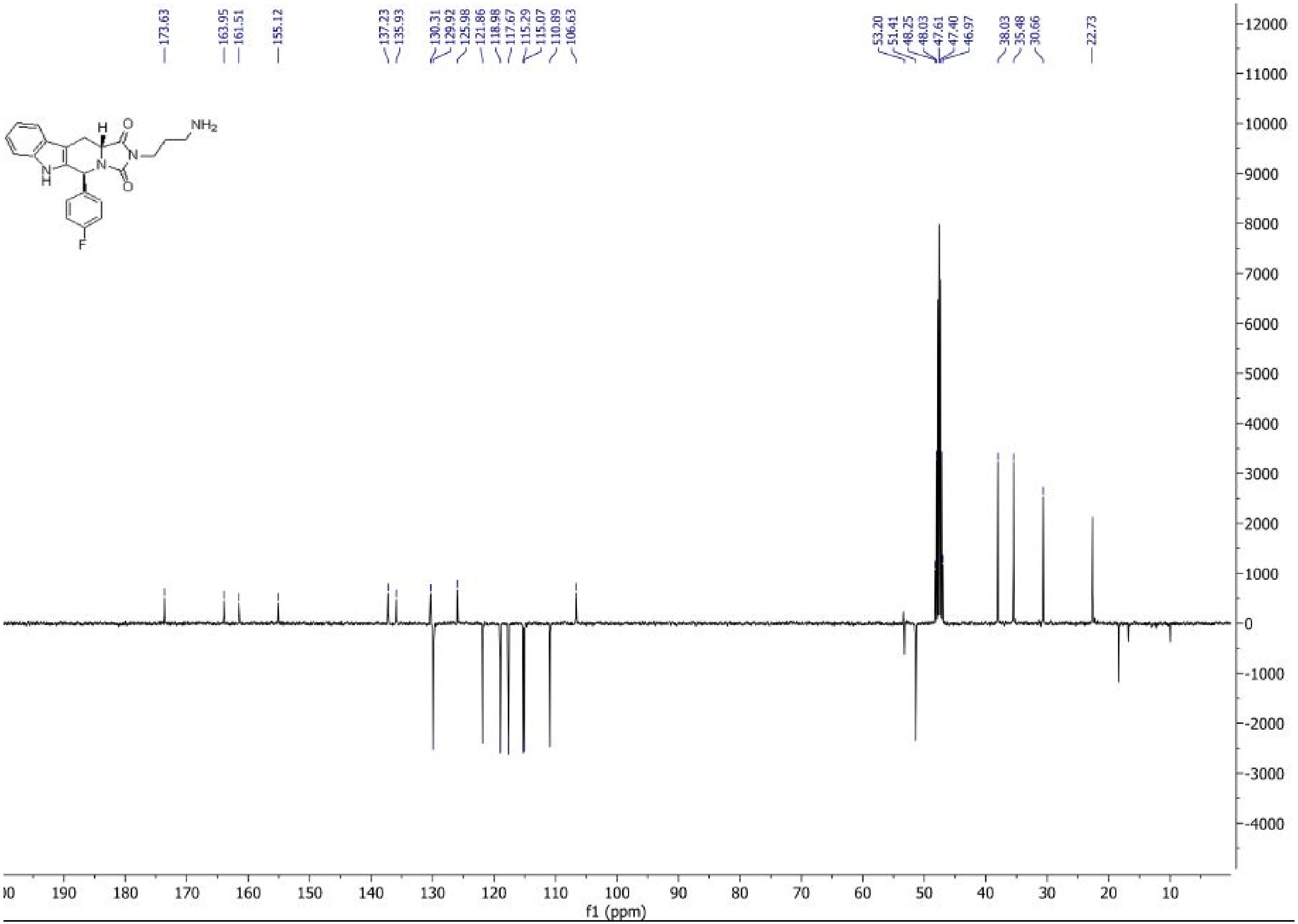

Figure S67: qDEPT spectra of **35a'**

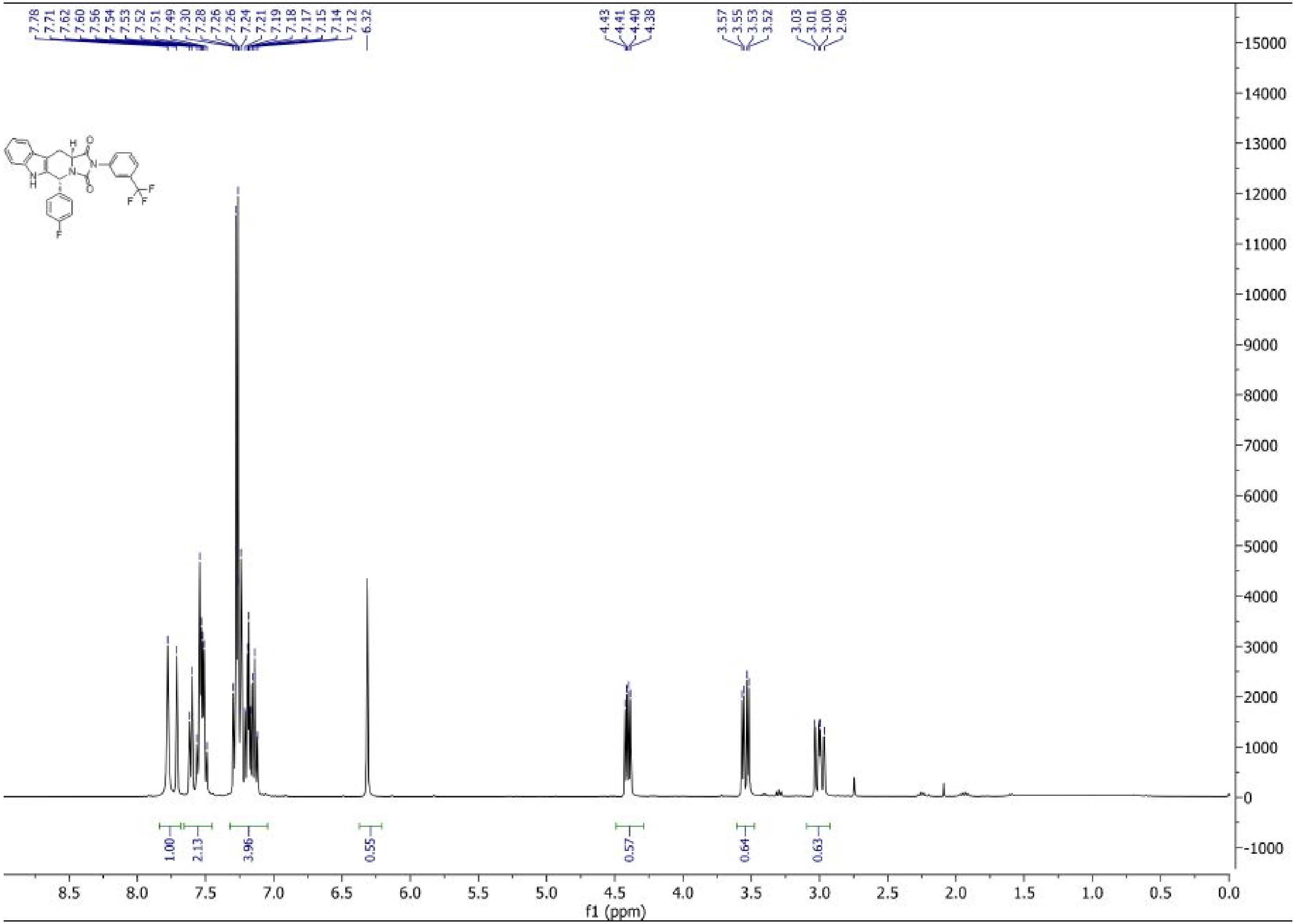

Figure S68: ^{1}H spectra of **36a**

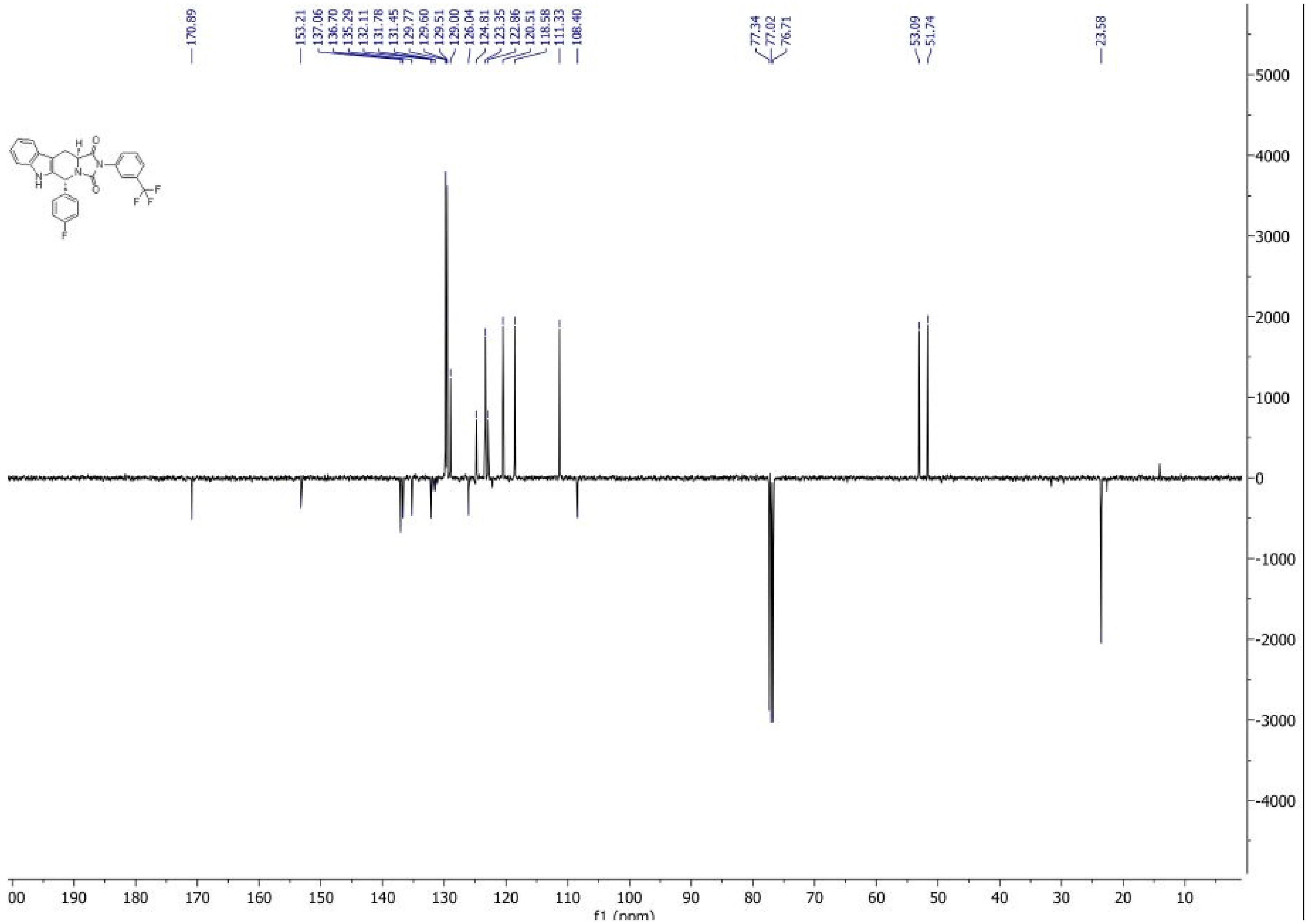

Figure S69: qDEPT spectra of **36a**

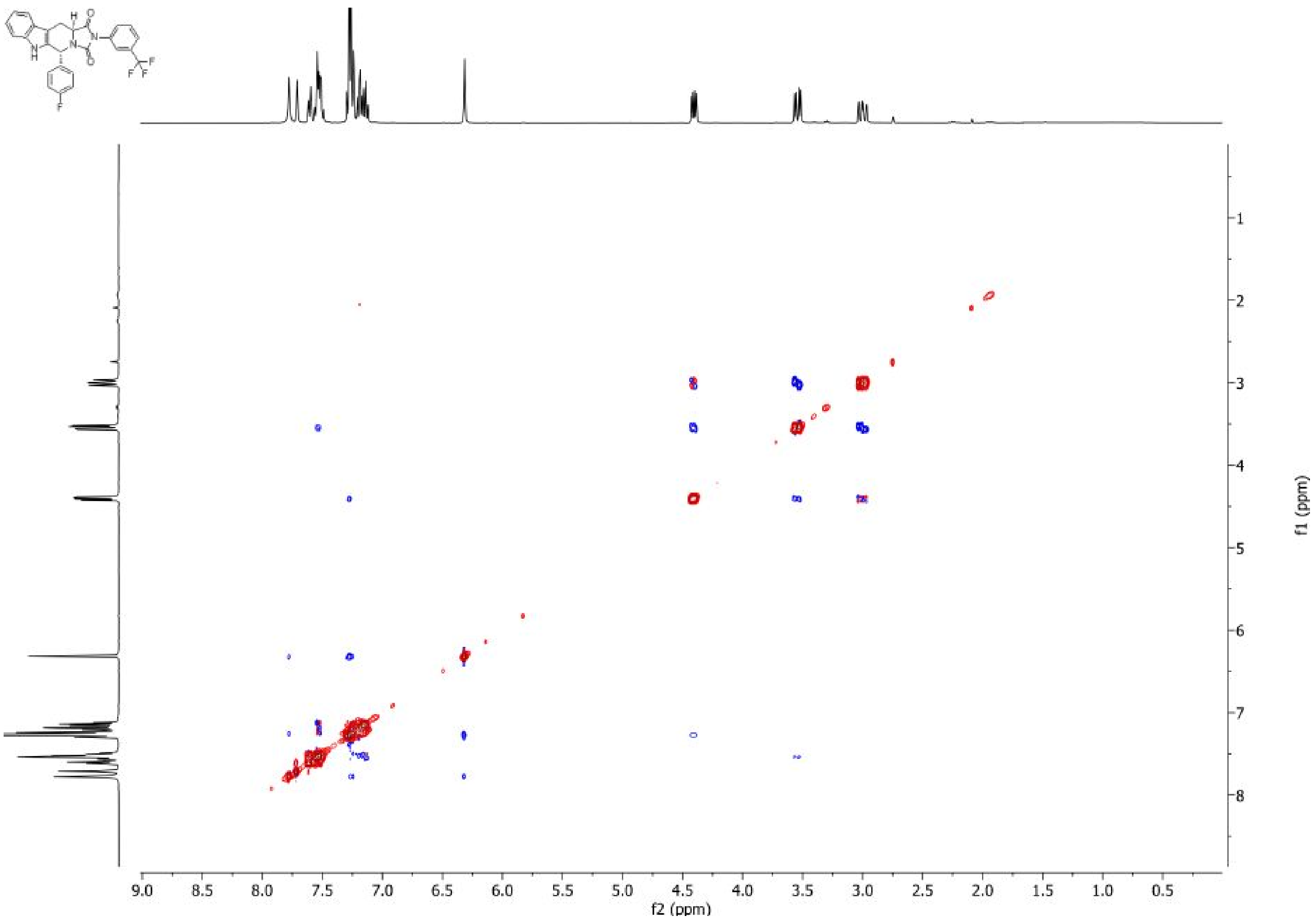

Figure S70: ROESY spectra of **36a**

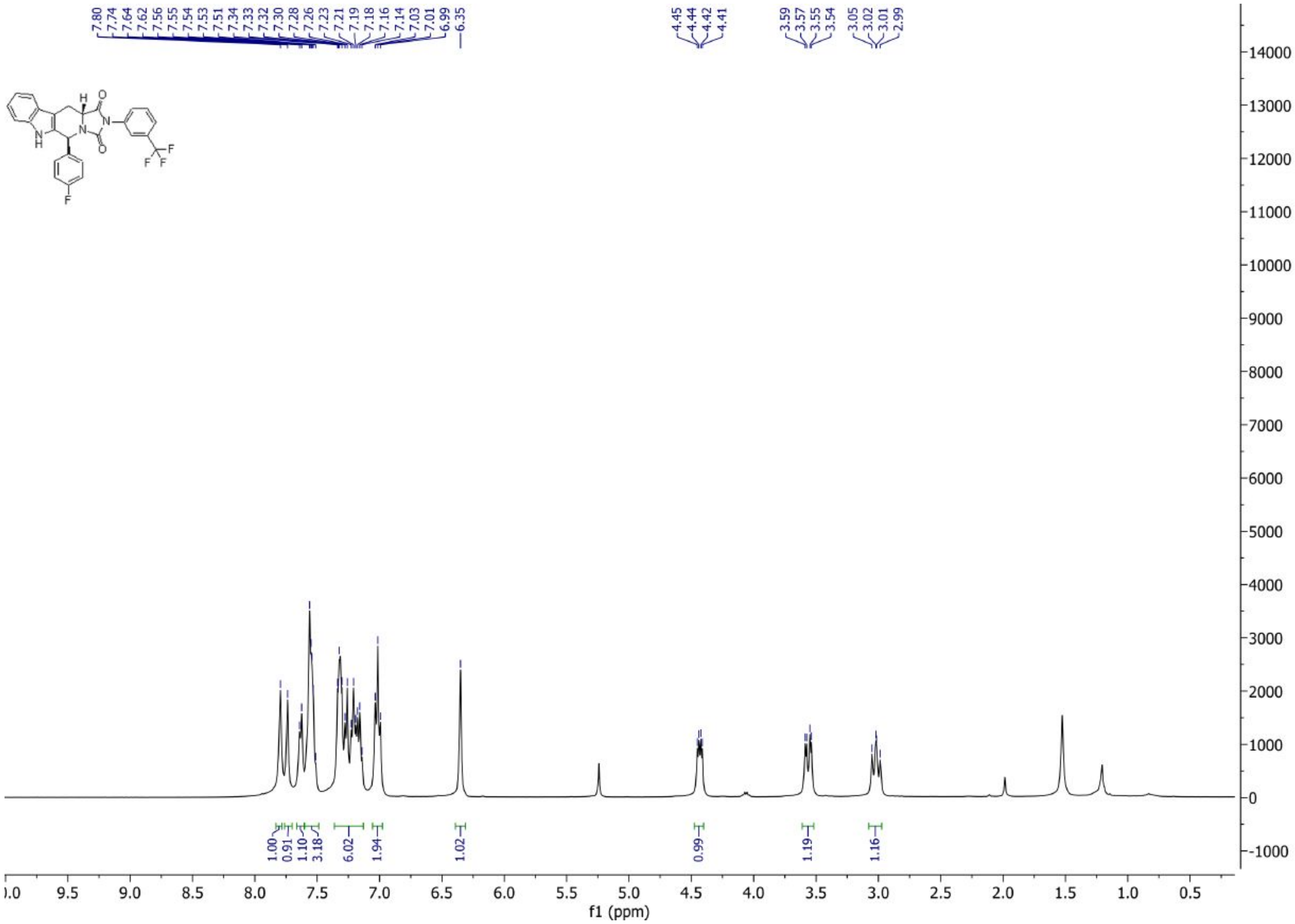

Figure S71: ^{1}H spectra of **36a'**

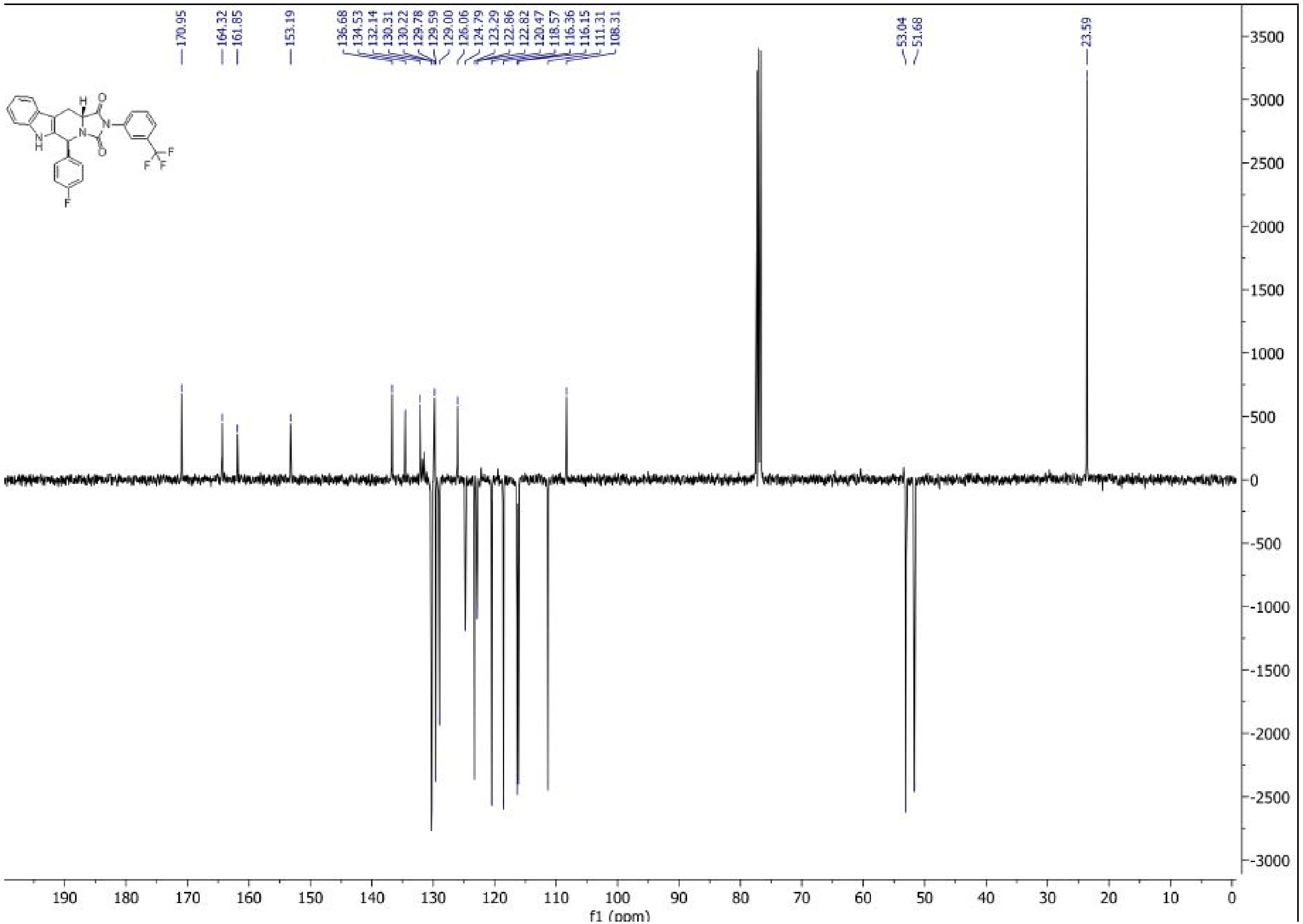

Figure S72: qDEPT spectra of **36a'**

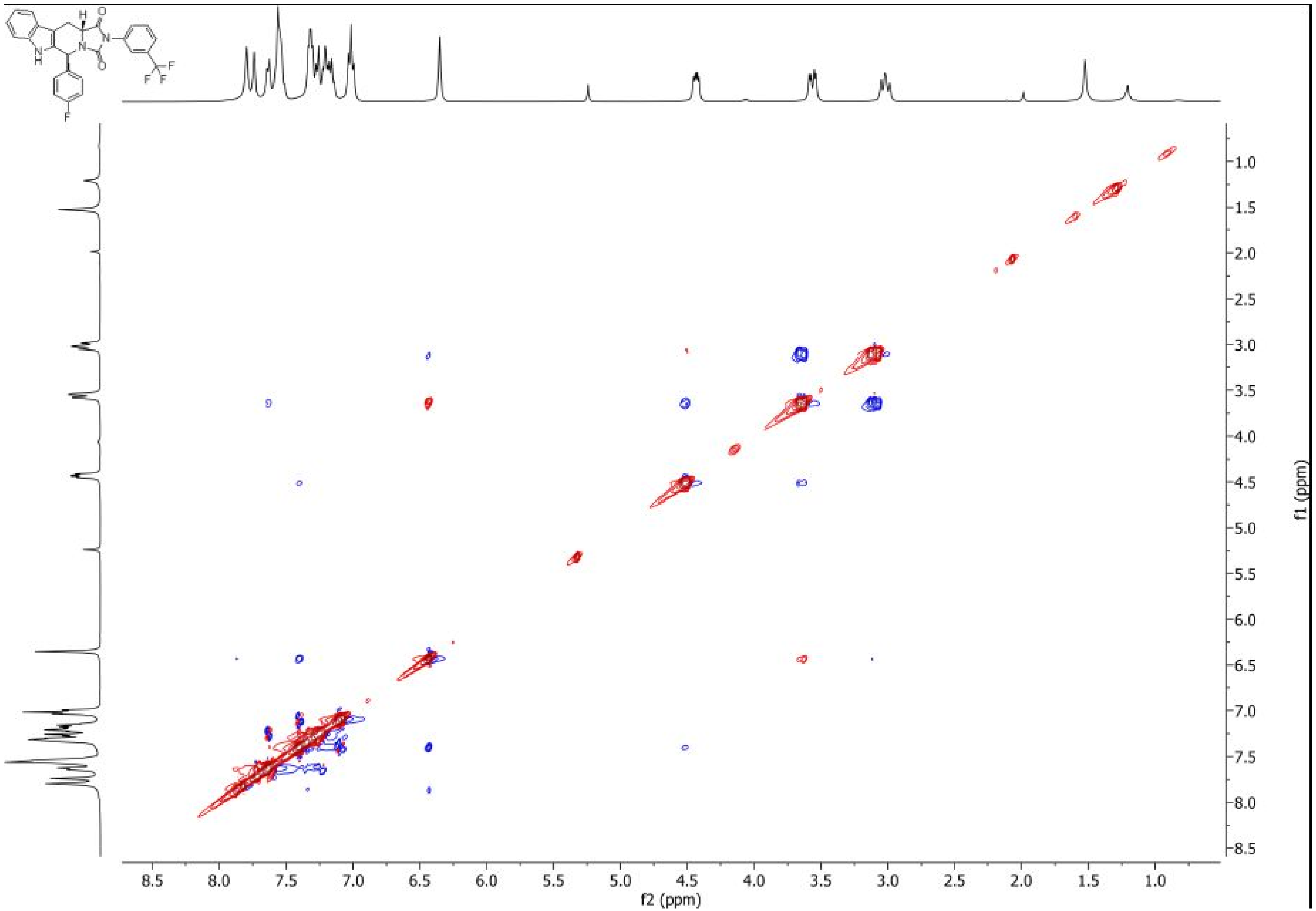

Figure S73: ROESY spectra of **36a’**

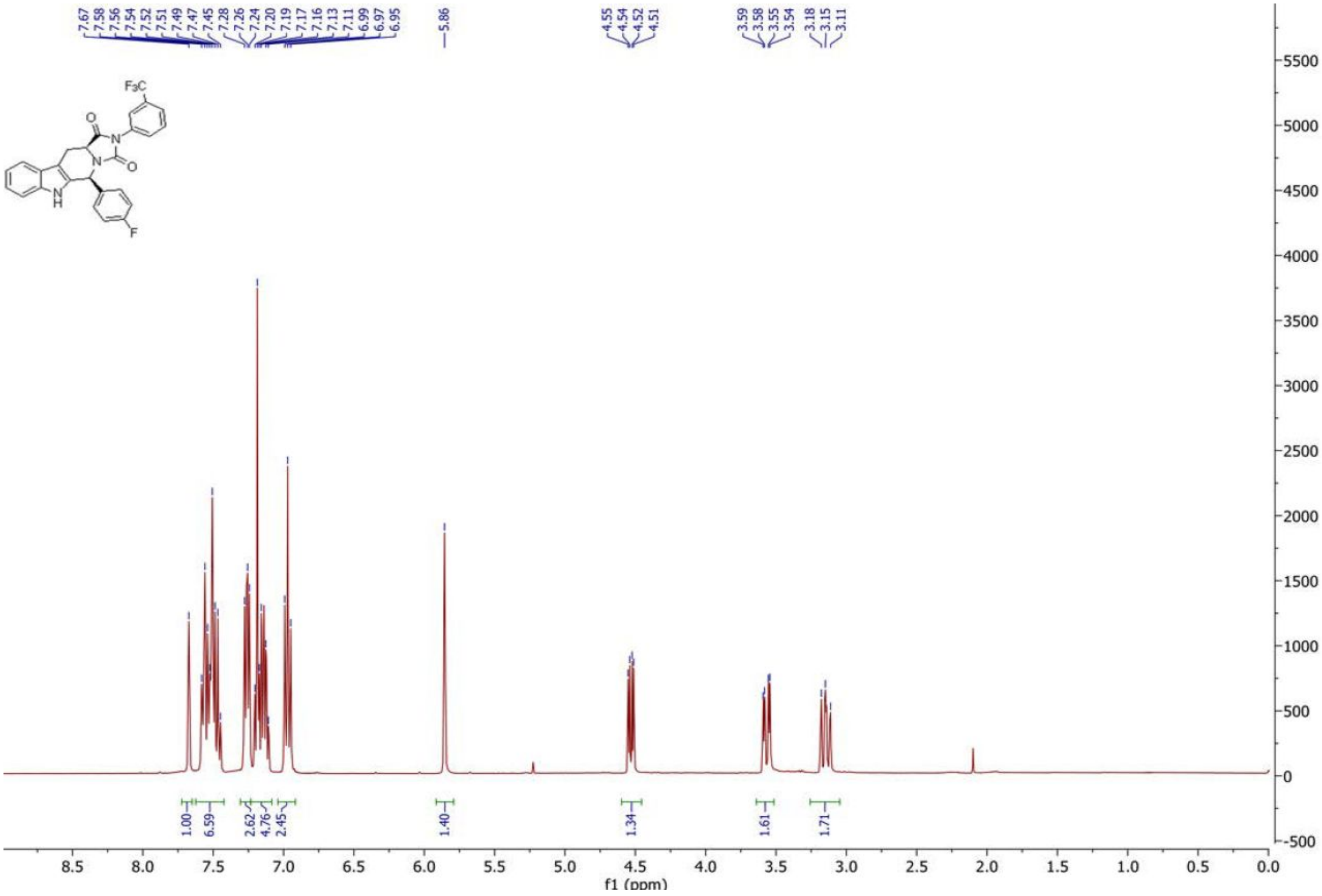

Figure S74: ^{1}H spectra of **36b**

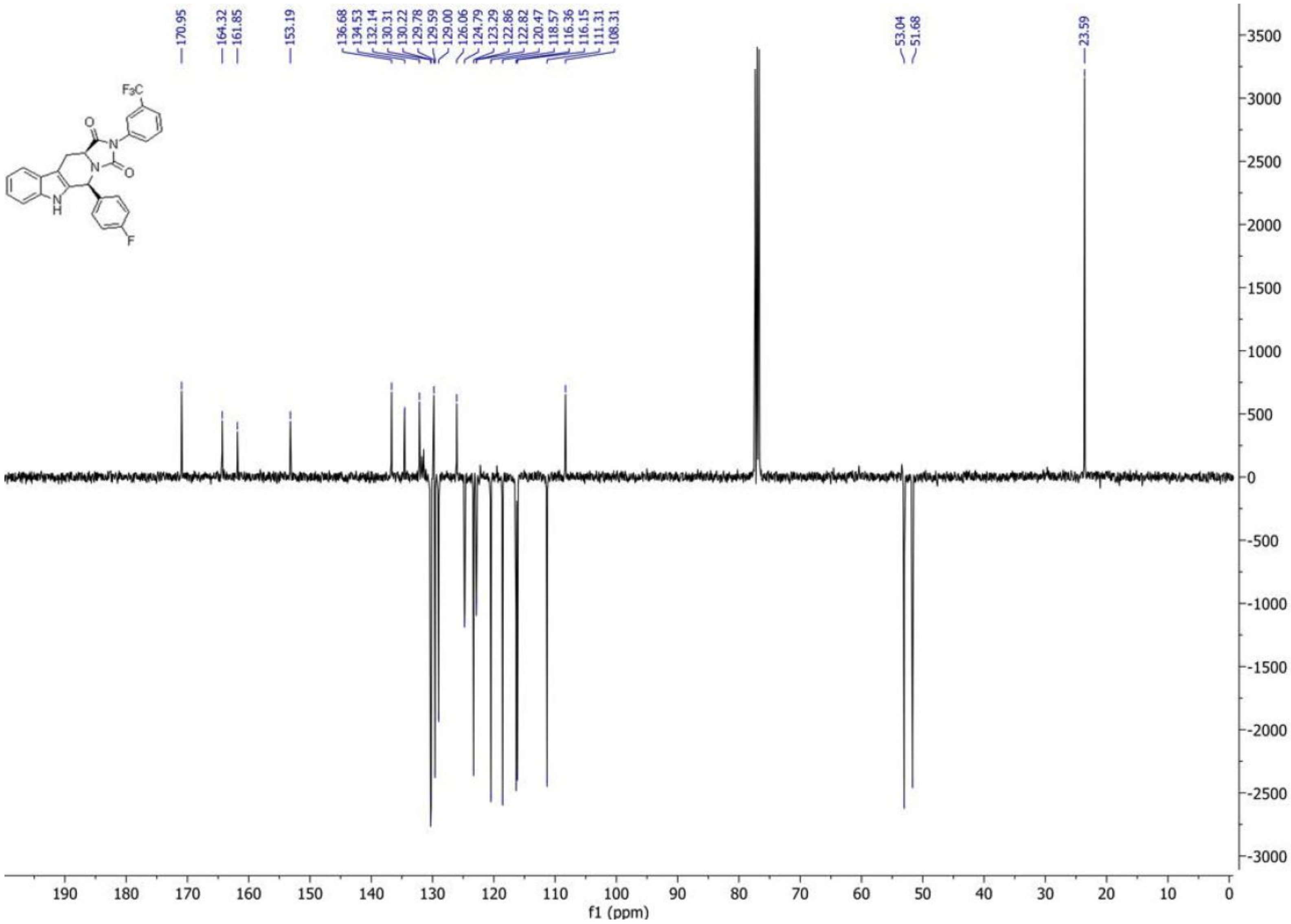

Figure S75: qDEPT spectra of **36b**

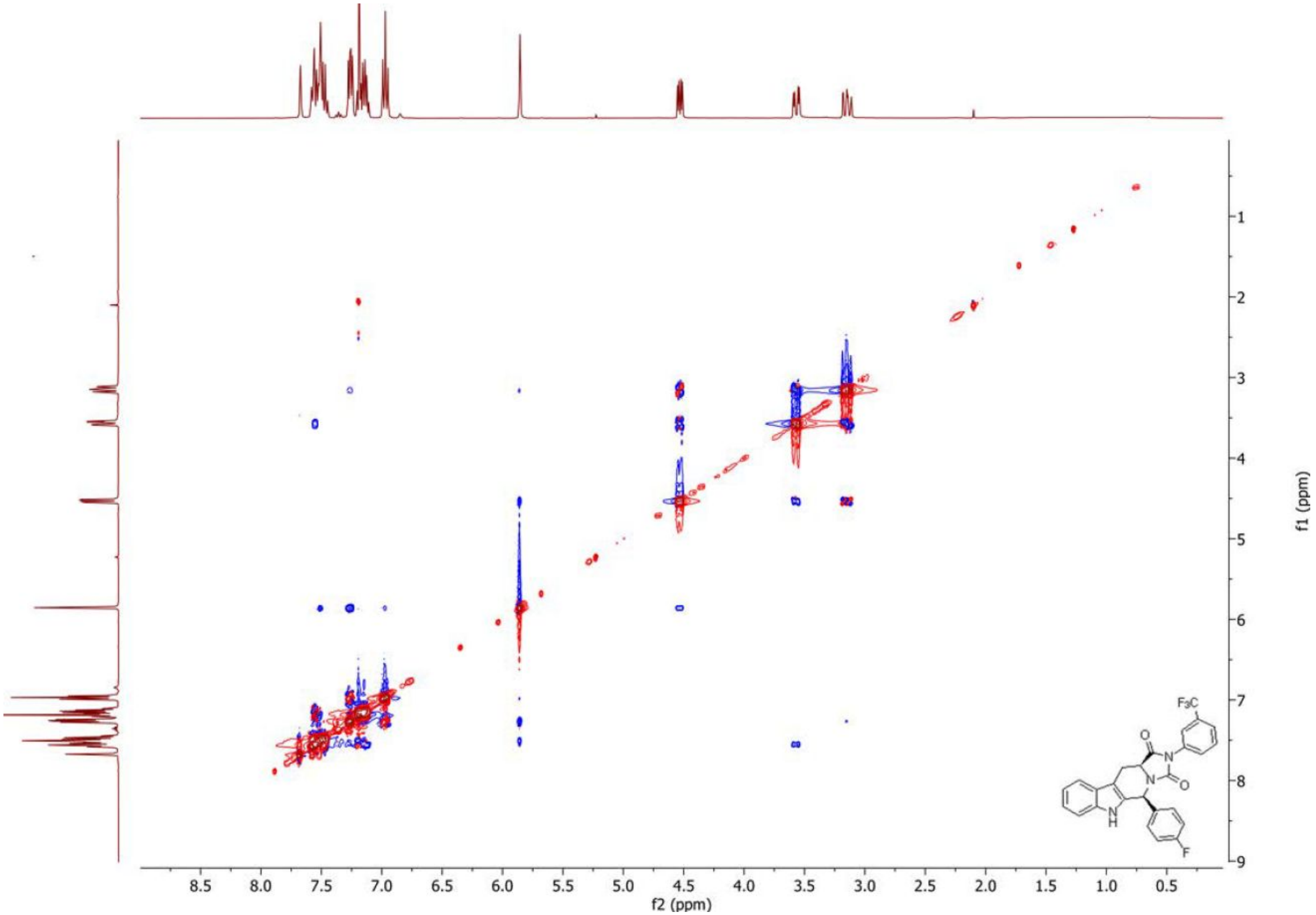

Figure S76: ROESY spectra of **36b**

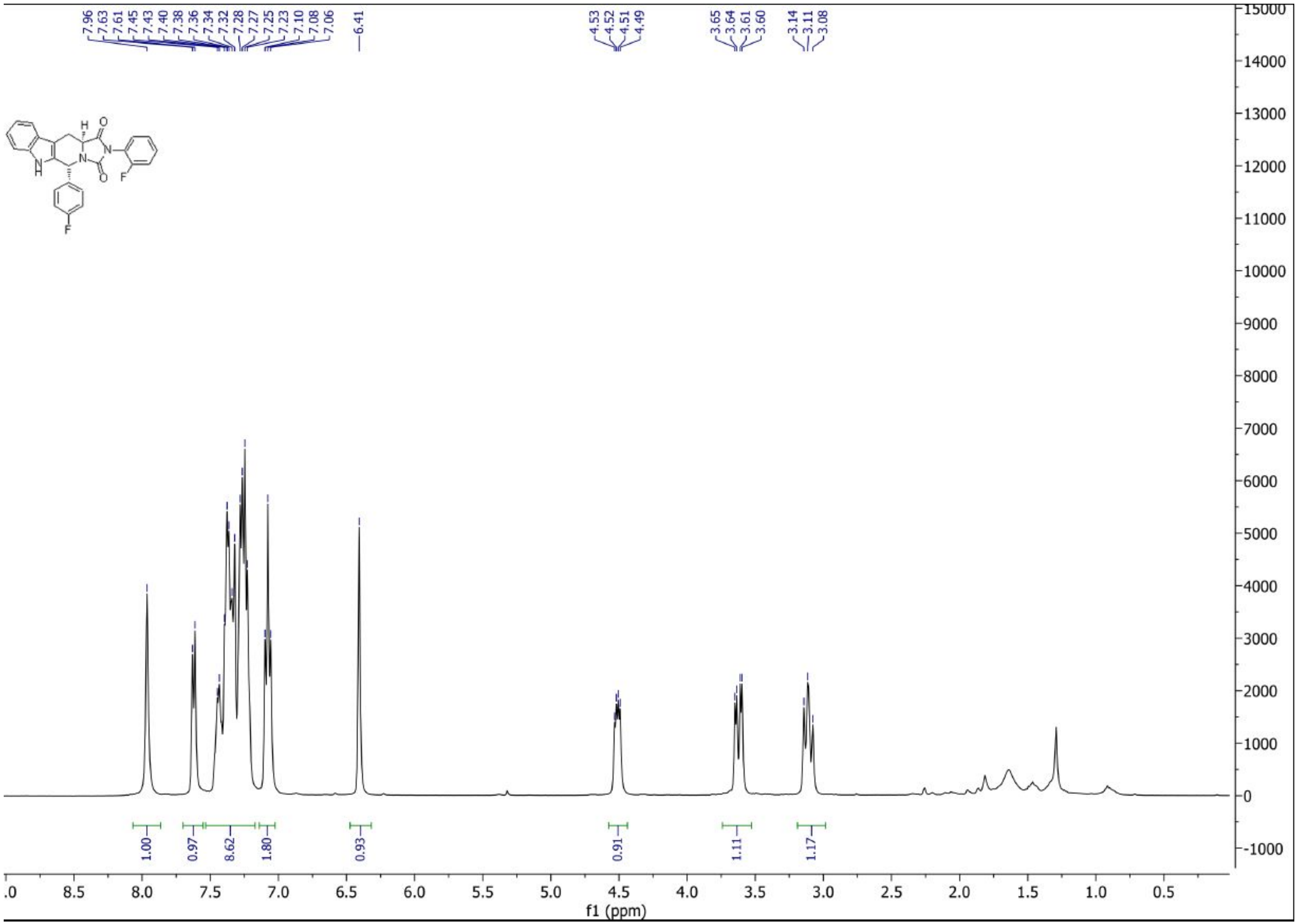

Figure S77: ^{1}H spectra of **37a**

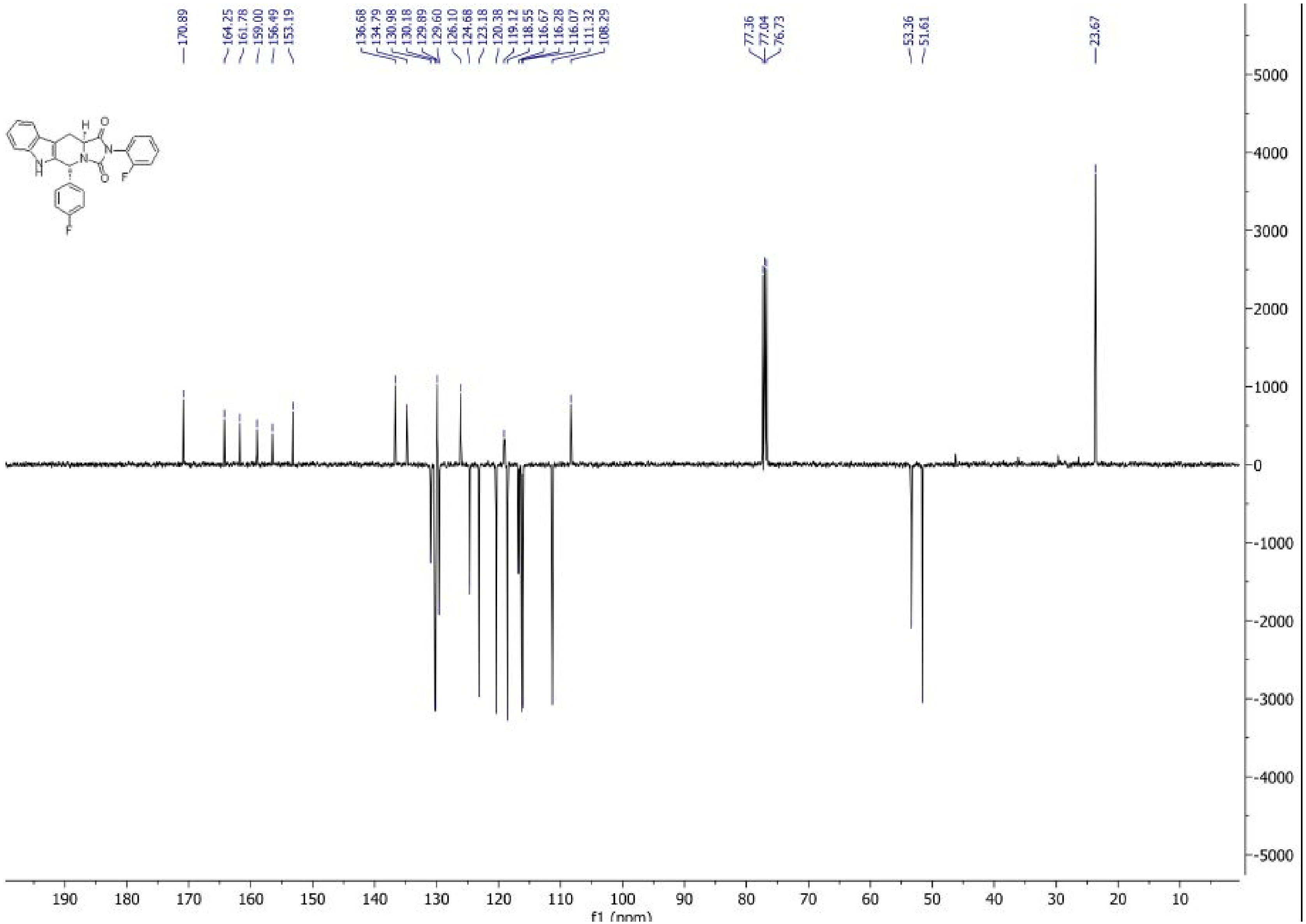

Figure S78: qDEPT spectra of **37a**

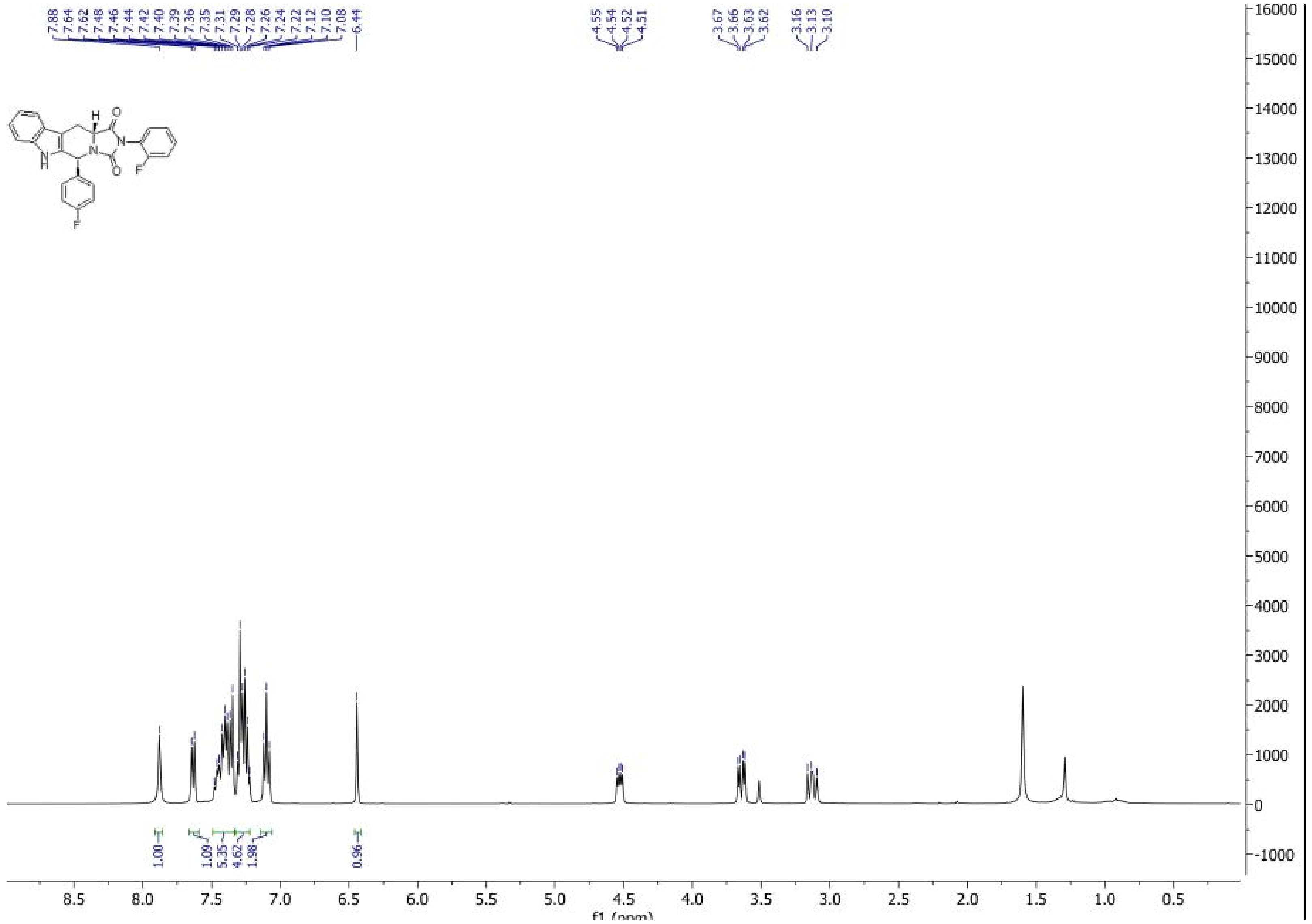

Figure S79: ^{1}H spectra of **37a’**

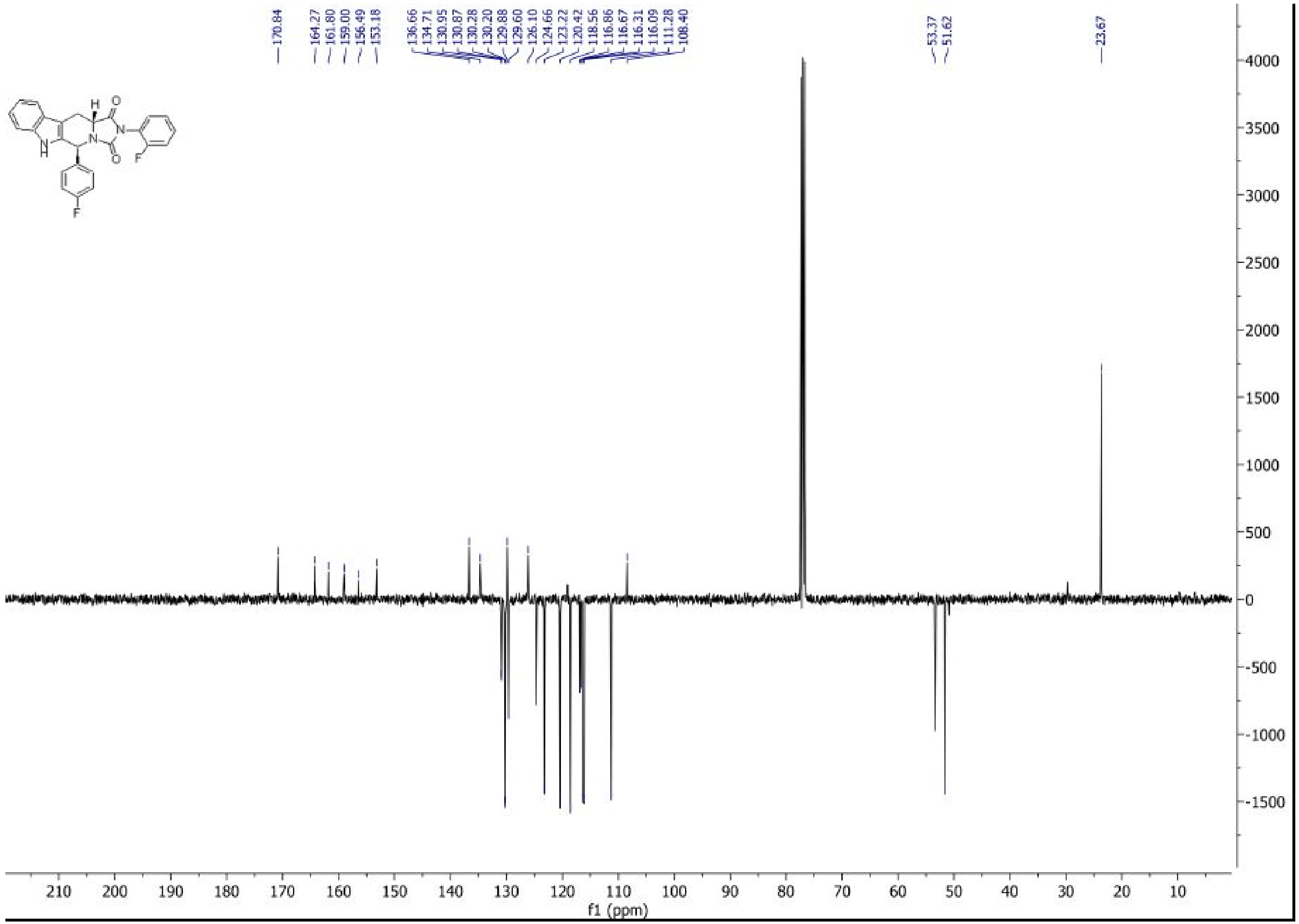

Figure S80: qDEPT spectra of **37a'**

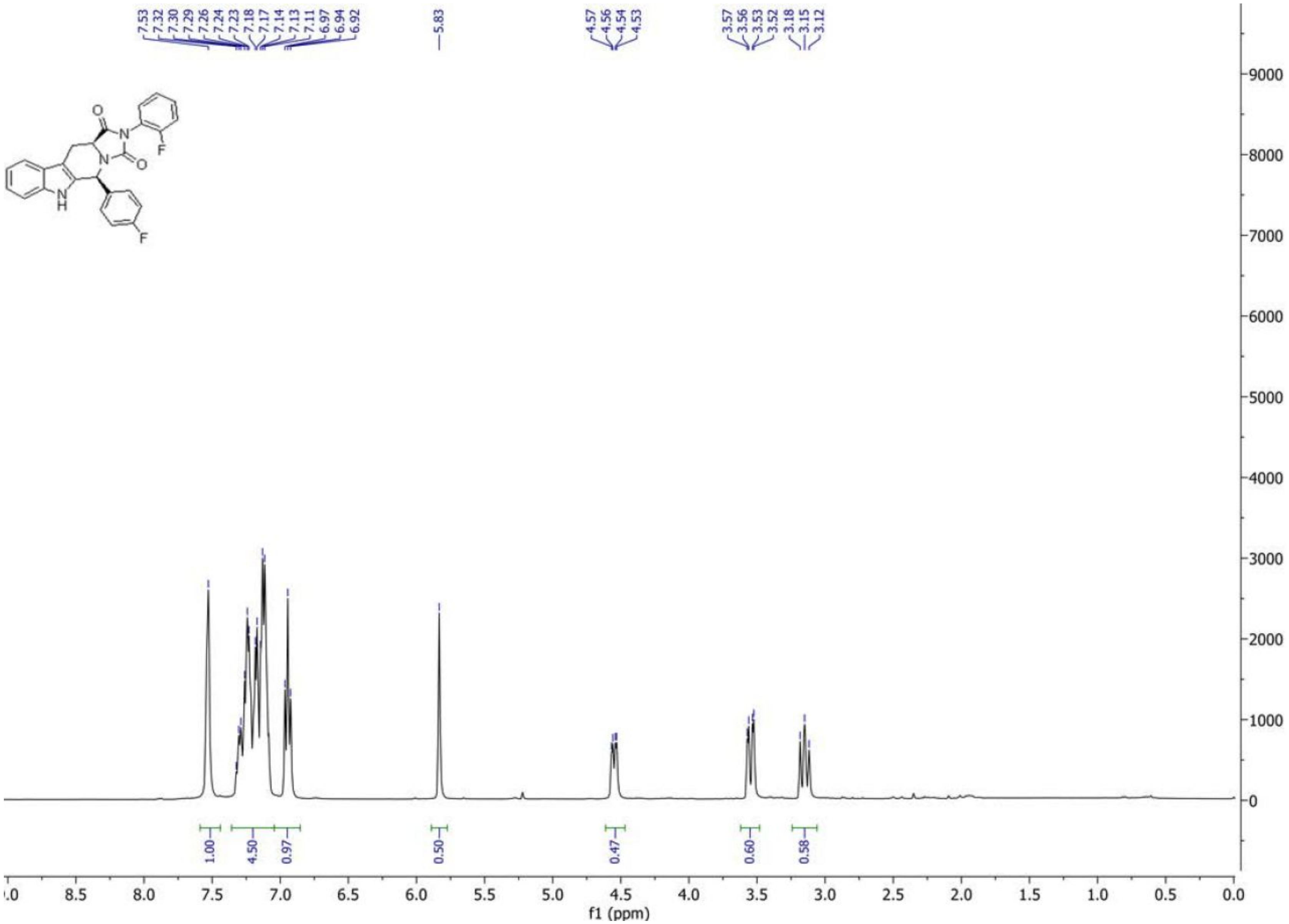

Figure S81: ^{1}H spectra of **37b**

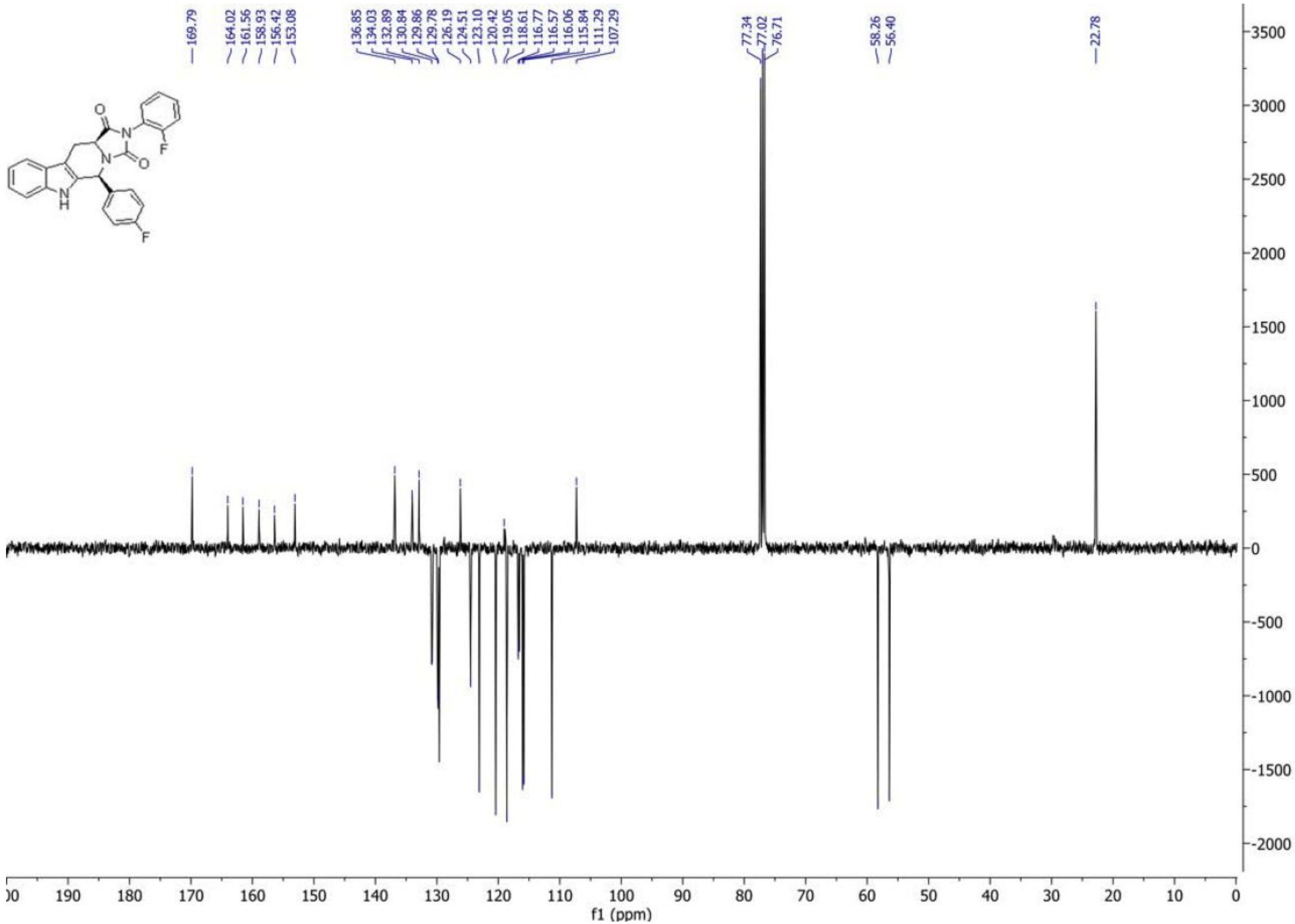

Figure S82: qDEPT spectra of **37b**

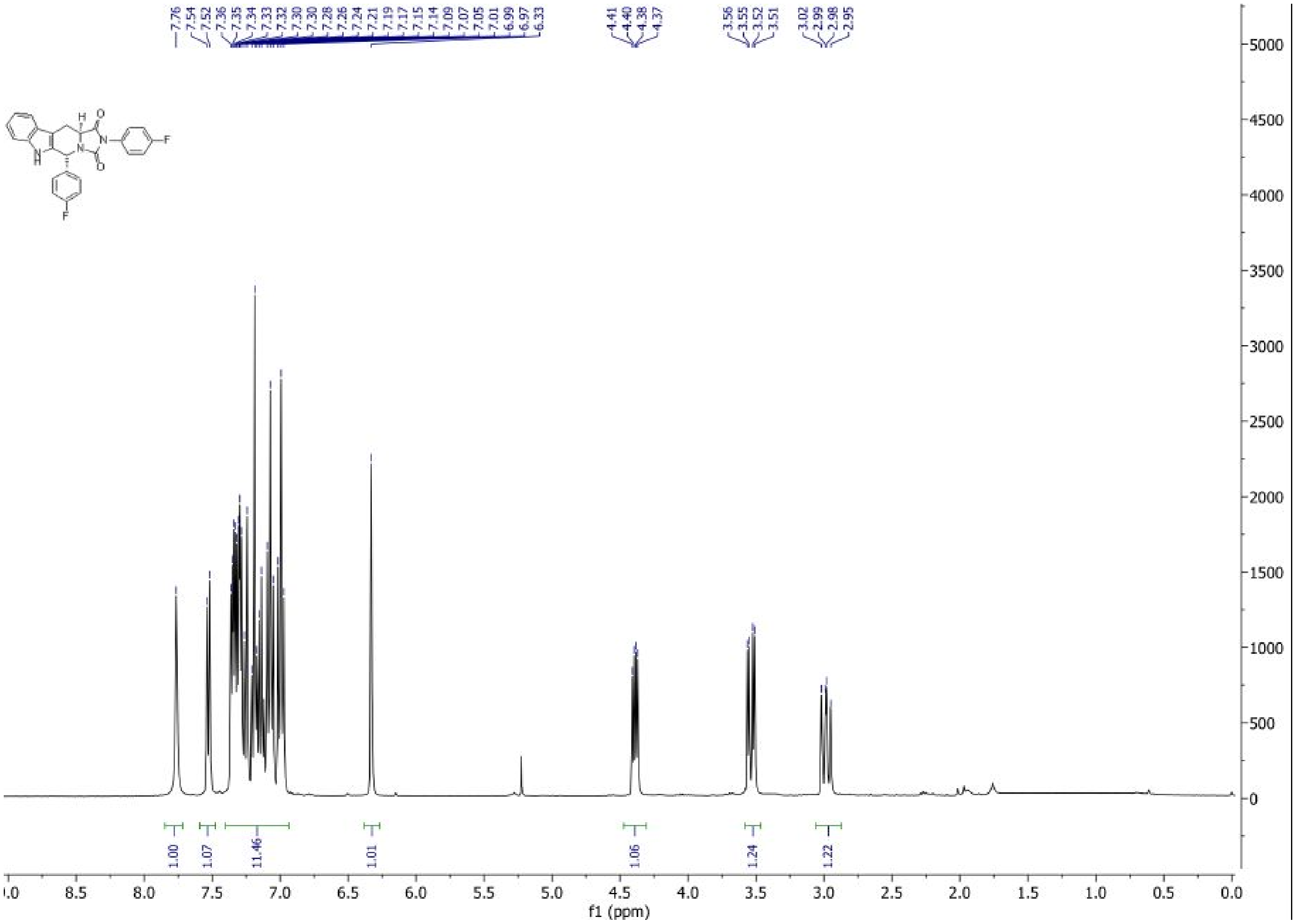

Figure S83: ^{1}H spectra of **38a**

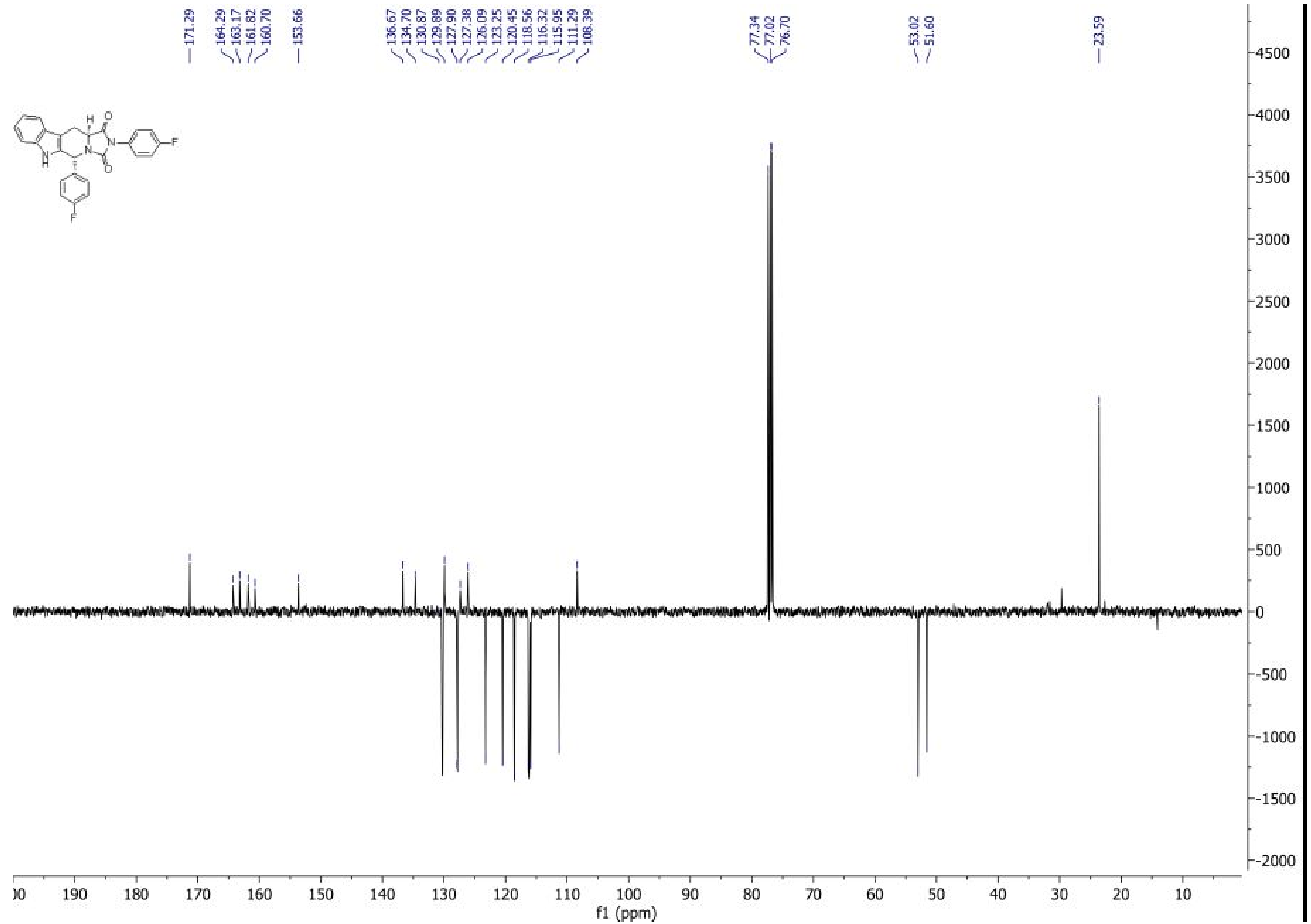

Figure S84: qDEPT spectra of **38a**

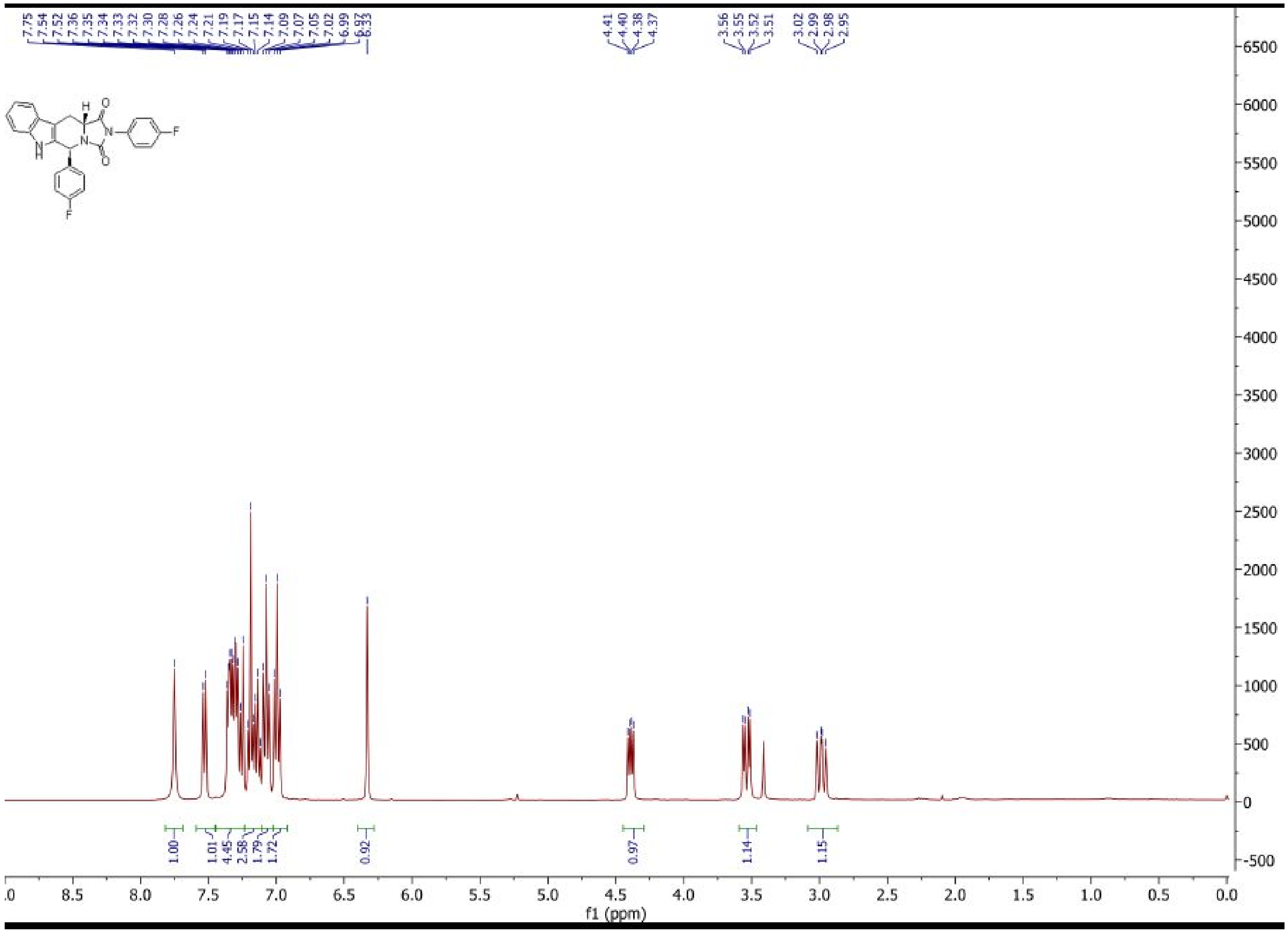

Figure S85: ^{1}H spectra of **38a'**

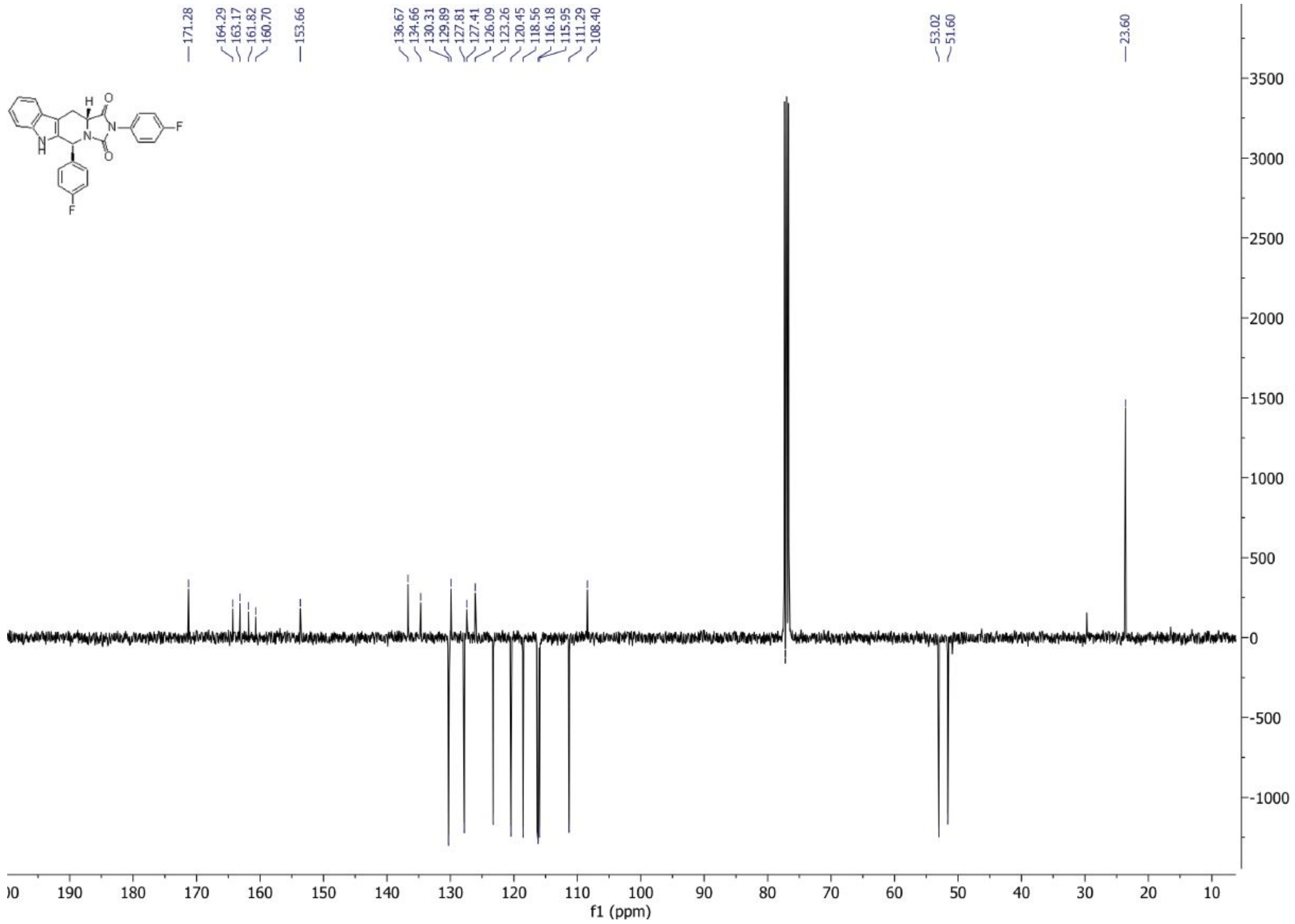

Figure S86: qDEPT spectra of **38a'**

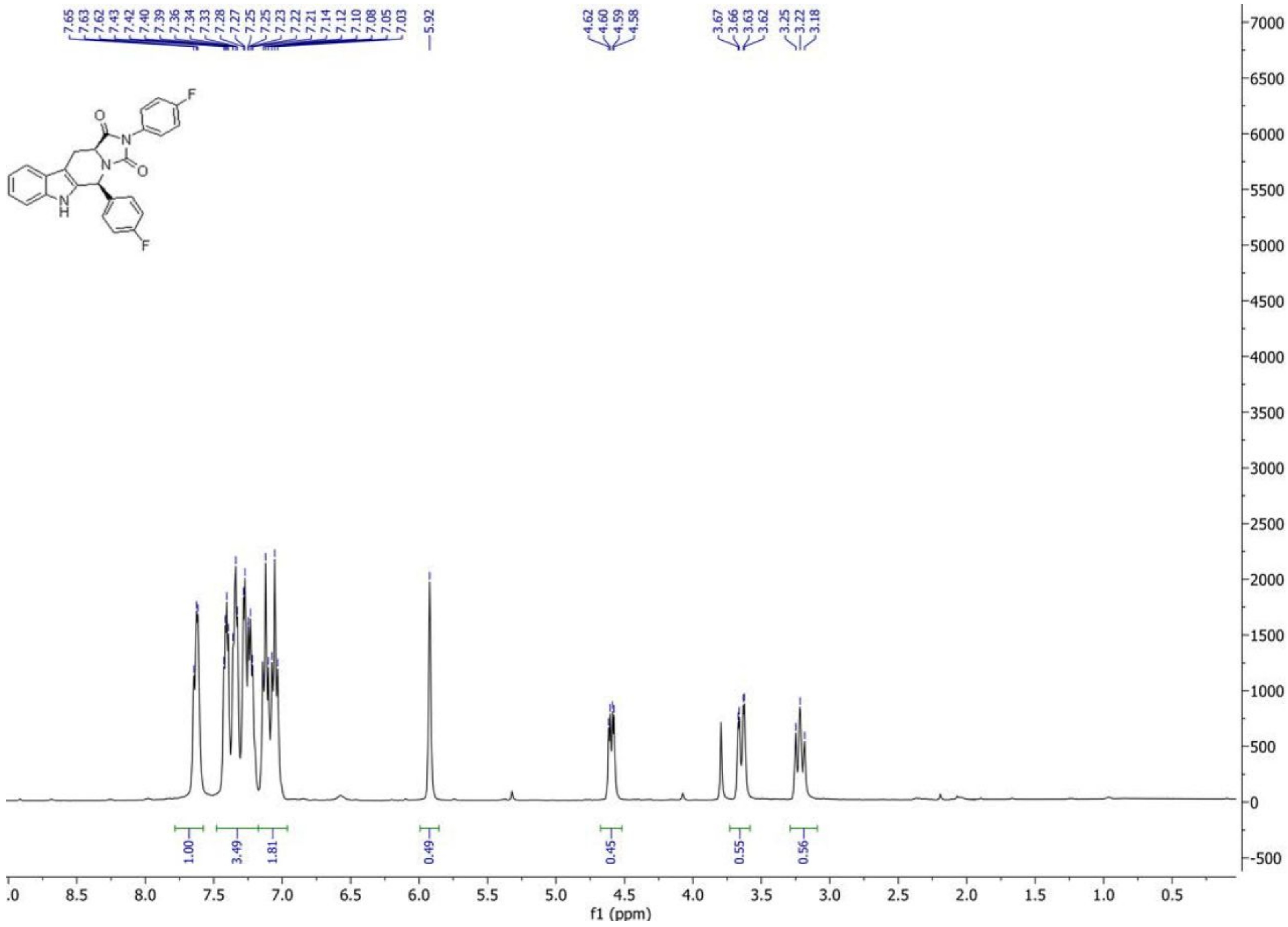

Figure S87: ^{1}H spectra of **38b**

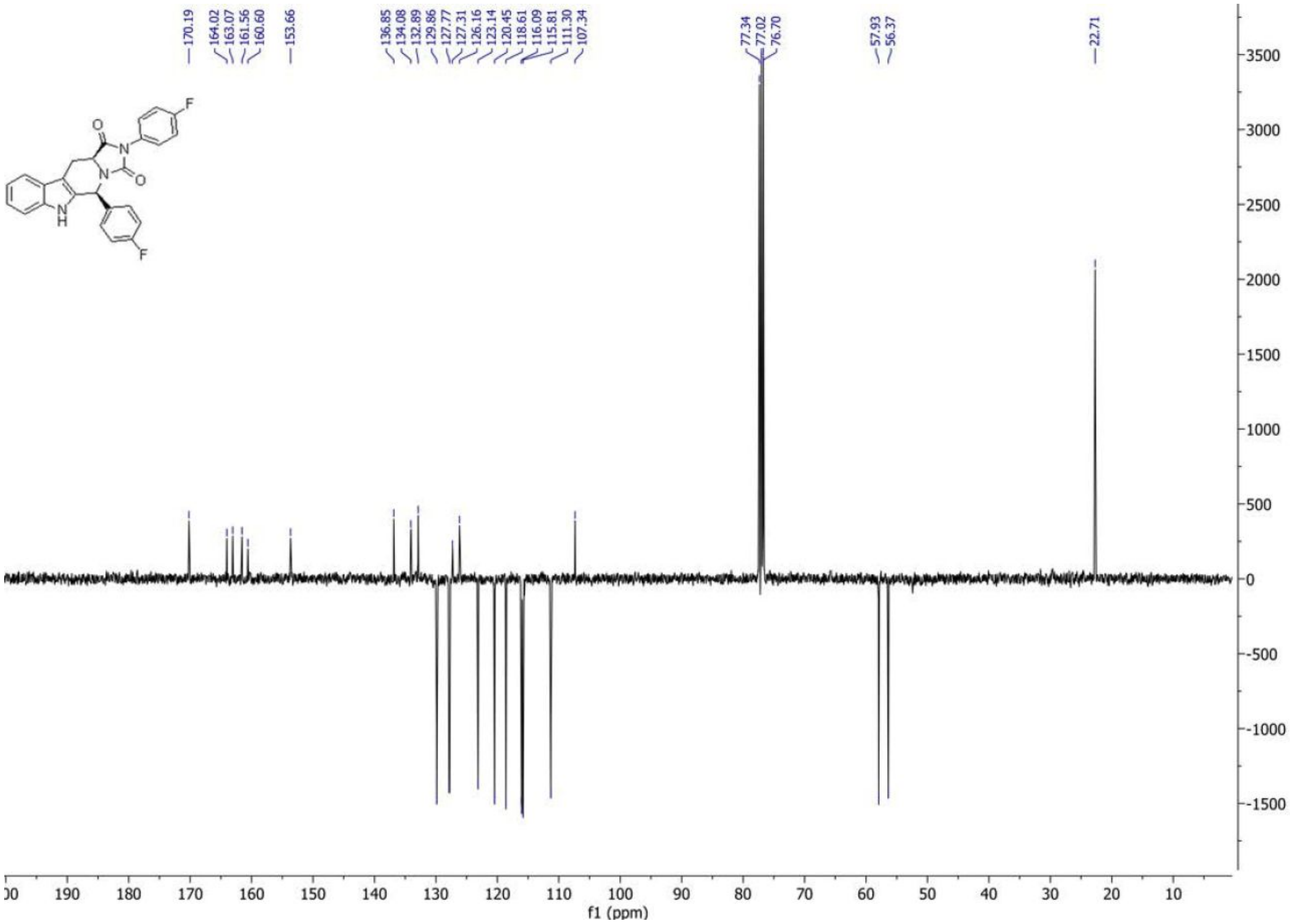

Figure S88: qDEPT spectra of **38a'**

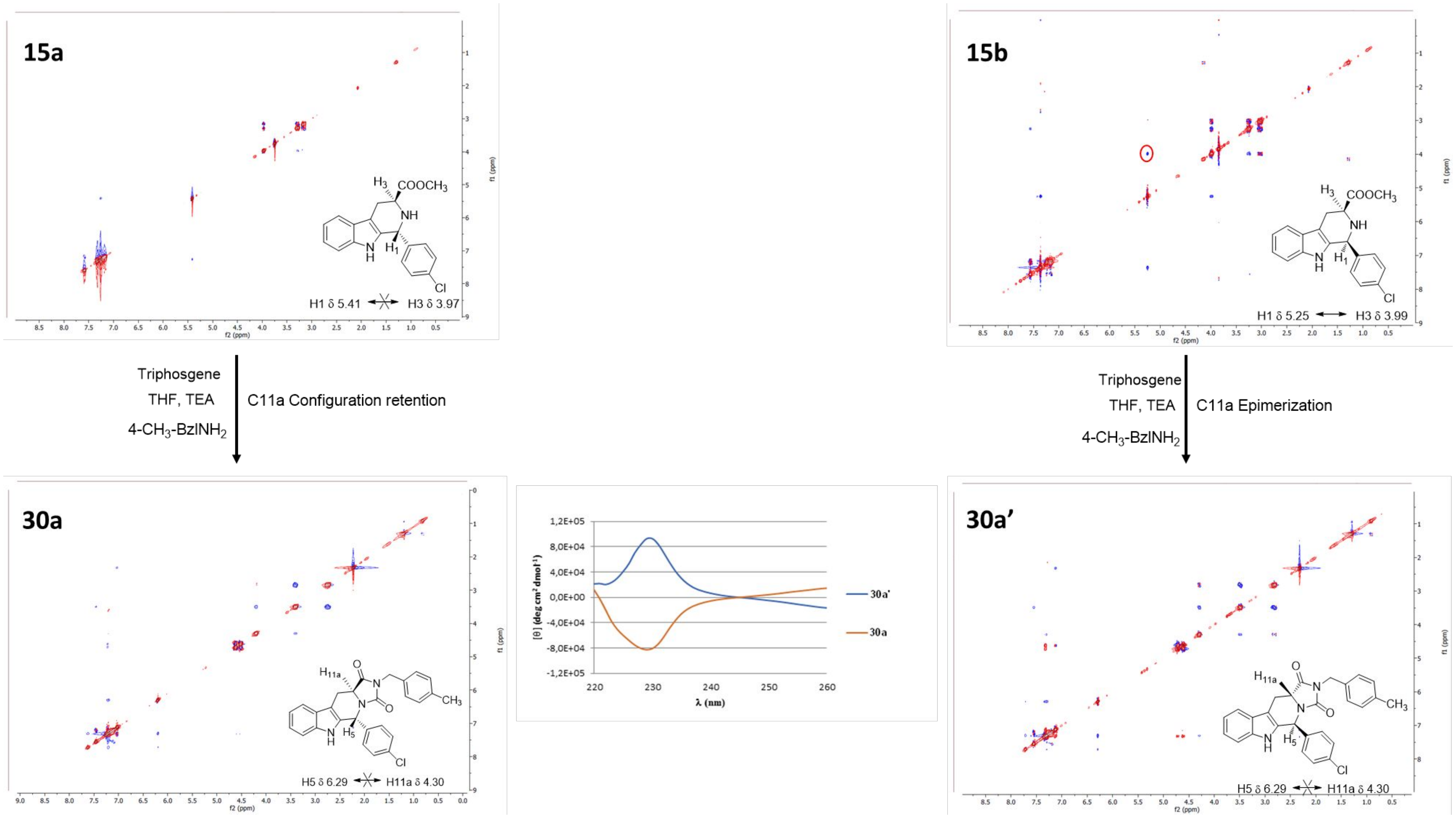

Figure S89: Attribution of the absolute configuration for derivatives **30a** and **30a'**

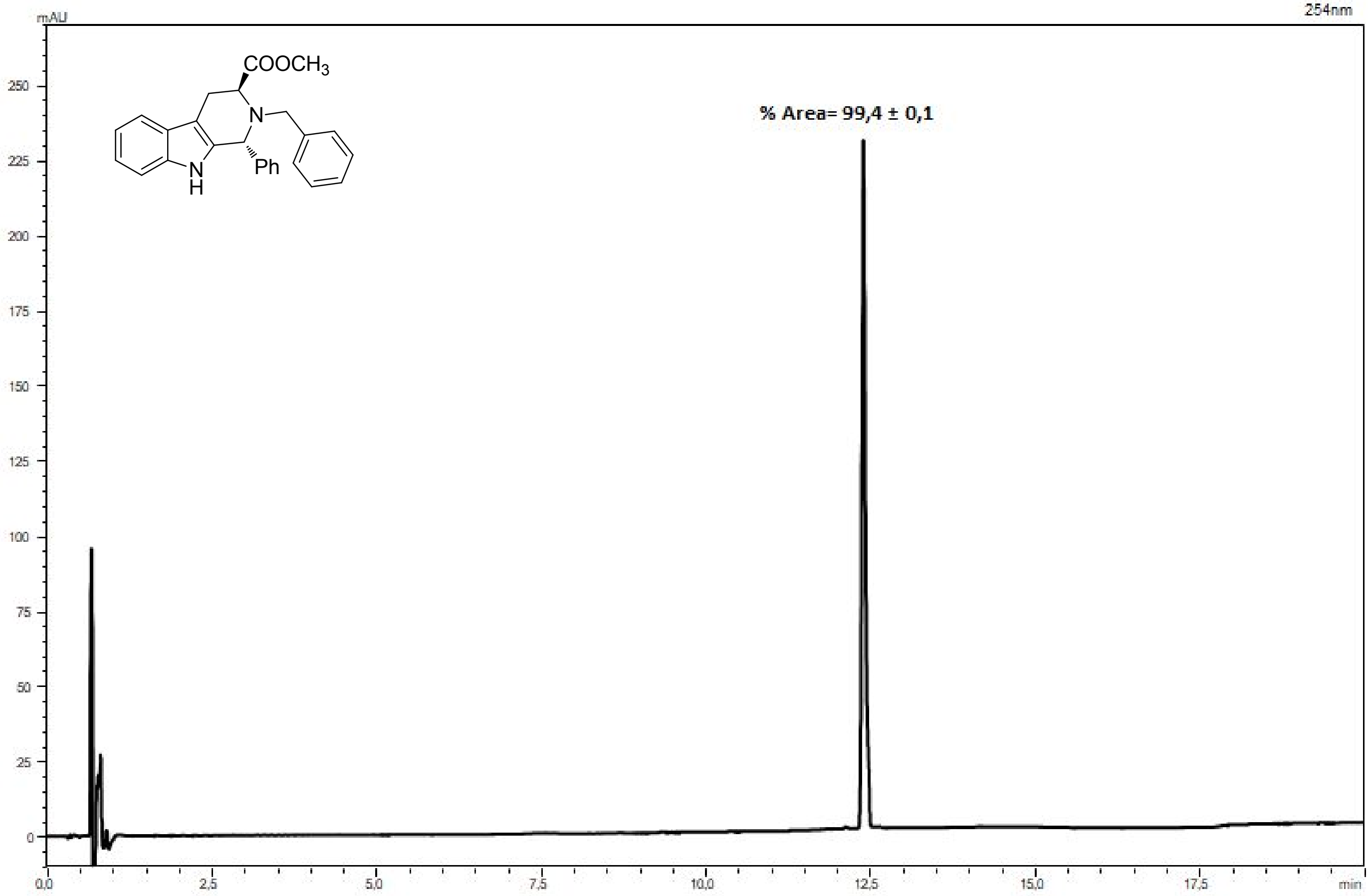

Figure S90: HPLC chromatogram for compound **6a**

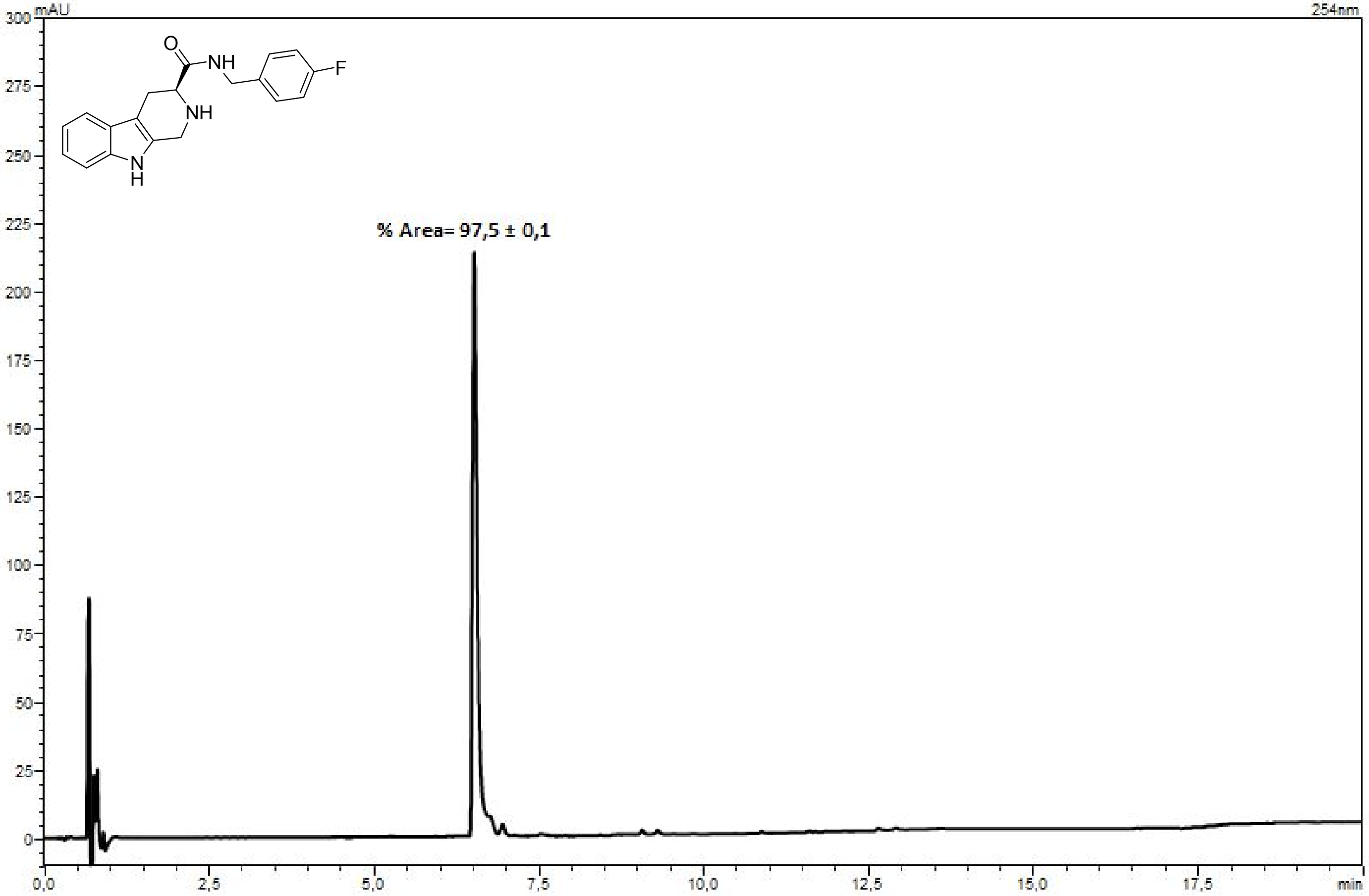

Figure S91: HPLC chromatogram for compound **9**

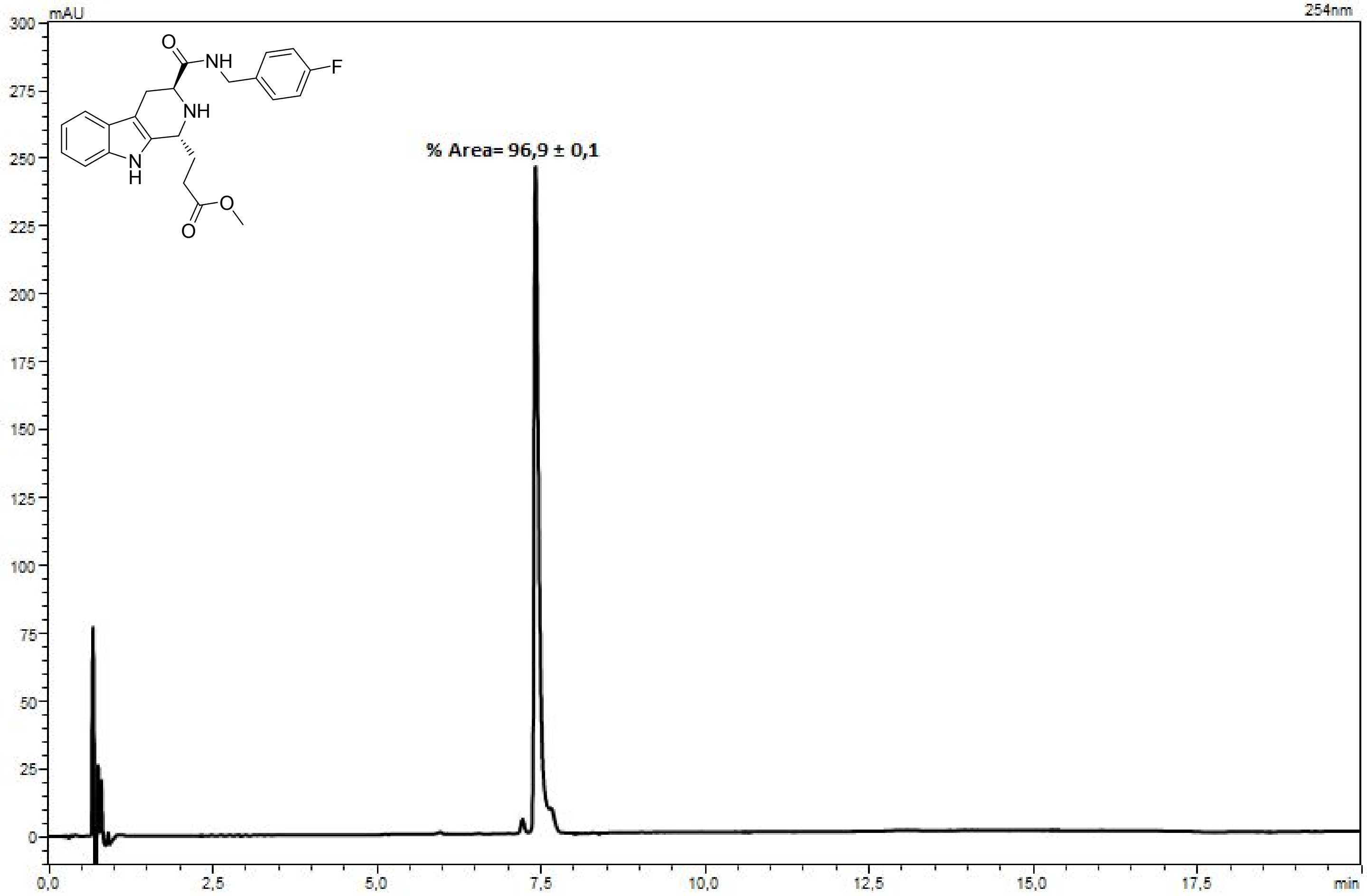

Figure S92: HPLC chromatogram for compound **11a**

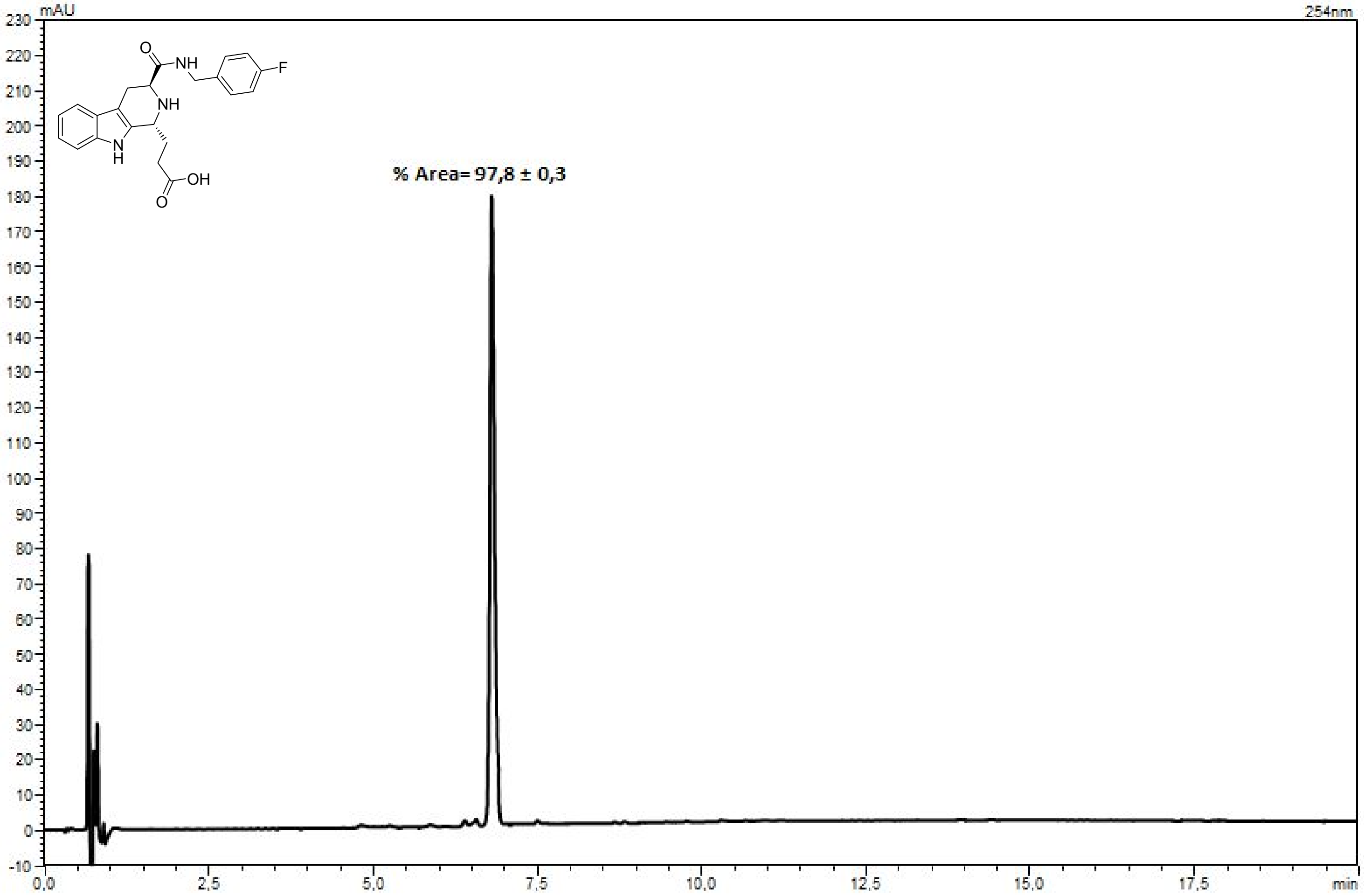

Figure S93: HPLC chromatogram for compound **12a**

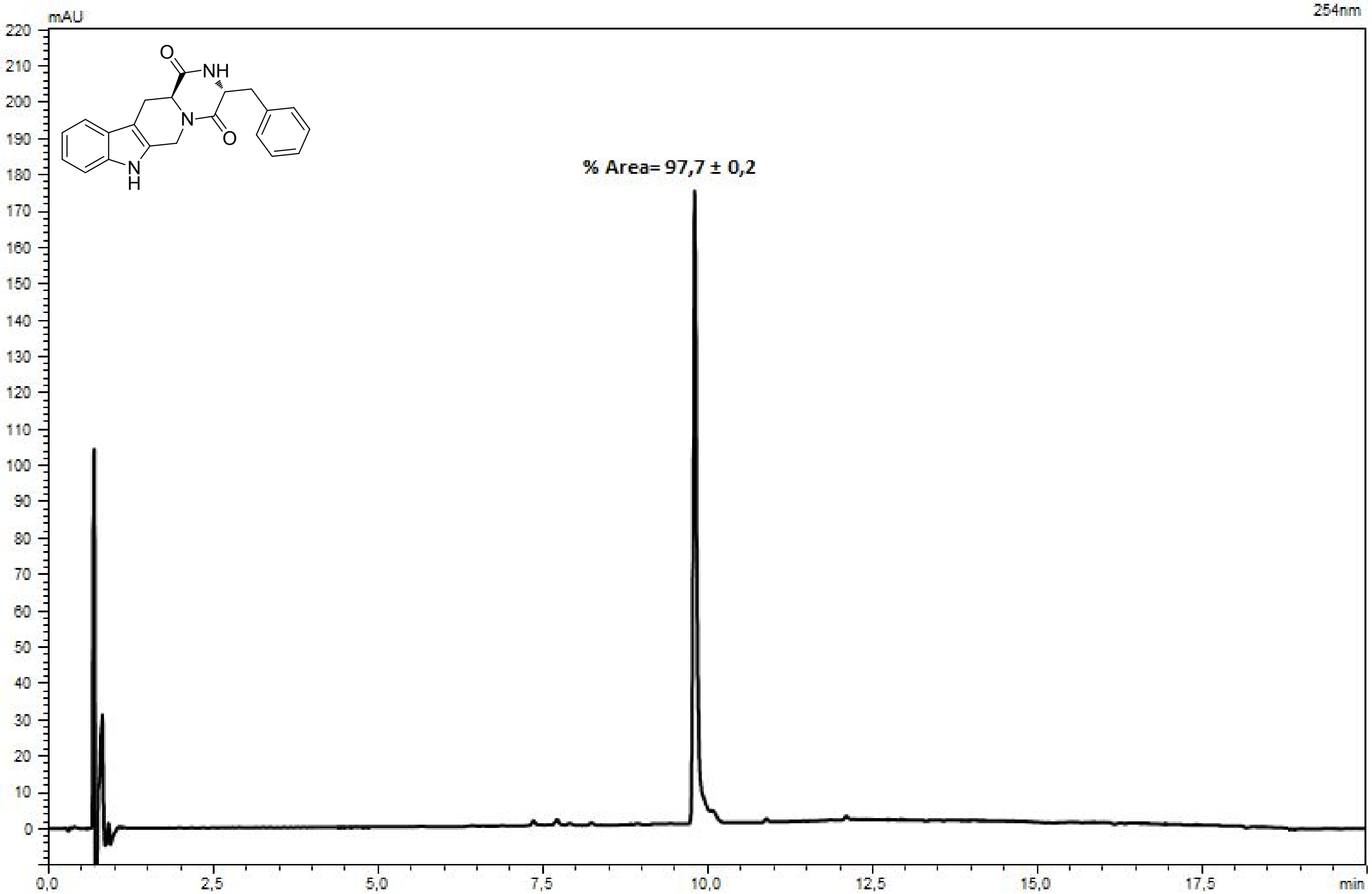

Figure S94: HPLC chromatogram for compound **23**

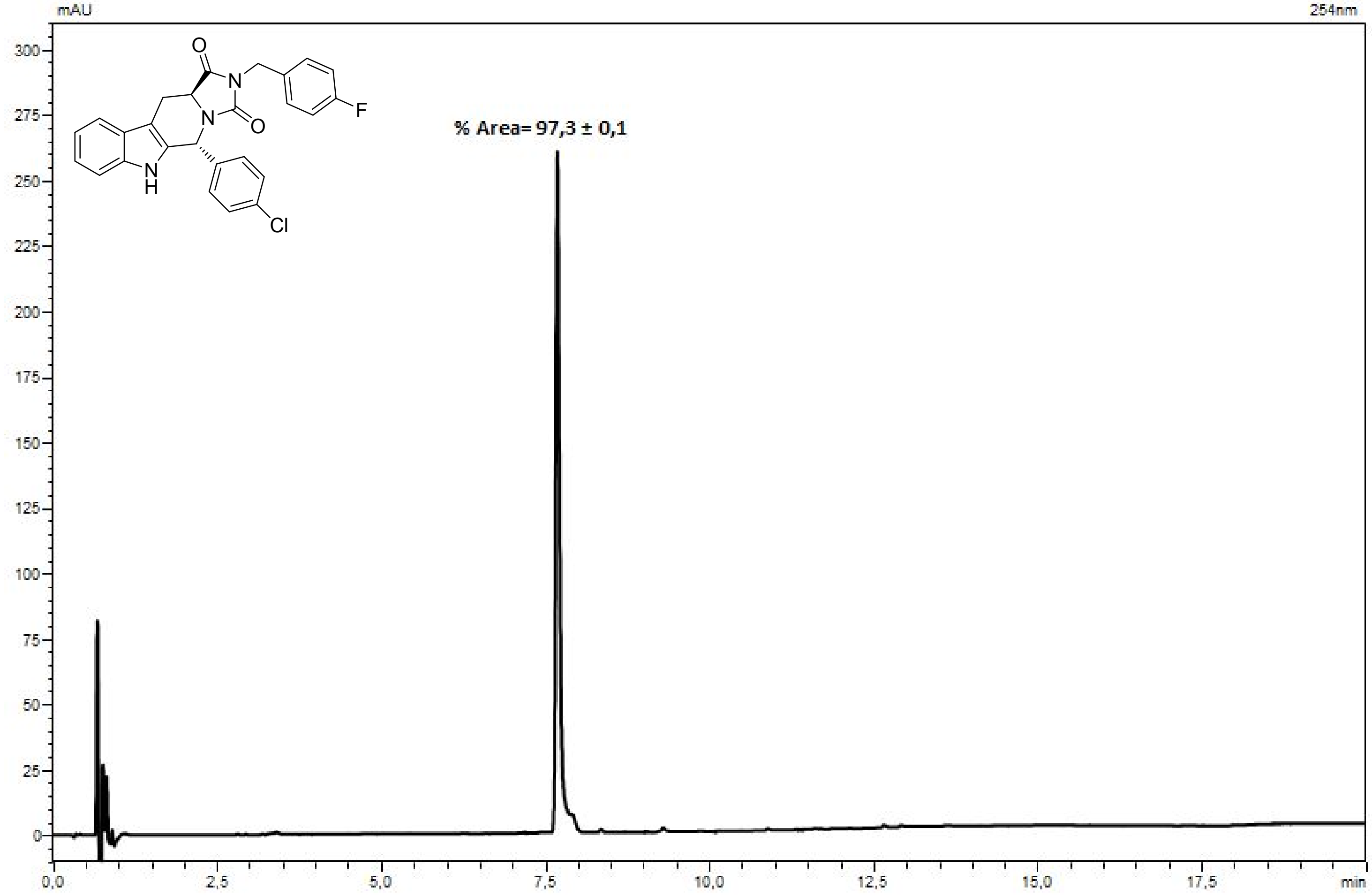

Figure S95: HPLC chromatogram for compound **31a**

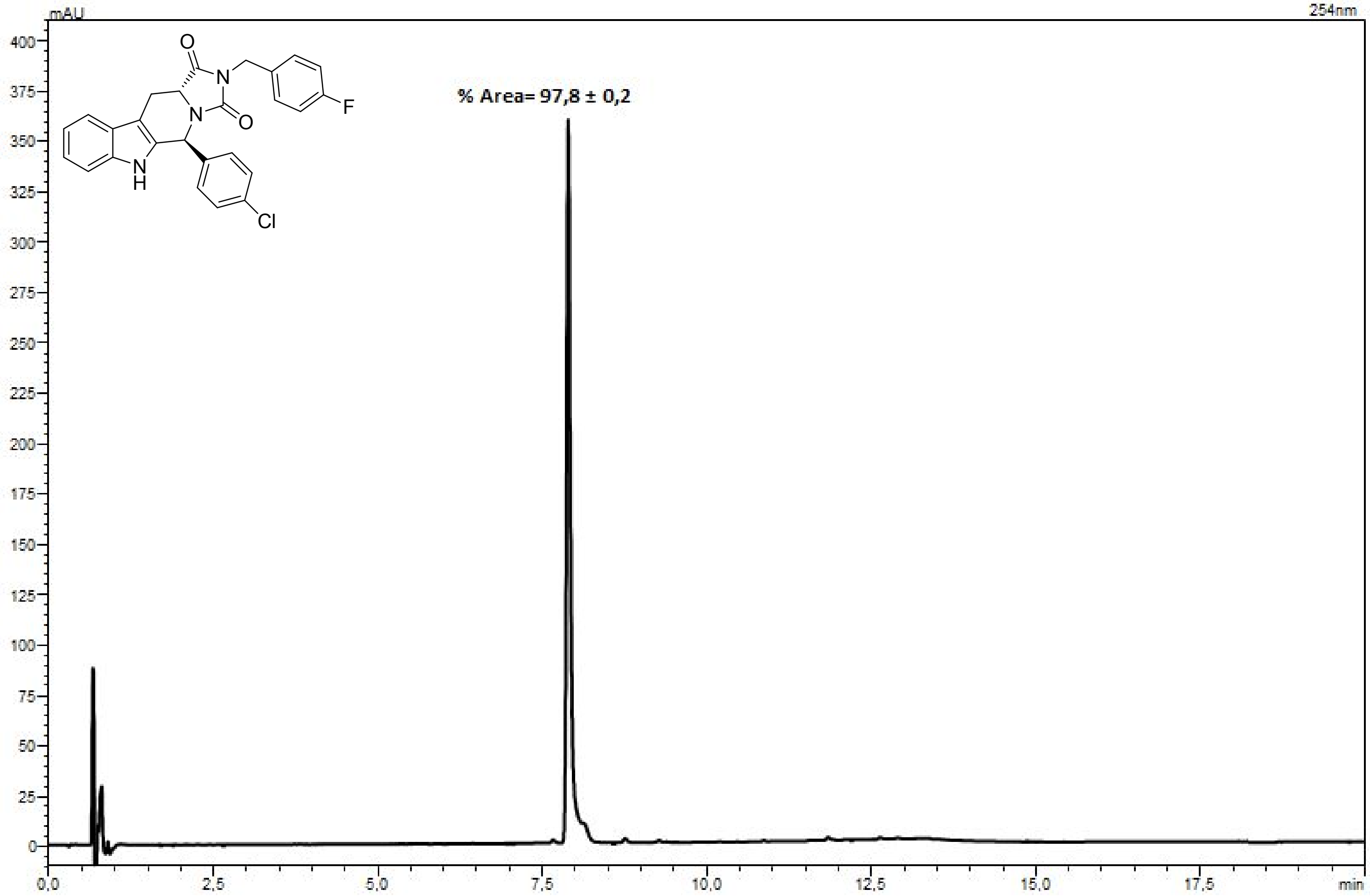

Figure S96: HPLC chromatogram for compound **31a’**

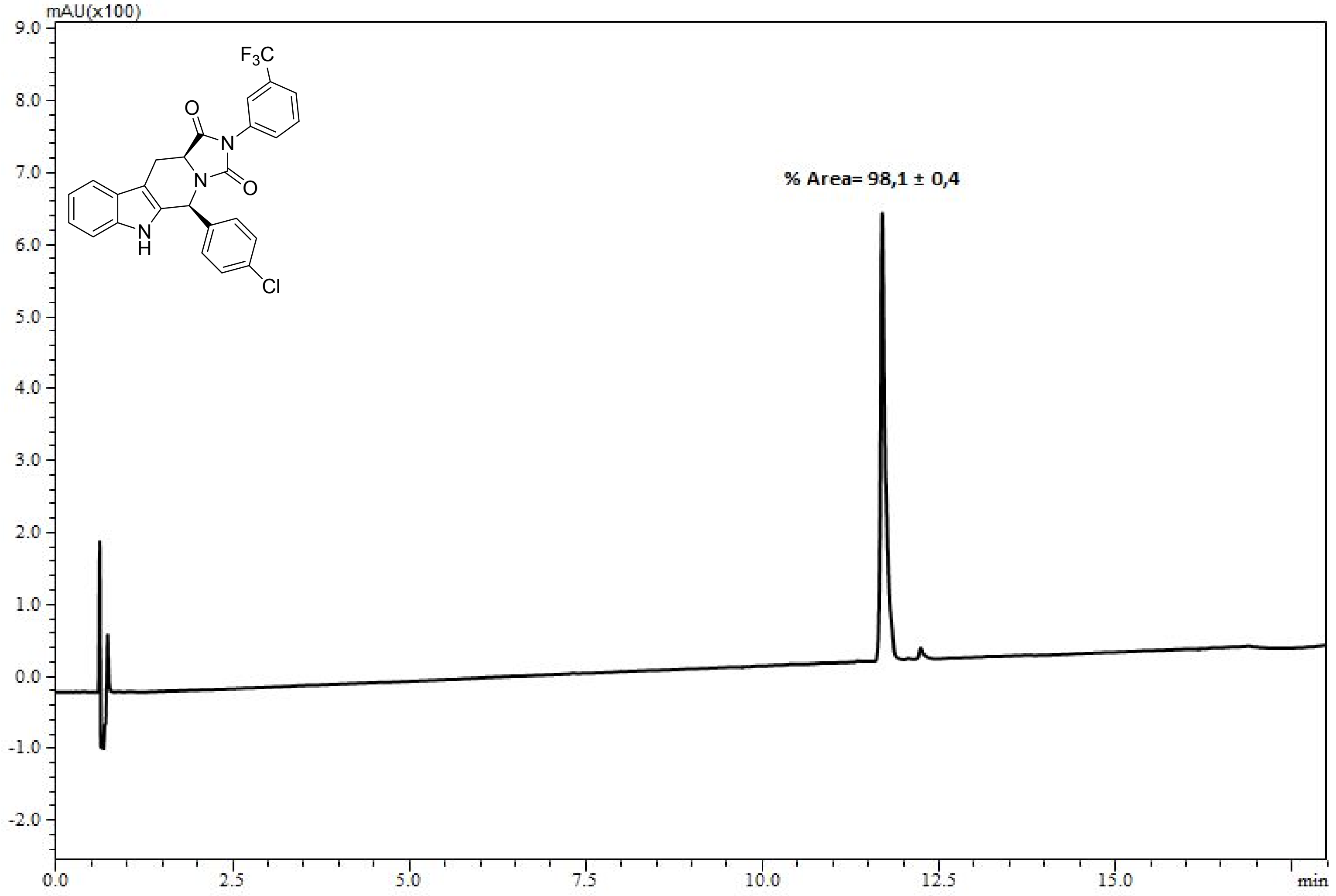

Figure S97: HPLC chromatogram for compound **36b**

Compound	Regression curve (2.5-40 µM)
**4**	y= 0.00004x+ 0.09312; R^2 = 0.9999
**9**	y= 0.00105x – 0.20996; R^2 = 0.9998
**11a**	y= 0.00038x+ 0.48612; R^2 = 0.9998
**12a**	y= 0.00051x+ 0.50935; R^2 = 0.9999
**23**	y= 0.00004x- 0.03730; R^2 = 0.9992
**6a**	y= 0.00007x+ 0.02376; R^2 = 0.9999
**31**	y= 0.00012x– 0.74827; R^2 = 0.9999
**31’**	y= 0.00011x– 1.16989; R^2 = 0.9999
**36’’**	y= 0.00003x+ 0.02555; R^2 = 0.9999

Table S1: Regression curves and R^2 for quantitative UHPLC determination of selected compounds
